# Synthesis of difluoromethylated allenes through trifunctionalization of 1,3-enynes

**DOI:** 10.1038/s41467-019-14254-3

**Published:** 2020-01-21

**Authors:** Munira Taj Muhammad, Yihang Jiao, Changqing Ye, Mong-Feng Chiou, Muhammad Israr, Xiaotao Zhu, Yajun Li, Zhenhai Wen, Armido Studer, Hongli Bao

**Affiliations:** 1 State Key Laboratory of Structural Chemistry, Key Laboratory of Coal to Ethylene Glycol and Its Related Technology, Center for Excellence in Molecular Synthesis, Fujian Institute of Research on the Structure of Matter, University of Chinese Academy of Sciences, 155 Yangqiao Road West, Fuzhou, Fujian, 350002 People’s Republic of China; 2 University of Chinese Academy of Sciences, Beijing, 100049 People’s Republic of China; 3 Organisch-Chemisches Institut, Westfälische Wilhelms-Universität Corrensstraße 40, 48149 Münster, Germany

**Keywords:** Synthetic chemistry methodology, Reaction mechanisms, Stereochemistry

## Abstract

Organofluorine compounds have shown their great value in many aspects. Moreover, allenes are also a class of important compounds. Fluorinated or fluoroalkylated allenes might provide an option as candidates for drug and material developments, as allenes allow a great number of valuable transformations. Herein, we report a metal-free synthesis of difluoromethylated allenes via regioselective trifunctionalization of 1,3-enynes. This method proceeds through double C–F bond formation with concomitant introduction of an amino group to the allene. Synthetic applications are conducted and preliminary mechanistic studies suggest that a two-step pathway is involved. DFT calculations revealed an unusual dibenzenesulfonimide-assisted fluorination/fluoroamination with NFSI. In addition, kinetic reaction study revealed the induction period of both major and side products to support the proposed reaction mechanism. This work offers a convenient approach for the synthesis of a range of difluoromethylated allenes and is also a rare example of trifunctionalization of 1,3-enynes.

## Abstract (web summary)

Fluorinated or fluoroalkylated allenes are versatile building blocks for medicinal and material chemistry. Here, the authors show a regioselective trifunctionalization of 1,3-enynes proceeding through double C-F bond formation and concomitant installation of a -NSO_2_Ph group to the allene moiety.

## Introduction

Organofluorine compounds have shown great importance over the years in the fields such as medicinal chemistry and agrochemistry, as the introduction of fluorine atom(s) onto molecules would modify considerably their chemical and physicochemical properties^1–6^. Thus, methods for the synthesis of organofluorine compounds have been flourished in both profundity and scope^7–13^. The difluoromethyl group (CF_2_H), as an analogue of the well-known trifluoromethyl group (CF_3_), has recently received increasing attentions and many powerful methods for its introduction into organic compounds have been developed^14–19^. On the other hand, allenes are key intermediates in organic synthesis and are important structural motifs that can also be found in natural products^20–24^. For the purpose of providing diverse fluorinated molecules to meet the increasing demand for drug discovery and new materials development, methods for efficient synthesis of various fluorinated or fluoroalkylated allenes are evidently of great value, as such allenes allow a great number of valuable skeletal and stereochemical transformations^21,24^.

Over the past few decades, many difluoromethylation reactions have been successfully developed, enabling facile synthesis of different types of difluoromethylated compounds. Most commonly, the CF_2_H group (or CF_2_R group) is accessed from a CF_2_-containing building block or agent^14–19,25–31^. For example, the syntheses of difluoromethylated allenes typically require the use of difluoromethyl metal compounds^32–35^. Recently, Wang et al. developed the nickel-catalyzed carbofluoroalkylation affording difluoroalkylated allenes using ethyl bromodifluoroacetate^36^. Notwithstanding these significant breakthroughs, the development of efficient and diversified approaches for the facile construction of difluoromethylated allenes is still challenging especially starts from non-CF_2_-containing building blocks and methods for the synthesis of difluoroalkylated allenes are still demanded.

**Fig. 1:**
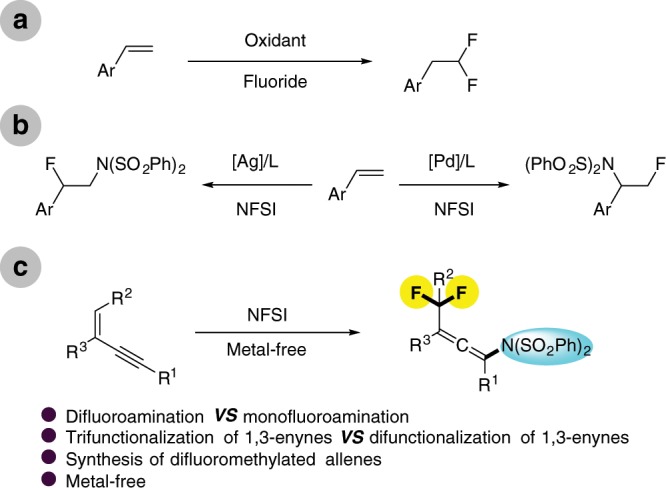
Fluorination or difluorination of C=C bonds. **a** *Gem*-difluorination of styrenes. **b** Fluoroamination of styrenes. **c** This work: facile synthesis of difluoromethylated allenes.

Inspired by earlier reports on *gem*-difluorination (Fig. 1a)^37–40^ and fluoroamination of styrenes (Fig. 1b)^41–45^, also as one part of our continuous efforts on functionalization of unsaturated compounds^46–49^, herein, we report a metal-free consecutive trifunctionalization of 1,3-enynes for the assembly of various difluoromethylated allenyl amines (Fig. 1c) using NFSI as the fluorination and amination source^41,44,50–59^. The synthetic potential of difluoromethylated allenes is demonstrated by transformations to a variety of difluoromethylated compounds^21^.

## Results

### Reaction optimization

We commenced reaction condition optimization using CuTC as the catalyst and 1,10-phenanthroline as the ligand. As shown in Table 1, the reaction between 1,3-enyne **1a** and NFSI was originally thought to involve intermolecular 1,4-fluoroamination, providing the monofluoromethylated allene **3a** as the desired product^49^. However, only a trace amount of **3a** was detected, and a new compound, the difluoromethylated allene **4a**, was obtained as major product albeit in a low yield (Table 1, entry 1). Formation of the product **4a** alluded to a previously unknown reaction, and we therefore decided to optimize the conditions towards formation of **4a**. With the Pd(OAc)_2_/BC catalyst system^45^, product **4a** was obtained in 34% yield (Table 1, entry 2). Use of a CoCl_2_ catalyst failed to provide any targeted **4a** (Table 1, entry 4), while Pt(COD)Cl_2_ and NiCl_2_ showed a better performance than the Pd(OAc)_2_/BC catalyst system (Table 1, entry 5 v. entry 6 v. entry 2). After extensive screening experiments, it was found that the reaction of the 1,3-enyne **1a** with NFSI afforded **4a** in 28% yield, even in the absence of catalyst and ligand (Table 1, entry 7). The yield of **4a** was further improved to 44% upon running the reaction at an elevated temperature (Table 1, entry 8). Other solvents were screened and it was found that toluene and chloroform provide the product **4a** in 66 and 61% yield (Table 1, entries 9 and 10), while acetonitrile, a polar solvent, afforded the targeted product in only 12% yield (Table 1, entry 11). Furthermore, the yield of **4a** dropped when the reaction was carried out in toluene at 100 ^o^C (Table 1, entry 12). Notably, a small amount of the monofluoromethylated allene **3a** was observed in all these reactions.

**Table 1:** Reaction condition optimization.


Entry	Cat.	Ligand	Solvent	Yield of 4a (%)^a^
1	CuTC	1,10-Phen	DCM	<5
2	Pd(OAc)_2_	BC	DCM	34
3	PdCl_2_	1,10-Phen	DCM	24
4	CoCl_2_	1,10-Phen	DCM	trace
5	Pt(COD)Cl_2_	1,10-Phen	DCM	40
6	NiCl_2_	1,10-Phen	DCM	48
7	–	–	DCM	28^b^
8	–	–	DCM	44^c^
9	–	–	toluene	66^c^ (65)^d, e^
10	–	–	CHCl_3_	61^c^
11	–	–	CH_3_CN	12^c^
12	–	–	toluene	51^f^

Reaction conditions: 1,3-enyne (**1a**, 0.25 mmol), NFSI (0.75 mmol), cat (10 mol%), ligand (10 mol%), solvent (0.5 mL), 65 ^o^C, under a N_2_ atmosphere

*1,10-Phen* 1,10-phenanthroline, *BC* bathocuproine

^a1^H NMR yield

^b^The reaction was performed at 60 ^o^C

^c^The reaction was performed at 80 ^o^C

^d^Isolated yield in parentheses

^e^The ^1^H NMR yield of **3a** is 8%

^f^The reaction was performed at 100 ^o^C

### Substrate scope

**Fig. 2:**
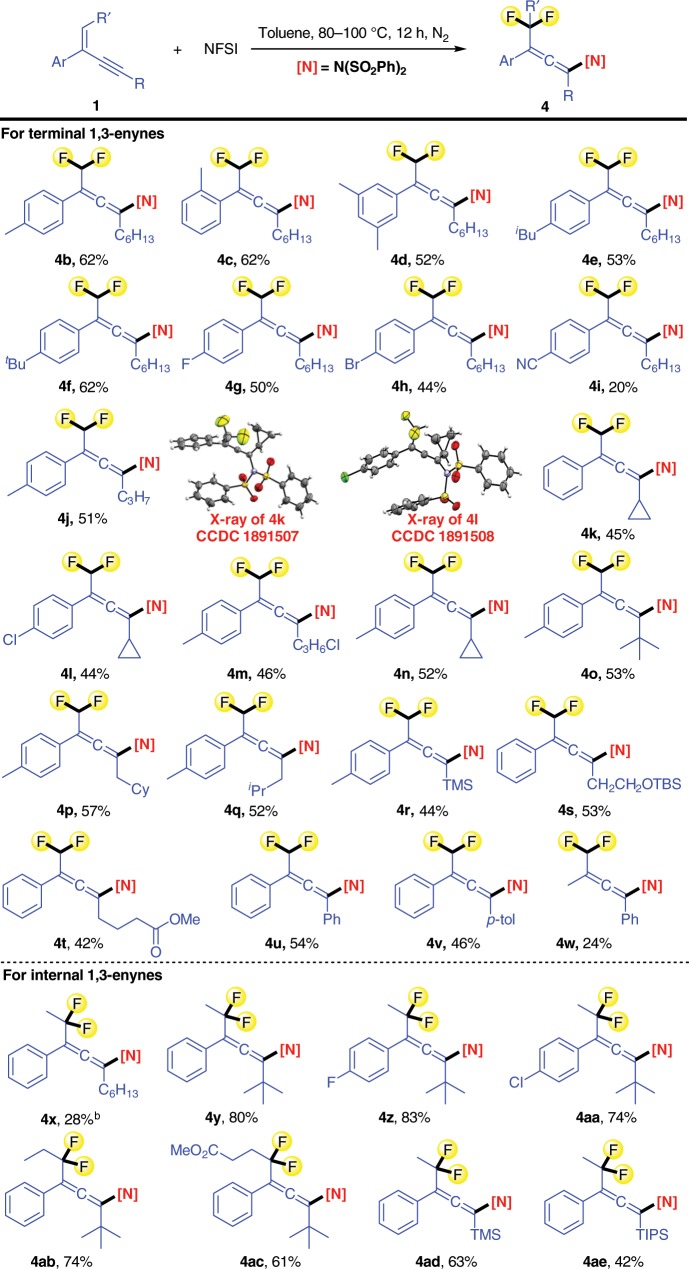
Substrate scope of 1,3-enynes. Reaction conditions: 1,3-enyne (**1**, 0.5 mmol), NFSI (1.5 mmol), toluene (1 mL), 80-100 ^o^C, N_2_ atmosphere, Isolated yield. ^a^With CHCl_3_ (1 mL) as the solvent. ^b^The reaction was performed in cyclohexane and the monofluoromethylated allene **3x** was obtained in 46% yield.

With the optimal reaction conditions in hand, the substrate scope was studied and various difluoromethylated allenes were successfully prepared in moderate to good yields. As shown in Fig. 2, the phenyl group at position 2 can be replaced by a substituted phenyl group (**4b**-**4v**). Electron-donating groups on the benzene ring, such as a methyl group, an *iso*-butyl group, or a *tert*-butyl group, and electron-withdrawing substituents such as Br, F, and CN on the benzene ring are tolerated. Enynes with primary, secondary, or tertiary alkyl groups linked with the C–C triple bond could also be diversified. As examples, 1,3-enynes with a *n*-hexyl group (**1b**-**1i**), a cyclopropyl group (**1k**, **1l**, and **1n**), a chloroalkyl group (**1m**), or a *tert*-butyl group (**1o**) are suitable substrates for the direct difluoroamination reaction. 1,3-Enynes bearing a silyl group (**1r**), a silyl ether moiety (**1s**), or an ester group (**1t**) also engage in this reaction. Moreover, 2,4-diaryl substituted 1,3-enynes can be used as the substrates to afford the corresponding products in moderate yields (see **4u** and **4v**). However, (3-methylbut-3-en-1-yn-1-yl)benzene (**1w**), as an example for non-styrene systems, only provided the desired product **4w** in 24% yield, which might be due to the less stability of the intermediate compared to that of 1-alkynyl styrenes. The structures of the difluoromethylated allenes **4k** and **4l** were unambiguously confirmed by single crystal X-ray diffraction.

Encouraged by the results using terminal 1,3-enynes, we next investigated difluoroamination of internal 1,3-enynes aiming at the preparation of difluoroalkylated allenes. With 1,3-enyne **1x**, bearing a primary alkyl group linked with the C–C triple bond, only a 28% yield of the product **4x** was obtained, and the accompanying monofluoroamination product **3x** was produced in 46% yield as major compound. However, it was found that the substrates bearing a bulky group at the triple bond deliver the corresponding products **4y**-**4ae** in higher yields and functionalities such as the ester group (**4ac**) and silyl groups (**4ad** and **4ae**) are tolerated.

### Synthetic applications

**Fig. 3:**
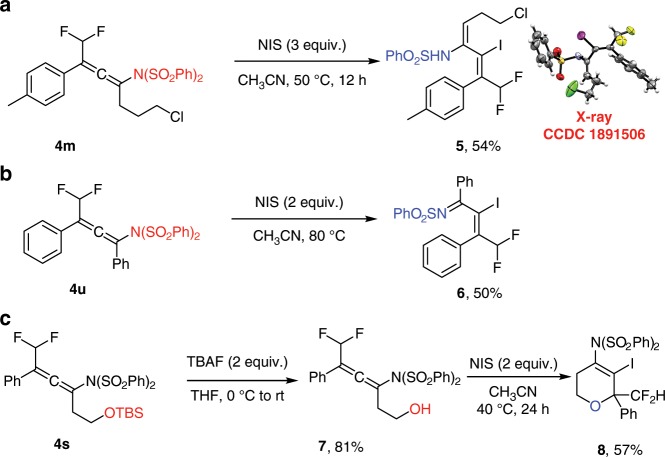
Synthetic applications. **a** Derivatizations of difluoromethylated allene **4m**. **b** Derivatizations of difluoromethylated allene **4u**. **c** Synthesis of multisubstituted 3,6-dihydro-2*H*-pyran **8** from difluoromethylated allene **4s**.

Synthetic applications of this consecutive difluoroamination of 1,3-enynes have been demonstrated and are shown in Fig. 3. In the presence of *N*-iodosuccinimide (NIS), the difluoromethylated allene **4m** was regioselectively transformed into the (*Z*,*Z*)-diene **5** in 54% yield. Note that during iodination one of the sulfonyl groups of the -N(SO_2_Ph)_2_ functionality was removed. Interestingly, when the allene **4u** bearing two phenyl groups was subjected to the reaction with NIS, the difluoromethylated vinyl imine **6** was formed. Such vinyl iodides, **5** and **6**, are also pivotal building blocks for further transformations^60^. Moreover, the silyl group of the allene **4s** could be readily removed to afford the allene **7** with a free alcohol group (81%), which could be further cyclized in 57% yield to the multisubstituted 3,6-dihydro-2*H*-pyran **8**.

### Mechanistic studies

**Fig. 4:**
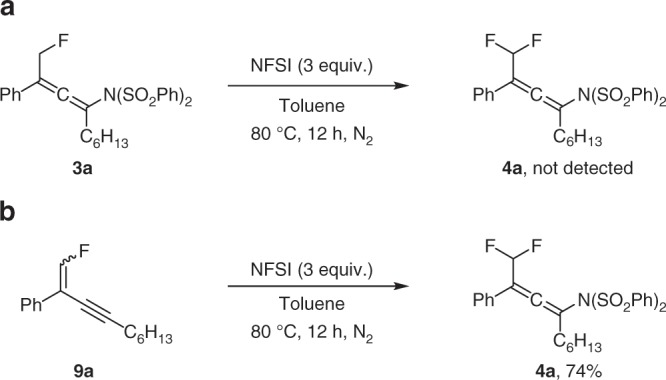
Mechanistic studies. **a** Test of **3a** as the possible reactive intermediate. **b** Test of **9a** as the possible reactive intermediate.

Finally, preliminary experiments to probe the mechanism of the difluoromethylated allene formation reaction were performed. Since the fluoroamination product **3a** was found as a side product (see Table 1), **3a** was supposed to be the reaction intermediate and was therefore subjected to the standard conditions. However, the desired product **4a** was not detected (Fig. 4a). Notably, during reaction optimization the fluorinated 1,3-enyne^61–68^ **9a** was observed. In addition, treatment of the 1,3-enyne **1a** with a reduced amount of NFSI afforded compound **9a** along with targeted **4a**. Therefore, we suspected that **9a** might be a key intermediate in this cascade. Indeed, when compound **9a** was subjected to the reaction with NFSI, product **4a** was isolated in 74% yield (Fig. 4b), suggesting that **9a**, rather than **3a**, is a possible intermediate for the difluoroamination and this reaction probably relies on a two-step process.

### DFT study

On the other hand, DFT study was also performed according to the experiments of mechanism studies. Initially, three kinds of 1,3-enyne radical cation species oxidized by NFSI were considered, but the enthalpies were too high (ΔH > 57 kcal/mol) to support the cation radical process (Supplementary Fig. 1)^44^.

We then focus on the allenyl cation pathways. Two-component reactions of an 1,3-enyne with NFSI were investigated in different orientations of NFSI to transfer the fluoride to 1,3-enyne, **1**. Two transition states, **TS1** and **TS1’**, were located leading to the reactive intermediate **9** with a dibenzenesulfonimide (DBSI) moiety synchronously and the 1,2-adduct *N*-(1-fluoro-2-phenylhept-3-yn-2-yl)-*N*-(phenylsulfonyl)benzenesulfonamide (**10**), with activation free energy of 25.1 and 31.1 kcal/mol, respectively (Supplementary Fig. 2), in which producing DBSI via **TS1** is a concerted process after fluorine transfer. Intrinsic reaction coordinate (IRC) calculations have been conducted for the validity of concerted proton transfer and 1,2-addition from **TS1** and **TS1’**, respectively (Supplementary Figs. 3 and 4). In addition, **TS6**, the transition state corresponding to the bisphenylsulfonyl imide moiety of NFSI directly addition to the 4 C atom of **1**, was calculated as well. However, the activation energy of **TS6** is so much higher (59.4 kcal/mol), and it results in a 3,4-addition compound *N*-(3-fluoro-2-phenylhepta-1,3-dien-4-yl)-*N*-(phenylsulfonyl)benzenesulfonamide (**11)** (Supplementary Fig. 2) which was confirmed by IRC calculation as well as the calculations from **TS1** and **TS1’**. It is worth noting that no transition state corresponding to 1,4-addition to form desired product **4** can be located. This is reasonable because the bond distance of N-F of the optimized NFSI molecule is 1.40 Å much less than the distance between 1 C and 4 C in the optimized structure of **1** (*d*(1C-4C) = 3.54 Å).

**Fig. 5:**
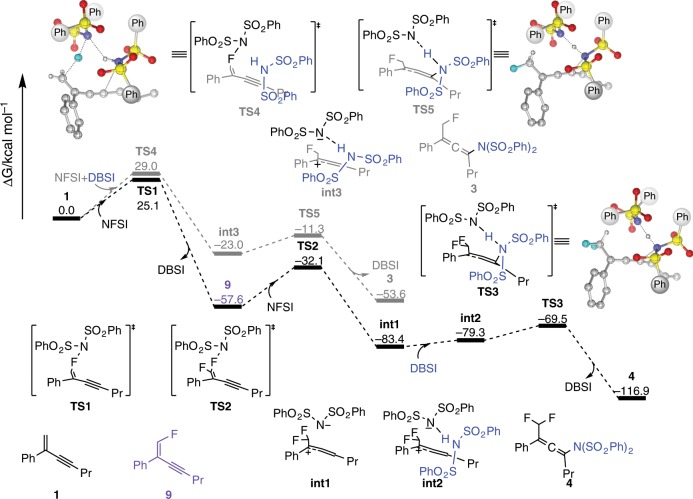
DFT study. The free energy profile of formations of fluoroamination and difluoroamination products, **3** and **4**.

Since the product (*N*-(1,1-difluoro-2-phenyldeca-2,3-dien-4-yl)-4-methyl-*N*-tosylbenzenesulfonamide, **12**) adducted by N(Ts)_2_ can be observed in presence of the HN(Ts)_2_ (see crossover reaction in Supplementary Methods), three-component reactions of 1,3-enyne with NFSI and DBSI were further considered and discussed. Figure 5 shows the overall potential energy surface from **1** to produce the 1,4-addition major product **4** and minor product **3**. As described above, **1** reacts with NFSI first to form the reactive intermediate **9** with the DBSI through the lowest energetic transition state **TS1**. Then, the reactive intermediate **9** reacts with a second NFSI to form an ion pair of difluoromethylallenyl cation and bisphenylsulfonyl imide anion (**int1**) through **TS2** with the energy barrier of 25.5 kcal/mol. Subsequently, an adjacent DBSI, generated from the first step, participates in the reaction to suspend the 2-addition of bisphenylsulfonyl imide anion to difluoromethylallenyl cation. A low barrier transition state **TS3** (13.9 kcal/mol) corresponding to simultaneous imide addition‒proton transfer was then located to form the product **4** and to regenerate a DBSI.

On the other hand, the pathway of three-component reaction combining **1** and NFSI with DBSI to deliver the minor product **3** was also calculated. A three-component transition state of fluorine transfer, **TS4**, was located with the energy barrier of 29.0 kcal/mol which is higher than that of **TS1** but lower than that of **TS1’**. As expectation, an intermediate **int3** was then conducted similar to **int2** in which the DBSI suspends the proton abstraction and the 2-addition of bisphenylsulfonyl imide anion. Subsequently, transition state **TS5** corresponding to simultaneous imide addition‒proton transfer can also be located with 11.7 kcal/mol of barrier resulting in the monofluoroamination product **3** and regenerating a DBSI. It is noteworthy that the barrier of **TS4**, 3.9 kcal/mol higher than that of **TS1**, may be somewhat too high to compete with the path of **9** generation due to the artificial overestimation of unfavorable DBSI association entropy, as seen in comparison with the energy difference between **int1** and **int2**. Nevertheless, according to the crossover reaction, the proposed trimolecular pathway with higher barrier to form the side product **3** is believed to take place in the reactions.

### Kinetic studies

**Fig. 6:**
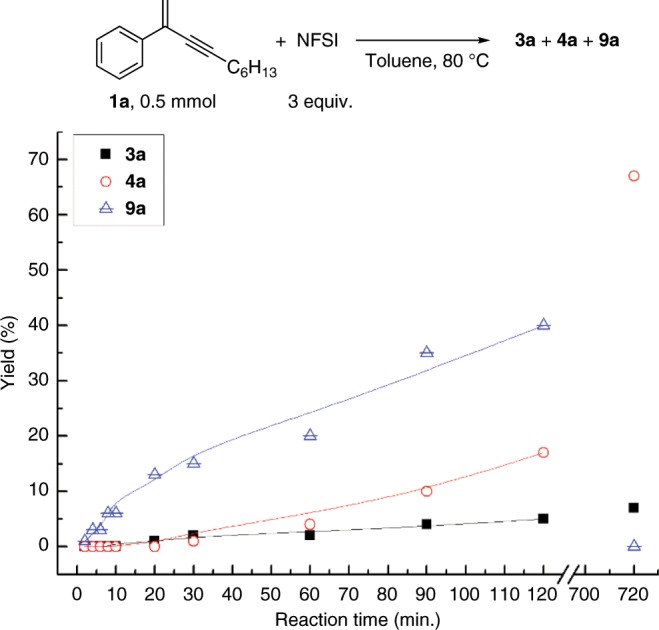
Kinetic studies. Kinetic profiles for the formation of **3a**, **4a** and active intermediate **9a**.

For further supporting this hypothesis, kinetic studies of the reaction was therefore conducted to track the formations of side product **3**, major product **4** and active intermediate **9** (Supplementary Fig. 5). Because of lacking **9** and DBSI, the induction period of formation of **3** and **4** should thus be seen if the trimolecular pathway is the favorable one. Figure 6 presents the kinetic profiles for the formations of **3a**, **4a** and **9a**.

As expected, the reaction rate of **9a** is faster than that of **3a**, and the induction period of **3a** and **4a** can be observed. Initially, even no trace amount of **3a** and **4a** can be detected in the first 6 minutes. After that, trace amounts of **3a** can be observed, but the rate of generation is very slow; nevertheless, the yield of **9a** has been over 10% at the 20 minute. Although the yields of **3a** and **4a** are almost the same in the first one hour, the generation rate of **4a** seems to accelerate probably due to the increase of concentration of **9a**. Finally, the active intermediate **9a** is consumed to deliver the major product **4a** at the end of reaction, and the amounts of **3a** should stop to grow after expending all of the reactant **1a**. The fast generation rate of **9** and the induction periods of formations of **3** and **4** are well consistent with the proposed reaction mechanism.

### Proposed mechanism

**Fig. 7:**
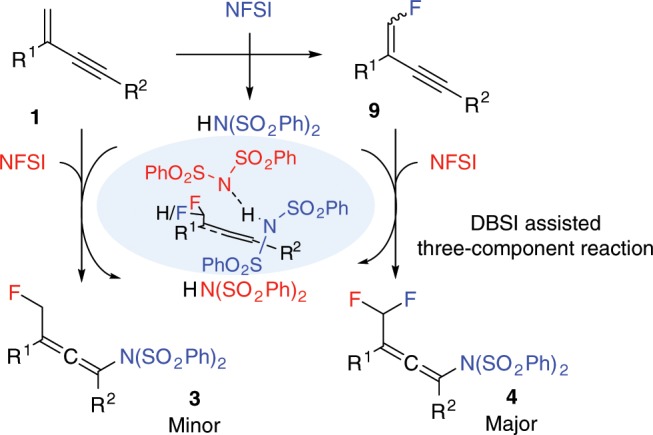
Proposed reaction mechanism. Dibenzenesulfonimide assisted three-component trifunctionalization of 1,3-enynes.

Based on our mechanistic experiments, theoretical studies and published work^41,44,45,69,70^, the proposed mechanism for the synthesis of difluoromethylated allenes from trifunctionalization of 1,3-enynes is depicted in Fig. 7. Initially, electrophilic fluorination of the 1,3-enyne **1** by NFSI synchronously generates the dibenzenesulfonimide and the fluorinated enyne **9** as the reactive intermediate. The second NFSI then reacts with **9** to afford the major product **4** assisted by the DBSI, adjacent to **9**. On the other hand, accumulation of DBSI will aid the formation of fluorination product **3**, which is observed as a side product cannot be converted into product **4** under the standard reaction conditions. The surplus DBSI can also assists the NFSI to react with the substrate **1** to form the side product **3**.

In conclusion, a metal-free synthesis of various difluoromethylated allenes through difluoroamination of 1,3-enynes has been developed. NFSI was used as the reactant and a broad substrate scope was obtained. The synthetic potential of difluoromethylated allenes has been demonstrated by transformations of them to a variety of useful difluoromethylated compounds. Moreover, this reaction is also a rare example of trifunctionalization of 1,3-enynes. Preliminary mechanistic studies suggest that a two-step pathway is involved and DFT studies revealed a dibenzenesulfonimide-assisted fluorination/fluoroamination with NFSI.

## Methods

### General procedure

In a flame-dried Schlenk tube, NFSI (1.5 mmol, 3.0 equiv.) was dissolved in toluene (1 mL) under a nitrogen atmosphere. Then, 1,3-enyne (0.5 mmol, 1.0 equiv.) was added. The reaction mixture was stirred at 80 ^o^C for 12 h. After the reaction completion as detected by TLC, the solvent was evaporated under reduced pressure. The residue was purified by flash column chromatography on silica gel (PE/EA or PE/DCM) to afford the allene product.

## Supplementary information

Supplementary Information

## Data Availability

Detailed experimental procedures and characterization of compounds can be found in the Supplementary Information. The X-ray crystallographic coordinates for structures reported in this article have been deposited at the Cambridge Crystallographic Data Center (**4k**: CCDC 1891507; **4l**: CCDC 1891508; **5**: CCDC 1891506). These data could be obtained free of charge from The Cambridge Crystallographic Data Center via www.ccdc.cam.ac.uk/data_request/cif. All data are available from the authors upon request.

## Source data

Source Data
