# Supplementary material for: Synthesis of difluoromethylated allenes through trifunctionalization of 1,3-enynes
Source: Nat Commun. 2020 Jan 21;11:416. doi: 10.1038/s41467-019-14254-3 (PMC6972827; doi:10.1038/s41467-019-14254-3)
Supplement: Supplementary file 1 — Supplementary Information [file 41467_2019_14254_MOESM1_ESM.pdf]

Supplementary Information

Synthesis of Difluoromethylated Allenes through Trifunctionalization of 1,3-Enynes

Muhammad et al.

Content

Supplementary Methods 3

Synthesis of 1,3-Enynes 4

Characterization Data for 1,3-Enynes 5

General Procedure for Amino-Difluorination of 1,3-Enynes 12

Characterization Data for the Difluoromethylated Allenes 13

Transformations of Amino-Difluorination Products 26

Mechanism Studies 30

Single Crystal Data 34

Computational Method and Details 38

NMR Spectra 46

Supplementary References 136

Supplementary Methods

General Information. All reactions were carried out under an atmosphere of nitrogen in glassware with magnetic stirring unless otherwise indicated. Commercially obtained reagents were used as received. Solvents were dried by Inert PureSolv MD5. Liquids and solutions were transferred via syringe. All reactions were monitored by thin-layer chromatography. ^{1}H, ^{19}F, and ^{13}C NMR spectra were recorded on Bruker-BioSpin AVANCE III HD or JEOL ECZ600S. Data for ^{1}H NMR spectra are reported relative to $CDCl_3$ as an internal standard (7.26 ppm) and are reported as follows: chemical shift (ppm), multiplicity, coupling constant (Hz), and integration. Data for ^{13}C NMR spectra are reported relative to $CDCl_3$ as an internal standard (77.0 ppm) and are reported in terms of chemical shift (ppm). GC-MS data were recorded on Thermo ISQ QD. HRMS data were recorded on Bruker Impact II UHR-TOF.

Synthesis of 1,3-Enynes

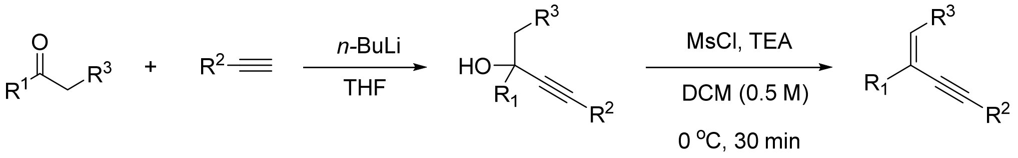

S1 **S2** **S3** **1,3-enyne**

To a 50 mL round bottomed flask was charged with compound **S2** (5 mmol, 1 equiv) and 10 mL of THF. The solution was cooled to -78 °C and *n*-BuLi (2.5 M in THF, 2 mL, 5 mmol, 1 equiv) was added. The resulting solution was stirred for 20 minutes at room temperature and then cooled to -78 °C again. Ketone **S1 (**5 mmol, 1 equiv) was added dropwise. The reaction mixture was then allowed to warm to room temperature and was monitored by TLC for completion. On completion the reaction was quenched with saturated aqueous NH_4Cl (40 mL). The aqueous layer was extracted with ethyl acetate and the combined organic layers were washed with brine (30 mL), dried over $MgSO_4$ and filtered. Then concentrated under reduced pressure to afford the crude material **S3**.[1]

The resulting crude propargyl alcohol **S3** was dissolved in dry DCM (5 mL), and the mixture was cooled to 0 °C with a cooling bath. To this solution was added TEA (25 mmol, 5 equiv) and methylsulfonyl chloride (12.5 mmol, 2.5 equiv) sequentially. After 30 min the reaction was monitored by TLC for completion. Once completion the reaction was quenched with saturated aqueous NH_4Cl (40 mL). The aqueous layer was extracted with ethyl acetate and the combined organic layers were washed with brine (30 mL), dried over $MgSO_4$, filtered, and concentrated under reduced pressure. The crude material was purified by flash chromatography to yield the 1,3-enyne.[2]

Characterization Data for 1,3-Enynes

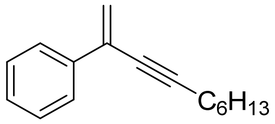

1a

^{1}H NMR (400 MHz, CDCl$_3$) δ 7.77 – 7.58 (m, 2H), 7.40 – 7.22 (m, 3H), 5.83 (d, *J* = 1.2 Hz, 1H), 5.57 (d, *J* = 1.2 Hz, 1H), 2.41 (t, *J* = 7.1 Hz, 2H), 1.66 – 1.57 (m, 2H), 1.50 – 1.41 (m, 2H), 1.35 – 1.28 (m, 4H), 0.93 – 0.88 (m, 3H). ^{13}C NMR (100 MHz, CDCl$_3$) δ 137.81, 130.98, 128.24, 128.08, 126.06, 119.31, 92.10, 79.75, 31.36, 28.72, 28.65, 22.59, 19.42, 14.06. These data matches with reported values.[3]

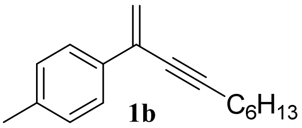

1b

^{1}H NMR (400 MHz, CDCl$_3$) δ 7.55 (d, *J* = 8.2 Hz, 2H), 7.14 (d, *J* = 7.9 Hz, 2H), 5.79 (s, 1H), 5.52 (s, 1H), 2.40 (t, *J* = 7.1 Hz, 2H), 2.35 (s, 3H), 1.66 – 1.54 (m, 2H), 1.51 – 1.39 (m, 2H), 1.37 – 1.27 (m, 4H), 0.94 – 0.86 (m, 3H). ^{13}C NMR (100 MHz, CDCl$_3$) δ 137.98, 135.03, 130.77, 128.96, 125.97, 118.47, 91.88, 79.88, 31.39, 28.74, 28.68, 22.61, 21.18, 19.44, 14.10.

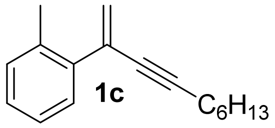

1c

^{1}H NMR (600 MHz, CDCl$_3$) δ 7.30 – 7.23 (m, 1H), 7.22 – 7.16 (m, 3H), 5.69 (d, *J* = 2.0 Hz, 1H), 5.39 (d, *J* = 2.0 Hz, 1H), 2.45 (s, 3H), 2.34 (t, *J* = 7.2 Hz, 2H), 1.58 – 1.53 (m, 2H), 1.43 – 1.38 (m, 2H), 1.33 – 1.28 (m, 4H), 0.90 (t, *J* = 7.2 Hz, 3H). ^{13}C NMR (150 MHz, CDCl$_3$) δ 140.10, 135.52, 132.36, 130.39, 128.76, 127.75, 125.88, 123.91, 92.07, 80.71, 31.44, 28.74, 22.65, 20.29, 19.56, 14.13.

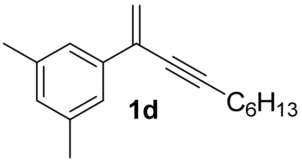

1d

^{1}H NMR (400 MHz, CDCl$_3$) δ 7.26 (s, 2H), 6.93 (s, 1H), 5.80 (s, 1H), 5.53 (s, 1H), 2.41 (t, *J* = 7.0 Hz, 2H), 2.32 (s, 6H), 1.66 – 1.54 (m, 2H), 1.52 – 1.40 (m, 2H), 1.37 – 1.27 (m, 4H), 0.90 (t, *J* = 7.0 Hz, 3H). ^{13}C NMR (100 MHz, CDCl$_3$) δ 137.81, 137.75, 131.12, 129.82, 123.99, 119.16, 91.82, 80.04, 31.45, 28.75, 28.71, 22.67, 21.38, 19.48, 14.13.

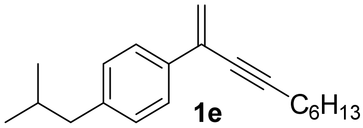

1e

^{1}H NMR (600 MHz, $CDCl_3$) δ 7.56 (d, *J* = 8.3 Hz, 2H), 7.11 (d, *J* = 8.4 Hz, 2H), 5.80 (d, *J* = 1.3 Hz, 1H), 5.52 (d, *J* = 1.1 Hz, 1H), 2.47 (d, *J* = 7.2 Hz, 2H), 2.40 (t, *J* = 7.1 Hz, 2H), 1.92 – 1.80 (m, 1H), 1.66 – 1.54 (m, 2H), 1.51 – 1.40 (m, 2H), 1.39 – 1.27 (m, 4H), 0.94 – 0.86 (m, 9H). ^{13}C NMR (150 MHz, $CDCl_3$) δ 141.90, 135.32, 130.91, 129.09, 125.88, 118.52, 91.93, 80.00, 45.19, 31.45, 30.29, 28.82, 28.74, 22.66, 22.45, 19.51, 14.14.

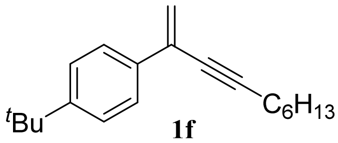

1f

^{1}H NMR (600 MHz, $CDCl_3$) δ 7.70 – 7.58 (m, 2H), 7.45 – 7.36 (m, 2H), 5.83 (s, 1H), 5.56 (s, 1H), 2.43 (t, *J* = 7.1 Hz, 2H), 1.68 – 1.59 (m, 2H), 1.53 – 1.43 (m, 2H), 1.37 – 1.33 (m, 13H), 0.98 – 0.89 (m, 3H). ^{13}C NMR (150 MHz, $CDCl_3$) δ 151.25, 135.16, 130.84, 125.88, 125.27, 118.64, 91.90, 80.00, 34.65, 31.47, 31.38, 28.85, 28.76, 22.68, 19.52, 14.16.

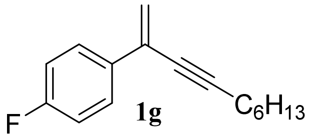

1g

^{1}H NMR (600 MHz, $CDCl_3$) δ 7.66 – 7.57 (m, 2H), 7.06 – 6.97 (m, 2H), 5.76 (s, 1H), 5.54 (s, 1H), 2.40 (t, *J* = 7.1 Hz, 2H), 1.64 – 1.54 (m, 2H), 1.48 – 1.41 (m, 2H), 1.35 – 1.29 (m, 4H), 0.91 – 0.88 (m, 3H). ^{13}C NMR (150 MHz, $CDCl_3$) δ 162.81 (d, *J* = 247.5 Hz), 134.01 (d, *J* = 3.0 Hz), 130.01, 127.86 (d, *J* = 8.1 Hz), 119.10, 115.16 (d, *J* = 21.5 Hz), 92.42, 79.67, 31.42, 28.76, 28.73, 22.66, 19.47, 14.13.

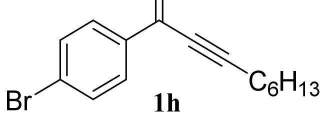

1h

^{1}H NMR (600 MHz, $CDCl_3$) δ 7.55 – 7.50 (m, 2H), 7.48 – 7.43 (m, 2H), 5.82 (s, 1H), 5.59 (s, 1H), 2.41 (t, *J* = 7.2 Hz, 2H), 1.64 – 1.56 (m, 2H), 1.48 – 1.41 (m, 2H), 1.36 – 1.30 (m, 4H), 0.91 (t, *J* = 6.9 Hz, 3H). ^{13}C NMR (150 MHz, $CDCl_3$) δ 136.85, 131.44, 130.11, 127.80, 122.28, 119.80, 92.68, 79.39, 31.46, 28.77, 22.70, 19.50, 14.18.

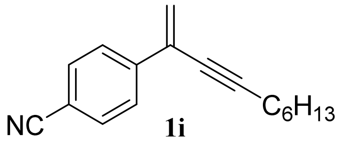

1i

^{1}H NMR (400 MHz, $CDCl_3$) δ 7.74 (d, *J* = 8.4 Hz, 2H), 7.63 (d, *J* = 8.3 Hz, 2H), 5.93 (s, 1H), 5.72 (s, 1H), 2.41 (t, *J* = 7.1 Hz, 2H), 1.65 – 1.58 (m, 2H), 1.49 – 1.42 (m, 2H), 1.34 – 1.28 (m, 4H), 0.9 (t, *J* = 6.9 Hz, 3H). ^{13}C NMR (100 MHz, $CDCl_3$) δ 142.15, 132.11, 126.69, 122.09, 118.82, 111.55, 93.44, 78.61, 31.31, 28.64, 28.57, 22.56, 19.37, 14.05.

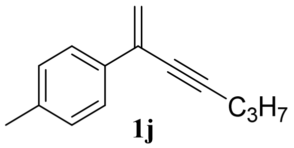

1j

^{1}H NMR (400 MHz, $CDCl_3$) δ 7.55 (d, *J* = 8.1 Hz, 2H), 7.14 (d, *J* = 7.9 Hz, 2H), 5.80 (s, 1H), 5.53 (s, 1H), 2.39 (t, *J* = 7.0 Hz, 2H), 2.34 (s, 3H), 1.63 (h, *J* = 7.3 Hz, 2H), 1.04 (t, *J* = 7.4 Hz, 3H). ^{13}C NMR (100 MHz, $CDCl_3$) δ 137.98, 135.03, 130.77, 128.97, 125.97, 118.52, 91.66, 80.04, 22.26, 21.43, 21.19, 13.67.

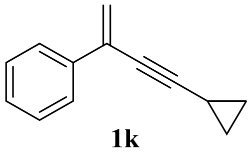

1k

^{1}H NMR (400 MHz, $CDCl_3$) δ 7.70 – 7.55 (m, 2H), 7.39 – 7.25 (m, 3H), 5.81 (d, *J* = 1.1 Hz, 1H), 5.55 (d, *J* = 1.2 Hz, 1H), 1.53 – 1.33 (m, 1H), 0.87 – 0.77 (m, 4H). ^{13}C NMR (100 MHz, $CDCl_3$) δ 137.89, 130.96, 128.36, 128.21, 126.14, 119.55, 95.20, 75.10, 8.77, 0.30. These data matches with reported values.[4]

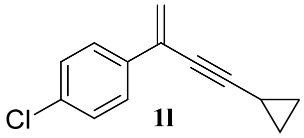

1l

^{1}H NMR (600 MHz, $CDCl_3$) δ 7.56 – 7.53 (m, 2H), 7.31 – 7.28 (m, 2H), 5.79 (s, 1H), 5.57 (s, 1H), 1.48 – 1.42 (m, 1H), 0.89 – 0.85 (m, 2H), 0.82 – 0.78 (m, 2H). ^{13}C NMR (150 MHz, $CDCl_3$) δ 136.34, 134.03, 129.87, 128.46, 127.45, 119.85, 95.62, 74.65, 8.80, 0.26.

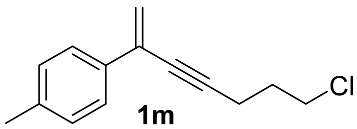

1m

^{1}H NMR (400 MHz, $CDCl_3$) δ 7.49 (d, *J* = 8.0 Hz, 2H), 7.15 (d, *J* = 7.9 Hz, 2H), 5.82 (s, 1H), 5.54 (s, 1H), 3.70 (t, *J* = 6.4 Hz, 2H), 2.61 (t, *J* = 6.8 Hz, 2H), 2.35 (s, 3H), 2.05

(p, J = 6.6 Hz, 2H). ^{13}C NMR (100 MHz, $CDCl_3$) δ 138.15, 134.78, 130.47, 129.05, 125.91, 119.14, 89.42, 80.90, 43.81, 31.43, 21.20, 16.88.

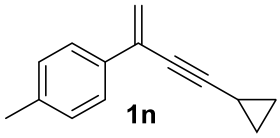

1n

^{1}H NMR (600 MHz, $CDCl_3$) δ 7.57 – 7.53 (m, 2H), 7.31 – 7.28 (m, 2H), 5.80 (s, 1H), 5.57 (s, 1H), 1.47 – 1.41 (m, 1H), 0.90 – 0.86 (m, 2H), 0.83 – 0.78 (m, 2H). ^{13}C NMR (150 MHz, $CDCl_3$) δ136.32, 129.82, 128.98, 128.46, 127.44, 119.87, 95.62, 74.61, 26.66, 8.79, 0.23.

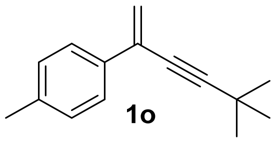

1o

^{1}H NMR (400 MHz, $CDCl_3$) δ 7.52 (d, J = 8.4 Hz, 2H), 7.14 (d, J = 7.9 Hz, 2H), 5.78 (s, 1H), 5.51 (s, 1H), 2.35 (s, 3H), 1.32 (s, 9H). ^{13}C NMR (100 MHz, $CDCl_3$) δ 137.95, 135.14, 130.65, 128.97, 125.96, 118.30, 99.92, 78.38, 31.05, 28.05, 21.20.

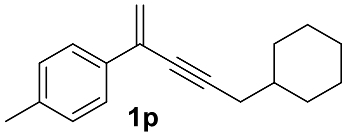

1p

^{1}H NMR (400 MHz, $CDCl_3$) δ 7.55 (d, J = 8.4 Hz, 2H), 7.14 (d, J = 7.9 Hz, 2H), 5.79 (s, 1H), 5.52 (s, 1H), 2.35 (s, 3H), 2.30 (d, J = 6.6 Hz, 2H), 1.89 – 1.83 (m, 2H), 1.75 – 1.70 (m, 2H), 1.56 (m, 1H), 1.28 – 1.00 (m, 6H). ^{13}C NMR (100 MHz, $CDCl_3$) δ 137.97, 135.09, 130.83, 128.98, 125.99, 118.47, 90.75, 80.76, 37.54, 32.85, 27.29, 26.33, 26.21, 21.19.

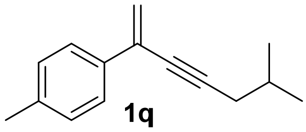

1q

^{1}H NMR (600 MHz, $CDCl_3$) δ 7.64 – 7.55 (m, 2H), 7.22 – 7.14 (m, 2H), 5.84 (s, 1H), 5.57 (s, 1H), 2.38 (s, 3H), 2.34 (d, J = 7.6 Hz, 2H), 2.00 – 1.91 (m, 1H), 1.08 (d, J = 6.7 Hz, 6H). ^{13}C NMR (150 MHz, $CDCl_3$) δ 138.16, 135.29, 131.04, 129.18, 126.18, 118.70, 90.93, 81.01, 28.83, 28.48, 22.34, 21.38.

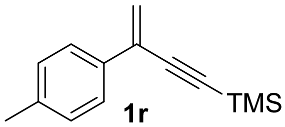

1r

^{1}H NMR (600 MHz, $CDCl_3$) δ 7.51 (d, J = 6.7 Hz,2H), 7.15 (d, J = 6.7 Hz, 2H), 5.88 (s, 1H), 5.65 (s, 1H), 2.35 (s, 3H), 0.24 (s, 9H). ^{13}C NMR (150 MHz, $CDCl_3$) δ 138.31, 134.23, 130.48, 129.13, 126.02, 126.01, 104.37, 95.72, 21.28, 0.05.

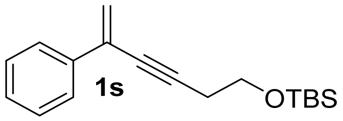

1s

^{1}H NMR (400 MHz, $CDCl_3$) δ 7.64 (d, *J* = 6.8 Hz, 2H), 7.40 – 7.29 (m, 3H), 5.89 (s, 1H), 5.63 (s, 1H), 3.85 (t, *J* = 7.0 Hz, 2H), 2.67 (t, *J* = 7.0 Hz, 2H), 0.95 (s, 9H), 0.13 (s, 6H). ^{13}C NMR (100 MHz, $CDCl_3$) δ 137.58, 130.81, 128.30, 128.19, 126.11, 119.77, 88.85, 80.76, 61.93, 25.95, 23.89, 18.39, -5.22.

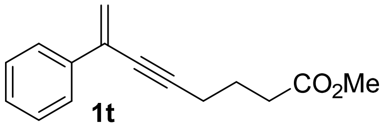

1t

^{1}H NMR (600 MHz, $CDCl_3$) δ 7.66 – 7.61 (m, 2H), 7.35 – 7.32 (m, 2H), 7.30 – 7.27 (m, 1H), 5.85 (d, *J* = 1.1 Hz, 1H), 5.59 (d, *J* = 1.1 Hz, 1H), 3.67 (s, 3H), 2.51 – 2.47 (m, 4H), 1.95 – 1.90 (m, 2H). ^{13}C NMR (150 MHz, $CDCl_3$) δ 173.69, 137.68, 130.83, 128.41, 128.28, 126.11, 119.93, 90.51, 80.71, 51.70, 33.01, 24.00, 18.96.

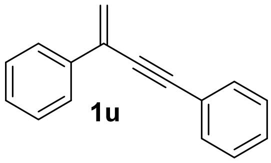

1u

^{1}H NMR (400 MHz, $CDCl_3$) δ 7.76 – 7.69 (m, 2H), 7.58 – 7.50 (m, 2H), 7.41 – 7.31 (m, 6H), 5.98 (s, 1H), 5.76 (s, 1H). ^{13}C NMR (100 MHz, $CDCl_3$) δ 137.28, 131.68, 130.62, 128.42, 128.36, 126.11, 123.11, 120.68, 90.78, 88.56. These data matches with reported values.[5]

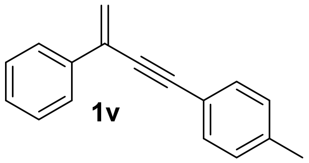

1v

^{1}H NMR (600 MHz, $CDCl_3$) δ 7.73 (d, *J* = 8.5 Hz, 2H), 7.43 (d, *J* = 8.0 Hz, 2H), 7.38 (t, *J* = 7.4 Hz, 2H), 7.34 – 7.30 (m, 1H), 7.15 (d, *J* = 7.8 Hz, 2H), 5.96 (s, 1H), 5.74 (s, 1H), 2.37 (s, 3H). ^{13}C NMR (150 MHz, $CDCl_3$) δ 138.66, 137.47, 131.66, 130.82, 129.22, 128.48, 128.38, 126.21, 120.41, 120.11, 91.08, 88.01, 21.61. These data matches with reported values.[6]

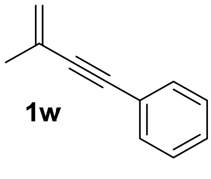

1w

^{1}H NMR (400 MHz, $CDCl_3$) δ 7.50 – 7.38 (m, 2H), 7.28 – 7.20 (m, 3H), 5.39 (s, 1H), 5.26 (s, 1H), 1.96 (s, 3H). ^{13}C NMR (100 MHz, $CDCl_3$) δ 131.68, 128.40, 128.25, 126.97, 123.41, 122.06, 90.72, 88.61, 23.60.

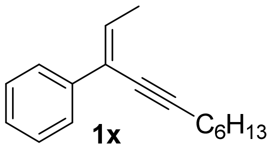

1x

^{1}H NMR (400 MHz, $CDCl_3$) δ 7.60 – 7.54 (m, 2H), 7.34 – 7.28 (m, 2H), 7.25 – 7.20 (m, 1H), 6.39 (q, *J* = 7.0 Hz, 1H), 2.46 (t, *J* = 7.0 Hz, 2H), 2.04 (d, *J* = 7.0 Hz, 3H), 1.67 – 1.59 (m, 2H), 1.52 – 1.44 (m, 2H), 1.36 – 1.30 (m, 4H), 0.94 – 0.85 (m, 3H). ^{13}C NMR (100 MHz, $CDCl_3$) δ 138.92, 131.50, 128.19, 127.12, 125.86, 124.75, 96.84, 77.70, 31.38, 28.95, 28.64, 22.61, 19.63, 16.78, 14.07. These data matches with reported values.[3]

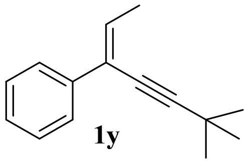

1y

^{1}H NMR (400 MHz, $CDCl_3$) δ 7.59 – 7.55 (m, 2H), 7.34 – 7.28 (m, 2H), 7.26 – 7.20 (m, 1H), 6.39 (q, *J* = 6.9 Hz, 1H), 2.03 (d, *J* = 6.9 Hz, 3H), 1.34 (s, 9H). ^{13}C NMR (150 MHz, $CDCl_3$) δ 138.93, 131.39, 128.28, 127.19, 125.87, 124.65, 105.25, 76.14, 31.31, 28.41, 16.85.

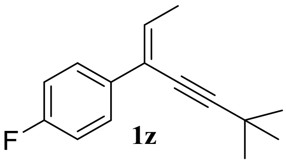

1z

^{1}H NMR (400 MHz, $CDCl_3$) δ 7.61 – 7.43 (m, 2H), 7.08 – 6.88 (m, 2H), 6.29 (q, *J* = 6.9 Hz, 1H), 2.01 (d, *J* = 6.9 Hz, 3H), 1.34 (s, 9H). ^{13}C NMR (100 MHz, $CDCl_3$) δ 162.19 (d, *J* = 246.0 Hz), 134.96 (d, *J* = 3.2 Hz), 131.03 (d, *J* = 1.8 Hz), 127.35 (d, *J* = 7.9 Hz), 123.62, 115.01 (d, *J* = 21.5 Hz), 105.39, 31.22, 28.36, 16.76.

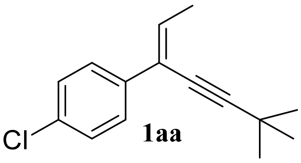

1aa

^{1}H NMR (400 MHz, $CDCl_3$) δ 7.48 (d, *J* = 8.6 Hz, 2H), 7.26 (d, *J* = 8.6 Hz, 2H), 6.35 (q, *J* = 6.9 Hz, 1H), 2.01 (d, *J* = 7.0 Hz, 3H), 1.33 (s, 9H). ^{13}C NMR (150 MHz, $CDCl_3$) δ 137.41, 132.93, 131.78, 128.40, 127.15, 123.71, 105.64, 75.81, 31.29, 28.42, 16.88.

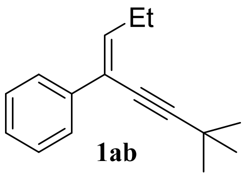

1ab

^{1}H NMR (400 MHz, $CDCl_3$) δ 7.64 – 7.53 (m, 2H), 7.35 – 7.26 (m, 2H), 7.25 – 7.17 (m, 1H), 6.30 (qd, J = 6.5, 2.7 Hz, 1H), 2.53 – 2.39 (m, 2H), 1.33 (s, 9H), 1.14 – 1.05 (m, 3H). ^{13}C NMR (100 MHz, $CDCl_3$) δ 138.82, 138.51, 128.24, 127.19, 125.89, 123.12, 104.67, 76.11, 31.24, 28.35, 24.48, 13.51.

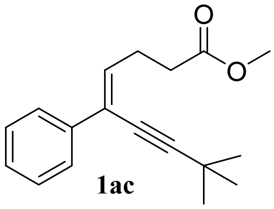

1ac

^{1}H NMR (400 MHz, $CDCl_3$) δ 7.57 (d, J = 7.2 Hz, 2H), 7.32 (d, J = 7.4 Hz, 2H), 7.24 (t, J = 7.4 Hz, 1H), 6.33 (t, J = 7.4 Hz, 1H), 3.69 (s, 3H), 2.76 (q, J = 7.5 Hz, 2H), 2.52 (t, J = 7.5 Hz, 2H), 1.34 (s, 9H). ^{13}C NMR (100 MHz, $CDCl_3$) δ 173.54, 138.34, 133.87, 128.27, 127.51, 125.95, 125.14, 105.60, 75.73, 51.68, 33.32, 31.13, 28.34, 26.39.

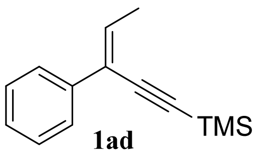

1ad

^{1}H NMR (600 MHz, $CDCl_3$) δ 7.68 – 7.65 (m, 2H), 7.40 (m, 2H), 7.34 – 7.30 (m, 1H), 6.57 (q, J = 7.0 Hz, 1H), 2.17 (d, J = 7.0 Hz, 3H), 0.37 (s, 9H). ^{13}C NMR (150 MHz, $CDCl_3$) δ 138.10, 134.39, 128.50, 127.56, 126.03, 124.76, 102.42, 100.89, 17.26, 0.32. These data matches with reported values.[4]

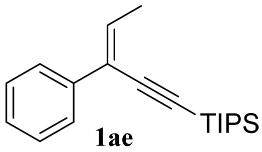

1ae

^{1}H NMR (600 MHz, $CDCl_3$) δ 7.72 – 7.62 (m, 2H), 7.42 – 7.35 (m, 2H), 7.30 (t, J = 7.0 Hz, 1H), 6.61 – 6.52 (m, 1H), 2.17 (m, 3H), 1.23 – 1.20 (m, 18H), 1.16 – 1.15 (m, 3H). ^{13}C NMR (150 MHz, $CDCl_3$) δ 138.18, 133.91, 128.42, 127.43, 125.94, 125.04, 104.07, 97.25, 18.88, 18.60, 11.52. These data matches with reported values.[7]

General Procedure for Amino-Difluorination of 1,3-Enynes

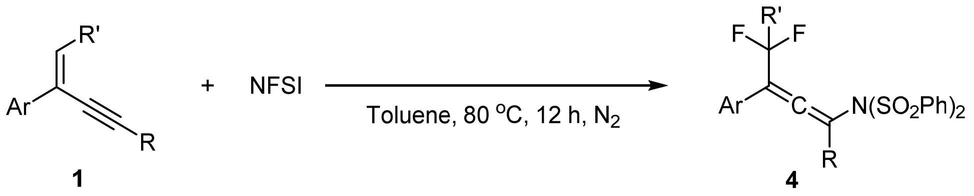

General Procedure: In a flame-dried Schlenk tube, NFSI (1.5 mmol, 3.0 equiv) were dissolved in toluene (1 mL) under a nitrogen atmosphere. Then, 1,3-enyne (0.5 mmol, 1.0 equiv) was added. The reaction mixture was stirred at 80 °C for 12 h. After the reaction completion as detected by TLC, the solvent was evaporated under reduced pressure. The residue was purified by flash column chromatography on silica gel (PE/EA or PE/DCM) to afford the allene product.

Characterization Data for the Difluoromethylated Allenes

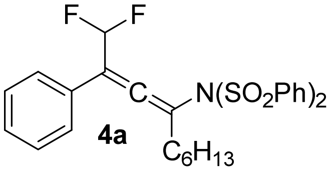

4a

Following the general procedure, compound **4a** was obtained as a pale yellow oil (177 mg, 65% yield). ^{1}H NMR (400 MHz, $CDCl_3$) δ 7.95 (d, *J* = 7.5 Hz, 4H), 7.60 – 7.50 (m, 2H), 7.45 – 7.36 (m, 5H), 7.42 – 7.32 (m, 4H), 6.26 (t, *J* = 55.0 Hz, 1H), 2.56 – 2.46 (m, 1H), 2.38 – 2.28 (m, 1H), 1.46 – 1.38 (m, 2H), 1.34 – 1.21 (m, 6H), 0.85 (t, *J* = 6.9 Hz, 3H). ^{13}C NMR (100 MHz, $CDCl_3$) δ 208.54 (t, *J* = 9.0 Hz), 139.39, 134.19, 129.19, 129.15, 128.93, 128.89, 128.47, 128.16, 114.06 (t, *J* = 245.4 Hz), 113.98, 109.64 (t, *J* = 24.8 Hz), 34.32, 31.69, 28.74, 26.59, 22.68, 14.17. ^{19}F NMR (376 MHz, $CDCl_3$) δ -110.26 – -112.17 (dm, $J_{H\text{-}F}$ = 55.0 Hz, 2F). HRMS (ESI) calcd for $[C_{28}H_{29}F_2NO_4S_2Na]^+$ ($[M+Na]^+$): 568.1398, found: 568.1397.

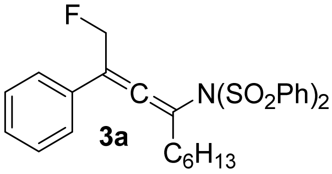

3a

Following the general procedure, compound **3a** was obtained as the side product. ^{1}H NMR (400 MHz, $CDCl_3$) δ 7.96 (d, *J* = 7.9 Hz, 4H), 7.63 – 7.45 (m, 3H), 7.43 – 7.29 (m, 8H), 5.18 – 5.07 (m, 1H), 5.07 – 4.94 (m, 1H), 2.57 – 2.44 (m, 1H), 2.34 – 2.21 (m, 1H), 1.50 – 1.36 (m, 2H), 1.36 – 1.27 (m, 2H), 1.27 – 1.18 (m, 4H), 0.84 (t, *J* = 6.8 Hz, 3H). ^{13}C NMR (100 MHz, $CDCl_3$) δ 207.89 (d, *J* = 9.5 Hz), 139.51, 133.89, 131.69, 128.94, 128.80, 128.67, 128.35, 127.15, 111.04 (d, *J* = 2.0 Hz), 109.50 (d, *J* = 18.3 Hz), 80.74 (d, *J* = 174.9 Hz), 34.13, 31.62, 28.66, 26.56, 22.59, 14.08. ^{19}F NMR (376 MHz, $CDCl_3$) δ -211.78 (s, 1F, decoupled with H-nuclei). HRMS (ESI) calcd for $[C_{28}H_{30}FNO_4S_2Na]^+$ ($[M+Na]^+$): 550.1492, found: 550.1493.

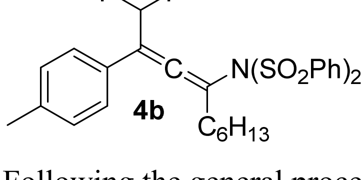

4b

Following the general procedure, compound **4b** was obtained as a pale yellow oil (173 mg, 62% yield). ^{1}H NMR (400 MHz, $CDCl_3$) δ 7.95 (d, *J* = 7.8 Hz, 4H), 7.62 – 7.53 (m, 2H), 7.51 – 7.34 (m, 4H), 7.30 (d, *J* = 8.0 Hz, 2H), 7.18 (d, *J* = 8.0 Hz, 2H), 6.22

(t, J = 55.1 Hz, 1H), 2.55 – 2.44 (m, 1H), 2.37 (s, 3H), 2.35 – 2.27 (m, 1H), 1.47 – 1.37 (m, 2H), 1.34 – 1.14 (m, 6H), 0.85 (t, J = 6.9 Hz, 3H). ^{13}C NMR (100 MHz, $CDCl_3$) δ 208.22 (t, J = 9.0 Hz), 139.45, 139.34, 134.19, 129.67, 129.18, 128.51, 128.07, 125.83, 114.11 (t, J = 245.4 Hz), 113.80, 109.65 (t, J = 24.8 Hz), 34.39, 31.74, 28.78, 26.60, 22.73, 21.51, 14.23. ^{19}F NMR (376 MHz, $CDCl_3$) δ -110.33 – -112.16 (dm, $J_{H\text{-}F}$ = 55.0 Hz, 2F). HRMS (ESI) calcd for $[C_{29}H_{31}F_2NO_4S_2Na]^+$ ($[M+Na]^+$): 582.1555, found: 582.1554.

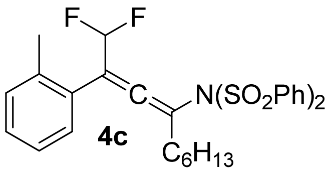

4c

Following the general procedure (the reaction was performed at 100 °C), compound **4c** was obtained as a pale yellow oil (173 mg, 62% yield). ^{1}H NMR (400 MHz, $CDCl_3$) δ 8.34 – 7.32 (m, 9H), 7.29 – 7.21 (m, 2H), 7.20 – 7.14 (m, 2H), 7.01 (d, J = 7.6 Hz, 1H), 6.21 (t, J = 56.0 Hz, 1H), 2.55 – 2.37 (m, 1H), 2.32 – 2.19 (m, 1H), 2.02 (s, 3H), 1.55 – 1.39 (m, 2H), 1.36 – 1.20 (m, 6H), 0.89 (t, J = 6.8 Hz, 3H). ^{13}C NMR (100 MHz, $CDCl_3$) δ 207.43 (t, J = 8.4 Hz), 137.12, 134.05, 130.55, 129.70, 128.97, 128.91, 128.54, 128.43, 113.40 (t, J = 245.3 Hz), 111.34, 107.41 (d, J = 26.0 Hz), 33.90, 31.61, 28.65, 26.92, 22.59, 20.39, 14.12. ^{19}F NMR (376 MHz, $CDCl_3$) δ -110.60 – -112.77 (dm, $J_{H\text{-}F}$ = 56.0 Hz, 2F). HRMS (ESI) calcd for $[C_{29}H_{31}F_2NO_4S_2Na]^+$ ($[M+Na]^+$): 582.1555, found: 582.1555.

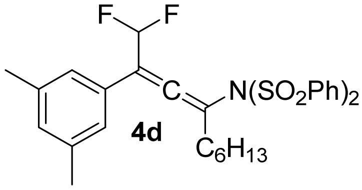

4d

Following the general procedure, compound **4d** was obtained as a pale yellow oil (149 mg, 52% yield). ^{1}H NMR (400 MHz, $CDCl_3$) δ 7.89-7.81 (m, 4H), 7.48-7.40 (m, 2H), 7.36-7.24 (m, 4H), 6.94 (s, 2H), 6.89 (s, 1H), 6.15 (t, J = 55.1 Hz, 1H), 2.50 – 2.36 (m, 1H), 2.29 – 2.22 (m, 7H), 1.41 – 1.28 (m, 2H), 1.26 – 1.11 (m, 6H), 0.76 (t, J = 6.8 Hz, 3H). ^{13}C NMR (100 MHz, $CDCl_3$) δ 208.38 (t, J = 8.9 Hz), 139.29, 138.31, 134.00, 130.78, 129.00, 128.50, 128.32, 125.76, 113.89 (t, J = 245.3 Hz), 113.52, 109.67 (t, J = 24.6 Hz), 34.18, 31.60, 28.62, 26.44, 22.59, 21.41, 14.09. ^{19}F NMR (376 MHz, $CDCl_3$) δ -110.19 – -112.01 (dm, $J_{H\text{-}F}$ = 55.1 Hz, 2F). HRMS (ESI) calcd for $[C_{30}H_{33}F_2NO_4S_2Na]^+$ ($[M+Na]^+$): 596.1711, found: 596.1711.

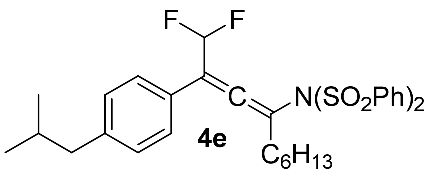
4e

Following the general procedure, compound **4e** was obtained as a pale yellow oil (159 mg, 53% yield). ^{1}H NMR (400 MHz, $CDCl_3$) δ 7.95 (d, *J* = 7.9 Hz, 4H), 7.58 – 7.30 (m, 8H), 7.14 (d, *J* = 8.0 Hz, 2H), 6.25 (t, *J* = 55.1 Hz, 1H), 2.58 – 2.47 (m, 3H), 2.39 – 2.27 (m, 1H), 1.95 – 1.82 (m, 1H), 1.48 – 1.38 (m, 2H), 1.33 – 1.19 (m, 6H), 0.92 (d, *J* = 6.6 Hz, 6H), 0.84 (t, *J* = 6.8 Hz, 3H). ^{13}C NMR (100 MHz, $CDCl_3$) δ 208.15 (t, *J* = 8.9 Hz), 143.00, 139.30, 134.05, 129.58, 129.03, 128.35, 127.76, 125.91, 114.02 (t, *J* = 245.4 Hz), 113.70, 109.55 (t, *J* = 24.8 Hz), 45.16, 34.26, 31.59, 30.24, 28.63, 26.45, 22.58, 22.42, 22.38, 14.08. ^{19}F NMR (376 MHz, $CDCl_3$) δ -100.32 – -112.16 (dm, $J_{H\text{-}F}$ = 55.1 Hz, 2F). HRMS (ESI) calcd for $[C_{32}H_{37}F_2NO_4S_2Na]^+$ ($[M+Na]^+$): 624.2024, found: 624.2022.

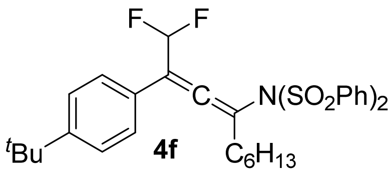
4f

Following the general procedure (the reaction was performed at 100 °C), compound **4f** was obtained as a pale yellow oil (171 mg, 62% yield). ^{1}H NMR (400 MHz, $CDCl_3$) δ 8.00 (d, *J* = 8.0 Hz, 4H), 7.67 – 7.53 (m, 3H), 7.48 – 7.37 (m, 7H), 6.28 (t, *J* = 55.1 Hz, 1H), 2.60 – 2.50 (m, 1H), 2.44 – 2.34 (m, 1H), 1.51 – 1.43 (m, 2H), 1.38 (s, 9H), 1.34 – 1.25 (m, 6H), 0.89 (t, *J* = 6.9 Hz, 3H). ^{13}C NMR (100 MHz, $CDCl_3$) δ 208.22 (t, *J* = 9.1 Hz), 152.30, 139.27, 134.02, 129.00, 128.34, 127.72, 125.76, 113.97 (t, *J* = 246.4 Hz), 113.64, 113.52, 109.42 (t, *J* = 25.2 Hz), 34.76, 34.22, 31.60, 31.24, 28.64, 26.46, 22.58, 14.08. ^{19}F NMR (376 MHz, $CDCl_3$) δ -110.31 – -112.11 (dm, $J_{H\text{-}F}$ = 55.0 Hz, 2F). HRMS (ESI) calcd for $[C_{32}H_{37}F_2NO_4S_2Na]^+$ ($[M+Na]^+$): 624.2024, found: 624.2024.

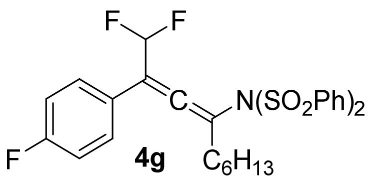
4g

Following the general procedure, compound **4g** was obtained as a pale yellow oil (141 mg, 50% yield). ^{1}H NMR (400 MHz, $CDCl_3$) δ 7.96 (d, *J* = 7.9 Hz, 4H), 7.58 (t, *J* = 7.5 Hz, 2H), 7.51 – 7.33 (m, 6H), 7.05 (t, *J* = 8.6 Hz, 2H), 6.21 (t, *J* = 54.9 Hz, 1H), 2.56 – 2.42 (m, 1H), 2.41 – 2.28 (m, 1H), 1.46 – 1.34 (m, 2H), 1.33 – 1.17 (m, 6H), 0.85 (t, *J* = 6.8 Hz, 3H). ^{13}C NMR (100 MHz, $CDCl_3$) δ 208.32 (t, *J* = 8.7 Hz), 163.05

(d, J = 250.0 Hz), 139.20, 134.16, 129.95 (d, J = 8.4 Hz), 129.08, 128.32, 124.61 (d, J = 3.5 Hz), 115.87 (d, J = 21.9 Hz), 113.95 , 113.92 (t, J = 245.7 Hz), 108.53 (t, J = 25.3 Hz), 34.22, 31.69, 28.71, 26.56, 22.68, 14.18. ^{19}F NMR (376 MHz, $CDCl_3$, decoupled with H-nuclei) δ-110.42 – -112.08 (m, 2F), 111.59 (s, 1F). HRMS (ESI) calcd for $[C_{28}H_{28}F_3NO_4S_2Na]^+$ ($[M+Na]^+$): 586.1304, found: 586.1303.

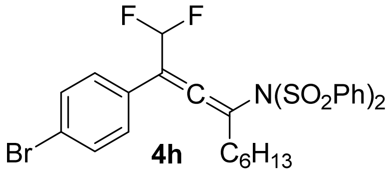

4h

Following the general procedure, compound **4h** was obtained as a brown liquid (138 mg, 44% yield). ^{1}H NMR (400 MHz, $CDCl_3$) δ 7.96 (d, J = 7.9 Hz, 4H), 7.59 (t, J = 7.5 Hz, 2H), 7.51 – 7.37 (m, 6H), 7.27 (d, J = 8.4 Hz, 2H), 6.20 (t, J = 54.9 Hz, 1H), 2.55 – 2.43 (m, 1H), 2.40 – 2.28 (m, 1H), 1.44 – 1.35 (m, 2H), 1.34 – 1.15 (m, 6H), 0.85 (t, J = 6.8 Hz, 3H). ^{13}C NMR (100 MHz, $CDCl_3$) δ 208.55 (t, J = 9.1 Hz), 139.16, 134.18, 131.97, 129.55, 129.10, 128.32, 127.57, 123.36, 114.23, 113.76 (t, J = 245.6 Hz), 108.58 (t, J = 25.4 Hz), 34.03, 31.54, 28.57, 26.40, 22.54, 14.05. ^{19}F NMR (376 MHz, $CDCl_3$) δ -110.56 – -112.40 (dm, $J_{H\text{-}F}$ = 54.8 Hz, 2F). HRMS (ESI) calcd for $[C_{28}H_{28}BrF_2NO_4S_2Na]^+$ ($[M+Na]^+$): 646.0503, found: 646.0504.

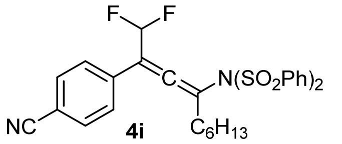

4i

Following the general procedure, compound **4i** was obtained as a yellow oil (57.0 mg, 20% yield). ^{1}H NMR (400 MHz, $CDCl_3$) δ 8.06 – 7.91 (m, 4H), 7.70 – 7.58 (m, 4H), 7.57 – 7.43 (m, 6H), 6.21 (t, J = 54.7 Hz, 1H), 2.55 – 2.41 (m, 1H), 2.40 – 2.31 (m, 1H), 1.43 – 1.34 (m, 2H), 1.33 – 1.23 (m, 4H), 1.22 – 1.16 (m, 2H), 0.84 (t, J = 6.8 Hz, 3H). ^{13}C NMR (100 MHz, CDCl3) δ 209.63 (t, J = 8.9 Hz), 139.11, 134.28, 133.43, 132.40, 129.15, 128.56, 128.32, 118.35, 113.58 (t, J = 246.2 Hz), 115.08, 112.49, 108.11 (t, J = 25.2 Hz), 33.92, 31.48, 28.52, 26.34, 22.50, 13.99. ^{19}F NMR (376 MHz, CDCl3) δ - 111.07 (d, J = 54.9 Hz, 2F). HRMS (ESI) calcd for $[C_{29}H_{28}F_2N_2O_4S_2Na]^+$ ($[M+Na]^+$): 593.1351, found: 593.1350.

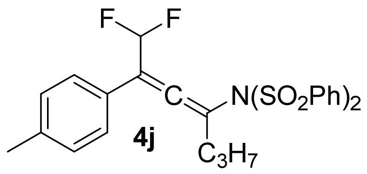

4j

Following the general procedure, compound **4j** was obtained as a pale yellow oil (132 mg, 51% yield). ^{1}H NMR (400 MHz, $CDCl_3$) δ 7.94 (d, J = 7.8 Hz, 4H), 7.63 – 7.50 (m, 2H), 7.47 – 7.34 (m, 4H), 7.30 (d, J = 8.0 Hz, 2H), 7.17 (d, J = 8.0 Hz, 2H), 6.22

(t, J = 55.0 Hz, 1H), 2.54 – 2.44 (m, 1H), 2.36 (s, 3H), 2.35 – 2.27 (m, 1H), 1.54 – 1.41 (m, 2H), 0.92 (t, J = 7.3 Hz, 3H). ^{13}C NMR (100 MHz, $CDCl_3$) δ 208.06 (t, J = 9.0 Hz), 139.23, 139.20, 134.05, 129.51, 129.02, 128.33, 127.89, 125.62, 113.93 (t, J = 245.4 Hz), 113.42, 109.51 (t, J = 24.8 Hz), 36.23, 21.33, 19.88, 13.52. ^{19}F NMR (376 MHz, $CDCl_3$) δ -110.38 – -112.16 (dm, J_{H-F} = 55.1 Hz, 2F). HRMS (ESI) calcd for $[C_{26}H_{25}F_2NO_4S_2Na]^+$ ($[M+Na]^+$): 540.1085, found: 540.1084.

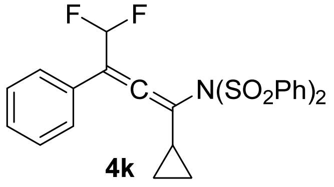

4k

Following the general procedure, compound **4k** was obtained as a white solid (113 mg, 45% yield). ^{1}H NMR (400 MHz, $CDCl_3$) δ 8.00 – 7.88 (m, 4H), 7.50 – 7.40 (m, 2H), 7.33 – 7.27 (m, 4H), 7.27 – 7.22 (m, 5H), 6.11 (t, J = 55.1 Hz, 1H), 1.56 – 1.47 (m, 1H), 0.82 – 0.76 (m, 2H), 0.66 – 0.58 (m, 2H). ^{13}C NMR (100 MHz, $CDCl_3$) δ 207.48 (t, J = 9.0 Hz), 139.55, 134.14, 129.27, 129.10, 128.95, 128.83, 128.56, 128.15, 116.33, 113.79 (t, J = 245.8 Hz), 110.10 (t, J = 24.9 Hz), 14.46, 9.66, 8.73. ^{19}F NMR (376 MHz, $CDCl_3$) δ -111.14 – -111.89 (dm, J_{H-F} = 55.0 Hz, 2F). HRMS (ESI) calcd for $[C_{25}H_{21}F_2NO_4S_2Na]^+$ ($[M+Na]^+$): 524.0772, found: 524.0774.

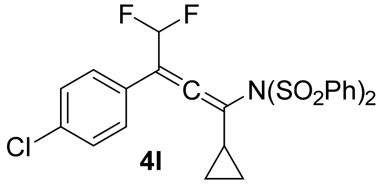

4l

Following the general procedure, compound **4l** was obtained as a white solid (118 mg, 44% yield). ^{1}H NMR (400 MHz, $CDCl_3$) δ 8.03 (d, J = 7.9 Hz, 4H), 7.56 (t, J = 7.6 Hz, 2H), 7.47 – 7.38 (m, 4H), 7.34 – 7.23 (m, 4H), 6.13 (t, J = 55.0 Hz, 1H), 1.64 – 1.55 (m, 1H), 0.83 – 0.92 (m, 2H), 0.72 – 0.64 (m, 2H). ^{13}C NMR (100 MHz, $CDCl_3$) δ 207.69 (t, J = 9.0 Hz), 139.50, 135.33, 134.25, 129.43, 129.18, 128.55, 127.20, 116.75, 113.70 (t, J = 246.0 Hz), 109.12 (t, J = 25.3 Hz), 14.42, 9.63, 8.96. ^{19}F NMR (376 MHz, $CDCl_3$) δ -111.00 (d, J = 54.6 Hz, 2F). HRMS (ESI) calcd for $[C_{25}H_{20}ClF_2NO_4S_2Na]^+$ ($[M+Na]^+$): 558.0383, found: 558.0381.

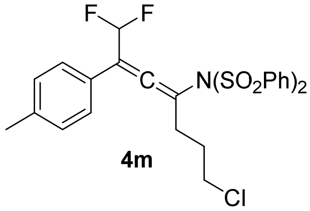

4m

Following the general procedure (the reaction was performed at 100 °C), compound **4m** was obtained as a pale yellow oil (127 mg, 46% yield). ^{1}H NMR (400 MHz, $CDCl_3$) δ

7.87 (d, J = 7.8 Hz, 4H), 7.48 (t, J = 7.5 Hz, 2H), 7.38 – 7.27 (m, 4H), 7.20 (d, J = 8.0 Hz, 2H), 7.09 (d, J = 8.1 Hz, 2H), 6.16 (t, J = 55.0 Hz, 1H), 3.45 (t, J = 6.3 Hz, 2H), 2.63 – 2.52 (m, 1H), 2.44 (dt, J = 15.8, 7.5 Hz, 1H), 2.28 (s, 3H), 1.88 – 1.78 (m, 2H). ^{13}C NMR (100 MHz, $CDCl_3$) δ 207.64 (t, J = 8.6 Hz), 139.61, 139.29, 134.29, 129.76, 129.23, 128.50, 127.97, 125.53, 113.66 (t, J = 245.5 Hz), 112.57, 110.15 (t, J = 24.7 Hz), 43.83, 31.65, 29.56, 21.46. ^{19}F NMR (376 MHz, $CDCl_3$) δ -110.62 – -112.36 (dm, $J_{H\text{-}F}$ = 55.1 Hz, 2F). HRMS (ESI) calcd for $[C_{26}H_{24}ClF_2NO_4S_2Na]^+$ ($[M+Na]^+$): 574.0696, found: 574.0694.

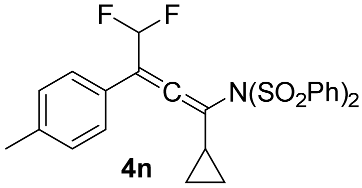

4n

Following the general procedure (the reaction was performed at 90 °C), compound **4n** was obtained as a white solid (134 mg, 52% yield). ^{1}H NMR (400 MHz, $CDCl_3$) δ 8.01 (d, J = 7.9 Hz, 4H), 7.54 (t, J = 7.2 Hz, 2H), 7.44 – 7.34 (m, 4H), 7.23 (d, J = 8.3 Hz, 2H), 7.15 (d, J = 7.7 Hz, 2H), 6.15 (t, J = 54.8 Hz, 1H), 2.36 (s, 3H), 1.63 – 1.53 (m, 1H), 0.92 – 0.80 (m, 2H), 0.74 – 0.64 (m, 2H). ^{13}C NMR (100 MHz, $CDCl_3$) δ 206.92 (d, J = 8.9 Hz), 139.76, 139.47, 139.24, 133.93, 133.78, 129.50, 128.93, 128.87, 128.42, 127.86, 127.01, 125.61, 115.98, 113.67 (t, J = 245.8 Hz), 109.95 (t, J = 24.8 Hz), 21.30, 14.36, 9.35, 8.68. ^{19}F NMR (376 MHz, $CDCl_3$) δ -111.09 (d, J = 55.0 Hz, 2F). HRMS (ESI) calcd for $[C_{26}H_{23}F_2NO_4S_2Na]^+$ ($[M+Na]^+$): 538.0929, found: 538.0928.

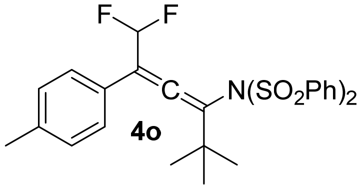

4o

Following the general procedure, compound **4o** was obtained as a pale yellow oil (141 mg, 53% yield). ^{1}H NMR (400 MHz, $CDCl_3$) δ 7.92 (d, J = 18.0 Hz, 2H), 7.70 – 7.59 (m, 3H), 7.49 (t, J = 7.9 Hz, 2H), 7.39 – 7.30 (m, 3H), 7.18 (d, J = 8.0 Hz, 2H), 7.06 (d, J = 8.0 Hz, 2H), 6.23 (t, J = 55.1 Hz, 1H), 2.38 (s, 3H), 1.29 (s, 9H). ^{13}C NMR (100 MHz, $CDCl_3$) δ 209.36 (t, J = 9.0 Hz), 139.20, 138.76, 138.42, 134.29, 133.77, 129.57, 129.05, 128.77, 128.52, 128.02, 125.56, 121.41, 114.30 (t, J = 245.9 Hz), 109.27 (dd, J = 25.8, 24.1 Hz), 37.06, 30.49, 21.34. ^{19}F NMR (376 MHz, $CDCl_3$) δ -109.30 – -111.87 (dm, $J_{H\text{-}F}$ = 54.9 Hz, 2F). HRMS (ESI) calcd for $[C_{27}H_{27}F_2NO_4S_2Na]^+$ ($[M+Na]^+$): 554.1242, found: 554.1237.

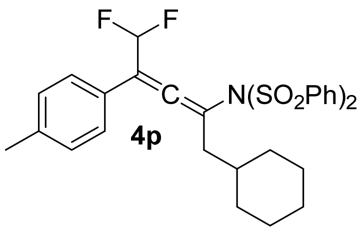

4p

Following the general procedure (the reaction was performed in chloroform), compound **4p** was obtained as a pale yellow oil (163 mg, 57% yield). ^{1}H NMR (400 MHz, $CDCl_3$) δ 7.96 (d, *J* = 8.1 Hz, 4H), 7.66 – 7.32 (m, 6H), 7.30 (d, *J* = 8.0 Hz, 2H), 7.17 (d, *J* = 8.0 Hz, 2H), 6.21 (t, *J* = 55.0 Hz, 1H), 2.42 (dd, *J* = 16.2, 5.8 Hz, 1H), 2.36 (s, 3H), 2.18 (dd, *J* = 16.2, 7.4 Hz, 1H), 1.84 – 1.50 (m, 6H), 1.44 – 1.31 (m, 1H), 1.16 – 1.10 (m, 2H), 0.98 – 0.86 (m, 2H). ^{13}C NMR (100 MHz, $CDCl_3$) δ 208.42 (t, *J* = 9.1 Hz), 139.34, 139.15, 134.06, 129.51, 129.04, 128.38, 128.05, 125.64, 114.03 (t, *J* = 245.6 Hz), 112.14, 109.08 (t, *J* = 25.0 Hz), 41.82, 35.33, 33.28, 33.07, 26.28, 26.02, 25.94, 21.34. ^{19}F NMR (376 MHz, $CDCl_3$) δ -111.18 (d, *J* = 54.9 Hz, 2F). HRMS (ESI) calcd for $[C_{30}H_{31}F_2NO_4S_2Na]^+$ ($[M+Na]^+$): 594.1555, found: 594.1550.

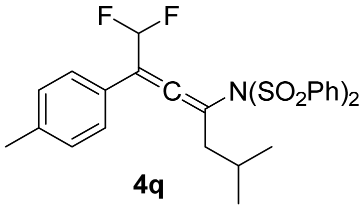

4q

Following the general procedure, compound **4q** was obtained as a pale yellow oil (138 mg, 52% yield). ^{1}H NMR (400 MHz, $CDCl_3$) δ 7.96 (d, *J* = 7.8 Hz, 4H), 7.58 (t, *J* = 7.5 Hz, 2H), 7.51 – 7.35 (m, 4H), 7.31 (d, *J* = 8.0 Hz, 2H), 7.17 (d, *J* = 8.0 Hz, 2H), 6.21 (t, *J* = 55.0 Hz, 1H), 2.43 (dd, *J* = 16.2, 6.0 Hz, 1H), 2.36 (s, 3H), 2.19 (dd, *J* = 16.2, 7.4 Hz, 1H), 1.71 (dp, *J* = 13.1, 6.5 Hz, 1H), 0.93 (d, *J* = 1.6 Hz, 3H), 0.91 (d, *J* = 1.7 Hz, 3H). ^{13}C NMR (100 MHz, $CDCl_3$) δ 208.65 (t, *J* = 9.1 Hz), 139.50, 139.36, 134.25, 129.70, 129.23, 128.56, 128.18, 125.73, 114.15 (t, *J* = 245.6 Hz), 112.82, 109.40 (t, *J* = 24.9 Hz), 43.54, 26.06, 22.89, 22.45, 21.53. ^{19}F NMR (376 MHz, $CDCl_3$) δ -110.34 – -112.07 (dm, $J_{H\text{-}F}$ = 55.1 Hz, 2F). HRMS (ESI) calcd for $[C_{27}H_{27}F_2NO_4S_2Na]^+$ ($[M+Na]^+$): 554.1242, found: 554.1241.

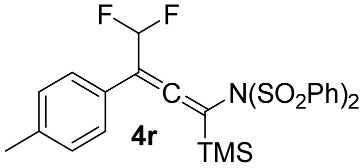

4r

Following the general procedure, compound **4r** was obtained as a pale yellow oil (120 mg, 44% yield). ^{1}H NMR (400 MHz, $CDCl_3$) δ 7.77 – 7.72 (m, 4H), 7.47 – 7.40 (m, 2H), 7.27 (t, *J* = 7.8 Hz, 4H), 7.12 (d, *J* = 8.3 Hz, 2H), 7.02 (d, *J* = 8.0 Hz, 2H), 6.16 (t, *J* = 55.2 Hz, 1H), 2.22 (s, 3H), 0.00 (s, 9H). ^{13}C NMR (100 MHz, $CDCl_3$) δ 213.48 (t,

$J = 8.5$ Hz), 140.05, 139.74, 134.87, 130.42, 129.88, 129.30, 128.33, 126.19, 115.17 (t, $J = 245.2$ Hz), 110.33, 106.73 (t, $J = 24.5$ Hz), 22.19, 0.00. ^{19}F NMR (376 MHz, $CDCl_3$) δ -110.87 (d, J = 55.2 Hz, 2F). HRMS (ESI) calcd for $[C_{26}H_{27}F_2NO_4S_2SiNa]^+$ ($[M+Na]^+$): 570.1011, found: 570.1010.

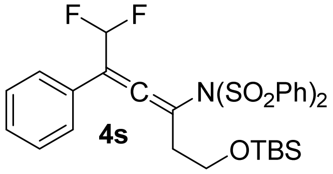

4s

Following the general procedure, compound **4s** was obtained as a pale yellow oil (168 mg, 53% yield). ^{1}H NMR (400 MHz, $CDCl_3$) δ 7.96 (d, J = 8.1 Hz, 4H), 7.59 – 7.50 (m, 2H), 7.46 – 7.34 (m, 8H), 6.32 (t, $J = 55.0$ Hz, 1H), 3.77 (t, $J = 6.3$ Hz, 2H), 2.81 – 2.73 (m, 1H), 2.65 – 2.57 (m, 1H), 0.88 (s, 9H), 0.03 (d, $J = 6.8$ Hz, 6H). ^{13}C NMR (100 MHz, $CDCl_3$) δ 208.93 (t, $J = 9.1$ Hz), 139.13, 134.09, 129.06, 129.02, 128.73, 128.62, 128.35, 128.29, 113.98 (t, $J = 245.5$ Hz), 110.45, 109.33 (d, $J = 24.8$ Hz), 59.83, 37.66, 25.92, 18.35, -5.35, -5.41. ^{19}F NMR (376 MHz, $CDCl_3$) δ -110.00 – -112.29 (dm, $J_{H\text{-}F}$ = 55.0 Hz, 2F). HRMS (ESI) calcd for $[C_{30}H_{35}F_2NO_5S_2SiNa]^+$ ($[M+Na]^+$): 642.1586, found: 642.1577.

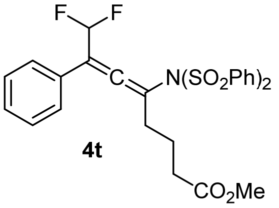

4t

Following the general procedure, compound **4t** was obtained as a pale white oil (117.8 mg, 42% yield). ^{1}H NMR (400 MHz, $CDCl_3$) δ 8.12 – 7.85 (m, 4H), 7.65 – 7.49 (m, 2H), 7.48 – 7.28 (m, 9H), 6.28 (t, J = 55.0 Hz, 1H), 3.64 (s, 3H), 2.68 – 2.53 (m, 1H), 2.47 – 2.39 (m, 1H), 2.36 (t, $J = 7.3$ Hz, 2H), 1.85 – 1.74 (m, 2H). ^{13}C NMR (150 MHz, $CDCl_3$) δ 208.08 (t, $J = 8.5$ Hz), 173.31, 139.15, 134.20, 129.27, 129.12, 128.91, 128.61, 128.42, 128.07, 113.67 (t, $J = 245.6$ Hz), 112.99, 110.06 (t, $J = 24.9$ Hz), 51.74, 33.53, 32.88, 21.78. ^{19}F NMR (376 MHz, $CDCl_3$) δ -110.46 – -112.20 (dm, $J_{H\text{-}F}$ = 55.0 Hz, 2F). HRMS (ESI) calcd for $[C_{27}H_{25}F_2NO_6S_2Na]^+$ ($[M+Na]^+$): 584.0984, found: 584.0984.

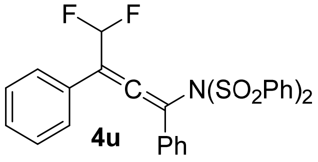

4u

Following the general procedure (the reaction was performed in choroform), compound **4u** was obtained as a pale yellow solid (145 mg, 54% yield). ^{1}H NMR (400 MHz, $CDCl_3$)

δ 8.10 – 7.71 (m, 4H), 7.69 – 7.44 (m, 5H), 7.43 – 7.36 (m, 4H), 7.36 – 7.28 (m, 3H), 7.26 – 7.16 (m, 4H), 6.51 (t, *J* = 54.8 Hz, 1H). ^{13}C NMR (100 MHz, $CDCl_3$) δ 210.42 (t, *J* = 8.8 Hz), 138.87, 134.07, 131.13, 129.45, 129.16, 128.97, 128.76, 128.67, 128.64, 128.25, 128.19, 126.46, 114.16, 113.53 (t, *J* = 246.4 Hz), 111.01 (t, *J* = 25.1 Hz). ^{19}F NMR (376 MHz, $CDCl_3$) δ -110.33 – -112.20 (dm, $J_{H\text{-}F}$ = 54.9 Hz, 2F). HRMS (ESI) calcd for $[C_{28}H_{21}F_2NO_4S_2Na]^+$ ($[M+Na]^+$): 560.0772, found: 560.0769.

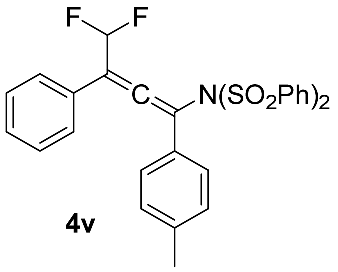

4v

Following the procedure, compound **4v** was obtained as a pale yellow solid (127 mg, 46% yield). ^{1}H NMR (400 MHz, $CDCl_3$) δ 8.01 – 7.69 (s, 4H), 7.62 – 7.36 (m, 5H), 7.34 – 7.28 (m, 4H), 7.20 – 7.08 (m, 4H), 6.96 (d, *J* = 7.9 Hz, 2H), 6.41 (t, *J* = 54.9 Hz, 1H), 2.25 (s, 3H). ^{13}C NMR (100 MHz, $CDCl_3$) δ 209.34 (t, *J* = 8.9 Hz), 138.37, 132.99, 128.35, 127.91, 127.70, 127.44, 127.26, 127.20, 125.38, 113.17, 112.62 (t, *J* = 246.4 Hz), 109.86, 20.21. ^{19}F NMR (376 MHz, $CDCl_3$) δ -110.25 – -112.09 (dm, $J_{H\text{-}F}$ = 55.0 Hz, 2F). HRMS (ESI) calcd for $[C_{29}H_{23}F_2NO_4S_2Na]^+$ ($[M+Na]^+$): 574.0929, found: 574.0925.

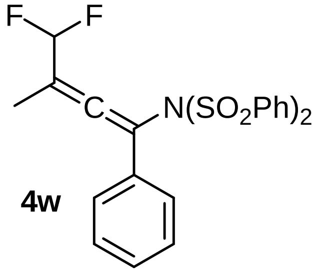

4w

Following the general procedure (the reaction was performed at 100 °C), compound **4w** was obtained as a yellow oil (57.0 mg, 24% yield). ^{1}H NMR (400 MHz, $CDCl_3$) δ 8.12 – 7.84 (d, *J* = 7.8 Hz, 4H), 7.70 – 7.61 (m, 2H), 7.56 – 7.46 (m, 4H), 7.25 – 7.14 (m, 5H), 6.09 (t, *J* = 55.9 Hz, 1H), 1.73 (s, 3H). ^{13}C NMR (100 MHz, CDCl3) δ 207.27 (t, *J* = 9.9 Hz), 138.97, 134.20, 131.72, 128.96, 128.82, 128.77, 128.45, 126.35, 113.06 (t, *J* = 242 Hz), 111.34, 104.95 (t, *J* = 54.5 Hz), 10.49. ^{19}F NMR (376 MHz, $CDCl_3$) δ -113.34 – -116.56 (dm, $J_{H\text{-}F}$ = 56.1 Hz, 2F). HRMS (ESI) calcd for $[C_{23}H_{19}F_2NO_4S_2Na]^+$ ($[M+Na]^+$): 498.0616, found: 498.0617.

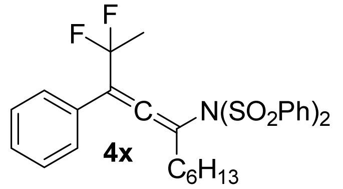

Following the general procedure (the reaction was performed in cyclohexane), compound **4x** was obtained as a pale yellow oil (78 mg, 28% yield); compound **3x** was obtained as a pale yellow oil (124 mg, 46% yield). For **4x**: ^{1}H NMR (400 MHz, $CDCl_3$) δ8.04 – 7.70 (m, 4H), 7.65 – 7.42 (m, 3H), 7.42 – 7.28 (m, 8H), 2.54 – 2.44 (m, 1H), 2.41 – 2.30 (m, 1H), 1.87 (t, *J* = 18.4 Hz, 3H), 1.54 – 1.41 (m, 2H), 1.37 – 1.30 (m, 2H), 1.29 – 1.22 (m, 4H), 0.87 (t, *J* = 6.8 Hz, 3H). ^{13}C NMR (100 MHz, $CDCl_3$) δ 206.46 (t, *J* = 7.6 Hz), 133.90, 130.14, 128.85, 128.77, 128.58, 128.57, 128.54, 128.27, 121.10 (t, *J* = 242.0 Hz), 113.32 (dd, *J* = 32.2, 27.6 Hz), 113.12, 34.38, 31.60, 28.81, 26.54, 24.26 (t, *J* = 27.2 Hz), 22.59, 14.09. ^{19}F NMR (376 MHz, $CDCl_3$) δ -81.92 – -84.95 (qm, J_{H-F} = 18.2 Hz, 2F). HRMS (ESI) calcd for $[C_{29}H_{31}F_2NO_4S_2Na]^+$ ($[M+Na]^+$): 582.1555, found: 582.1552.

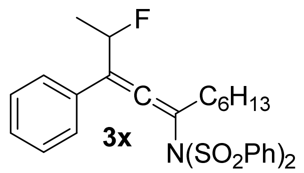

For **3x**: ^{1}H NMR (400 MHz, $CDCl_3$) δ 8.10-7.74 (m, 4H), 7.63-7.21 (m, 11H), 5.59-5.35 (m, 1H), 2.55 – 2.24 (m, 2H), 1.60-1.49 (m, 3H), 1.50 – 1.38 (m, 2H), 1.34 – 1.19 (m, 6H), 0.89-0.80 (m, 3H). ^{13}C NMR (100 MHz, $CDCl_3$) δ 206.42 (d, *J* = 8.1 Hz), 205.93 (d, *J* = 8.3 Hz), 133.79, 132.72, 132.08, 128.82, 128.65, 128.50, 128.44, 128.31, 127.90, 127.70, 114.86 (d, *J* = 21.5 Hz), 114.33 (d, *J* = 18.3 Hz), 112.42 (d, *J* = 1.6 Hz), 111.47 (d, *J* = 1.3 Hz), 88.65 (d, *J* = 173.4 Hz), 87.74 (d, *J* = 173.7 Hz), 34.44 (d, *J* = 1.8 Hz), 34.34, 31.65, 28.80, 26.77, 26.68, 22.60, 22.59, 20.16 (d, *J* = 23.2 Hz), 20.03 (d, *J* = 23.9 Hz), 14.10, 14.09. ^{19}F NMR (376 MHz, $CDCl_3$) δ -165.28, -167.52. HRMS (ESI) calcd for $[C_{29}H_{32}FNO_4S_2Na]^+$ ($[M+Na]^+$): 564.1649, found: 564.1649.

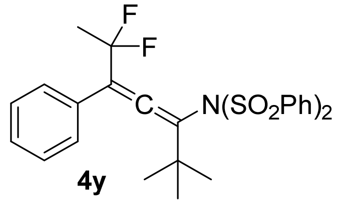

Following the general procedure, compound **4y** was obtained as a white solid (212 mg, 80% yield). ^{1}H NMR (400 MHz, $CDCl_3$) δ 8.07 – 8.02 (m, 2H), 7.68 – 7.62 (m, 1H), 7.52 (t, *J* = 7.9 Hz, 2H), 7.41 – 7.33 (m, 5H), 7.34 – 7.29 (m, 2H), 7.24 – 7.19 (m, 1H),

6.90 – 6.84 (m, 2H), 1.90 (t, *J* = 18.6 Hz, 3H), 1.35 (s, 9H). ^{13}C NMR (100 MHz, $CDCl_3$) δ 207.35 (t, *J* = 7.0 Hz), 139.43, 138.12, 134.25, 133.69, 130.63, 129.50, 129.11, 128.93, 128.83, 128.77, 128.45, 128.39, 121.34, 121.34 (t, *J* = 242.5 Hz), 112.99 (t, *J* = 31.5 Hz), 37.50, 30.83, 24.54 (t, *J* = 27.2 Hz). ^{19}F NMR (376 MHz, $CDCl_3$) δ -80.72 – -83.41 (qm, J_{H-F} = 18.8 Hz, 2F). HRMS (ESI) calcd for $[C_{27}H_{27}F_2NO_4S_2Na]^+$ ($[M+Na]^+$): 554.1242, found: 554.1239.

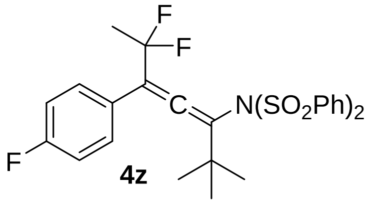

4z

Following the general procedure, compound **4z** was obtained as a white solid (228 mg, 83% yield). ^{1}H NMR (400 MHz, $CDCl_3$) δ 7.96 (d, *J* = 7.3 Hz, 2H), 7.56 (d, *J* = 7.5 Hz, 1H), 7.44 (t, *J* = 7.9 Hz, 2H), 7.37 – 7.26 (m, 4H), 7.24 – 7.15 (m, 1H), 7.00 – 6.87 (m, 4H), 1.81 (t, *J* = 18.6 Hz, 3H), 1.24 (s, 9H). ^{13}C NMR (100 MHz, $CDCl_3$) δ 206.17 (t, *J* = 6.7 Hz), 161.85 (d, *J* = 249.4 Hz), 138.28, 137.22, 133.10, 132.60, 129.47 (d, *J* = 8.0 Hz), 128.25, 127.92, 127.31, 127.27, 125.32 (d, *J* = 3.6 Hz), 120.14, 120.09 (t, *J* = 242.2 Hz), 114.55 (d, *J* = 21.5 Hz), 111.19 (d, *J* = 27.8 Hz), 110.87 (d, *J* = 27.7 Hz), 36.32, 29.56, 23.21 (t, *J* = 27.2 Hz). ^{19}F NMR (376 MHz, $CDCl_3$) δ -81.01 – -83.81 (qm, J_{H-F} = 18.8 Hz, 2F), -112.28 – -112.28 (m, 1F). HRMS (ESI) calcd for $[C_{27}H_{26}F_3NO_4S_2Na]^+$ ($[M+Na]^+$): 572.1148, found: 572.1146.

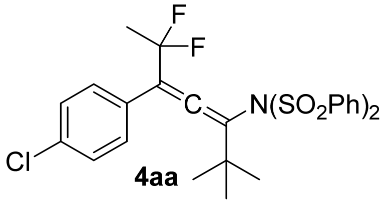

4aa

Following the general procedure (the reaction was performed at 100 °C), compound **4aa** was obtained as a colorless oil (209 mg, 74% yield). ^{1}H NMR (400 MHz, $CDCl_3$) δ 8.00 – 7.92 (m, 2H), 7.60 – 7.53 (m, 1H), 7.43 (t, *J* = 7.9 Hz, 2H), 7.36 – 7.31 (m, 2H), 7.25 – 7.15 (m, 5H), 6.93 – 6.87 (m, 2H), 1.81 (t, *J* = 18.6 Hz, 3H), 1.23 (s, 9H). ^{13}C NMR (100 MHz, $CDCl_3$) δ 207.46 (t, *J* = 7.2 Hz), 139.32, 138.22, 134.81, 134.20, 133.73, 129.96, 129.30, 128.99, 128.85, 128.79, 128.37, 128.34, 121.40, 121.09 (t, *J* = 242.4 Hz), 112.06 (dd, *J* = 32.2, 27.9 Hz), 37.39, 30.62, 24.28 (t, *J* = 27.1 Hz). ^{19}F NMR (376 MHz, $CDCl_3$) δ -80.83 – -83.73 (dm, J_{H-F} = 18.4 Hz, 2F). HRMS (ESI) calcd for $[C_{27}H_{26}ClF_2NO_4S_2Na]^+$ ($[M+Na]^+$): 588.0852, found: 588.0852.

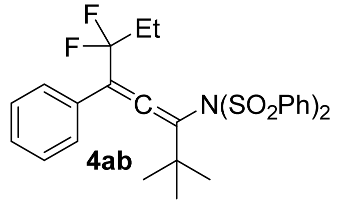

Following the general procedure, compound **4ab** was obtained as a white solid (202 mg, 74% yield). ^{1}H NMR (400 MHz, $CDCl_3$) δ 8.04 (d, *J* = 7.9 Hz, 2H), 7.65 (t, *J* = 7.5 Hz, 1H), 7.51 (t, *J* = 7.7 Hz, 2H), 7.38 – 7.31 (m, 5H), 7.29 – 7.17 (m, 3H), 6.86 (t, *J* = 7.8 Hz, 2H), 2.24 – 1.86 (m, 2H), 1.36 (s, 9H), 0.99 (t, *J* = 7.4 Hz, 3H). ^{13}C NMR (100 MHz, $CDCl_3$) δ 207.25 (t, *J* = 6.1 Hz), 139.25, 137.95, 134.01, 133.48, 130.82, 129.40, 128.90, 128.76, 128.66, 128.58, 128.30, 128.20, 122.11 (t, *J* = 245.4 Hz), 121.16, 112.36 (t, *J* = 27.9 Hz), 37.11, 30.67, 29.92 (t, *J* = 25.9 Hz), 6.39 (t, *J* = 4.8 Hz). ^{19}F NMR (376 MHz, $CDCl_3$) δ -90.43 – -95.13 (ddm, $J_{H\text{-}F}$ = 21.3, 8.7 Hz, 2F). HRMS (ESI) calcd for $[C_{28}H_{29}F_2NO_4S_2Na]^+$ ($[M+Na]^+$): 568.1398, found: 568.1398.

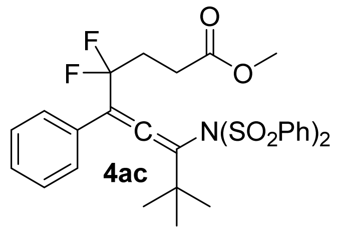

Following the general procedure, compound **4ac** was obtained as a colorless oil (184 mg, 61% yield). ^{1}H NMR (400 MHz, $CDCl_3$) δ 8.03 (d, *J* = 7.2 Hz, 2H), 7.64 (t, *J* = 7.5 Hz, 1H), 7.50 (t, *J* = 7.8 Hz, 2H), 7.37 – 7.34 (m, 5H), 7.31 (d, *J* = 8.4 Hz, 2H), 7.21 (t, *J* = 7.5 Hz, 1H), 6.88 (t, *J* = 7.8 Hz, 2H), 3.65 (s, 3H), 2.55 – 2.26 (m, 4H), 1.34 (s, 9H). ^{13}C NMR (100 MHz, $CDCl_3$) δ 207.30 (t, *J* = 6.3 Hz), 172.49, 139.20, 138.05, 134.12, 133.58, 130.28, 129.39, 128.94, 128.78, 128.55, 128.34, 128.26, 121.46, 120.97 (t, *J* = 246.0 Hz), 112.09 (t, *J* = 27.9 Hz), 51.94, 37.21, 32.02 (t, *J* = 25.2 Hz), 30.65, 26.77 (t, *J* = 3.8 Hz). ^{19}F NMR (376 MHz, $CDCl_3$) δ -91.09 – -93.78 (ddm, $J_{H\text{-}F}$ = 26.2, 10.5 Hz, 2F). HRMS (ESI) calcd for $[C_{30}H_{31}F_2NO_6S_2Na]^+$ ($[M+Na]^+$): 626.1453, found: 626.1456.

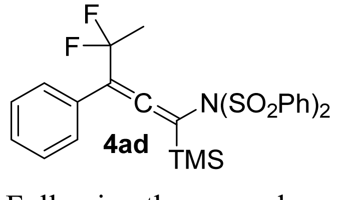

Following the general procedure (the reaction was performed at 90 °C), compound **4ad** was obtained as a pale yellow oil (172 mg, 63% yield). ^{1}H NMR (400 MHz, $CDCl_3$) δ 7.72 – 7.62 (m, 4H), 7.41 – 7.31 (m, 2H), 7.24 – 7.14 (m, 9H), 1.71 (t, *J* = 18.3 Hz, 3H), 0.00 (s, 9H). ^{13}C NMR (100 MHz, $CDCl_3$) δ 211.35 (t, *J* = 6.8 Hz), 139.90, 134.60,

130.78, 129.58, 129.32, 129.28, 129.13, 129.06, 121.83 (t, J = 242.1 Hz), 110.35 (t, J = 29.5 Hz), 109.22, 24.99 (t, J = 27.5 Hz), 0.00. ^{19}F NMR (376 MHz, $CDCl_3$) δ -82.01 – -83.70 (qm, J_{H-F} = 18.2 Hz, 2F). HRMS (ESI) calcd for $[C_{26}H_{27}F_2NO_4S_2SiNa]^+$ ($[M+Na]^+$): 570.1011, found: 570.1009.

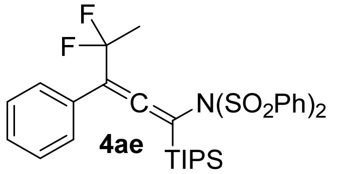

Following the general procedure, compound **4ae** was obtained as a pale yellow oil (133 mg, 42% yield). ^{1}H NMR (400 MHz, $CDCl_3$) δ 7.90 (d, J = 7.8 Hz, 4H), 7.54 (t, J = 7.5 Hz, 2H), 7.41 – 7.33 (m, 4H), 7.33 – 7.28 (m, 5H), 1.51 (t, J = 18.1 Hz, 3H), 1.35 (m, 3H), 1.11 (dd, J = 7.5, 3.3 Hz, 18H). ^{13}C NMR (100 MHz, $CDCl_3$) δ 210.76 (t, J = 5.3 Hz), 139.01, 133.84, 130.67, 129.31, 129.21, 128.65, 128.60, 128.34, 120.82 (t, J = 243.6 Hz), 108.48 (t, J = 27.6 Hz), 107.39, 23.98 (t, J = 27.8 Hz), 18.90, 18.74, 11.99. ^{19}F NMR (376 MHz, $CDCl_3$) δ -82.82 – -84.57 (qm, J_{H-F} = 18.2 Hz, 2F). HRMS (ESI) calcd for $[C_{32}H_{39}F_2NO_4S_2SiNa]^+$ ($[M+Na]^+$): 654.1950, found: 654.1944.

Transformations of Amino-Difluorination Products

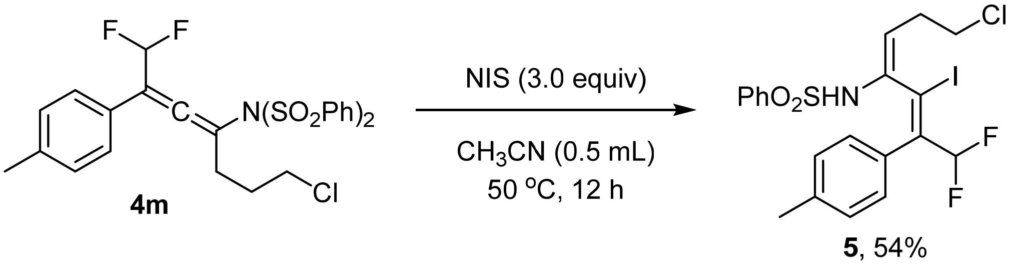

In a flame-dried sealed tube was charged with allene (**4m**, 0.1 mmol), NIS (0.3 mmol), and CH_3CN (0.5 mL). The resulting suspension was stirred at 50 °C for 12 h. Upon completion of the reaction as monitored by TLC, the solvent was concentrated under vacuum. The crude residue was purified by flash column chromatography on silica gel to give product **5** in 54% yield.

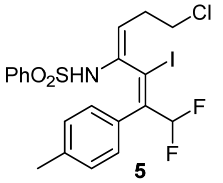

^{1}H NMR (400 MHz, $CDCl_3$) δ 8.00 – 7.87 (m, 2H), 7.68 – 7.59 (m, 1H), 7. 49 – 7.56 (m, 2H), 7.12 (d, J = 7.9 Hz, 2H), 7.04 (d, J = 7.9 Hz, 2H), 6.54 (t, J = 54.6 Hz, 1H), 5.92 (s, 1H), 5.43 – 5.37 (m, 1H), 3.31 – 3.11 (m, 2H), 2.35 (s, 3H), 2.18 – 2.30 (m, 1H), 2.05 – 1.93 (m, 1H). ^{13}C NMR (100 MHz, $CDCl_3$) δ 139.34, 137.77, 134.22, 133.62, 129.31, 129.18, 128.97, 128.12, 127.90, 119.08 (t, J = 239.6 Hz), 114.84, 112.11, 42.48, 31.68, 21.35. ^{19}F NMR (376 MHz, $CDCl_3$) δ -113.76 (d, J = 117.7 Hz, 2F). HRMS (ESI) calcd for $[C_{20}H_{19}F_2NO_2SNa]^+$ ($[M+Na]^+$): 433.0685, found: 433.0693.

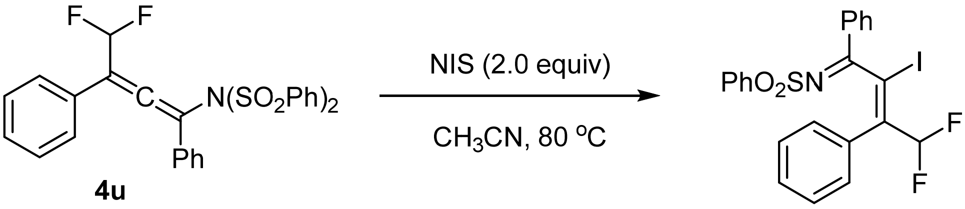

4u → **6**, 50%

In a flame-dried sealed tube was charged with allene (**4u**, 0.1 mmol), NIS (0.3 mmol), and CH_3CN (0.5 mL). The resulting suspension was stirred at 80 °C for 12 h. Upon completion of the reaction as monitored by TLC, the solvent was concentrated under vacuum. The crude residue was purified by flash column chromatography on silica gel to give product **6** in 50% yield.

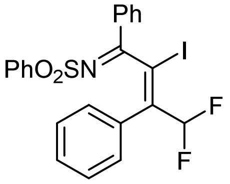

6

^1H NMR (400 MHz, $CDCl_3$) δ 8.13 (d, J = 7.4 Hz, 2H), 8.00 (d, J = 7.3 Hz, 2H), 7.71 – 7.55 (m, 4H), 7.57 – 7.44 (m, 7H), 6.45 (t, J = 54.5 Hz, 1H). ^{13}C NMR (101 MHz, $CDCl_3$) δ 171.86, 144.62 (dd, J = 25.1, 20.5 Hz), 140.30, 136.59, 134.54, 133.37, 132.29, 130.00, 129.37, 129.15, 129.12, 129.09, 128.71, 127.57, 110.46 (t, J = 241.6 Hz), 98.70 (t, J = 8.7 Hz). ^{19}F NMR (376 MHz, $CDCl_3$) δ -80.62 – -83.49 (m, 2F). HRMS (ESI) calcd for $[C_{22}H_{16}F_2INO_2SNa]^+$ ($[M+Na]^+$): 545.9807, found: 545.9804.

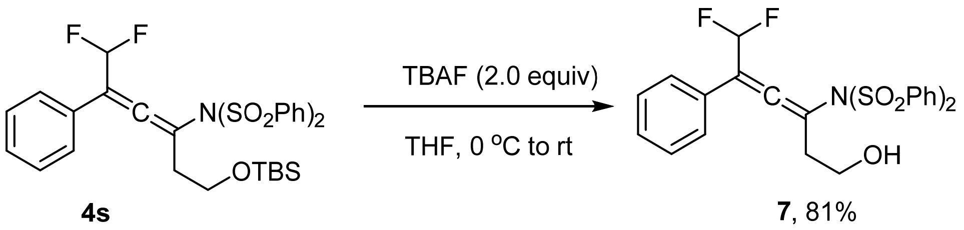

In a flame-dried sealed tube was charged with allene (**4s**, 0.1 mmol), TBAF (2.0 equiv), and THF (1 mL). The resulting suspension was stirred at 0 °C and then warmed up to rt for 2 h. Upon completion of the reaction as monitored by TLC, the solvent was concentrated under vacuum. The crude residue was purified by flash column chromatography on silica gel to give the product **7** in 81% yield.

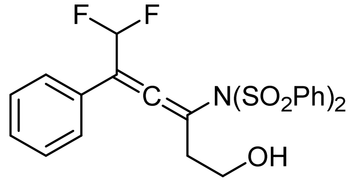

7

^{1}H NMR (400 MHz, $CDCl_3$) δ 7.99 – 7.83 (d, *J* = 7.9 Hz, 4H), 7.63 – 7.51 (m, 2H), 7.47 – 7.29 (m, 9H), 6.28 (t, *J* = 55.0 Hz, 1H), 3.79 (t, *J* = 5.7 Hz, 2H), 2.76 (dt, *J* = 16.0, 5.5 Hz, 1H), 2.67 (dt, *J* = 15.9, 5.9 Hz, 1H). ^{13}C NMR (100 MHz, $CDCl_3$) δ 208.53 (t, *J* = 8.5 Hz), 138.71, 134.25, 129.27, 129.05, 128.89, 128.43, 128.38, 128.13, 113.53 (t, *J* = 245.4 Hz), 109.89, 109.05 (t, *J* = 24.7 Hz), 58.74, 37.69. ^{19}F NMR (376 MHz, $CDCl_3$) δ -111.32 (d, *J* = 67.5 Hz, 2F). HRMS (ESI) calcd for $[C_{24}H_{21}F_2NO_5S_2Na]^+$ ($[M+Na]^+$): 528.0721, found: 528.0721.

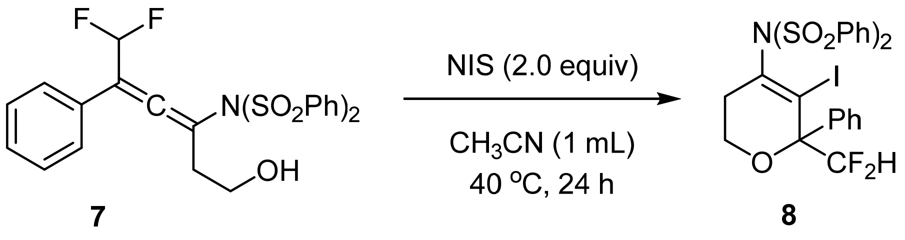

In a flame-dried sealed tube was charged with allene (**7**, 0.1 mmol), NIS (2.0 equiv), and CH_3CN (1 mL). The resulting suspension was stirred at 40 °C for 24 h. Upon completion of the reaction as monitored by TLC, the solvent was concentrated under vacuum. The crude residue was purified by flash column chromatography on silica gel to give the product **8** in 57% yield.

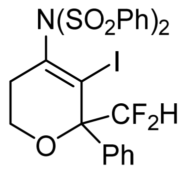

8

^{1}H NMR (400 MHz, $CDCl_3$) δ 8.21 – 8.13 (m, 2H), 8.10 – 8.01 (m, 2H), 7.72 – 7.65 (m, 2H), 7.61 – 7.51 (m, 6H), 7.45 – 7.35 (m, 3H), 6.48 (t, J = 53.6 Hz, 1H), 4.07 (dt, J = 11.5, 4.9 Hz, 1H), 3.86 (ddd, J = 11.8, 7.7, 4.4 Hz, 1H), 2.94 (ddd, J = 17.1, 7.7, 5.3 Hz, 1H), 2.60 (dt, J = 17.1, 4.6 Hz, 1H). ^{13}C NMR (100 MHz, $CDCl_3$) δ 140.72, 140.20, 138.96, 136.63, 136.59, 134.60, 134.41, 129.64, 129.32, 129.28, 129.19, 129.06, 128.60, 128.55, 128.52, 116.22 (t, J = 250.5 Hz), 82.43 (t, J = 19.8 Hz), 60.69, 33.86. ^{19}F NMR (376 MHz, $CDCl_3$) -123.70 – -131.15 (m, 2F). HRMS (ESI) calcd for $[C_{24}H_{20}F_2NO_5S_2Na]^+$ ($[M+Na]^+$): 653.9688, found: 653.9692.

Mechanism Studies

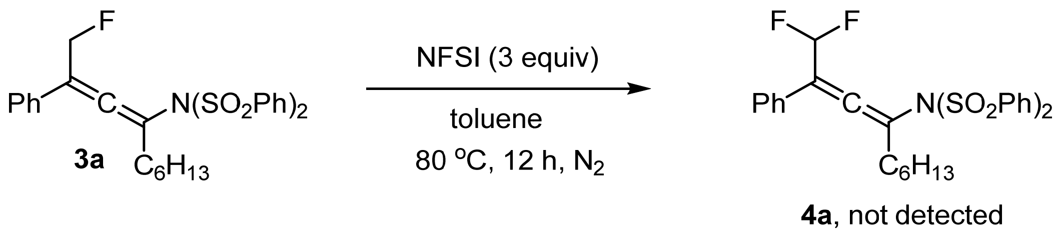

In a flame-dried Schlenk tube, NFSI (0.75 mmol, 3.0 equiv) were dissolved in toluene (0.5 mL) under a nitrogen atmosphere. Then, allene **3a** (0.25 mmol, 1.0 equiv) was added. The reaction mixture was stirred at 80 °C for 12 h. After the reaction completion, the reaction mixture was detected by GC-MS analysis and no desired product **4a** was detected.

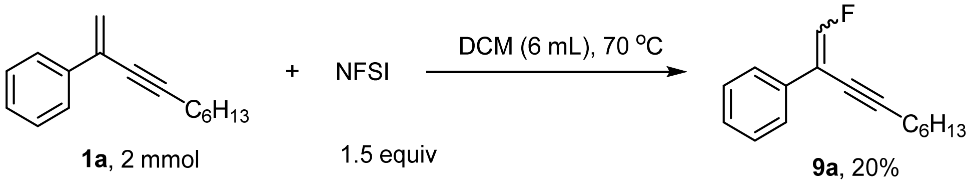

In a flame-dried Schlenk tube, NFSI (3 mmol, 1.5 equiv) and 1,3-enyne **1a** (2 mmol, 1.0 equiv) were dissolved in DCM (6 mL) under a nitrogen atmosphere. The reaction mixture was stirred at 70 °C. After the reaction completion as detected by TLC, the solvent was concentrated under vacuum. The crude residue was purified by flash column chromatography on silica gel to give the product **9a** in 20% yield.

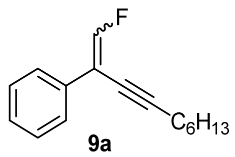

^{1}H NMR (400 MHz, $CDCl_3$) δ 7.49 – 7.44 (m, 2H), 7.37 – 7.26 (m, 3H), 7.28 (d, *J* = 81.3 Hz, 1H), 2.46 (td, *J* = 7.1, 1.6 Hz, 2H), 1.62 (p, *J* = 7.1 Hz, 2H), 1.46 (dq, *J* = 9.6, 6.8 Hz, 2H), 1.38 – 1.28 (m, *J* = 4.6, 3.7 Hz, 4H), 0.90 (t, *J* = 6.8 Hz, 3H). ^{13}C NMR (100 MHz, $CDCl_3$) δ 152.63 (d, *J* = 270.9 Hz), 133.70 (d, *J* = 4.7 Hz), 128.62, 128.02 (d, *J* = 1.5 Hz), 126.11 (d, *J* = 3.5 Hz), 110.30 (d, *J* = 9.5 Hz), 99.46 (d, *J* = 9.0 Hz), 72.54 (d, *J* = 2.4 Hz), 31.35, 28.61, 28.60, 22.59, 19.73, 14.08. ^{19}F NMR (376 MHz, $CDCl_3$) δ -119.55 (s, 1F). MS (EI) calcd for $[C_{16}H_{19}F]^+$: 230.15, found: 230.24.

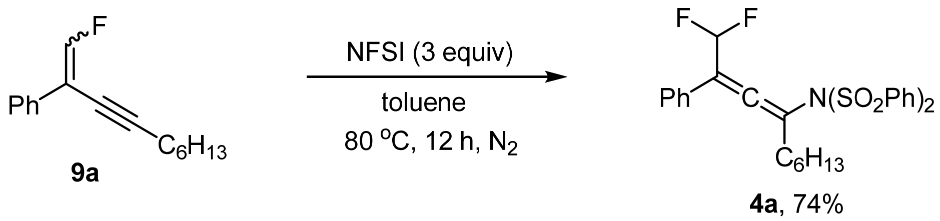

In a flame-dried Schlenk tube, NFSI (0.6 mmol, 3.0 equiv) were dissolved in toluene (0.5 mL) under a nitrogen atmosphere. Then, fluoro-1,3-enyne **9a** (0.2 mmol, 1.0 equiv) was added. The reaction mixture was stirred at 80 °C for 12 h. After the reaction completion, the crude residue was purified by flash column chromatography on silica gel to give the product **4a** in 74% yield.

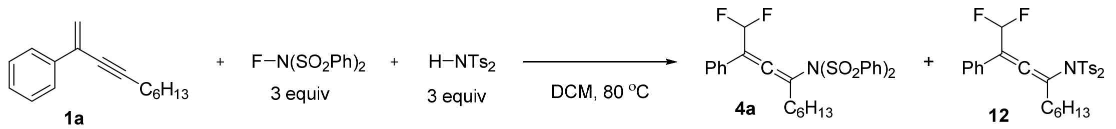

In a flame-dried Seal tube, NFSI (0.6 mmol, 3.0 equiv), $HNTs_2$ (0.6 mmol, 3 equiv) were dissolved in DCM (0.5 mL) under a nitrogen atmosphere. Then, 1,3-enyne **1a** (0.2 mmol, 1.0 equiv) was added. The reaction mixture was stirred at 80 °C for 12 h. After the reaction completion, the crude residue was purified by flash column chromatography on silica gel to give the product **4a** in 32% yield and crossover product **12** in 12% yield.

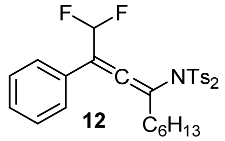

12

^{1}H NMR (400 MHz, $CDCl_3$) δ 7.92 – 7.74 (m, 4H), 7.45 – 7.28 (m, 6H), 7.24 – 7.11 (m, 3H), 6.24 (t, J = 55.0 Hz, 1H), 2.57 – 2.46 (m, 1H), 2.38 (s, 6H), 2.35 – 2.28 (m, 1H), 1.48 – 1.36 (m, 2H), 1.35 – 1.28 (m, 2H), 1.27 – 1.19 (m, 4H), 0.85 (t, J = 6.8 Hz, 3H). ^{13}C NMR (100 MHz, $CDCl_3$) δ 208.54 (t, J = 9.3 Hz), 145.15, 136.28, 129.58, 128.91, 128.77, 128.65, 128.35, 128.00, 114.10 (t, J = 244 Hz), 113.76, 109.22 (t, J = 25.2 Hz), 34.11, 31.58, 30.99, 28.62, 26.42, 22.56, 21.69, 14.06. ^{19}F NMR (376 MHz, $CDCl_3$) δ -110.23 – -111.96 (m, 2F). HRMS (ESI) calcd for $[C_{30}H_{33}F_2NO_4S_2Na]^+$ ($[M+Na]^+$): 596.1711, found: 596.1710.

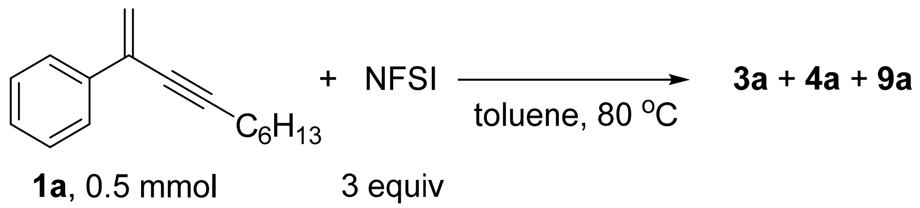

In a flame-dried Seal tube, NFSI (1.5 mmol, 3.0 equiv) were dissolved in toluene (1 mL) under a nitrogen atmosphere. Then, 1,3-enyne **1a** (0.5 mmol, 1.0 equiv) was added. The reaction mixture was stirred at 80 °C. After given time, the mixture was diluted by 2 mL of hexane and cooled by ice. After removal of solvents, the residue was detected by ^{19}F NMR with trifluoromethylbenzene as the internal standard, and the yield was given below (also see, Supplementary Figure 5).

Supplementary Table 1. Kinetic study of reaction.

Reaction time (min)	Yield of **3a** (%)	Yield of **4a** (%)	Yield of **9a** (%)
2	<1	<1	1
4	<1	<1	3
6	<1	<1	3
8	<1	<1	6
10	<1	<1	6
20	1	<1	13
30	2	1	15
60	2	4	20
90	4	10	35
120	5	17	40
720	7	67	<1

Single Crystal Data

CCDC 1891507 **4k**, CCDC 1891508 **4l**, and CCDC 1891506 **5** contain the supplementary crystallographic data. Crystal data and structure refinements of **4k**, **4l**, and **5** are listed in Supplementary Table 2, Supplementary Table 3, and Supplementary Table 4. These data can be obtained free of charge from the Cambridge Crystallographic Data Centre via www.ccdc.cam.ac.uk/data_request/cif.

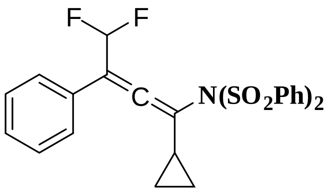

4k

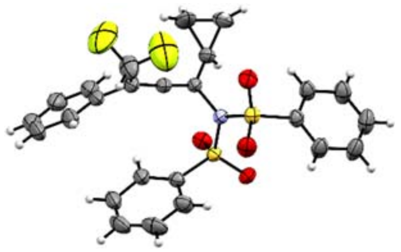

Supplementary Table 2. Crystal data and structure refinement for 4k

Identification code	4k	
Empirical formula	C25 H21 F2 N O4 S2	
Formula weight	501.55	
Temperature	293(2) K	
Wavelength	0.71073 Å	
Crystal system	Monoclinic	
Space group	P 21/c	
Unit cell dimensions	a = 12.273(6) Å	α= 90°.
	b = 15.428(8) Å	β= 103.565(10)°.
	c = 13.609(7) Å	γ = 90°.
Volume	2505(2) $Å^3$	
Z	4	
Density (calculated)	1.330 Mg/m^3	
Absorption coefficient	0.258 mm^{-1}	
F(000)	1040	
Crystal size	0.25 x 0.20 x 0.20 mm^3	
Theta range for data collection	3.418 to 27.518°.	
Index ranges	-13<=h<=15, -20<=k<=19, -17<=l<=17	
Reflections collected	20874	
Independent reflections	5715 [R(int) = 0.0272]	
Completeness to theta = 25.242°	99.5 %	
Absorption correction	Semi-empirical from equivalents	
Max. and min. transmission	1.0000 and 0.8599	
Refinement method	Full-matrix least-squares on F^2	
Data / restraints / parameters	5715 / 12 / 307	
Goodness-of-fit on F^2	1.042	
Final R indices [I>2sigma(I)]	R1 = 0.0637, wR2 = 0.1980	
R indices (all data)	R1 = 0.0767, wR2 = 0.2127	
Extinction coefficient	n/a	
Largest diff. peak and hole	1.194 and -0.585 e.$Å^{-3}$	

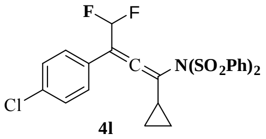

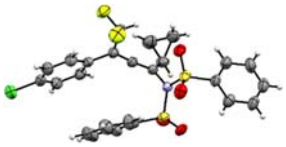

Supplementary Table 3. Crystal data and structure refinement for 4l.

Identification code	4l	
Empirical formula	C25 H20 Cl F2 N O4 S2	
Formula weight	535.99	
Temperature	293(2) K	
Wavelength	0.71073 Å	
Crystal system	Monoclinic	
Space group	P 21	
Unit cell dimensions	a = 7.816(8) Å	α= 90°.
	b = 15.143(16) Å	β= 91.30(2)°.
	c = 10.494(9) Å	γ = 90°.
Volume	1242(2) $Å^3$	
Z	2	
Density (calculated)	1.434 Mg/m^3	
Absorption coefficient	0.370 mm^{-1}	
F(000)	552	
Crystal size	0.25 x 0.10 x 0.07 mm^3	
Theta range for data collection	2.362 to 27.500°.	
Index ranges	-9<=h<=10, -19<=k<=19, -10<=l<=13	
Reflections collected	10637	
Independent reflections	5442 [R(int) = 0.0522]	
Completeness to theta = 25.242°	99.7 %	
Absorption correction	Semi-empirical from equivalents	
Max. and min. transmission	1.0000 and 0.8^{1}03	
Refinement method	Full-matrix least-squares on F^2	
Data / restraints / parameters	5442 / 1 / 317	
Goodness-of-fit on F^2	1.059	
Final R indices [I>2sigma(I)]	R1 = 0.0553, wR2 = 0.1004	
R indices (all data)	R1 = 0.1099, wR2 = 0.1268	
Absolute structure parameter	0.02(11)	
Extinction coefficient	n/a	
Largest diff. peak and hole	0.184 and -0.238 e.$Å^{-3}$	

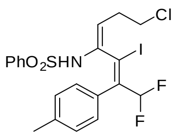

5

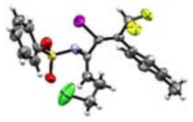

Supplementary Table 4. Crystal data and structure refinement for 5.

Identification code	5
Empirical formula	$C_{20}H_{19}ClF_2INO_2S$
Formula weight	537.77
Temperature/K	99.99(10)
Crystal system	monoclinic
Space group	P21/c
a/Å	11.8972(2)
b/Å	9.67490(10)
c/Å	19.2882(3)
α/°	90
β/°	106.101(2)
γ/°	90
Volume/Å^3	2133.07(6)
Z	4
ρ_{calc}g/cm^3	1.675
μ/mm^{-1}	9.423
F(000)	1064.0
Crystal size/mm^3	0.25 × 0.2 × 0.1
Radiation	GaKα (λ = 1.3405)
2Θ range for data collection/°	6.724 to 107.706
Index ranges	-14 ≤ h ≤ 14, -11 ≤ k ≤ 5, -23 ≤ l ≤ 23
Reflections collected	14357
Independent reflections	3910 [Rint = 0.0417, R_{sigma} = 0.0337]
Data/restraints/parameters	3910/0/254
Goodness-of-fit on F^2	1.056
Final R indexes [I>=2σ (I)]	R1 = 0.0317, wR$_2$ = 0.0846
Final R indexes [all data]	R1 = 0.0327, wR$_2$ = 0.0853
Largest diff. peak/hole / e Å^{-3}	1.16/-1.10

Computational Method and Details

The formations of reactive intermediate **9**, fluoroamination and difluoroamination products, **3** and **4**, were studied by density functional theory (DFT) at B3LYP[8, 9]-D3[10, 11]/Def2-SVP[12, 13] level of theory in gas-phase for geometrical optimizations, thermal energy calculations and frequency analyses, in which the D3 dispersion correction is the original D3 damping function. Transition state structures were searched by simply performing a crude relaxed potential energy surface (RPES) scan connecting reactants and products, and then optimized by the rational function optimization (RFO) method of TS.[14] In addition, all of transition states were verified to have only one imaginary frequency, and the intrinsic reaction coordinate (IRC) path calculations were performed to confirm the correctness of the imaginary frequency and the connection between transition states and intermediates or products.[15] The integration grid option are required at ultrafine for all of calculations. In addition, the initial force constants calculations, connectivity for geometrical specification and initial guess wavefunction reading are employed for the intermediates that generated from the displacement manual TS or IRC path. Single point energies based upon the optimized structures were calculated at the B3LYP-D3/Def2-TZVP[12, 13] level of theory with SMD solvation model calculation in toluene solution,[16] and the reported Gibbs free energy is obtained by adding the solution-phase electronic energy with the gas-phase Gibbs free energy correction for saving the computational time consumption.

To confirm the validity of optimization in gas-phase and the concerted process of the fluoride transfer to **1** accompanying with DBSI generation, transition state of fluoride transfer **TS1** was optimized at B3LYP-D3/Def2-SVP level with SMD solvation method in toluene and was verified by IRC path calculation as well (cf. Supplementary Figure 3a and 3b). The concerted process of **TS1** is invariant in SMD calculation. Although the imaginary frequency of **TS1** does not involve any CH stretch, it is logical for the hydrogen transfer back to $N(SO_2Ph)_2$ since the initial kinetic energy of fluorine atom can transfer to the rotational energy of C1-C2 bond and then fling the proton out. The C1-H bond elongates bit by bit from ~1.09 to ~1.21 Å in gas-phase and to ~1.15 Å in toluene, respectively, and no local minima can be found in both paths. The distance variation of C1-H bond was also depicted in Supplementary Figure 3. On the other hand, the IRC path starting from **TS1'** is also provided for depicting the directly 1,2-addition. The same C1-C2 bond rotation can be observed in this path as well as in the path through **TS1**. However, the C1-H bond distance almost doesn't change; instead, the C2-N distance reduces to ~2.89 from ~3.79 Å (cf. Supplementary Figure 4).

Moreover, natural bond orbital (NBO) charge analysis[17, 18, 19, 20] was carried out at B3LYP-D3/Def2-TZVP/(SMD-toluene) level for all of stationary points. It is noteworthy that the sum of NBO charges are all positive for these enynes moiety in **TS1**, **TS2** and **TS4** although the partial charges on the fluorine atom is negative (Supplementary Table 5). This indicates that the 1,3-enynes are oxidized

by NFSI while fluoride transfer from NFSI to the 1,3-enyne. All calculations were performed by the Gaussian 09 package.[21]

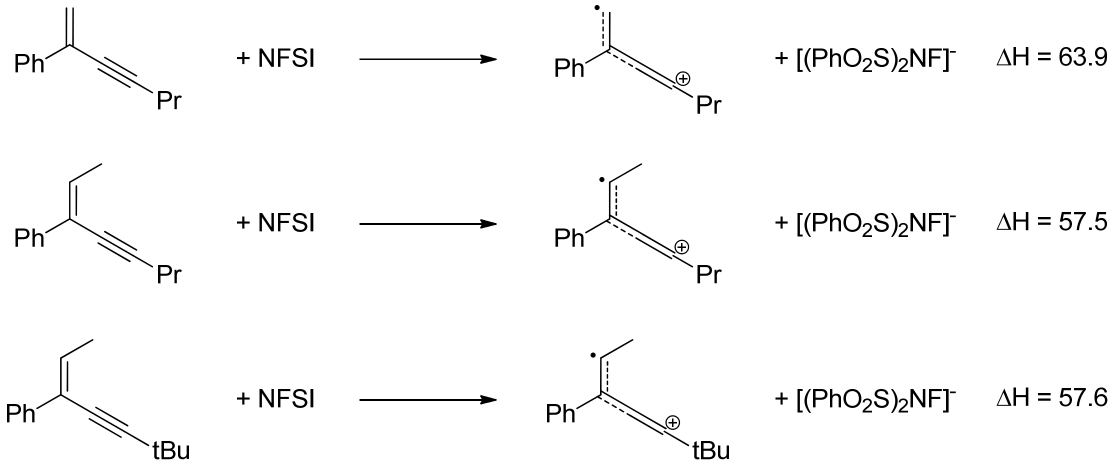

Supplementary Figure 1. Enthalpies for three kinds of radical cation species oxidized by NFSI.

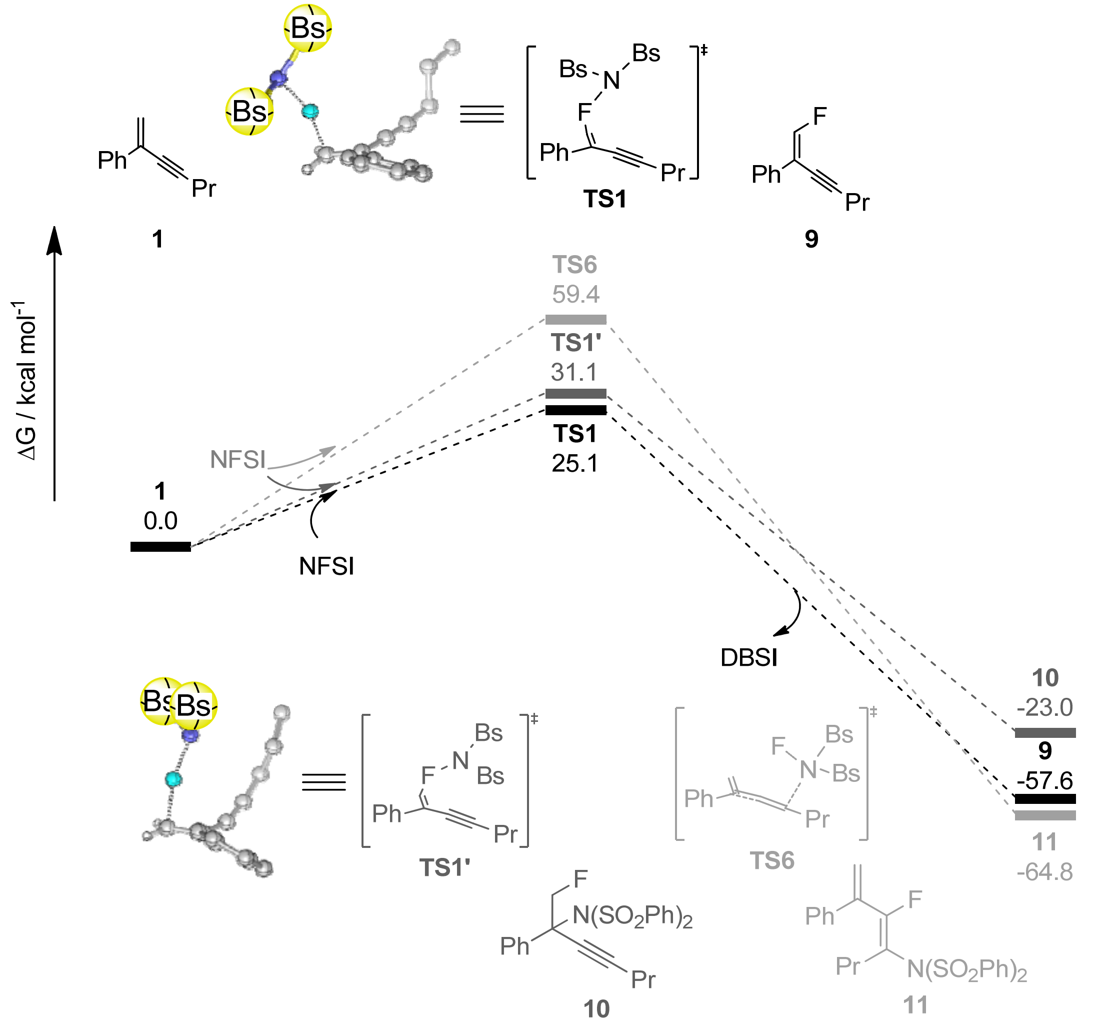

Supplementary Figure 2. The free energy profiles for reaction 1,3-enyne, **1**, and NFSI with different orientations.

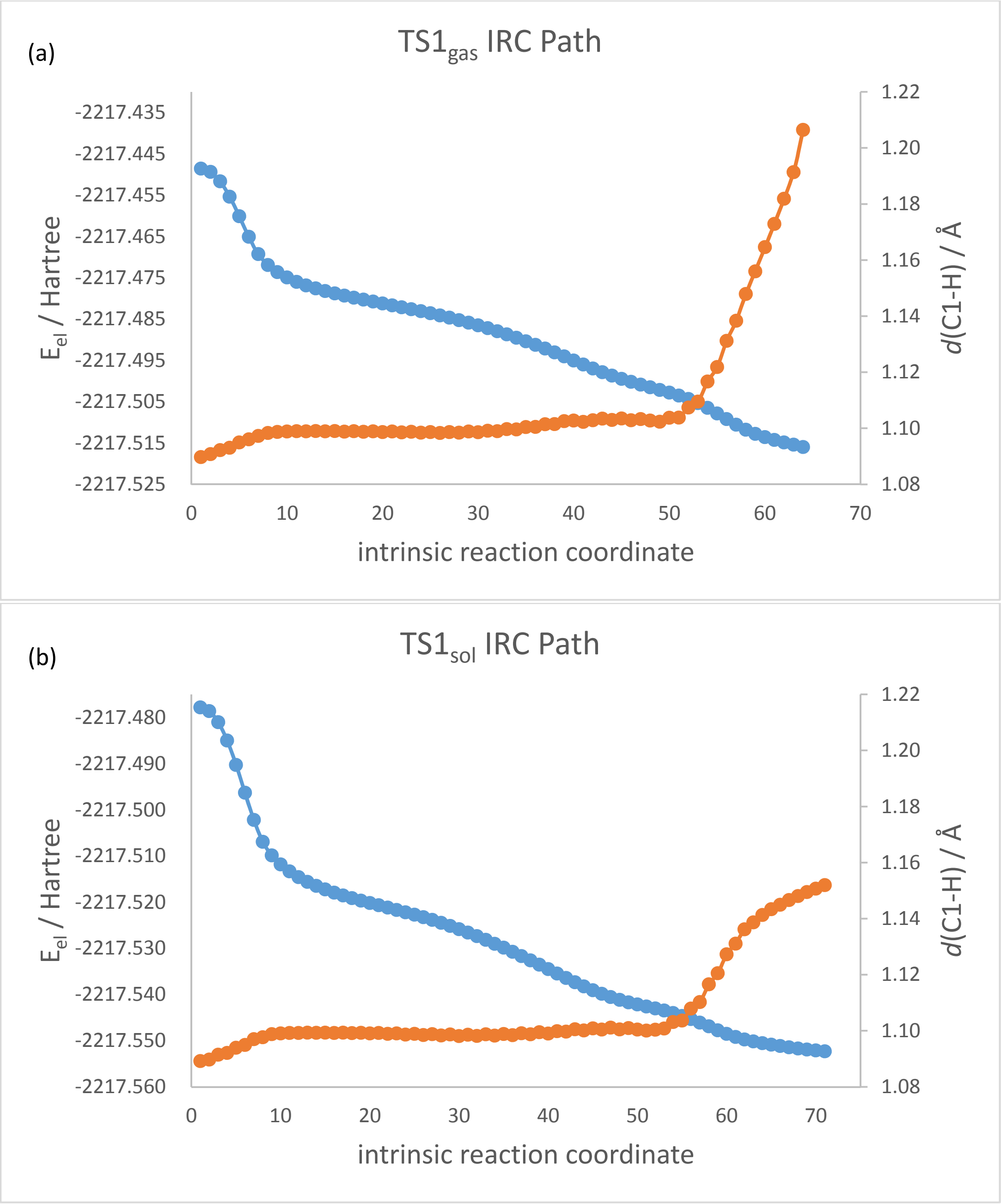

Supplementary Figure 3. The **TS1** IRC paths (blue) calculated (a) in gas-phase and (b) in toluene with the bond distance variations of C1-H bond (red).

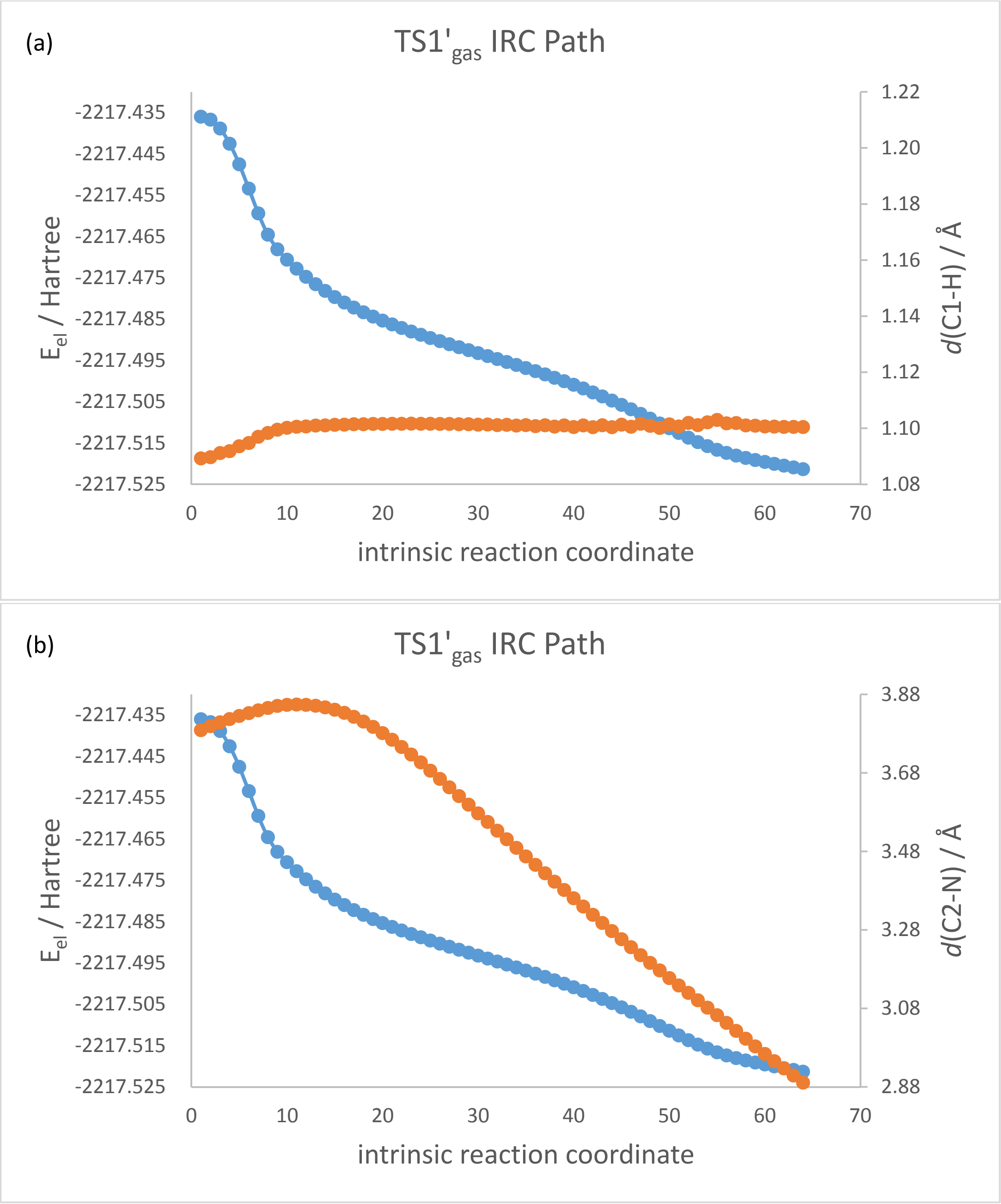

Supplementary Figure 4. The **TS1’** IRC paths (blue) calculated in gas-phase with the distance variations of (a) C1-H bond and (b) C2-N (red).

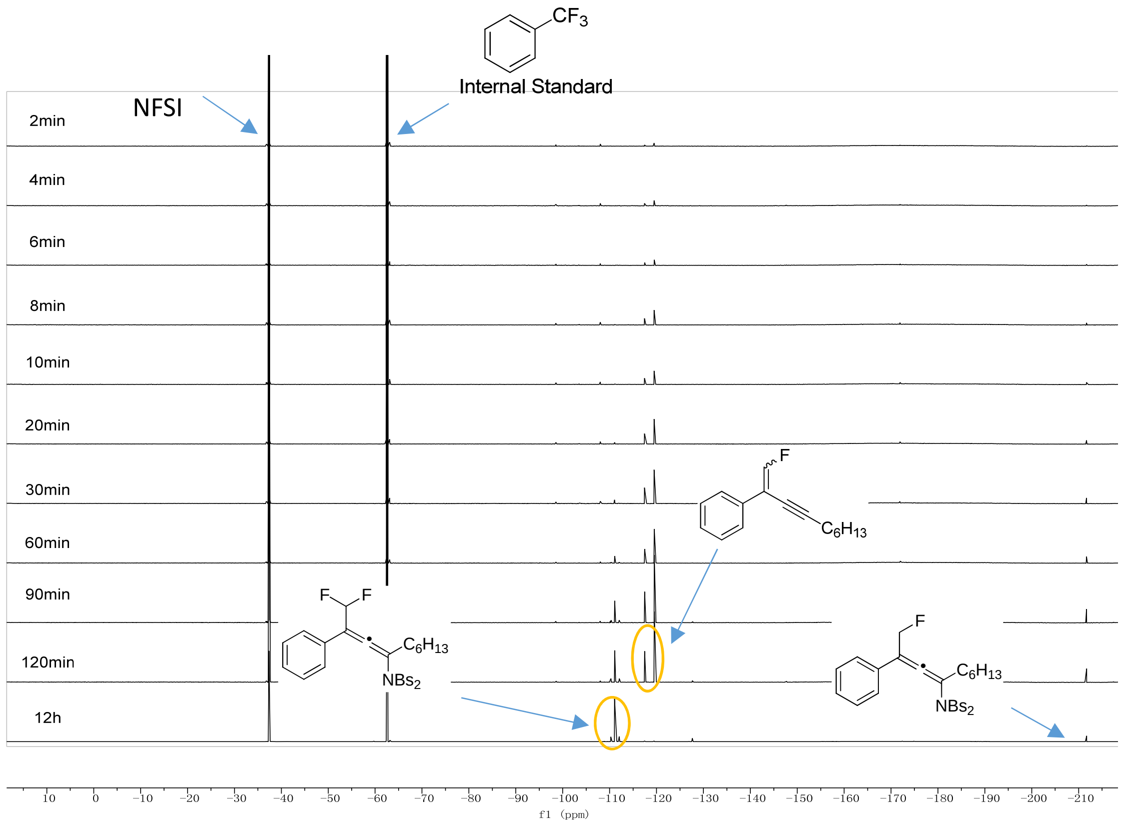

Supplementary Figure 5. The ^{19}F NMR of crude reaction solution with trifluoromethylbenzene as the internal standard.

Supplementary Table 5. The NBO charges in **TS1**, **TS1’**, **TS2** and **TS4**.

	**TS1**	**TS1’**	**TS2**	**TS4**
Enyen	0.57	0.62	0.55	0.52
F atom	-0.38	-0.38	-0.40	-0.40
$N(SO_2Ph)_2$	-0.19	-0.24	-0.15	-0.11
BDSI	–	–	–	-0.01

Supplementary Table 6. Electronic potential energies and correction to zero point energies, thermal energies, enthalpies, free energies (in Hartree) and imaginary frequencies (cm^{-1}) of optimized structures calculated at the B3LYP-D3/Def2-TZVP/(SMD-toluene)//B3LYP-D3/Def2-SVP.

Entry	Structure	$E_{el,sol}$	$E_{el,gas}$	$cZPE_{gas}$	$cU_{298,gas}$	$cH_{298,gas}$	$cG_{298,gas}$	Imaginary Frequency
1	**1**	-503.964227	-503.403189	0.228032	0.240939	0.241883	0.185730	
2	NFSI	-1715.505098	-1714.054493	0.209285	0.226943	0.227887	0.162097	
3	**TS1**	-2219.452829	-2217.448588	0.437738	0.469433	0.470377	0.371290	-501.6486
4	**9**	-603.243725	-602.560905	0.220753	0.234511	0.235455	0.176945	
5	DBSI	-1616.317263	-1614.985748	0.218237	0.235168	0.236112	0.170799	
6	**TS2**	-2318.731515	-2316.607977	0.430051	0.462633	0.463577	0.362383	-501.0049
7	**int1**	-2318.816597	-2316.690765	0.433334	0.465784	0.466728	0.365616	
8	**int2**	-3935.156813	-3931.730862	0.652614	0.702697	0.703641	0.566001	
9	**TS3**	-3935.141204	-3931.722895	0.651641	0.700895	0.701839	0.565898	-230.2572
10	**4**	-2318.876404	-2316.767066	0.436597	0.467946	0.468890	0.372037	
11	**TS4**	-3835.793007	-3832.487833	0.658197	0.707963	0.708907	0.571184	-440.8247
12	**int3**	-3835.878977	-3832.574270	0.660717	0.710080	0.711024	0.574438	
13	**TS5**	-3835.862223	-3832.564975	0.659560	0.707910	0.708855	0.576304	-265.6259
14	**3**	-2219.587518	-2217.596415	0.444265	0.474936	0.475880	0.380573	
15	**TS1'**	-2219.441147	-2217.436020	0.437026	0.468994	0.469939	0.369235	-486.9694
16	**10**	-2219.552954	-2217.568254	0.443167	0.473965	0.474909	0.379428	
17	**TS6**	-2219.399672	-2217.402607	0.436841	0.468420	0.469364	0.372891	-528.2098
18	**11**	-2219.605279	-2217.618615	0.443676	0.473969	0.474913	0.380562	

NMR Spectra

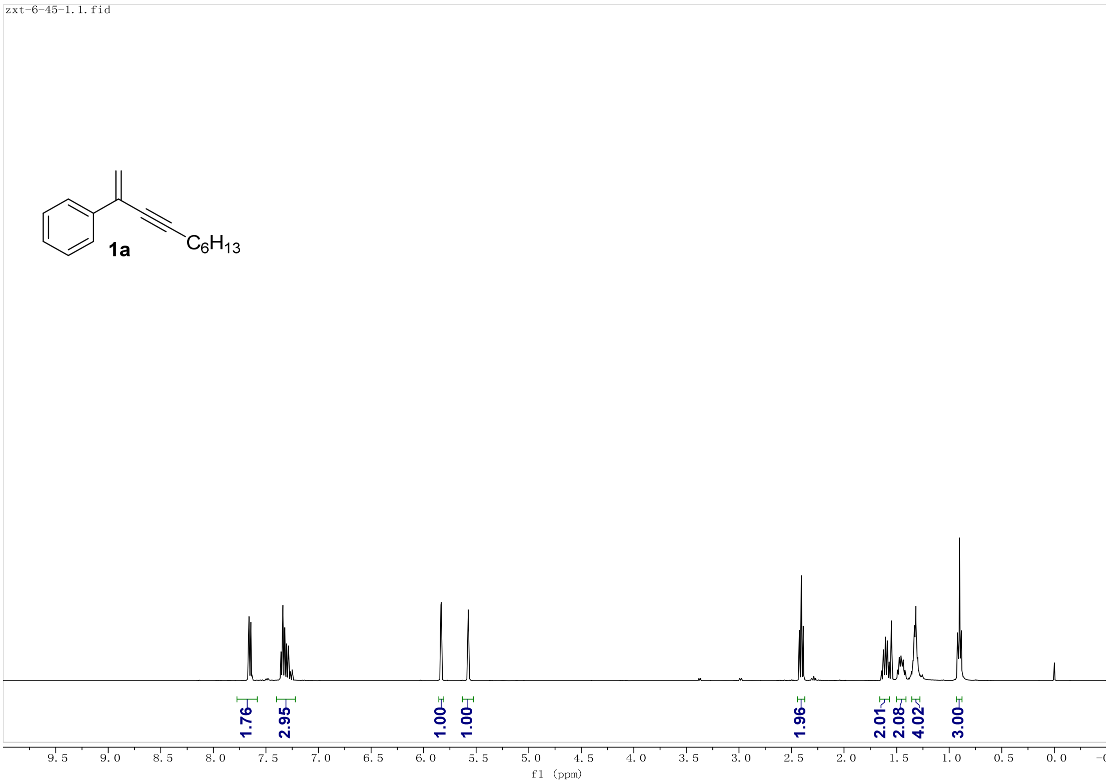

Supplementary Figure 6. **^{1}H NMR spectrum of compound 1a.**

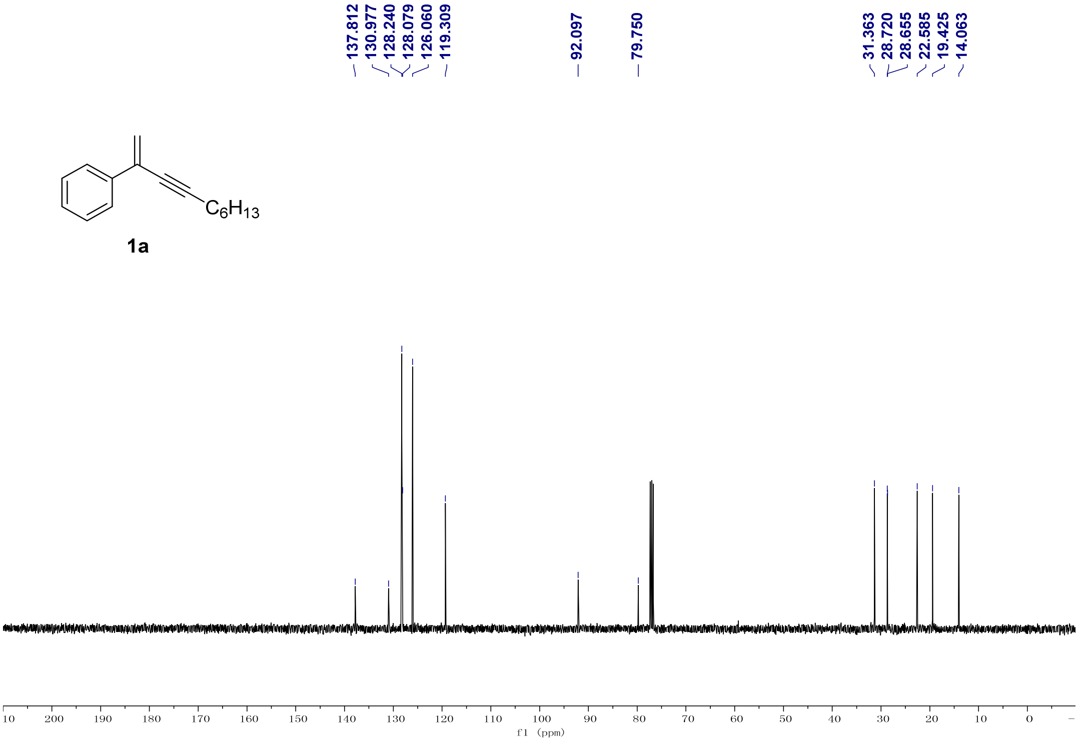

Supplementary Figure 7. **^{13}C NMR spectrum of compound 1a.**

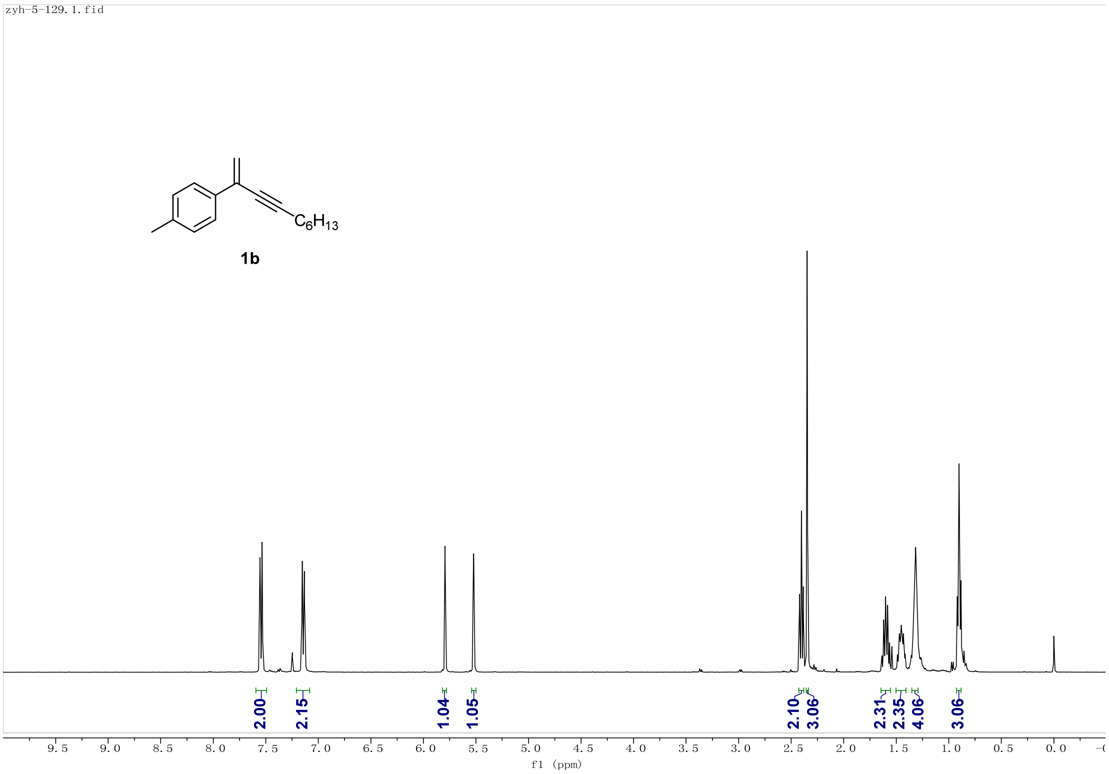

Supplementary Figure 8. ^{1}H NMR spectrum of compound 1b.

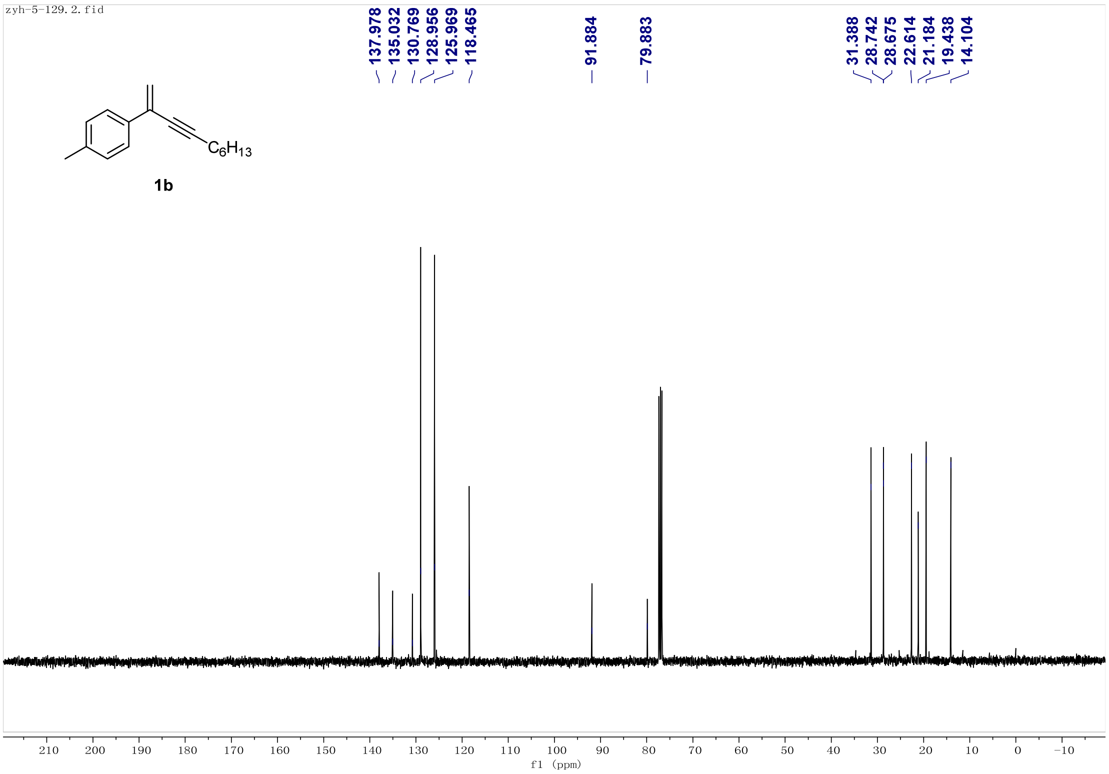

Supplementary Figure 9. ^{13}C NMR spectrum of compound 1b.

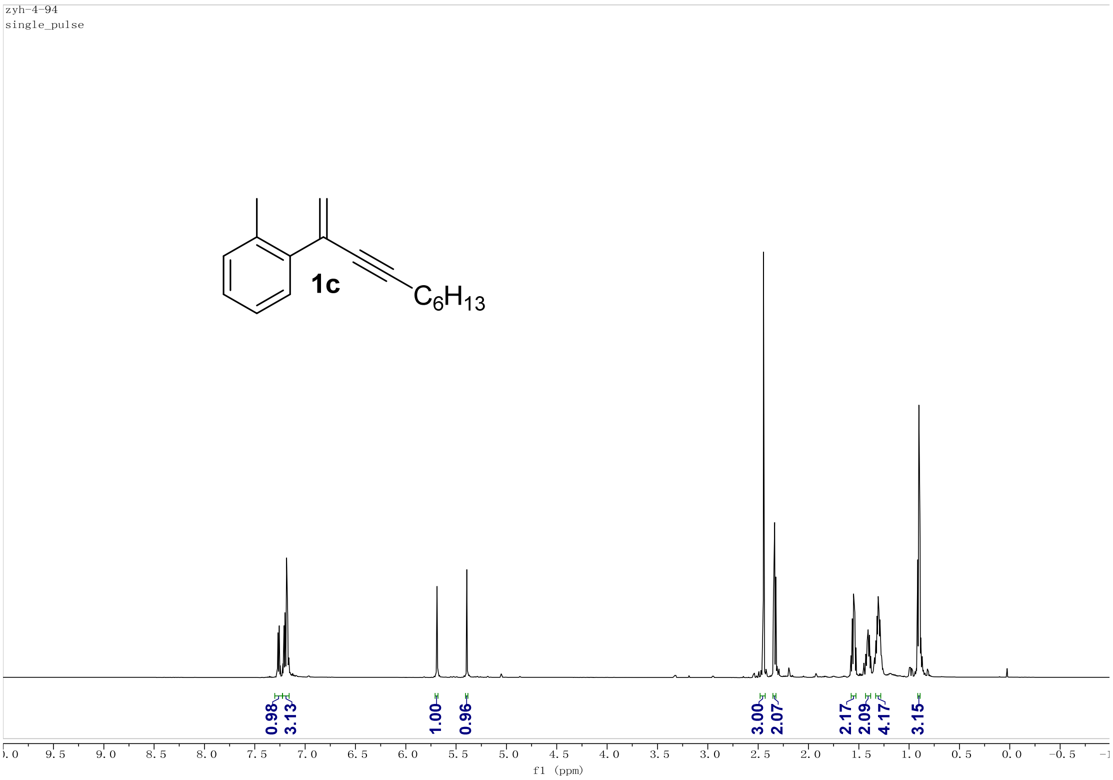

Supplementary Figure 10. ^{1}H NMR spectrum of compound 1c.

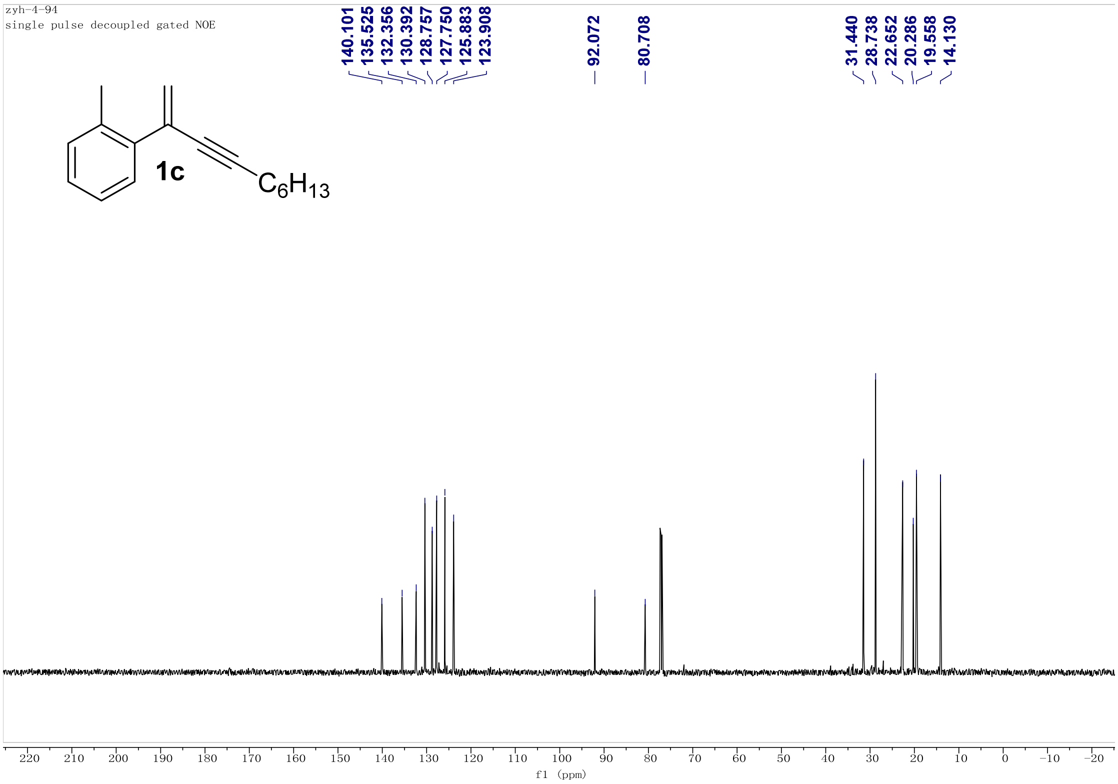

Supplementary Figure 11. ^{13}C NMR spectrum of compound 1c.

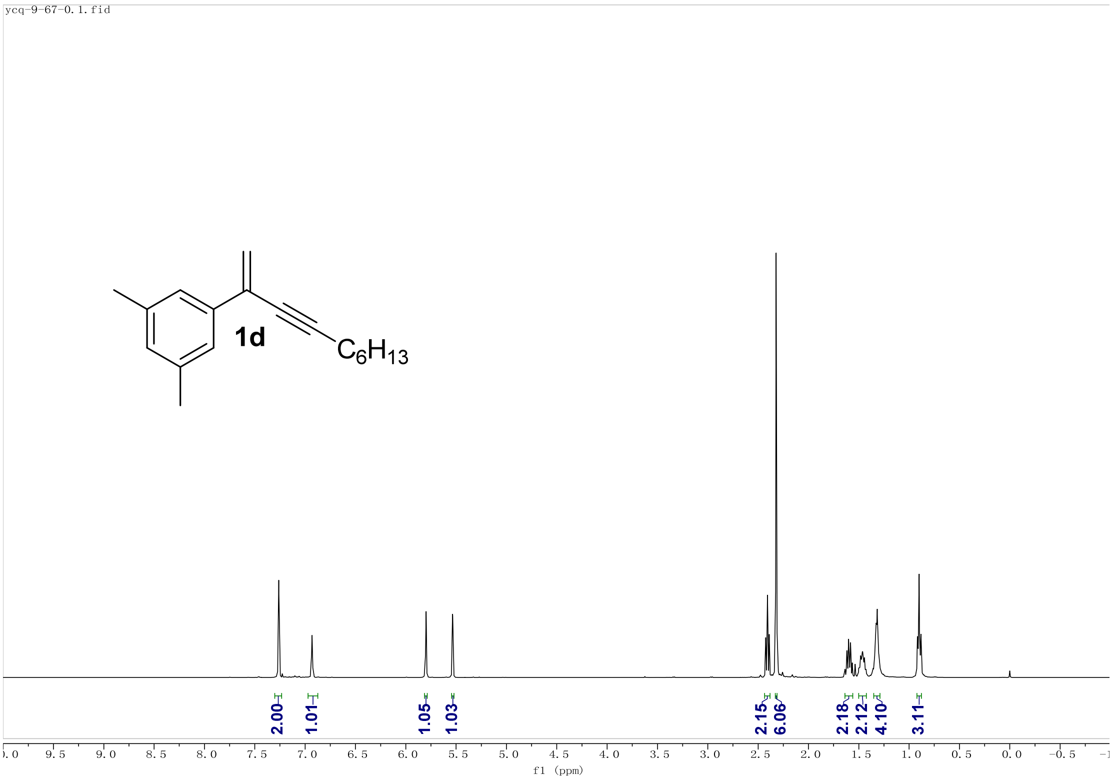

Supplementary Figure 12. **^{1}H NMR spectrum of compound 1d.**

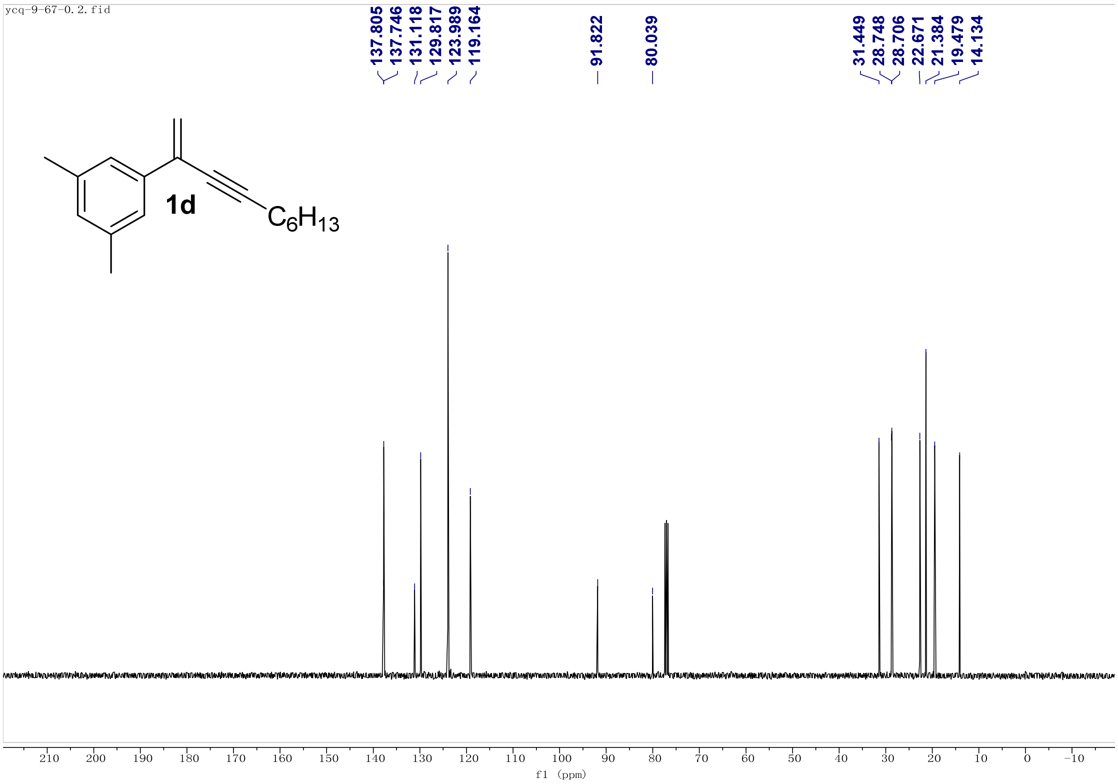

Supplementary Figure 13. **^{13}C NMR spectrum of compound 1d.**

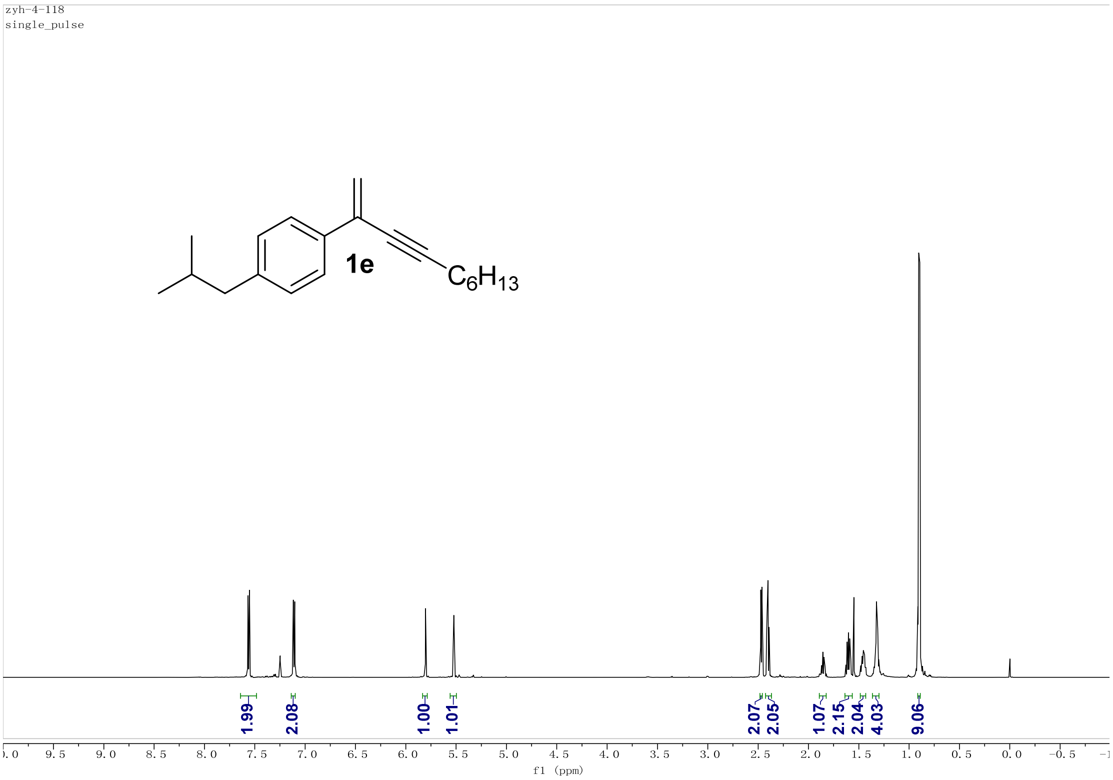

Supplementary Figure 14. ^{1}H NMR spectrum of compound 1e.

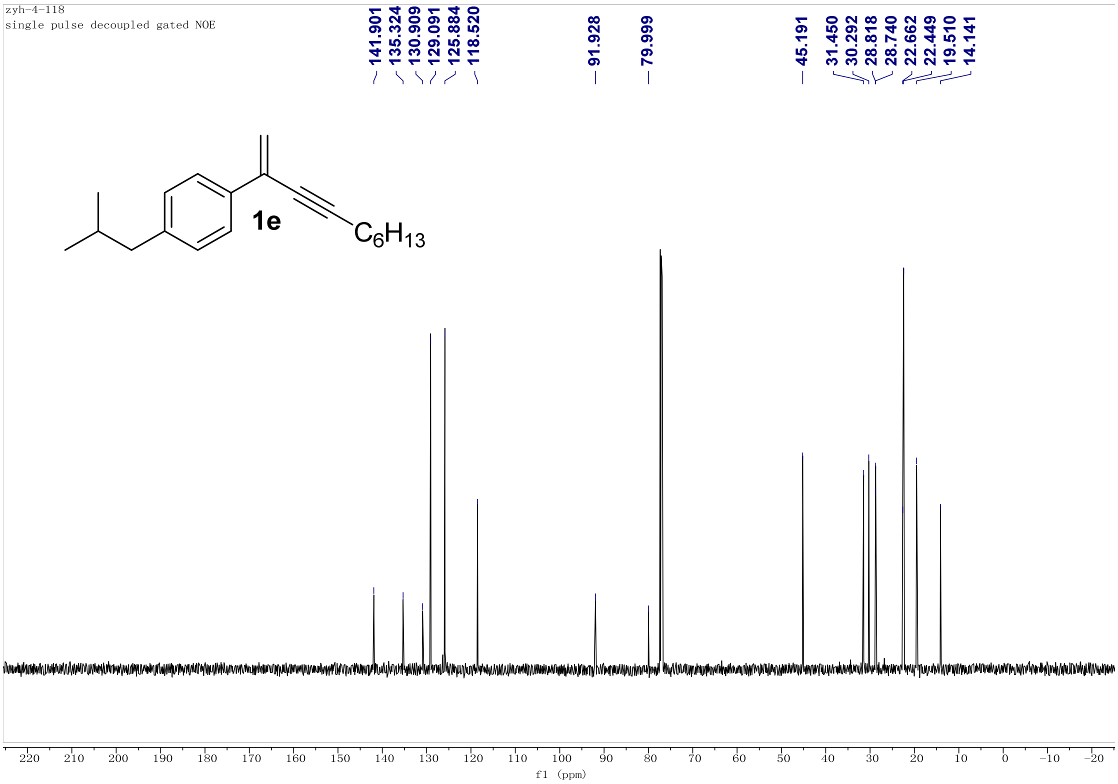

Supplementary Figure 15. ^{13}C NMR spectrum of compound 1e.

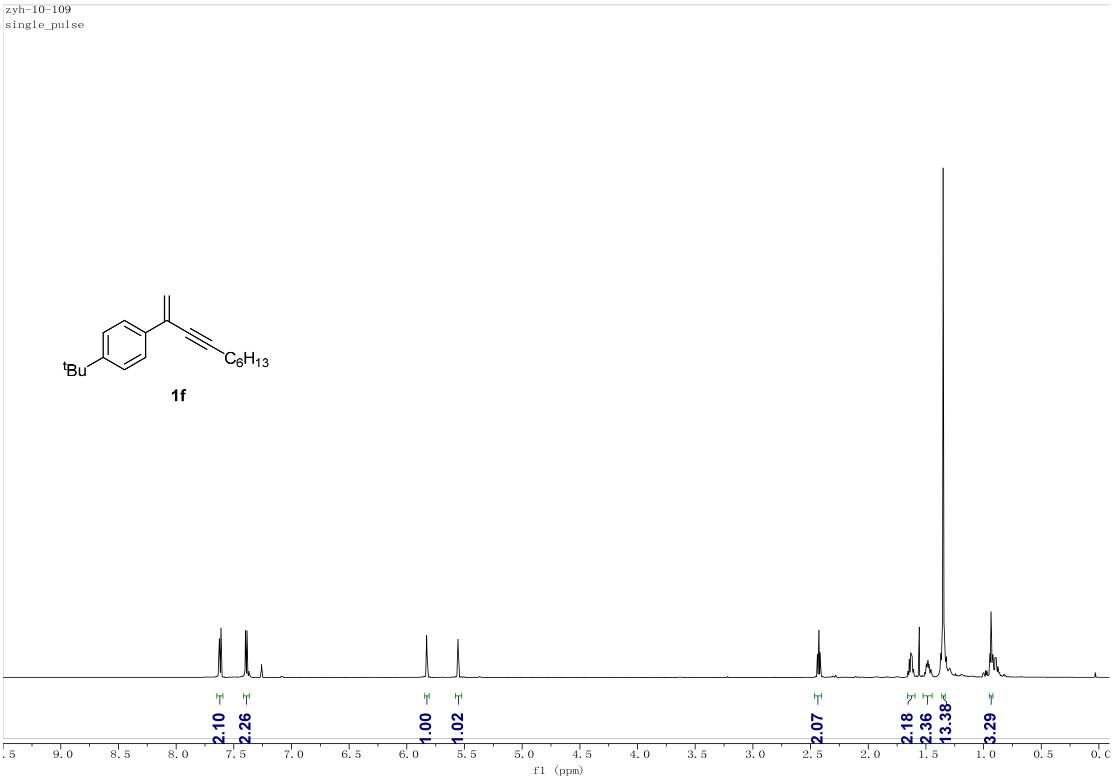

Supplementary Figure 16. ^{1}H NMR spectrum of compound 1f.

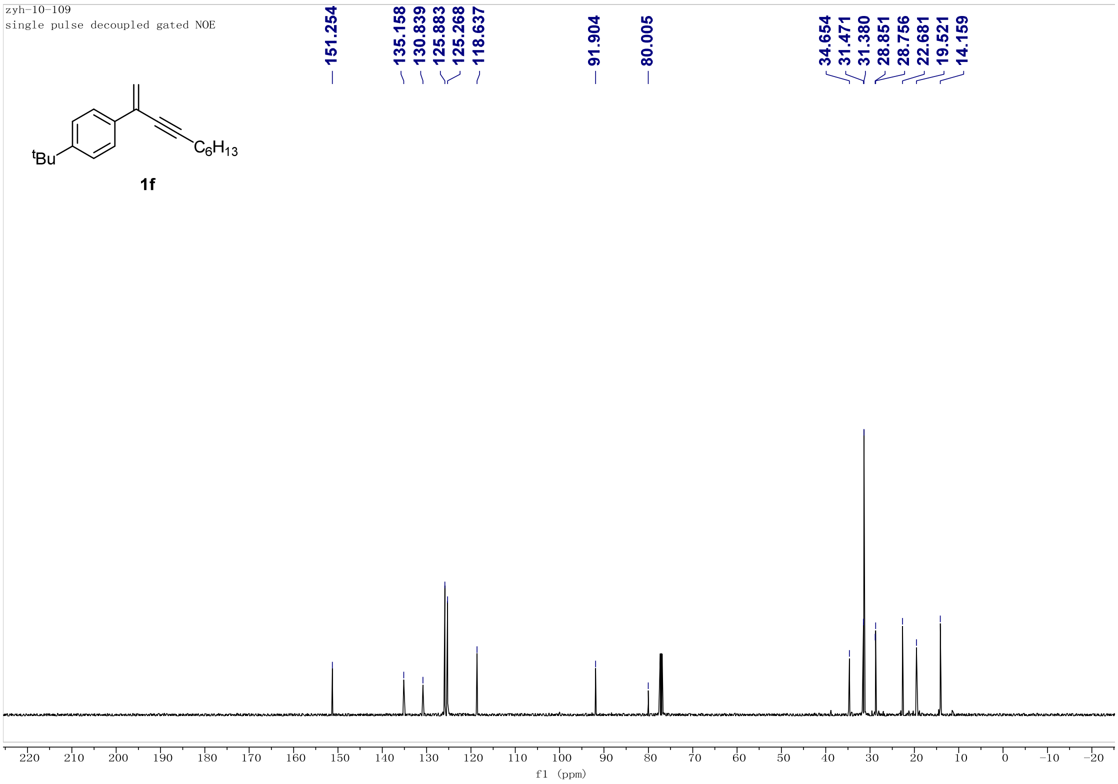

Supplementary Figure 17. ^{13}C NMR spectrum of compound 1f.

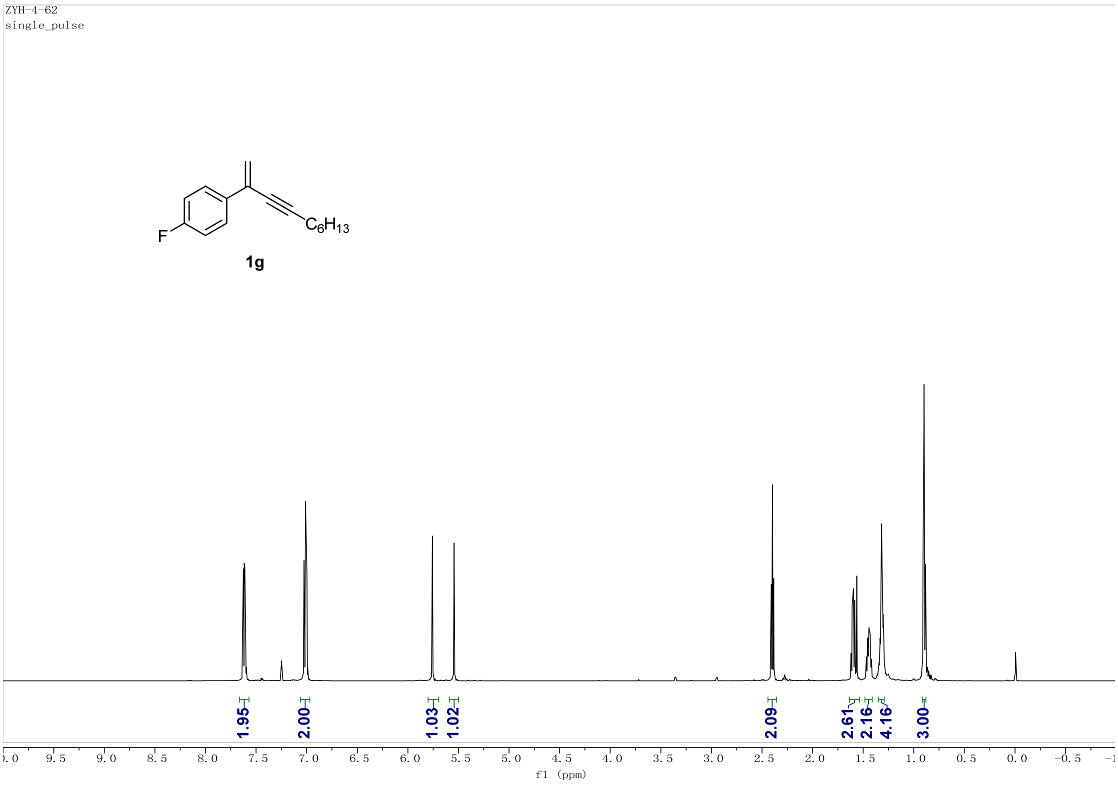

Supplementary Figure 18. ^{1}H NMR spectrum of compound 1g.

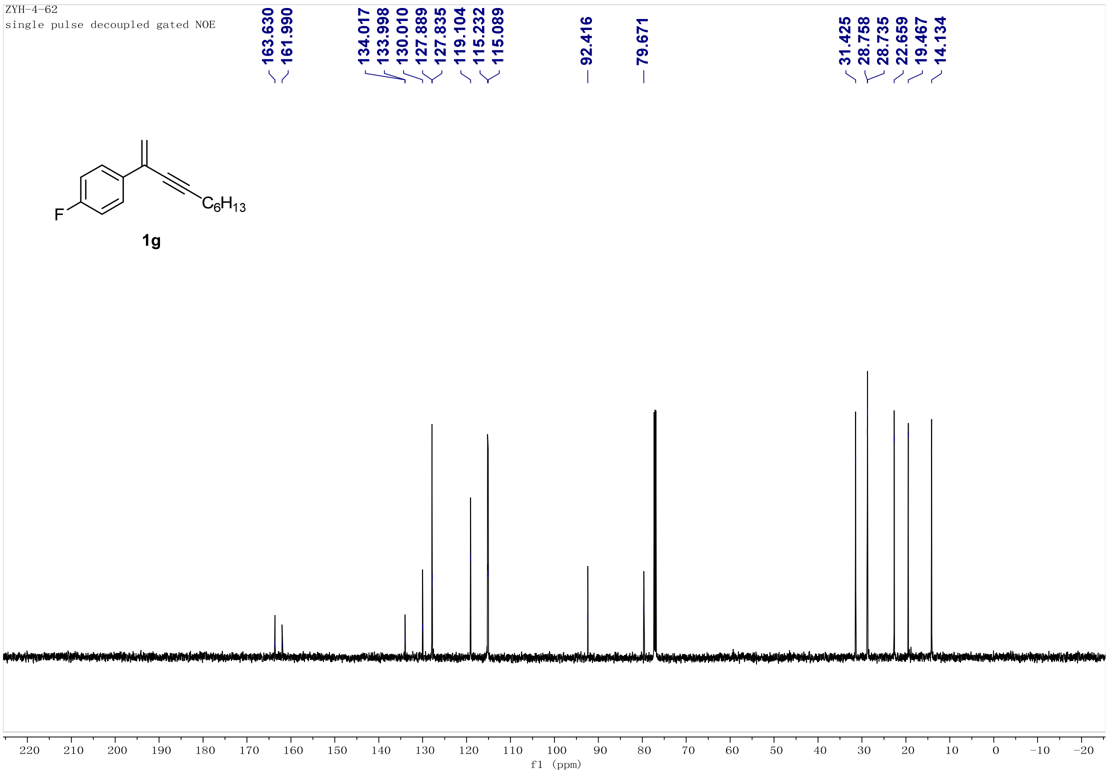

Supplementary Figure 19. ^{13}C NMR spectrum of compound 1g.

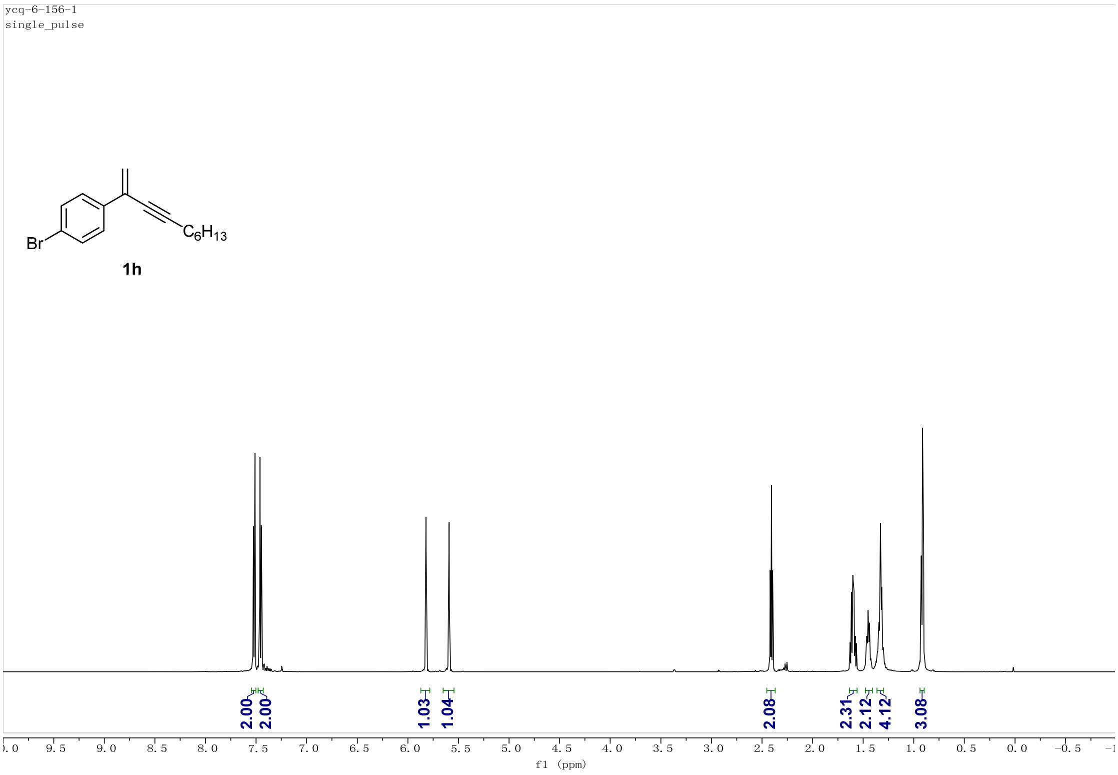

Supplementary Figure 20. ^{1}H NMR spectrum of compound 1h.

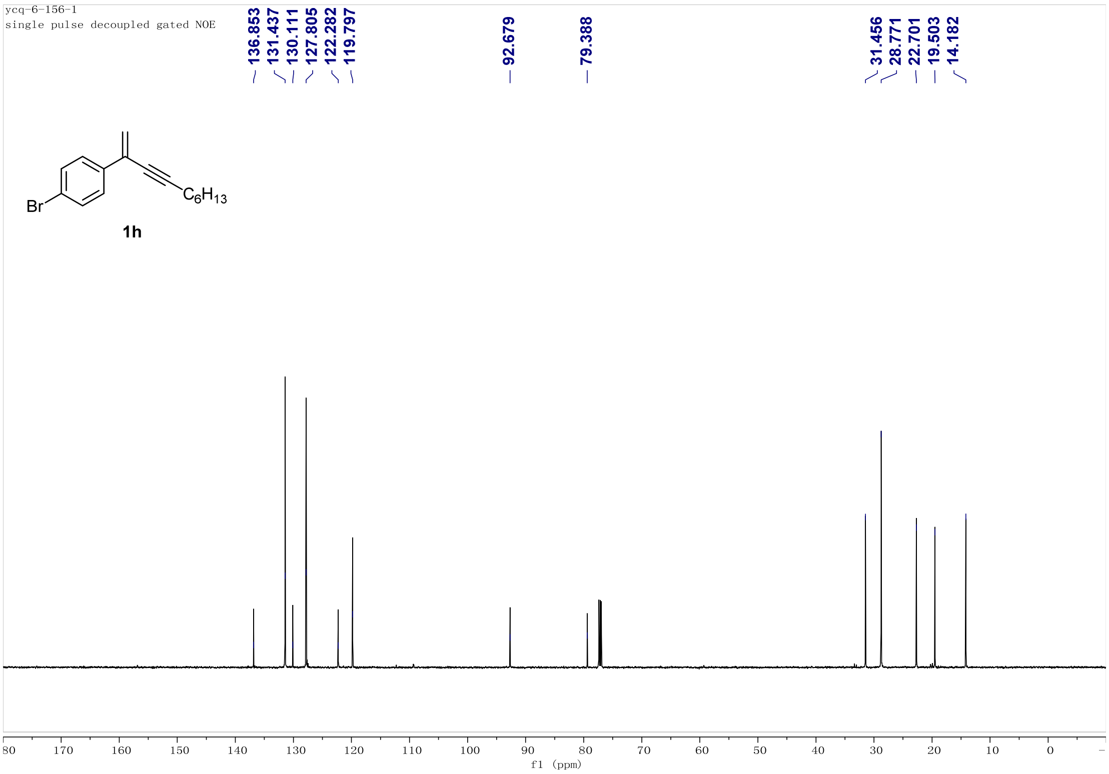

Supplementary Figure 21. ^{13}C NMR spectrum of compound 1h.

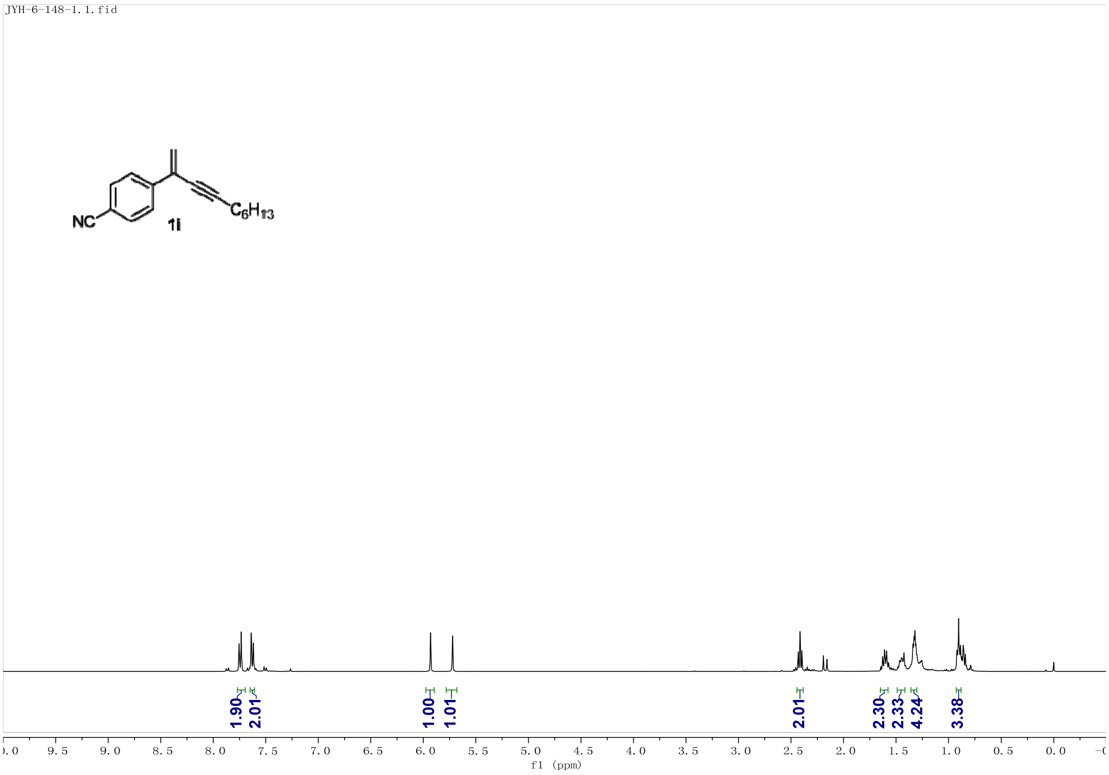

Supplementary Figure 22. ^{1}H **NMR spectrum of compound 1i.**

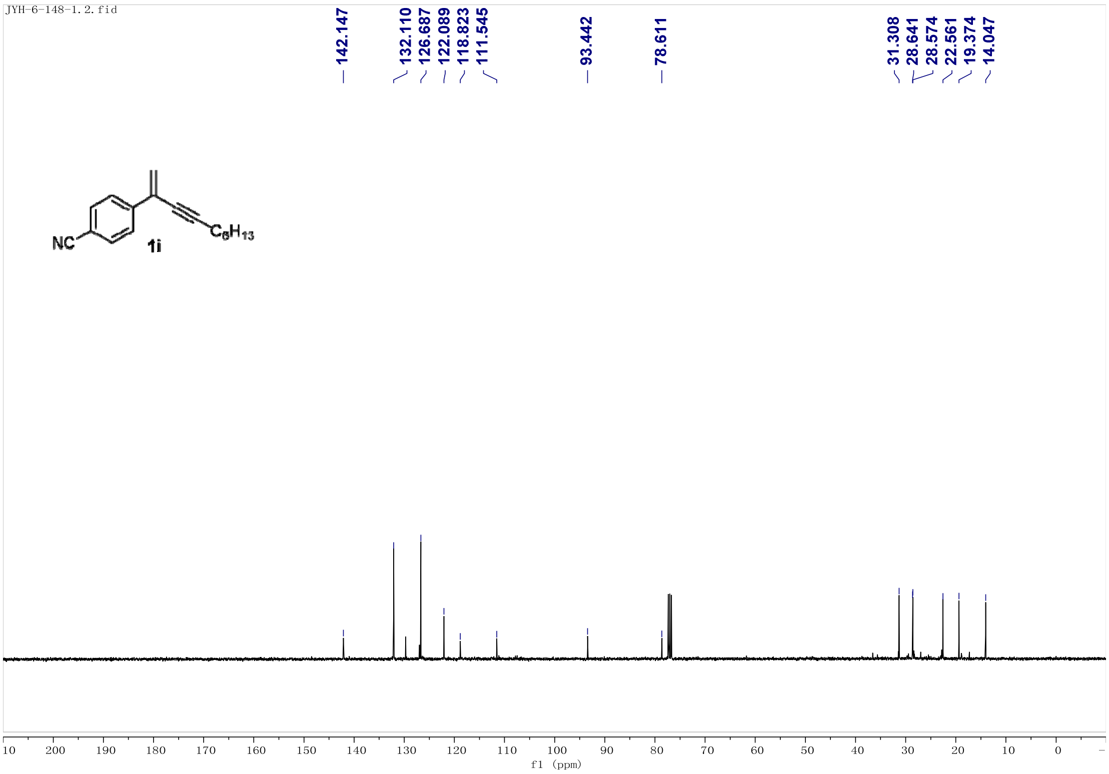

Supplementary Figure 23. ^{13}C **NMR spectrum of compound 1i.**

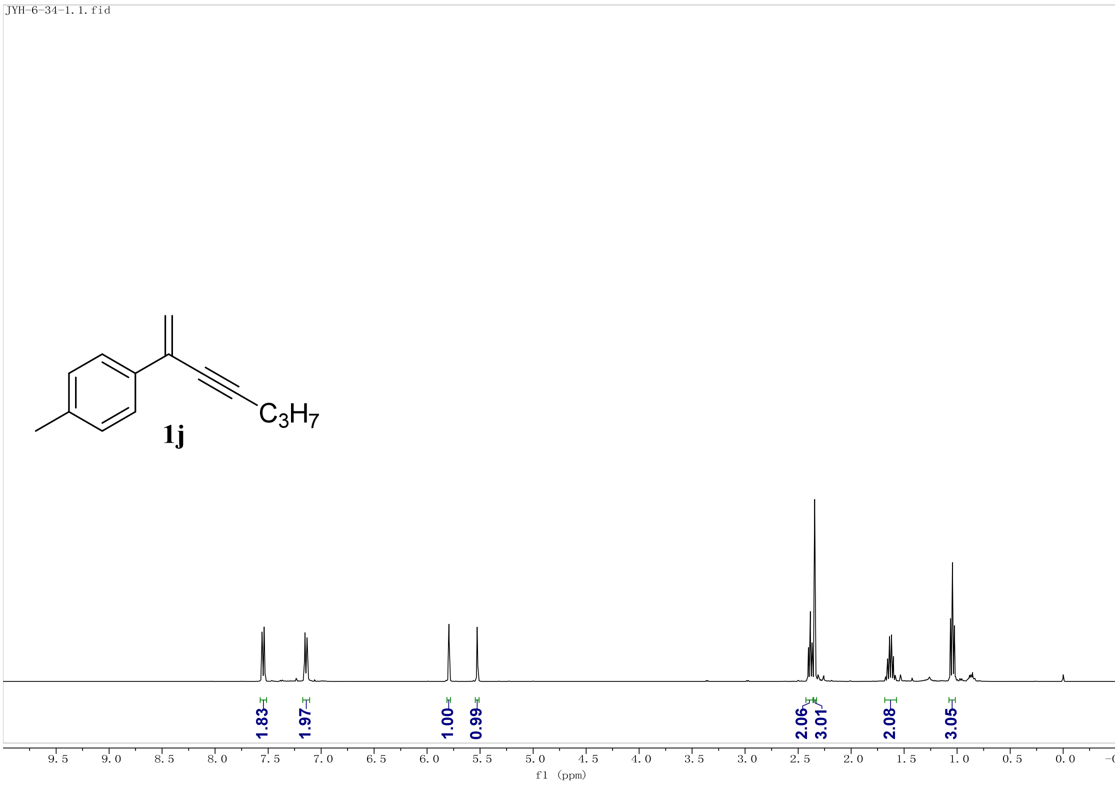

Supplementary Figure 24. ^{1}H NMR spectrum of compound 1j.

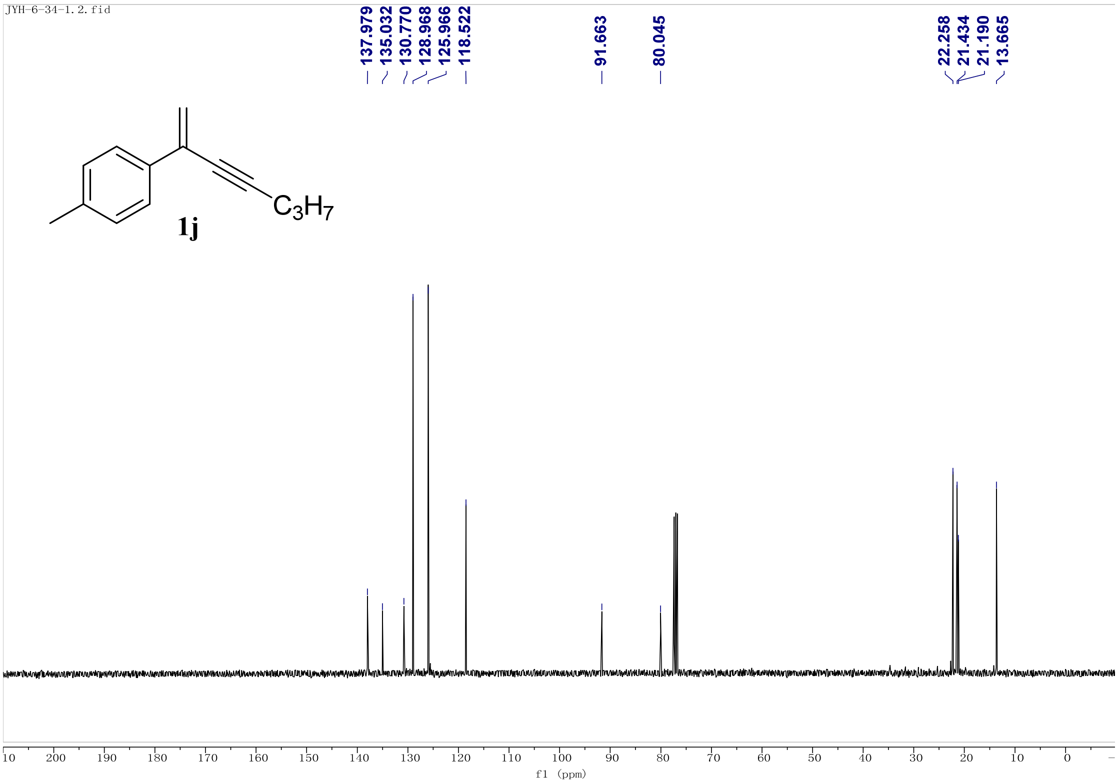

Supplementary Figure 25. **^{13}C NMR spectrum of compound 1j.**

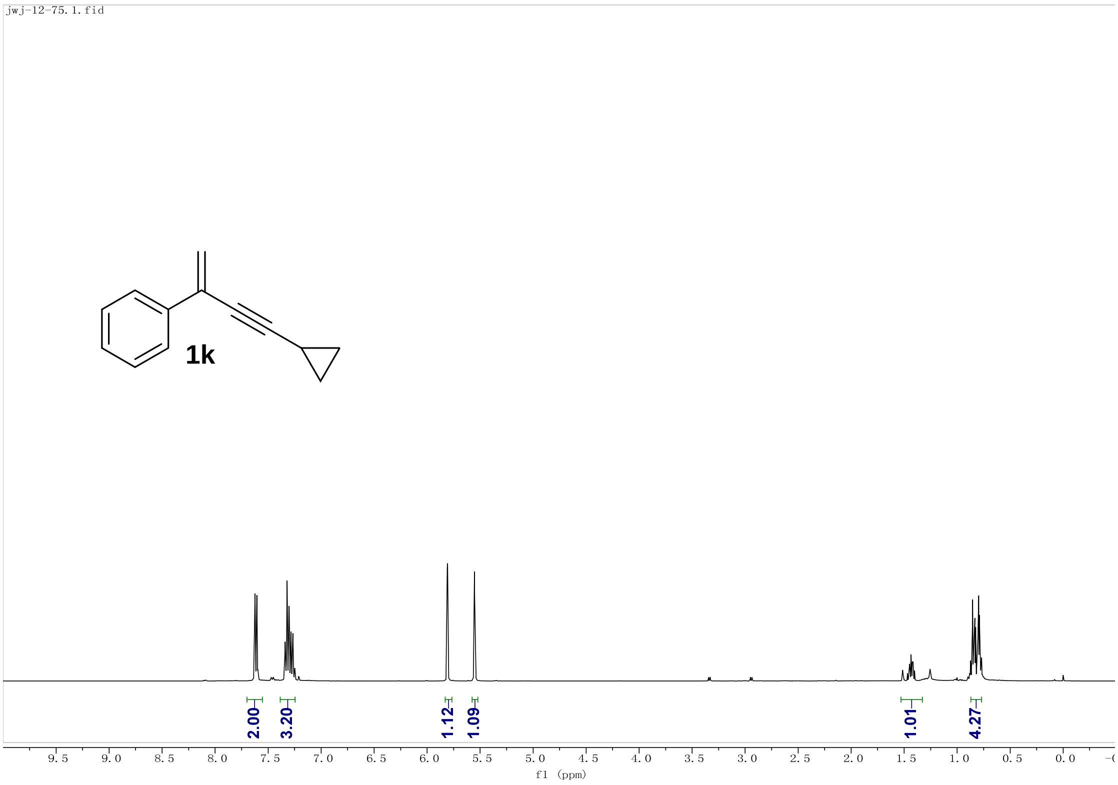

Supplementary Figure 26. **^{1}H NMR spectrum of compound 1k.**

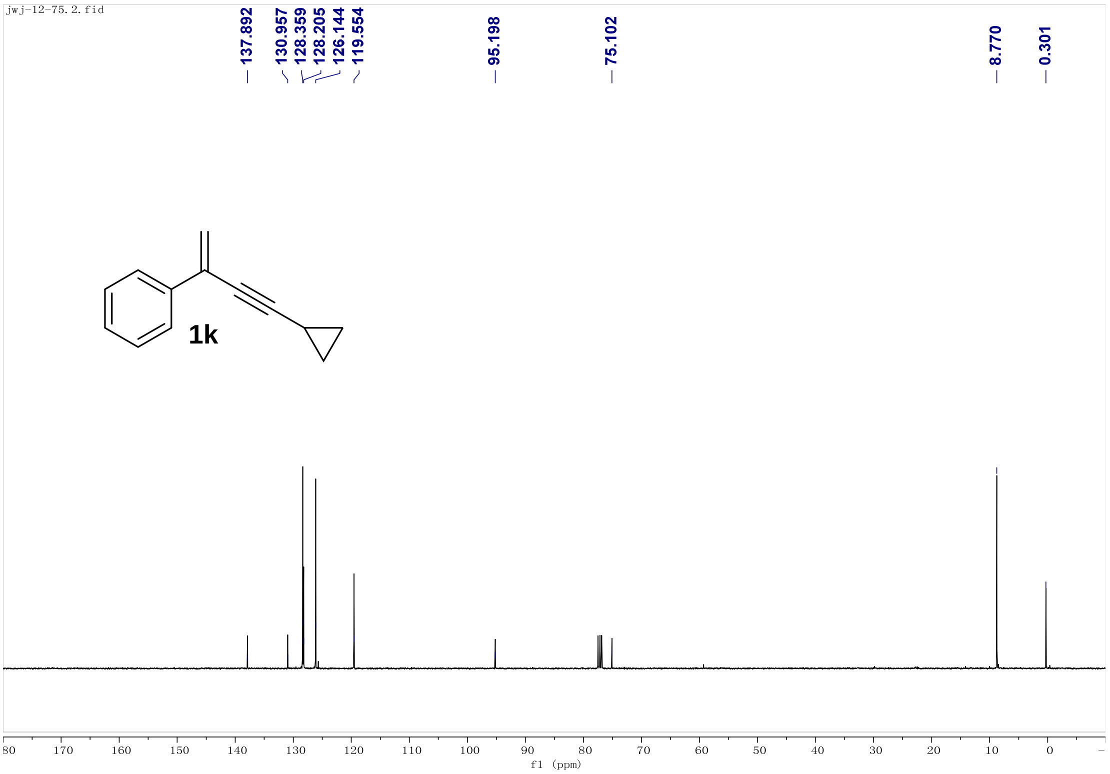

Supplementary Figure 27. **^{13}C NMR spectrum of compound 1k.**

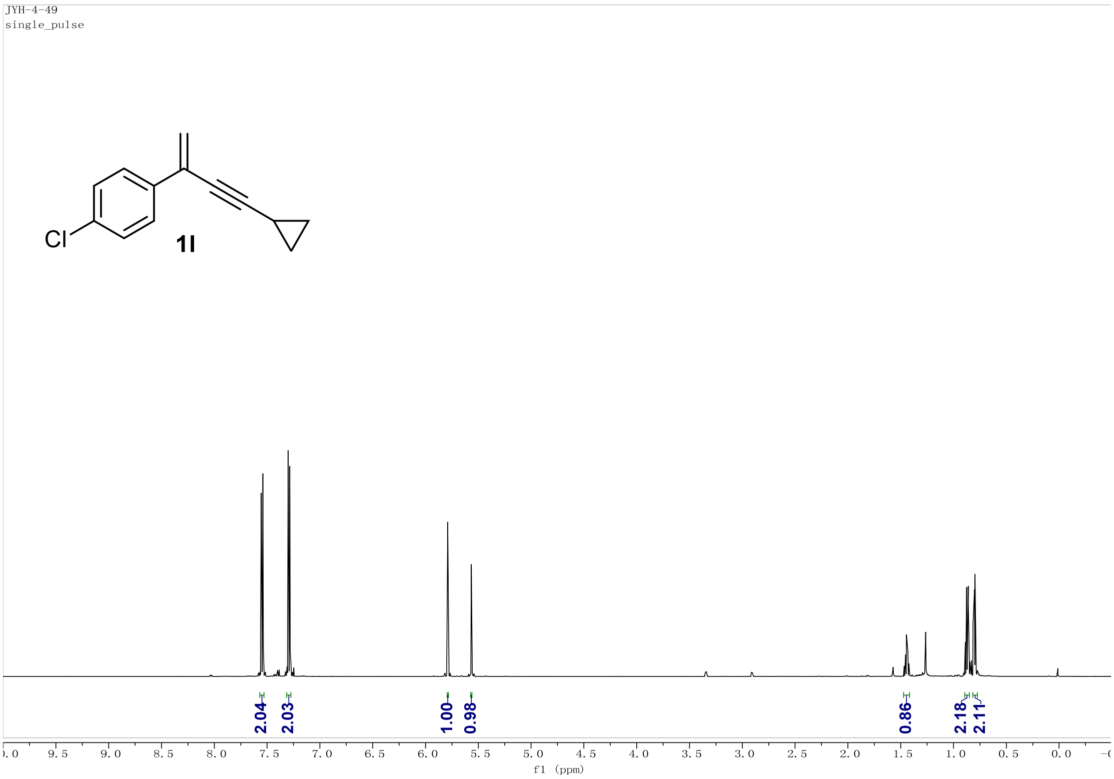

Supplementary Figure 28. ^{1}H NMR spectrum of compound 1l.

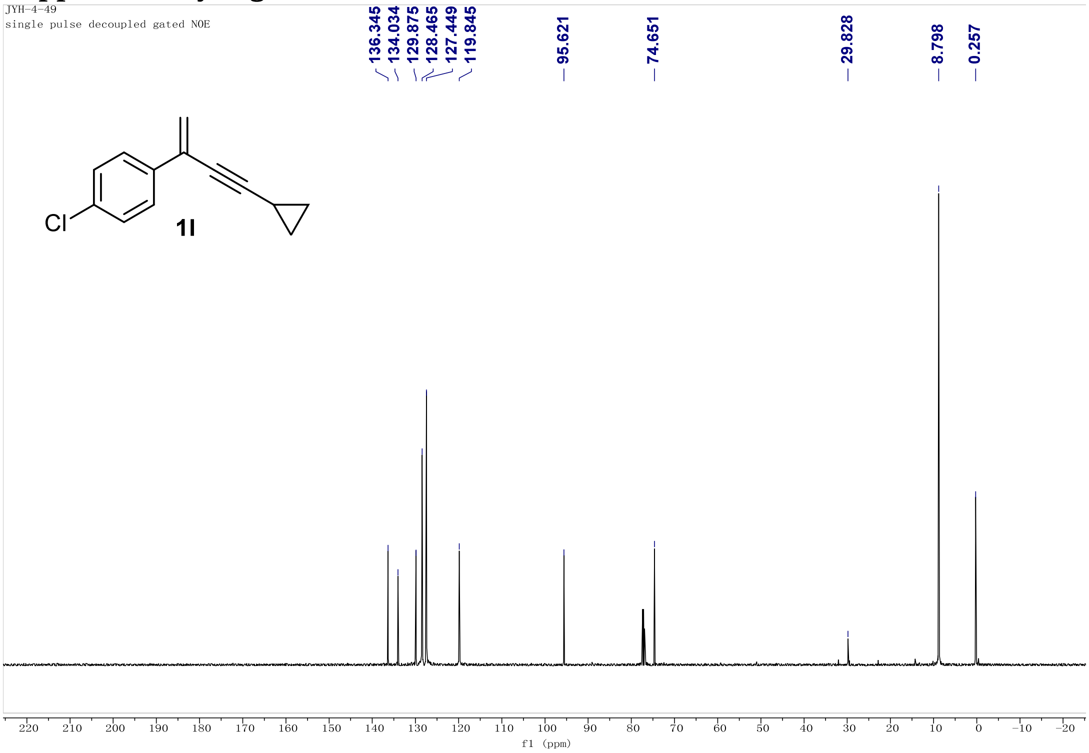

Supplementary Figure 29. ^{13}C NMR spectrum of compound 1l.

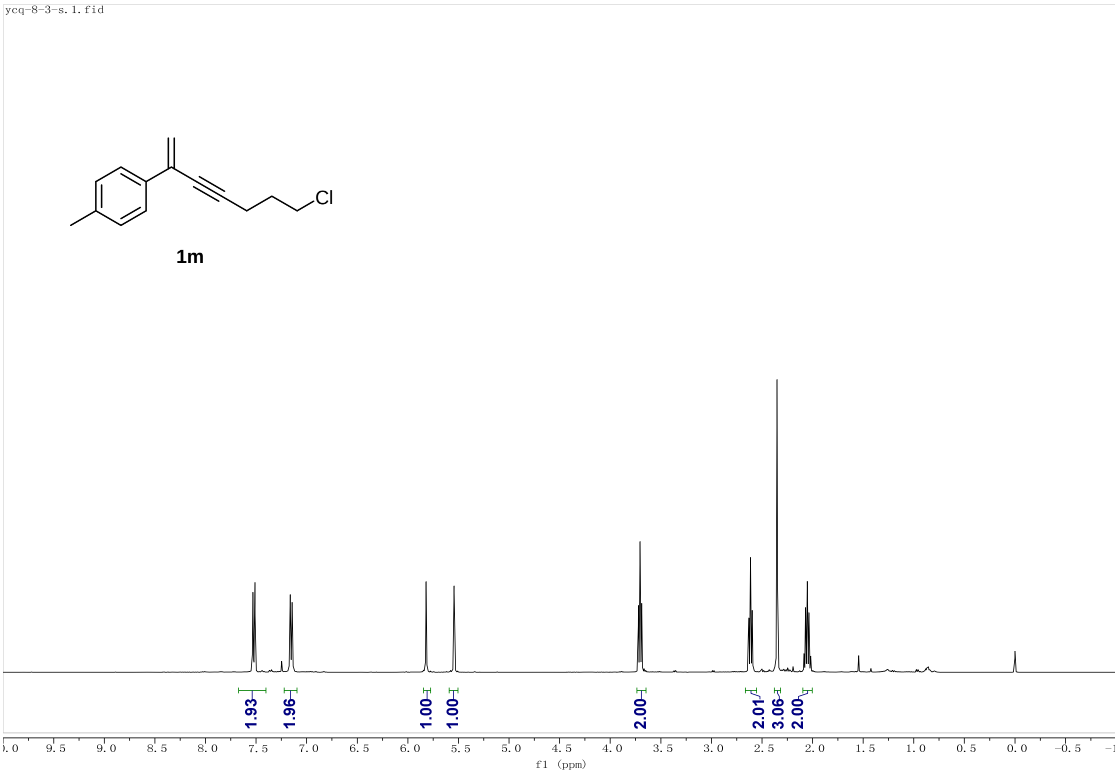

Supplementary Figure 30. ^{1}H NMR spectrum of compound 1m.

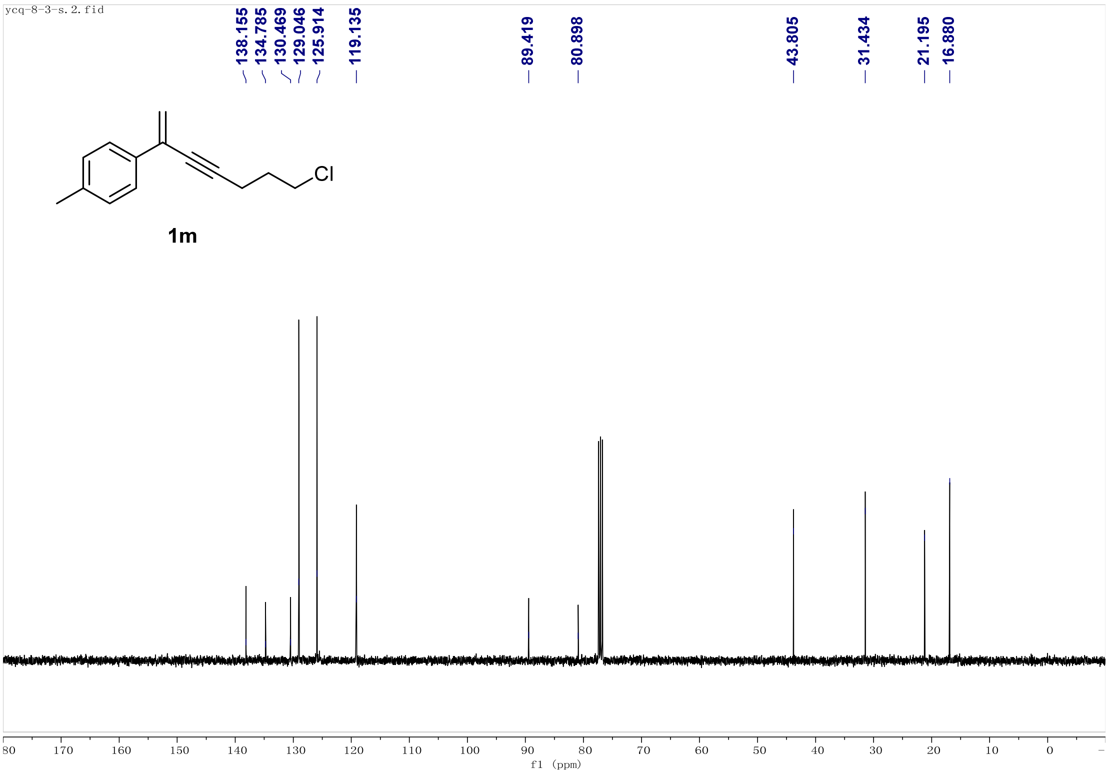

Supplementary Figure 31. ^{13}C NMR spectrum of compound 1m.

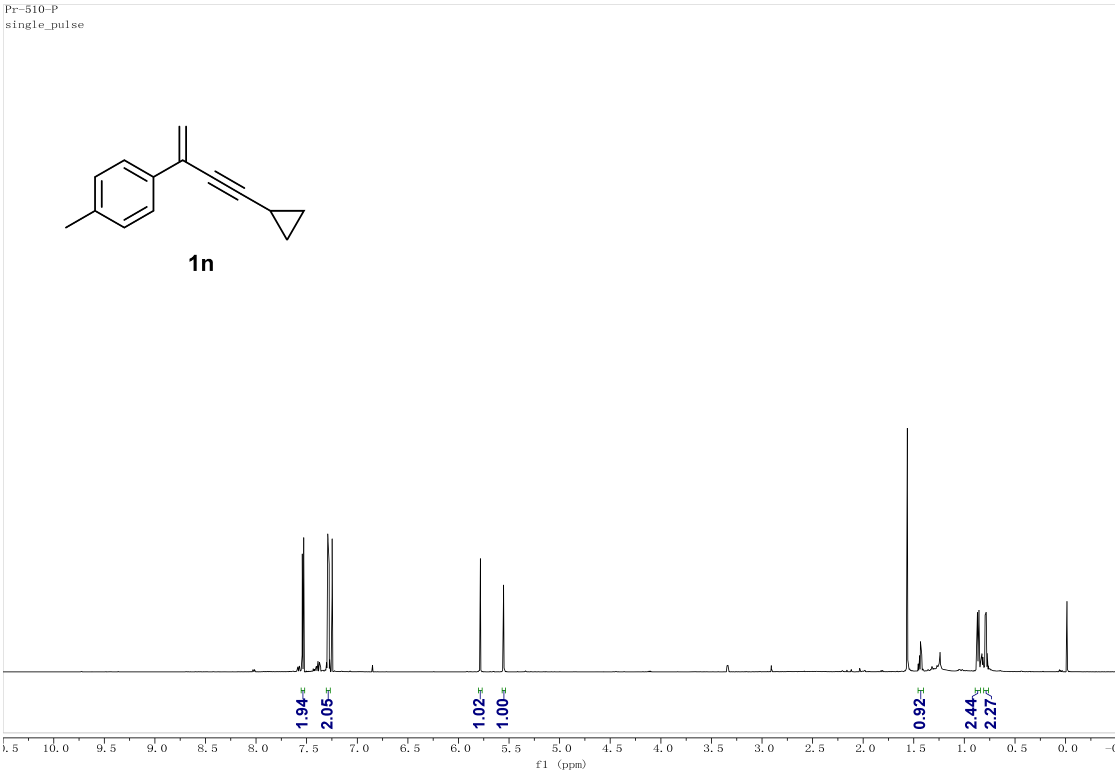

Supplementary Figure 32. ^{1}H **NMR spectrum of compound 1n.**

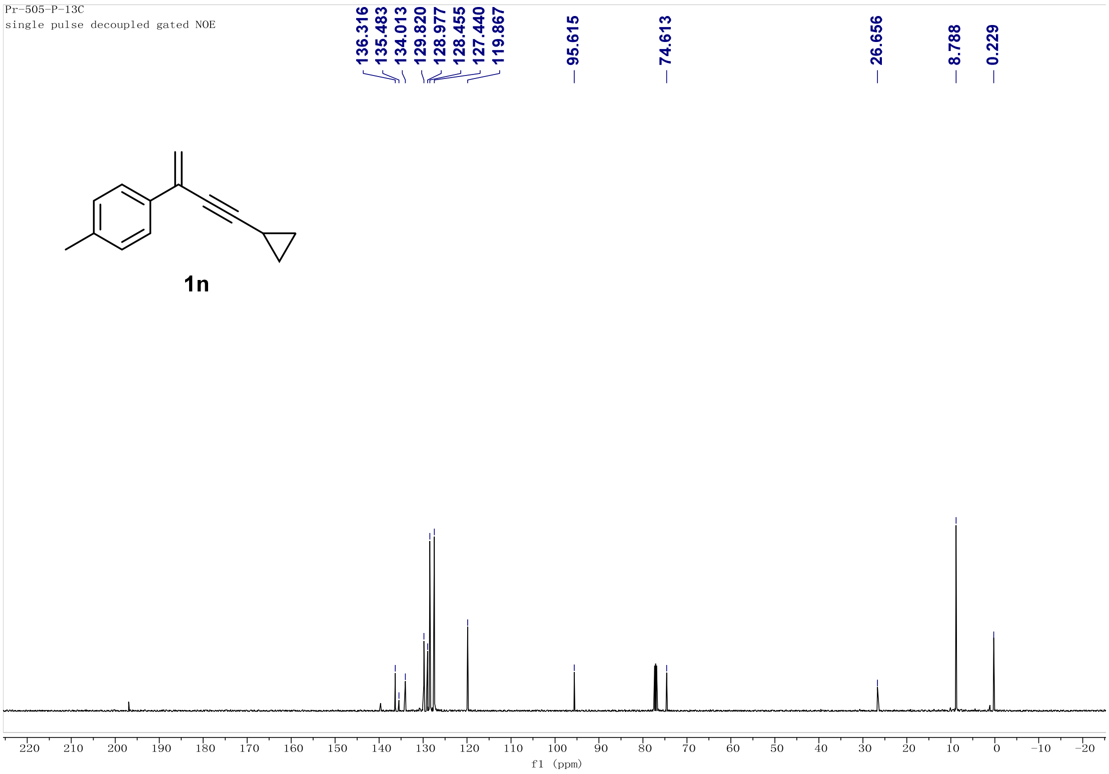

Supplementary Figure 33. ^{13}C **NMR spectrum of compound 1n.**

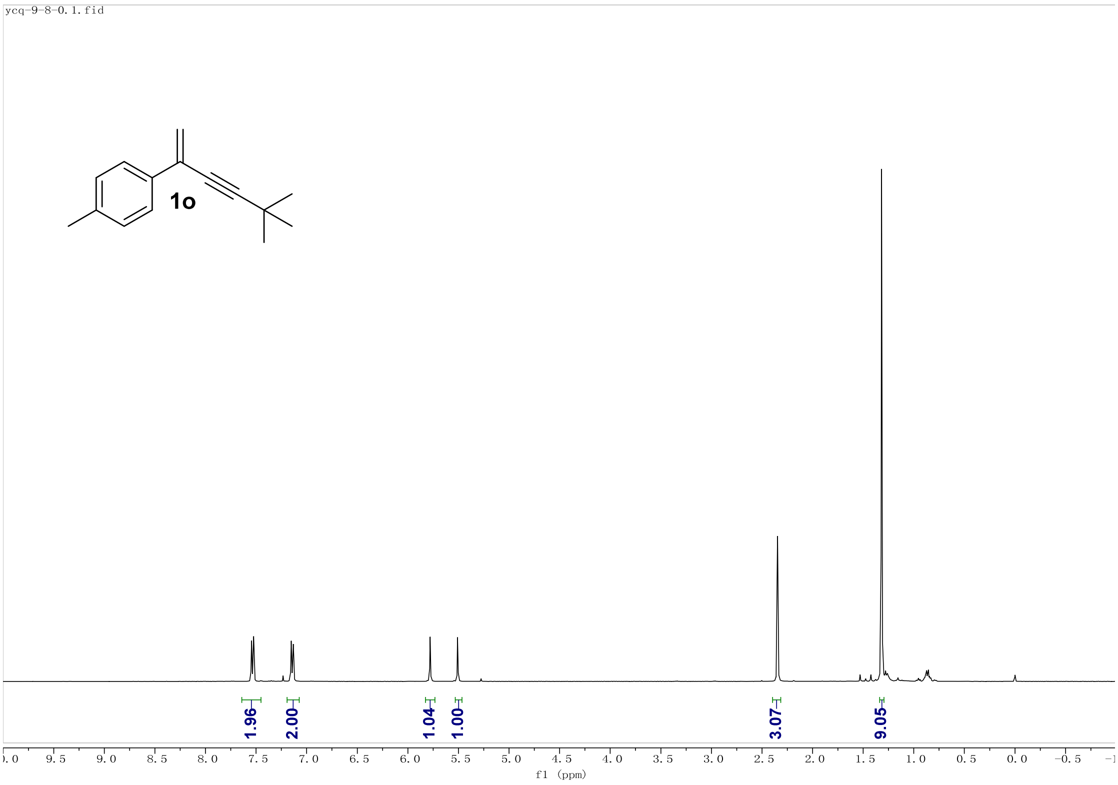

Supplementary Figure 34. ^{1}H **NMR spectrum of compound 1o.**

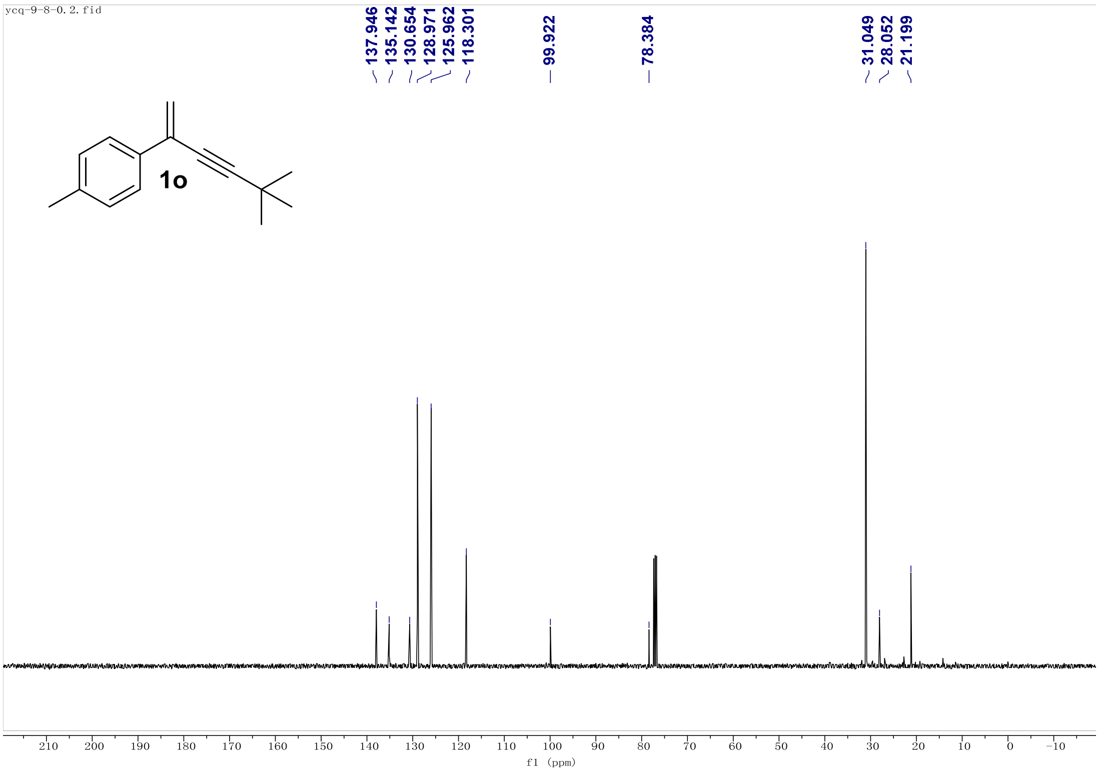

Supplementary Figure 35. ^{13}C **NMR spectrum of compound 1o.**

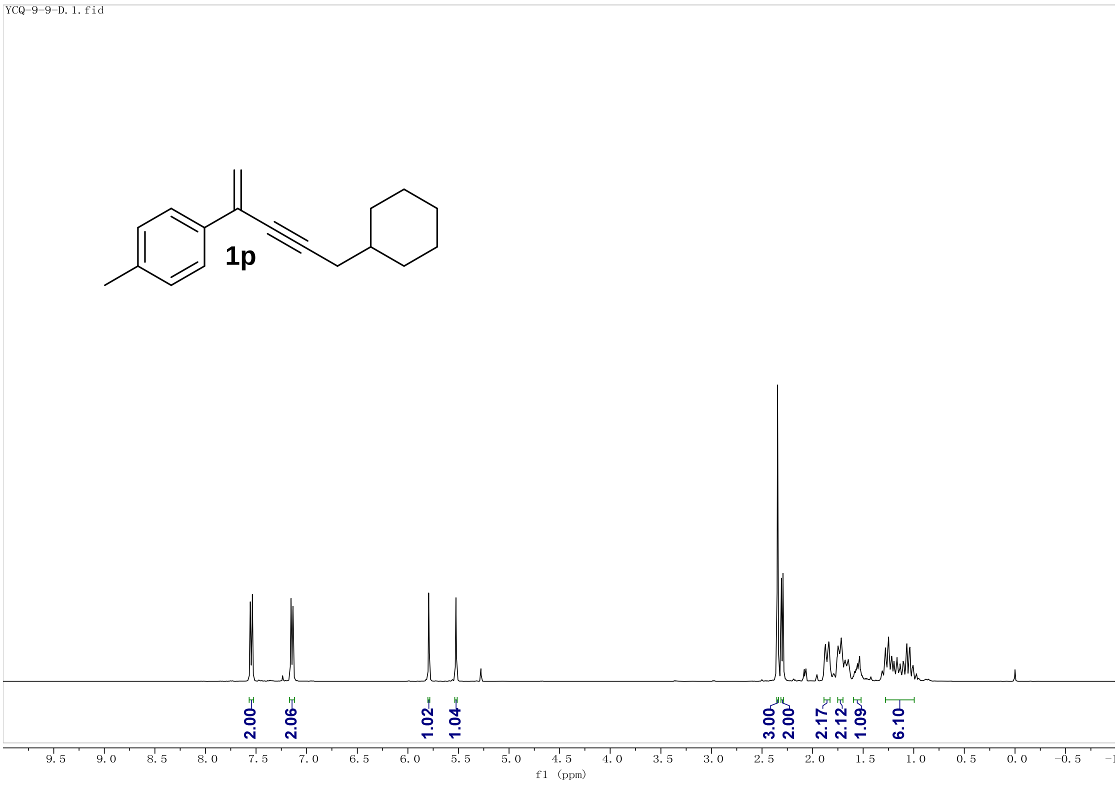

Supplementary Figure 36. ^{1}H NMR spectrum of compound 1p.

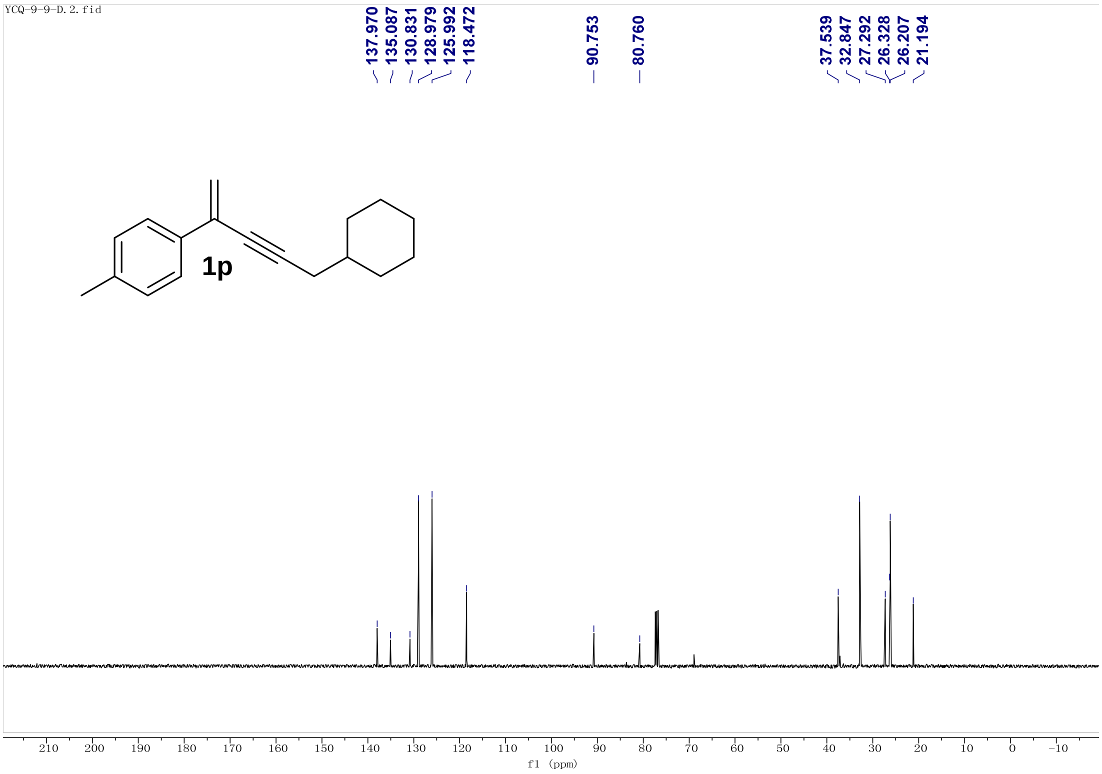

Supplementary Figure 37. ^{13}C NMR spectrum of compound 1p.

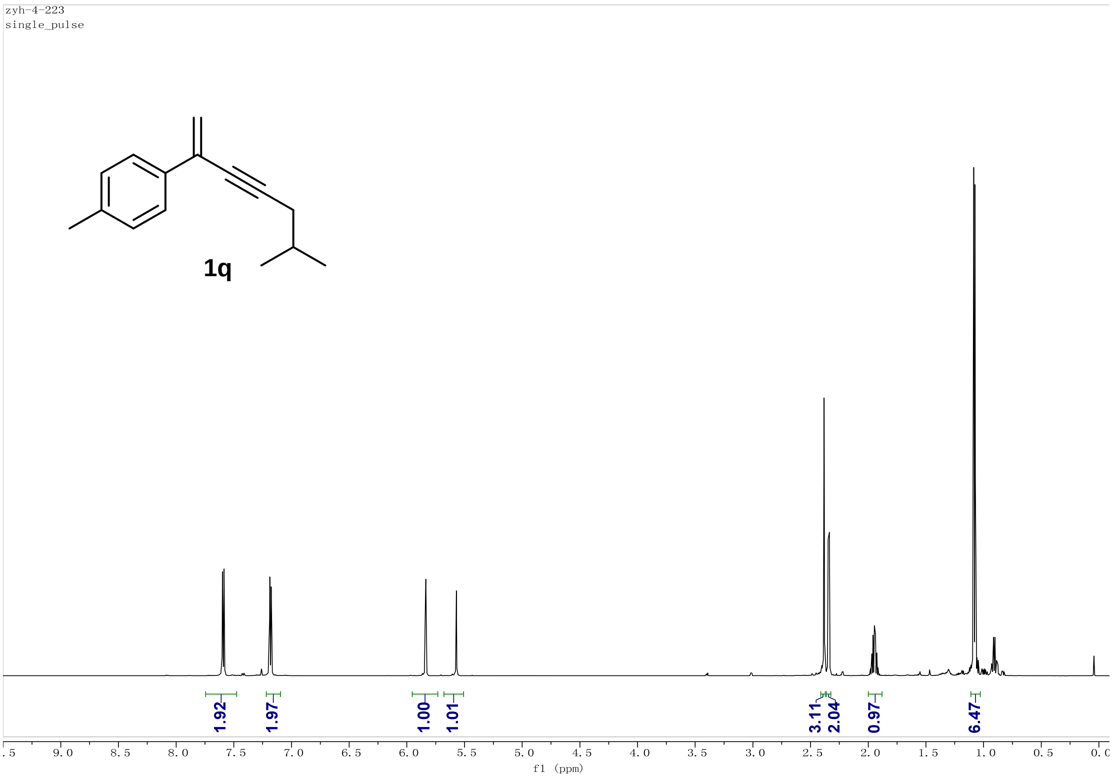

Supplementary Figure 38. ^1H NMR spectrum of compound **1q**.

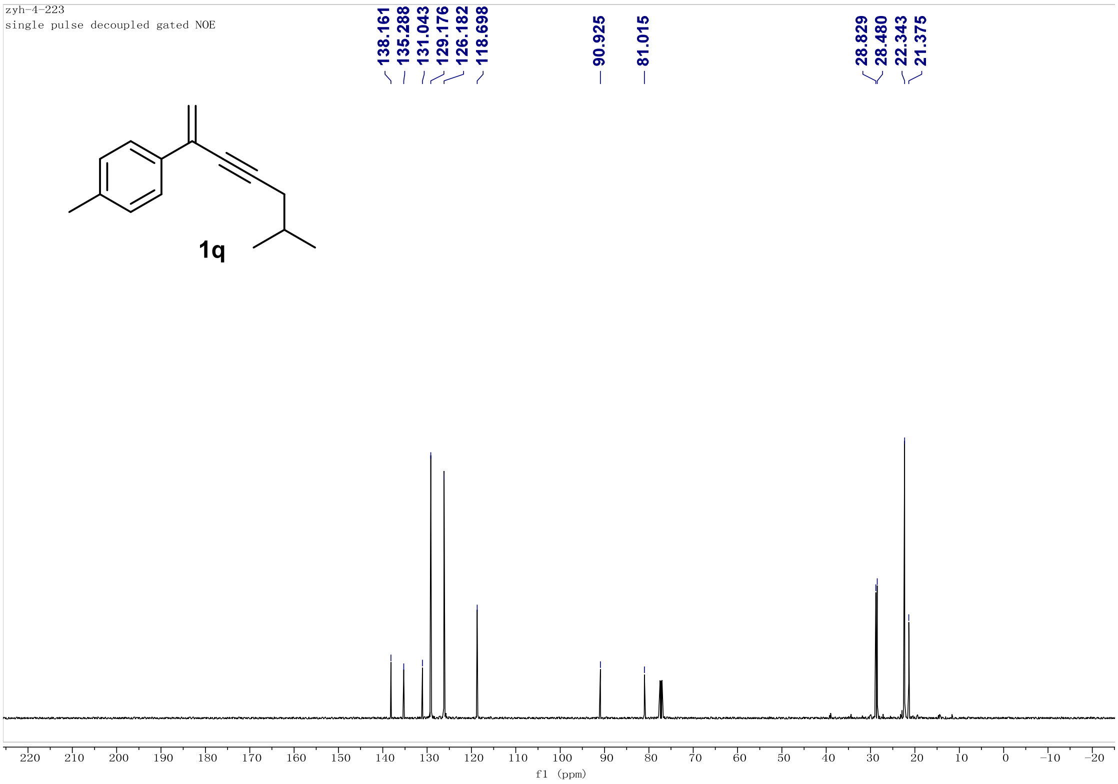

Supplementary Figure 39. ^{13}C NMR spectrum of compound **1q**.

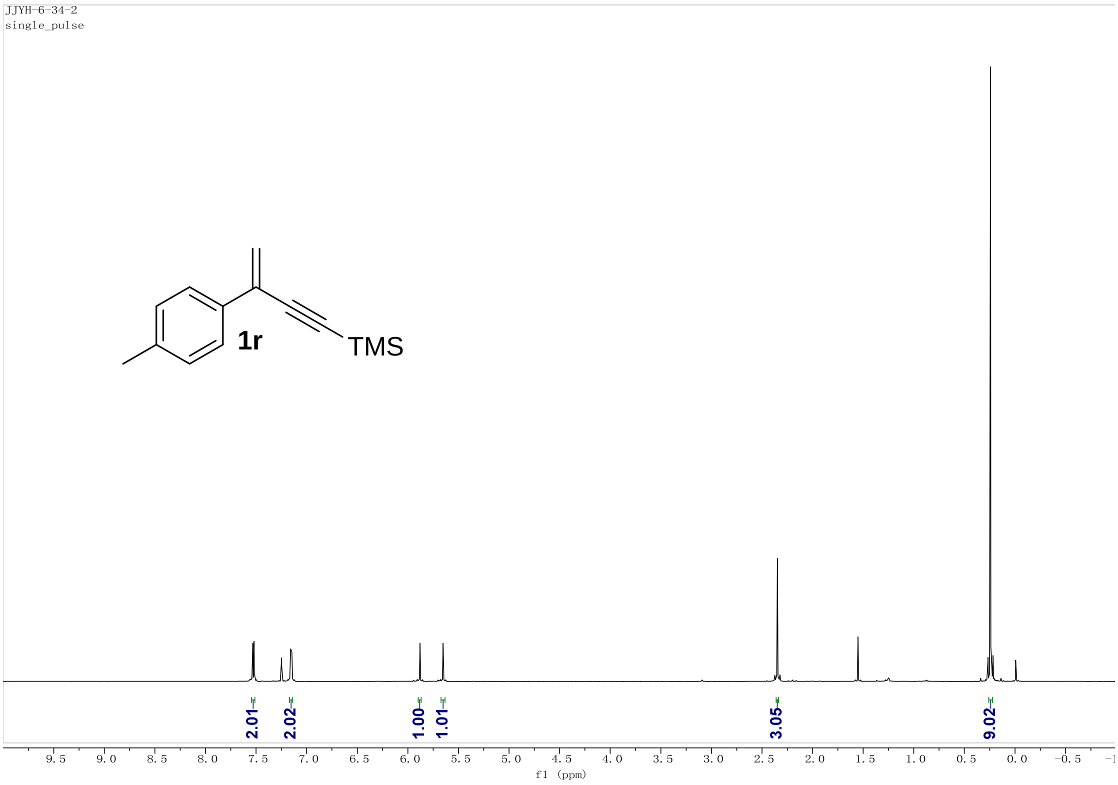

Supplementary Figure 40. ^{1}H NMR spectrum of compound 1r.

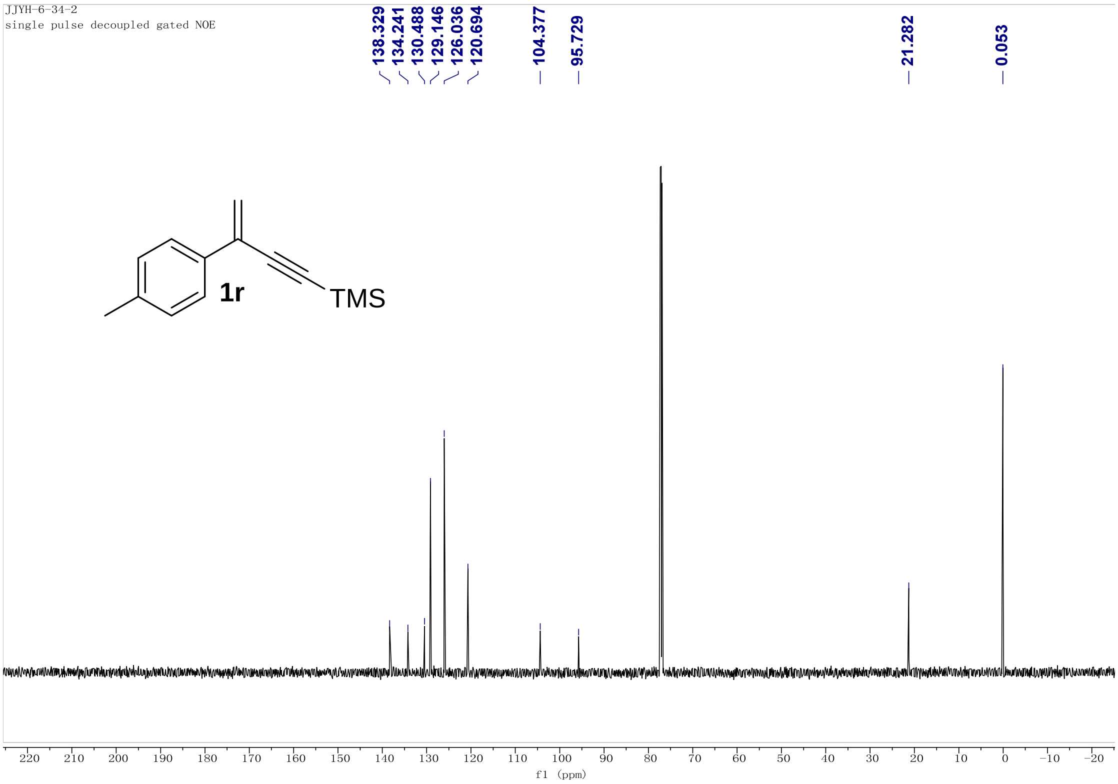

Supplementary Figure 41. ^{13}C NMR spectrum of compound 1r.

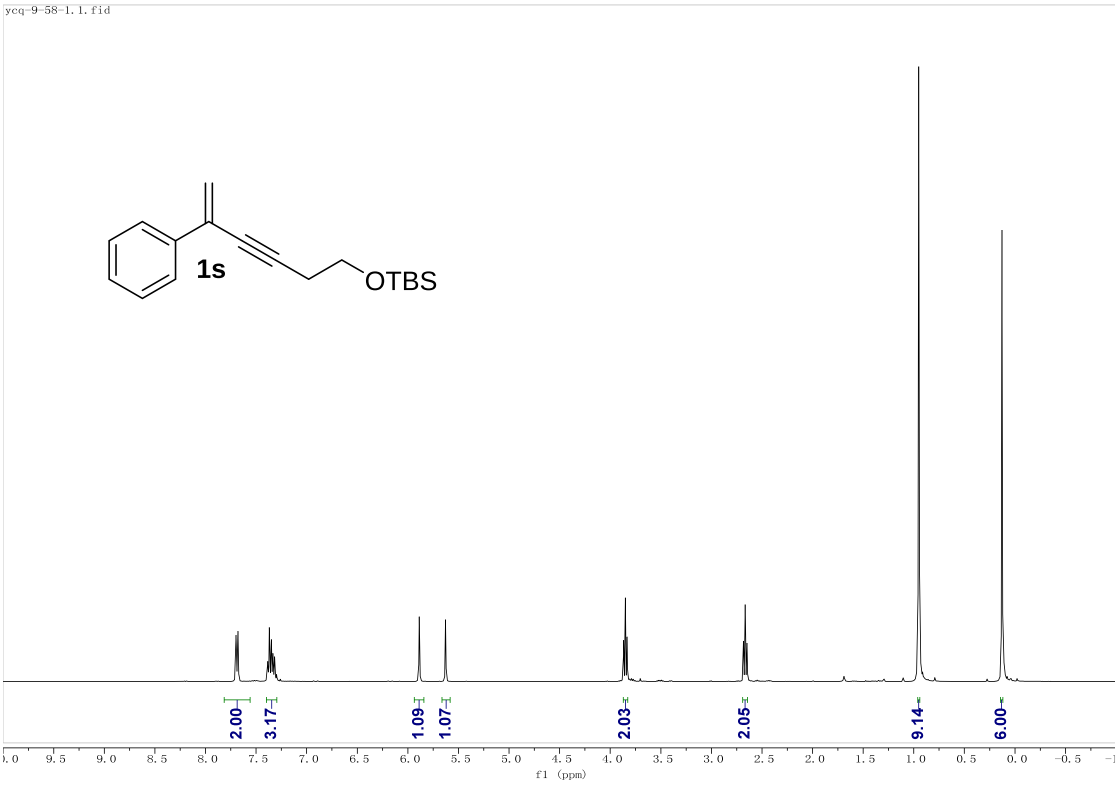

Supplementary Figure 42. ^{1}H **NMR spectrum of compound 1s.**

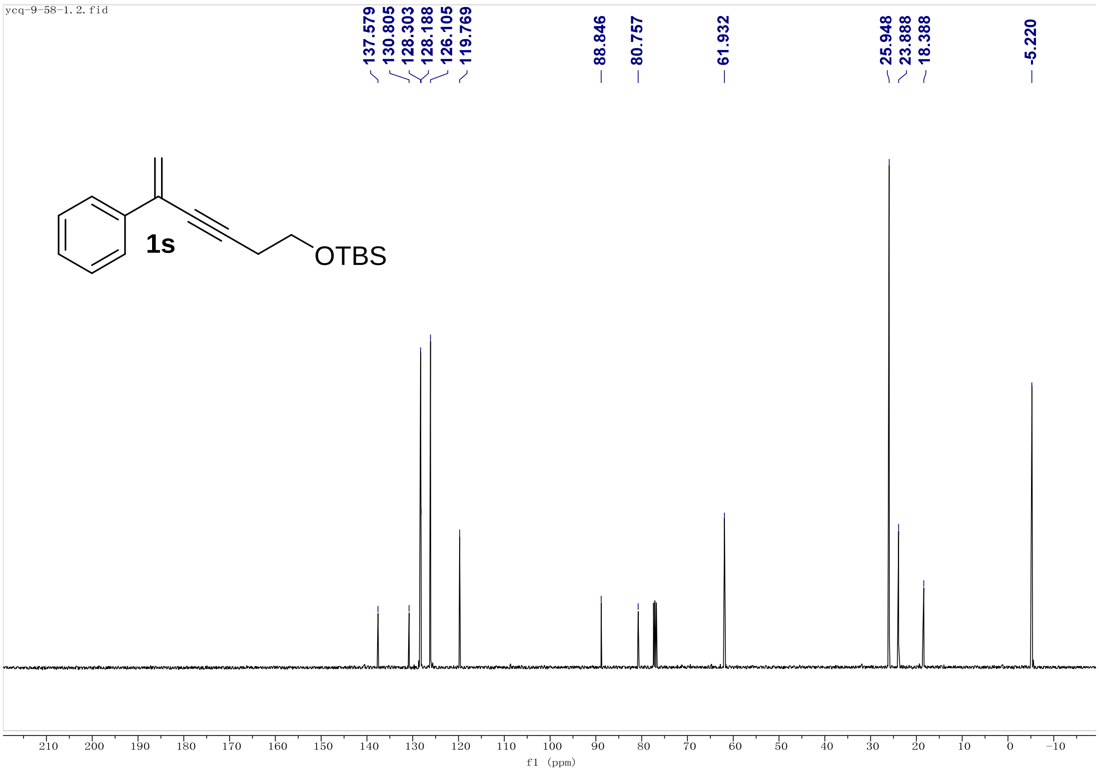

Supplementary Figure 43. ^{13}C **NMR spectrum of compound 1s.**

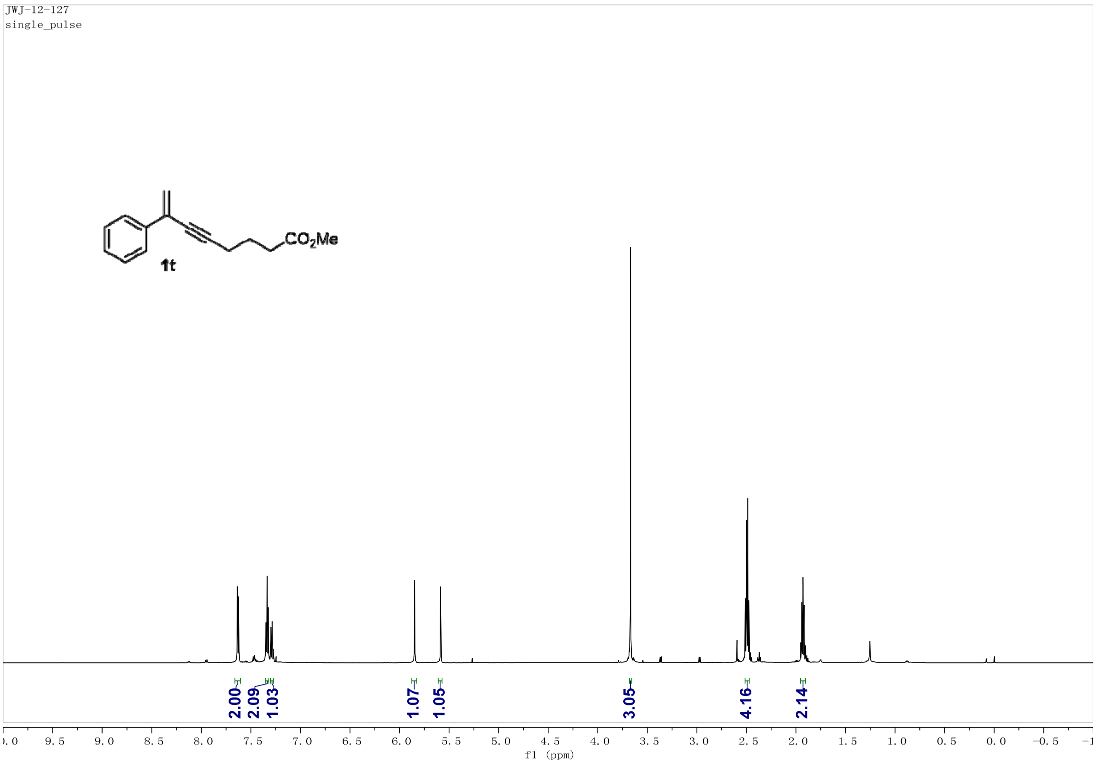

Supplementary Figure 44. ^{1}H NMR spectrum of compound 1t.

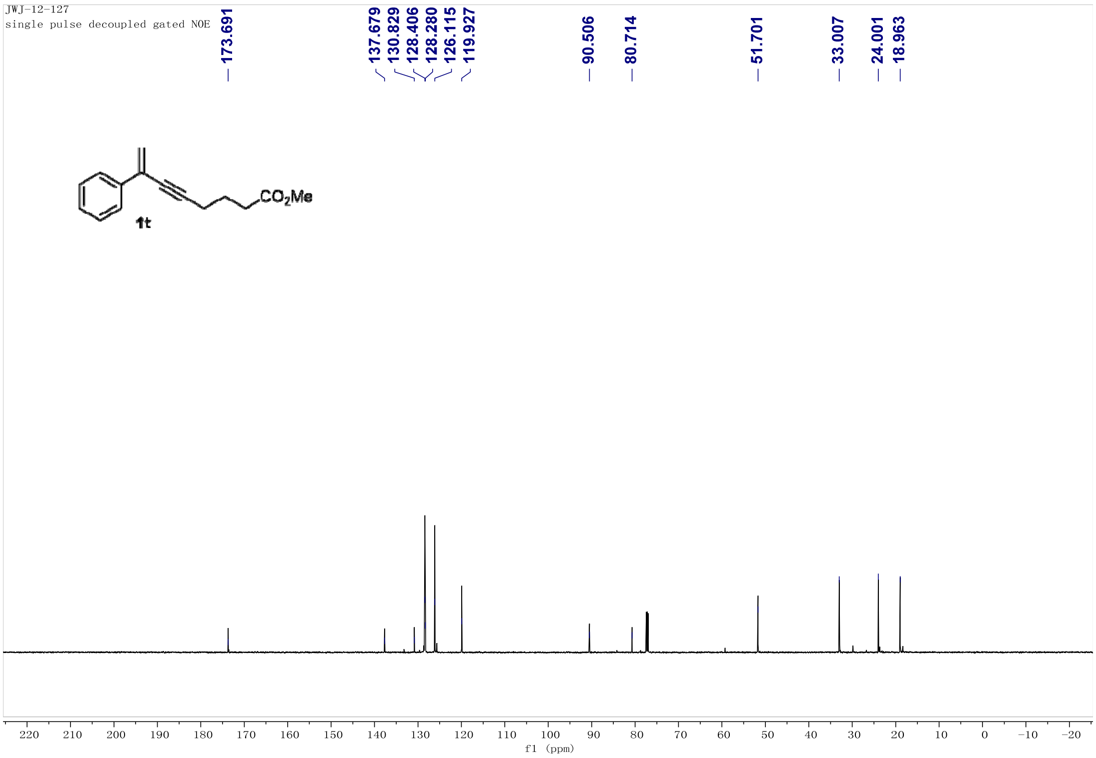

Supplementary Figure 45. ^{13}C NMR spectrum of compound 1t.

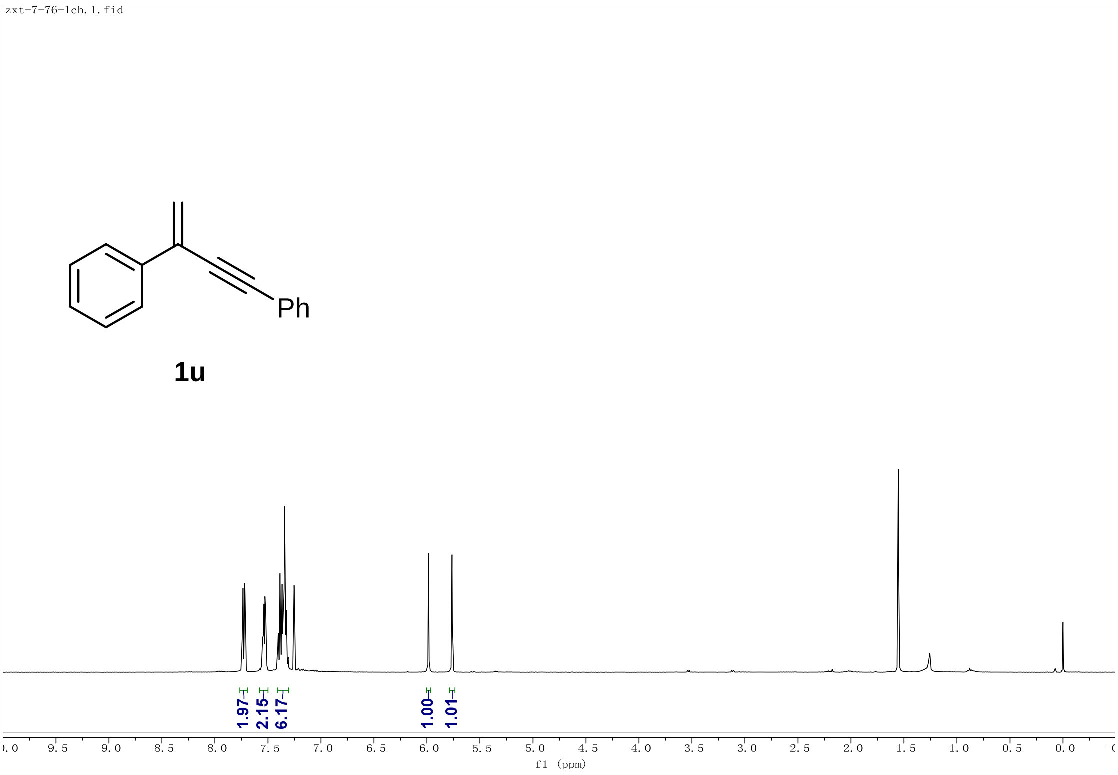

Supplementary Figure 46. ^{1}H NMR spectrum of compound 1u.

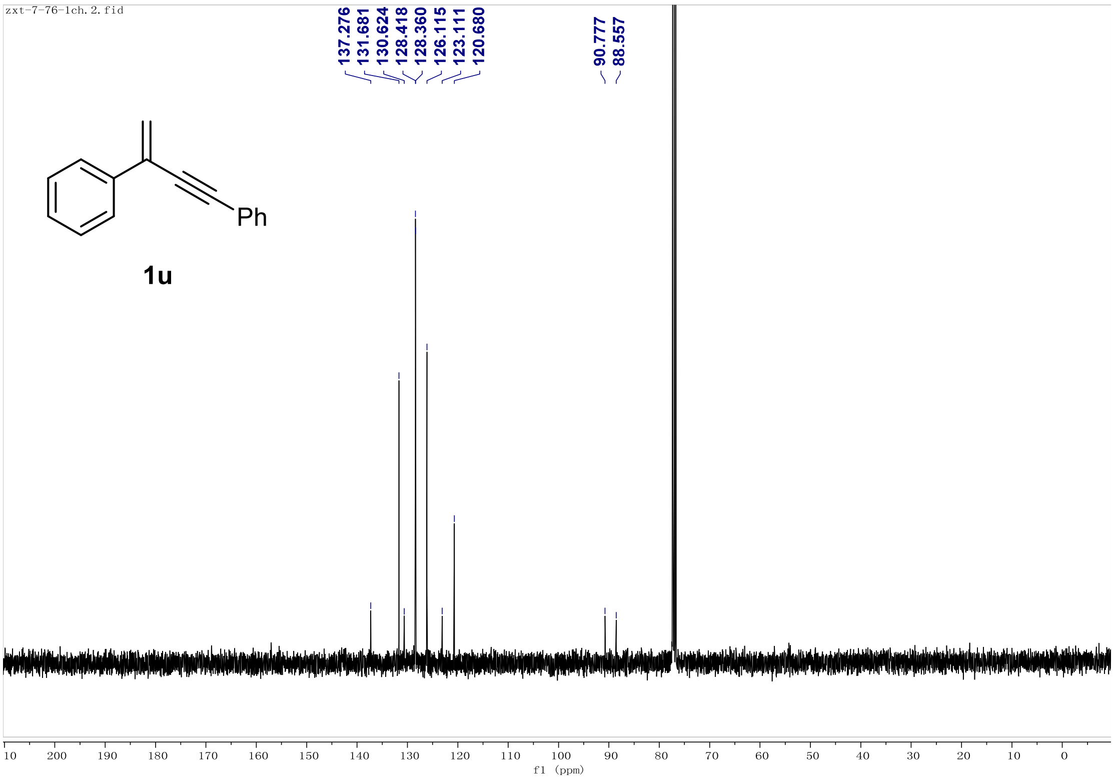

Supplementary Figure 47. ^{13}C NMR spectrum of compound 1u.

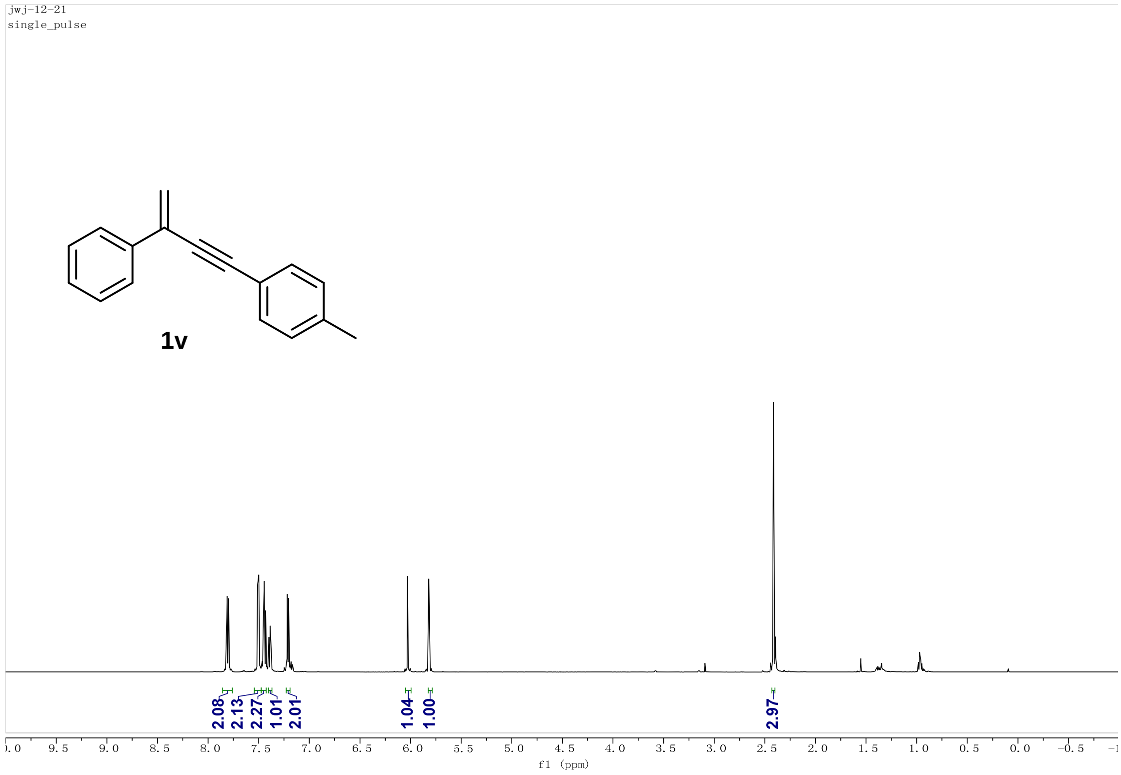

Supplementary Figure 48. ^{1}H NMR spectrum of compound 1v.

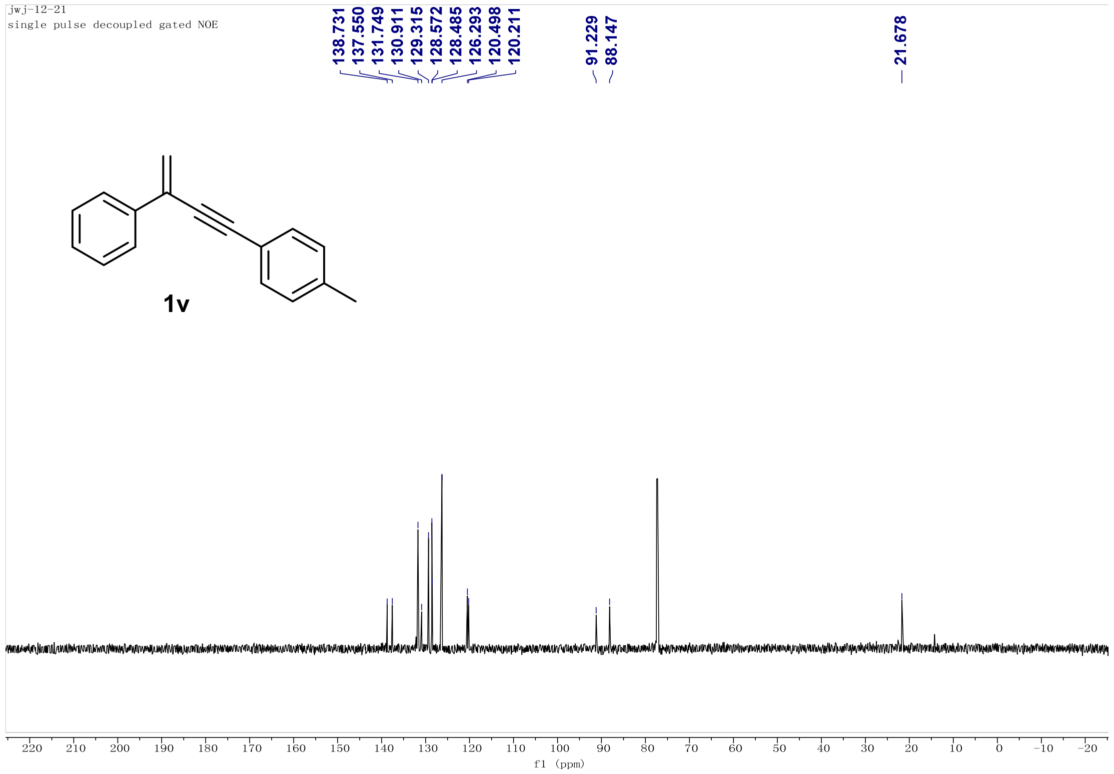

Supplementary Figure 49. ^{13}C NMR spectrum of compound 1v.

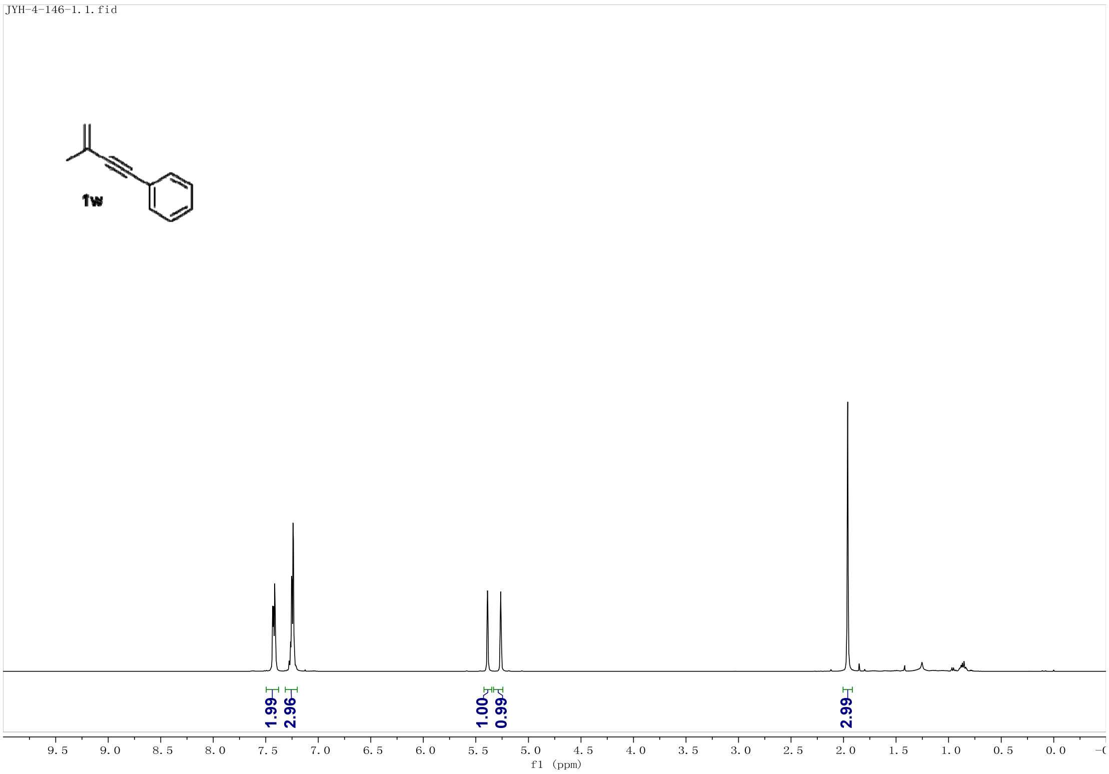

Supplementary Figure 50. ^{1}H **NMR spectrum of compound 1w.**

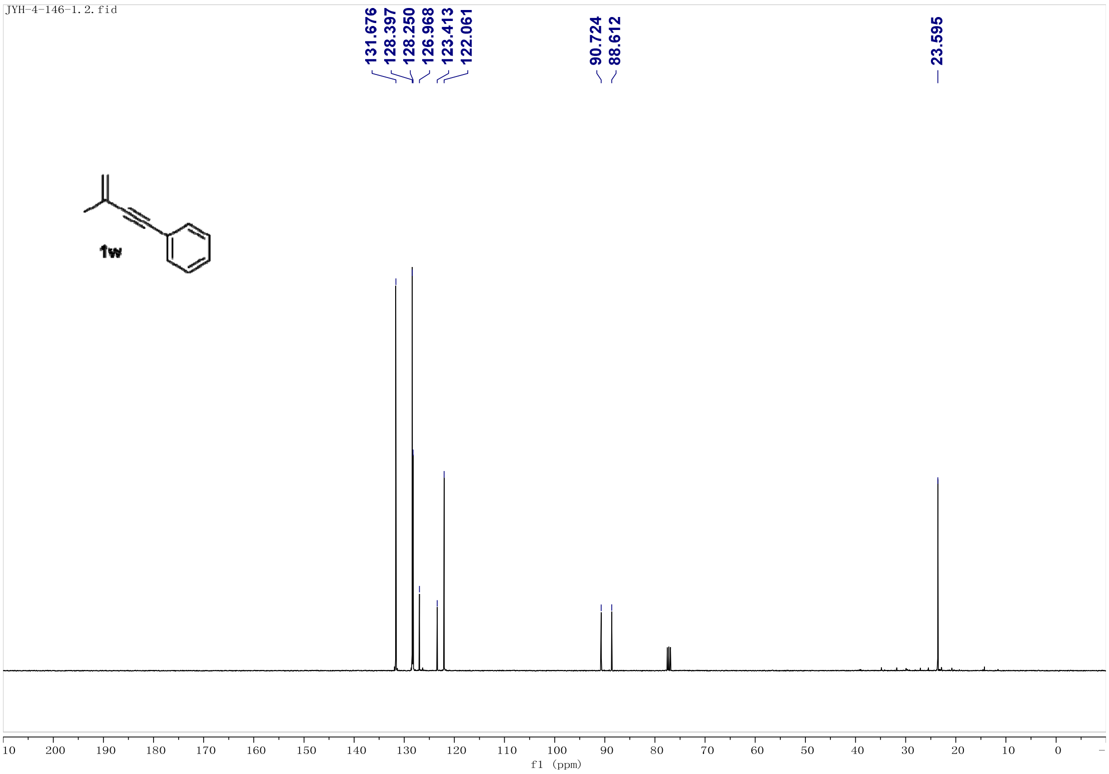

Supplementary Figure 51. ^{13}C **NMR spectrum of compound 1w.**

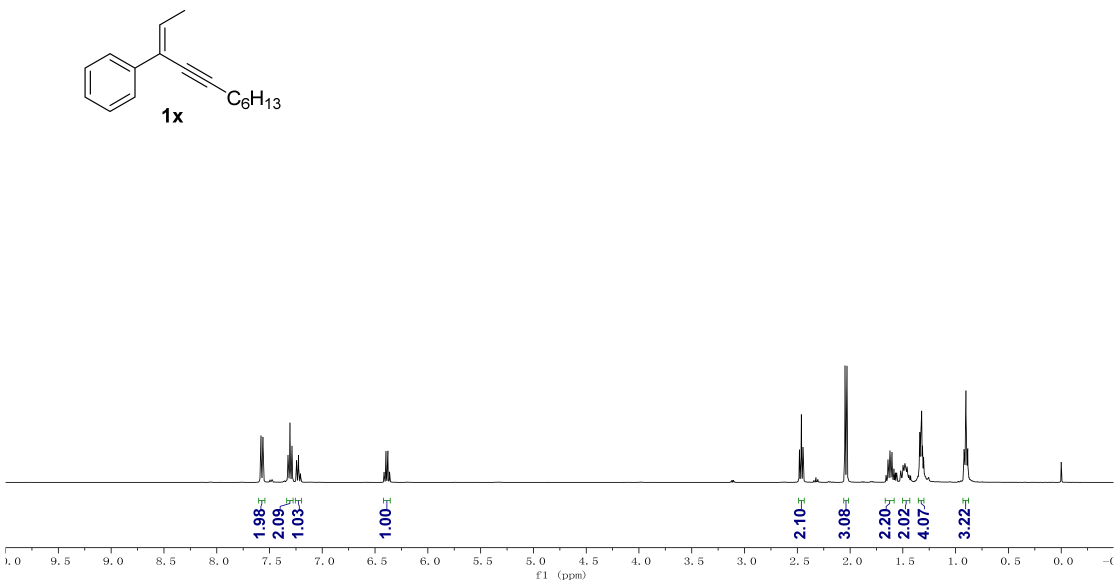

Supplementary Figure 52. ^{1}H NMR spectrum of compound 1x.

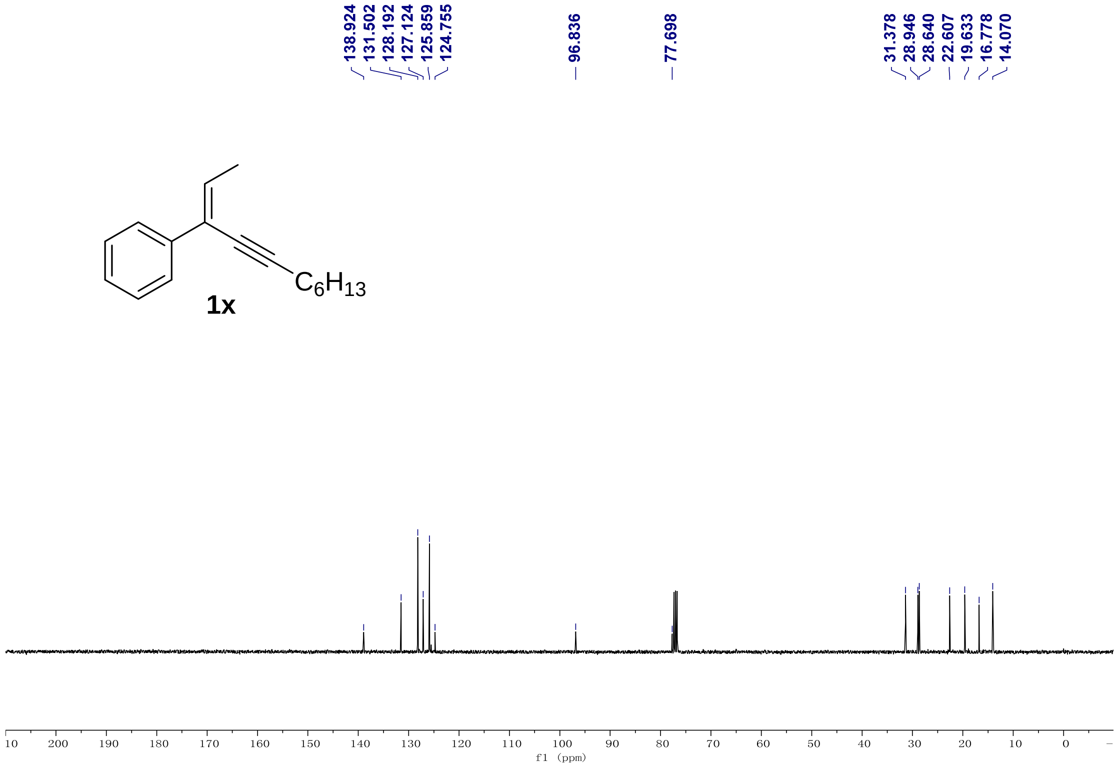

Supplementary Figure 53. ^{13}C NMR spectrum of compound 1x.

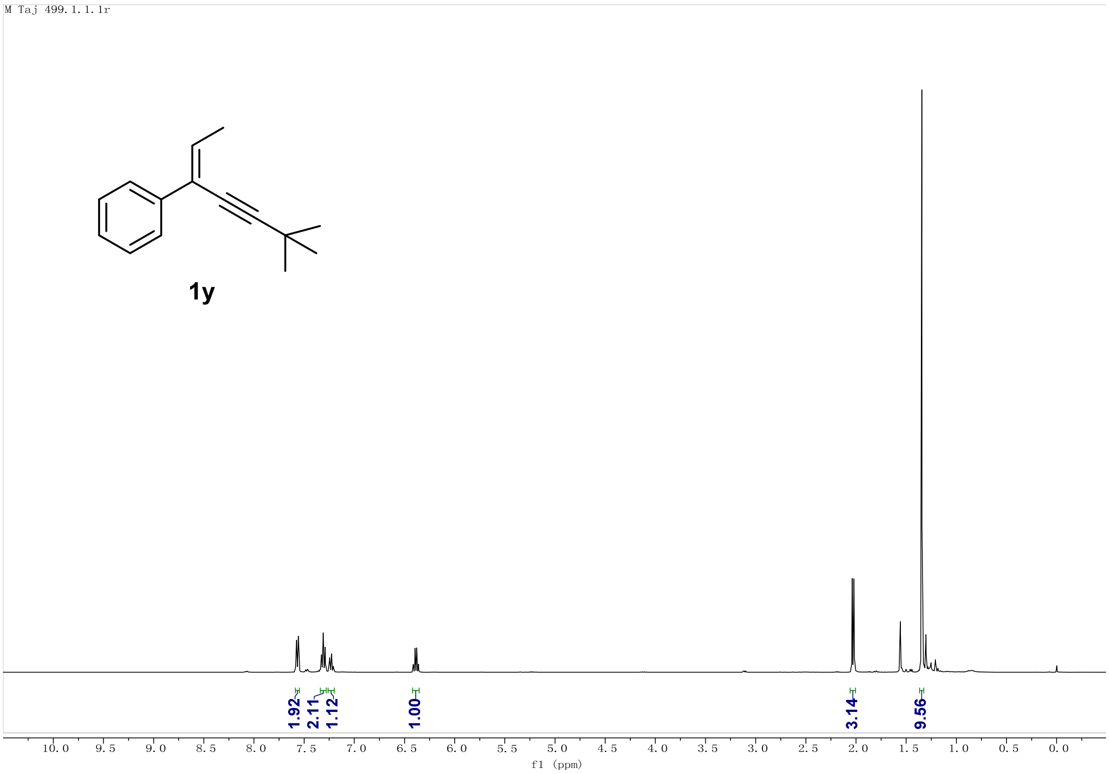

Supplementary Figure 54. **^{1}H NMR spectrum of compound 1y.**

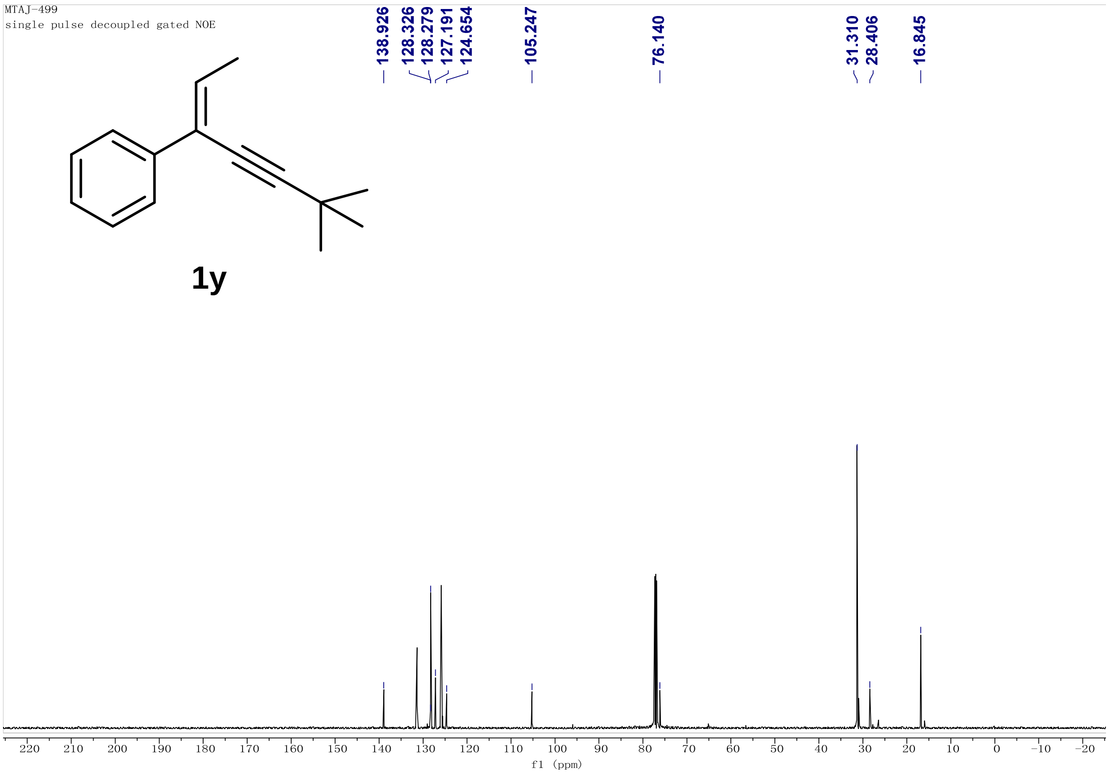

Supplementary Figure 55. **^{13}C NMR spectrum of compound 1y.**

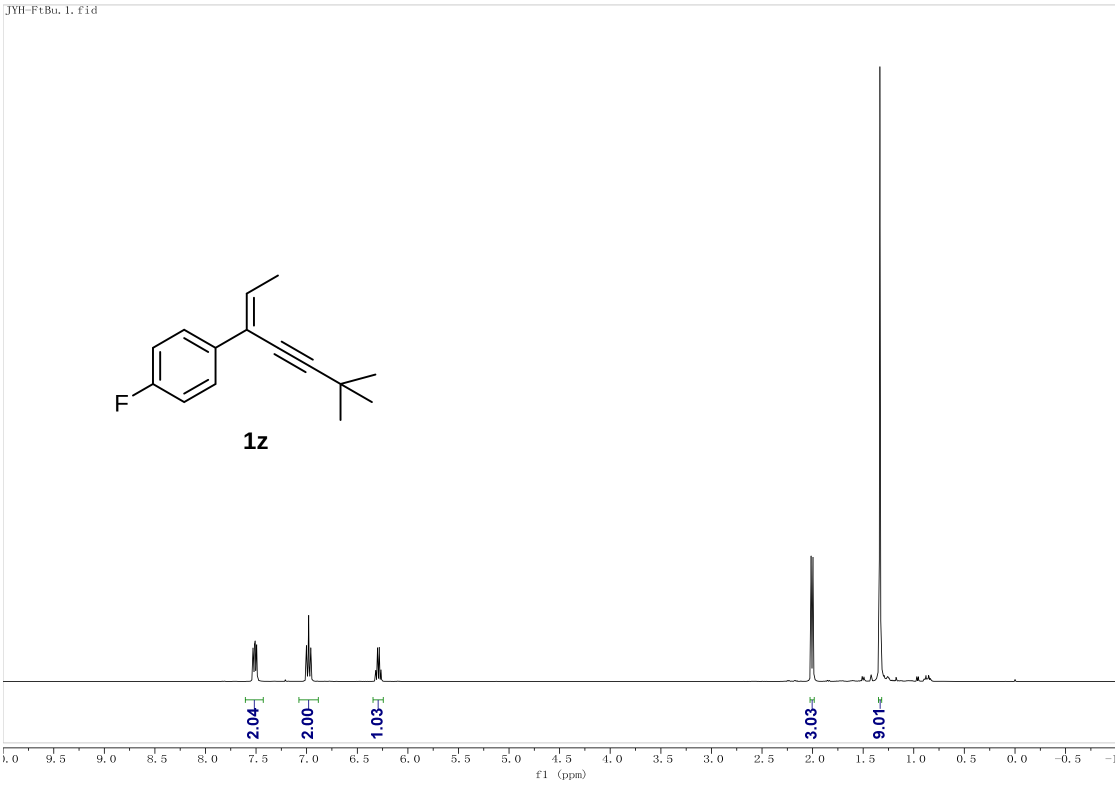

Supplementary Figure 56. ^{1}H NMR spectrum of compound 1z.

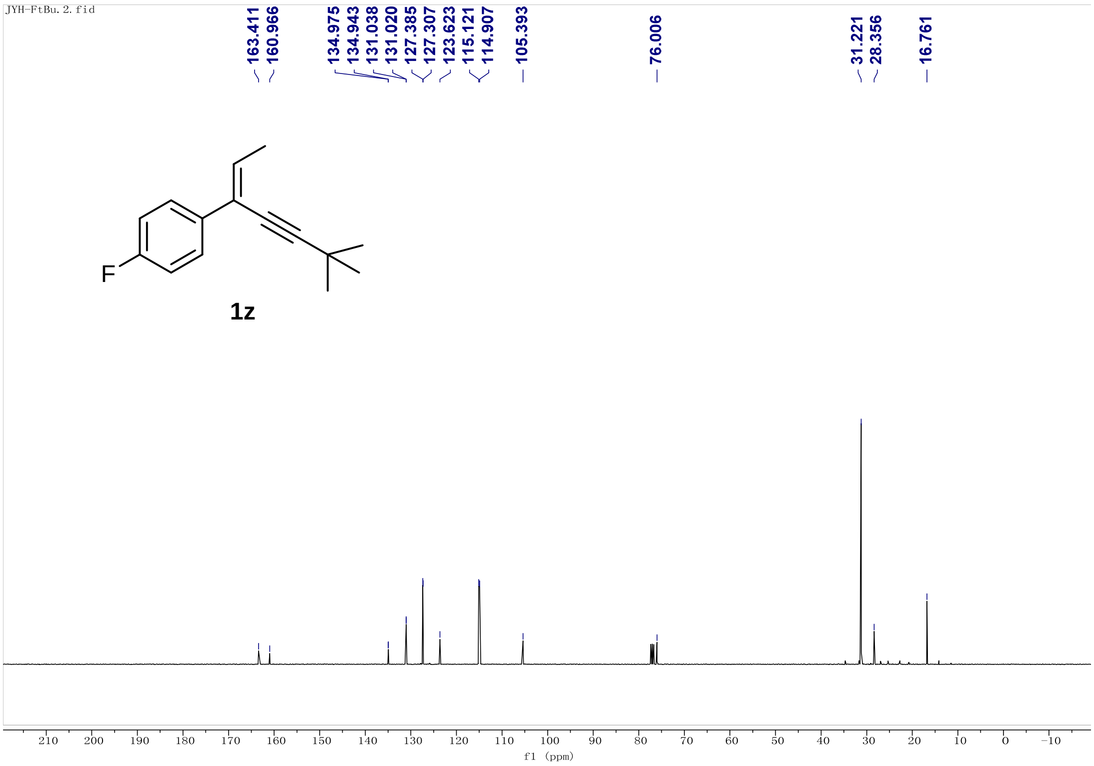

Supplementary Figure 57. ^{13}C NMR spectrum of compound 1z.

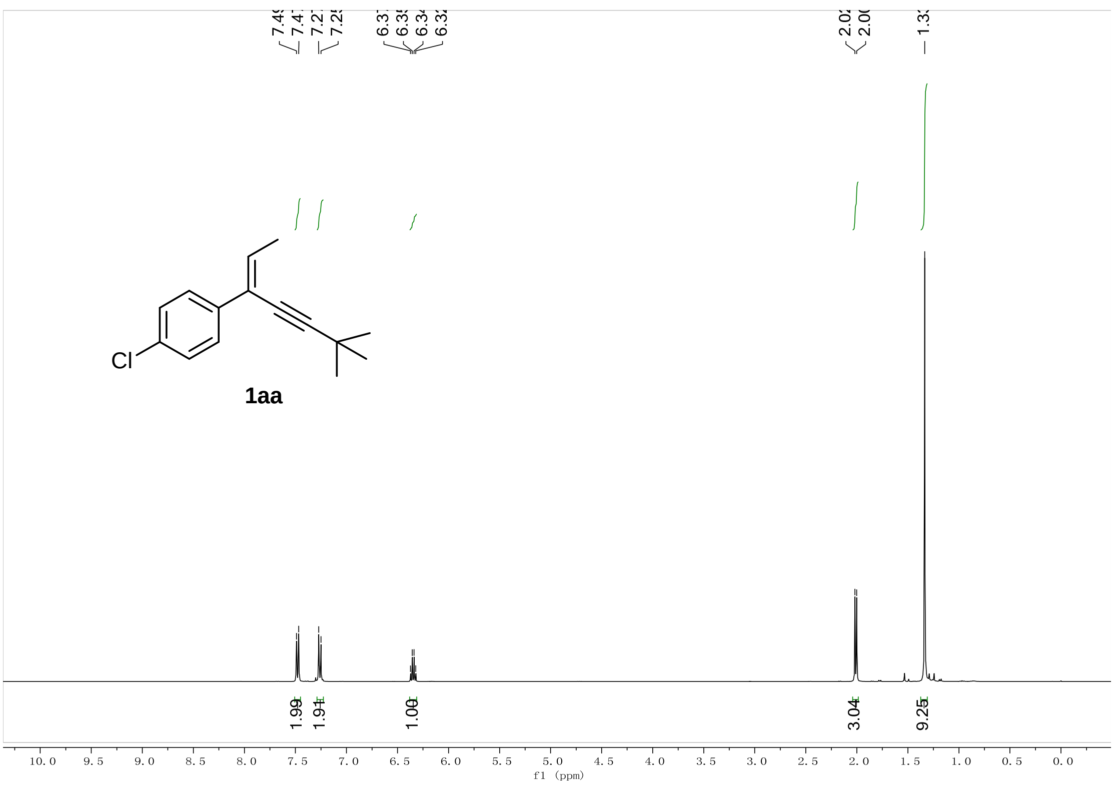

Supplementary Figure 58. ^{1}**H NMR spectrum of compound 1aa.**

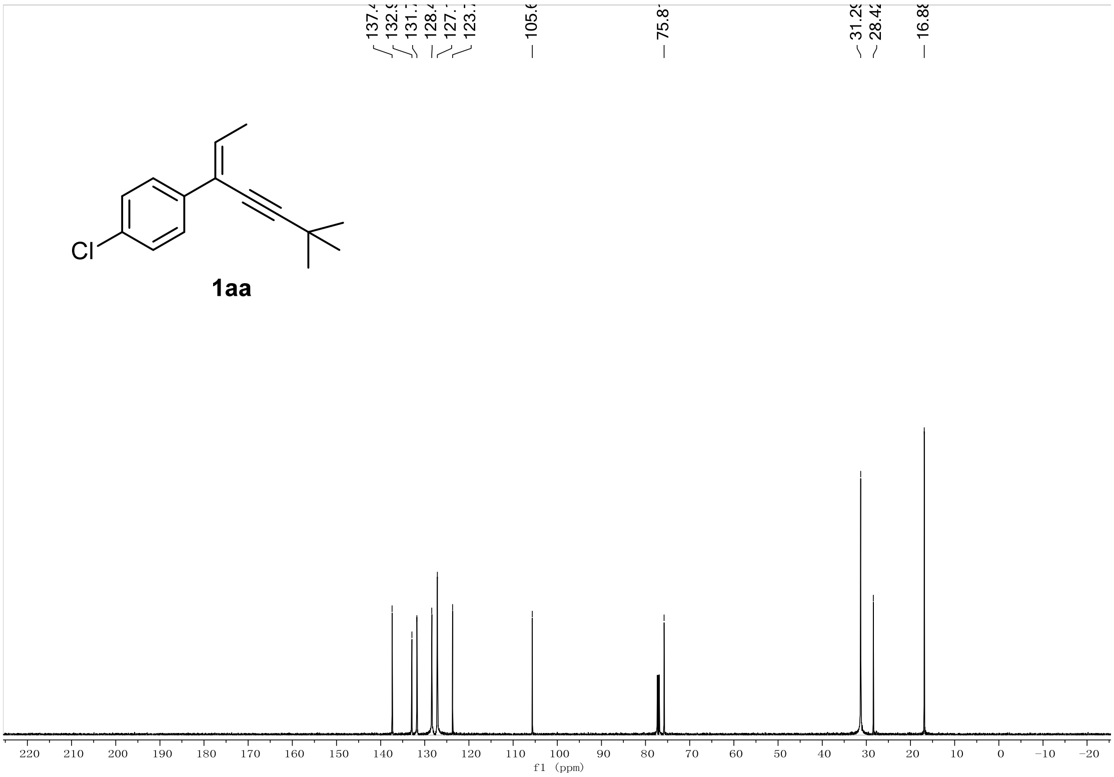

Supplementary Figure 59. ^{13}**C NMR spectrum of compound 1aa.**

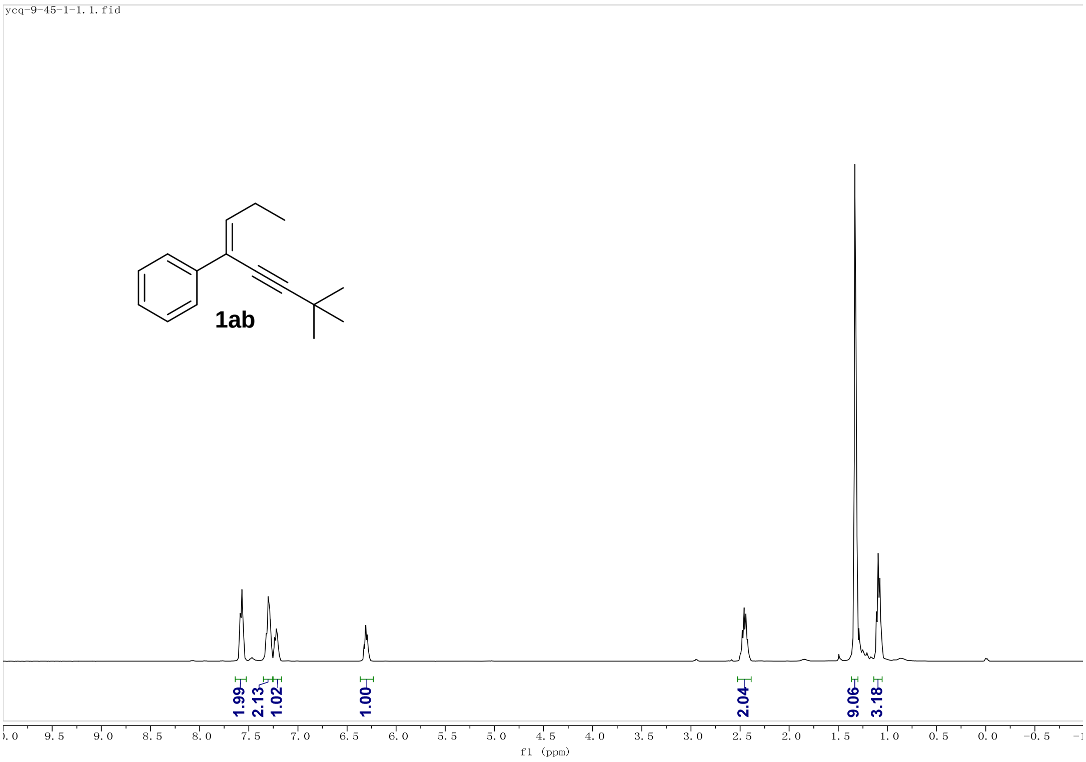

Supplementary Figure 60. **^{1}H NMR spectrum of compound 1ab.**

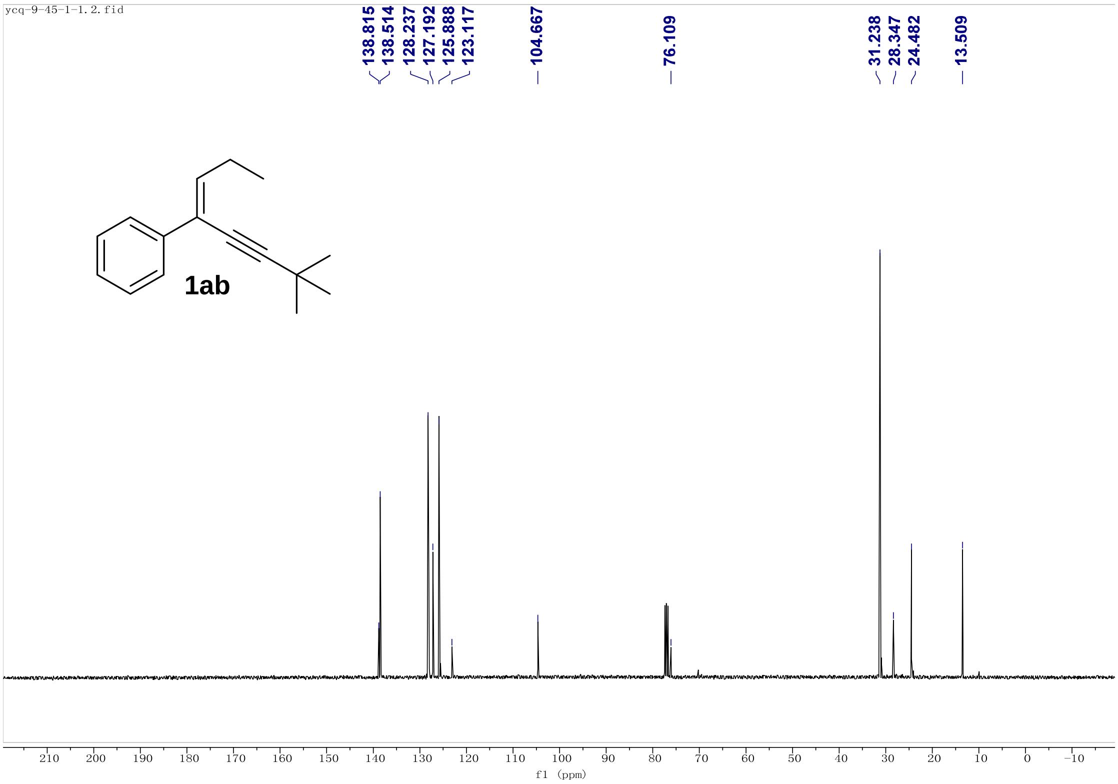

Supplementary Figure 61. **^{13}C NMR spectrum of compound 1ab.**

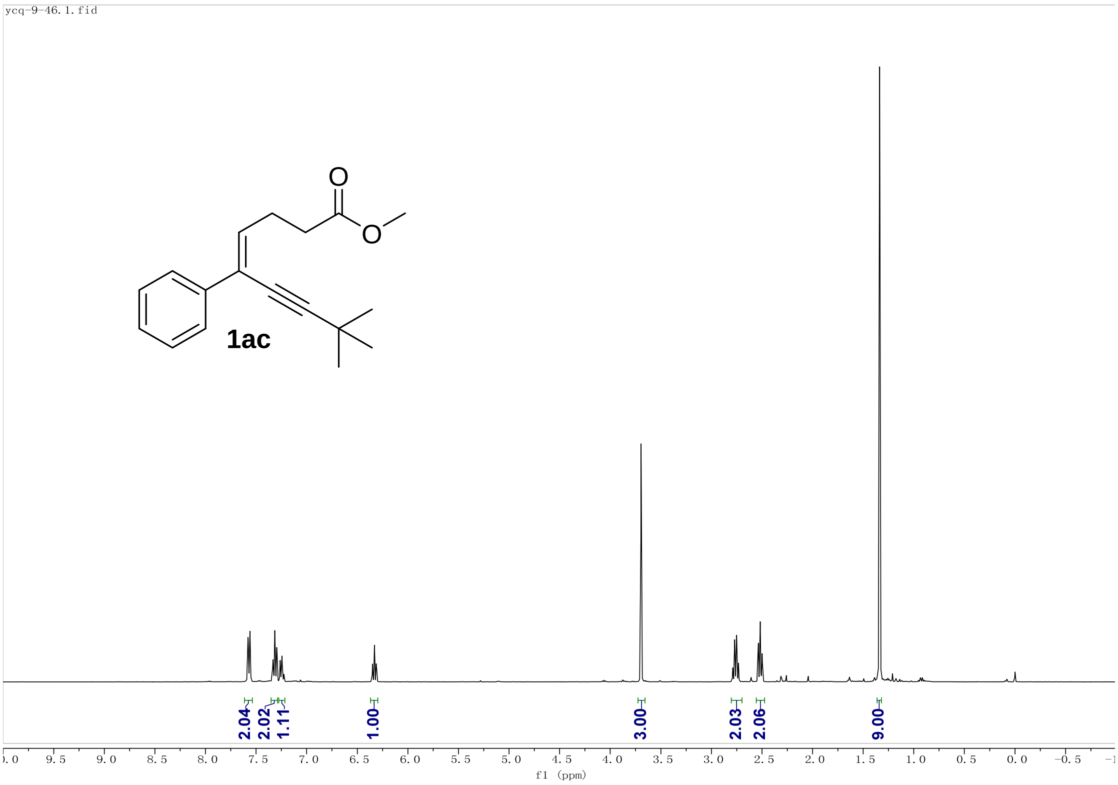

Supplementary Figure 62. ^{1}H NMR spectrum of compound 1ac.

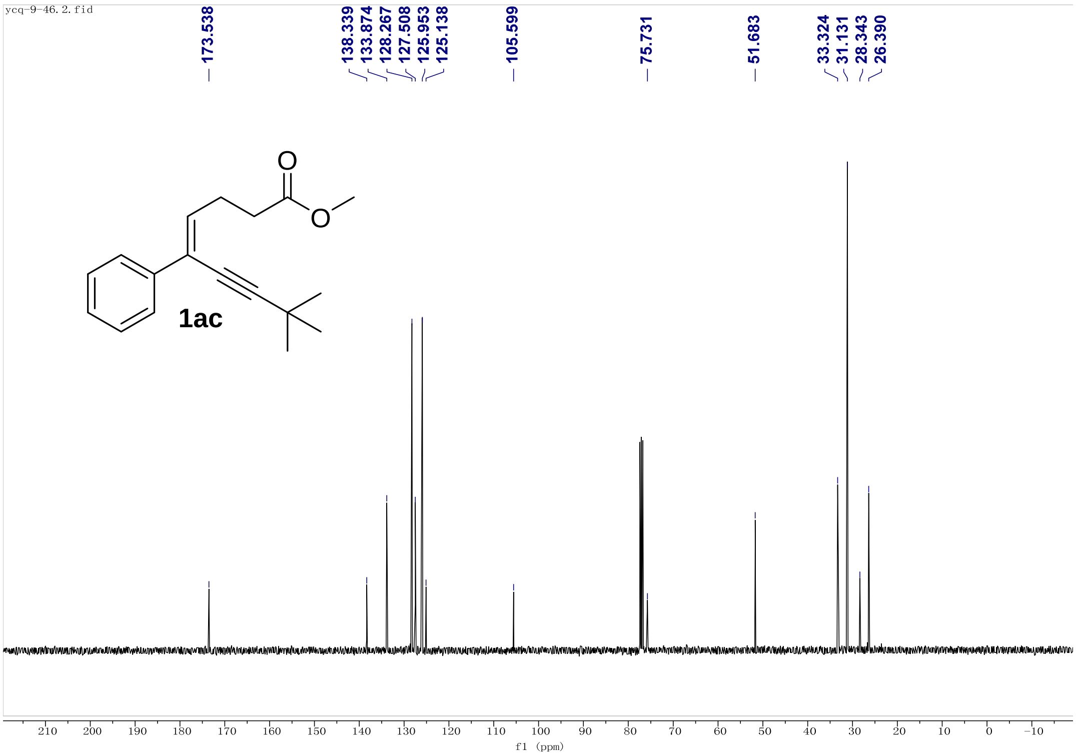

Supplementary Figure 63. ^{13}C NMR spectrum of compound 1ac.

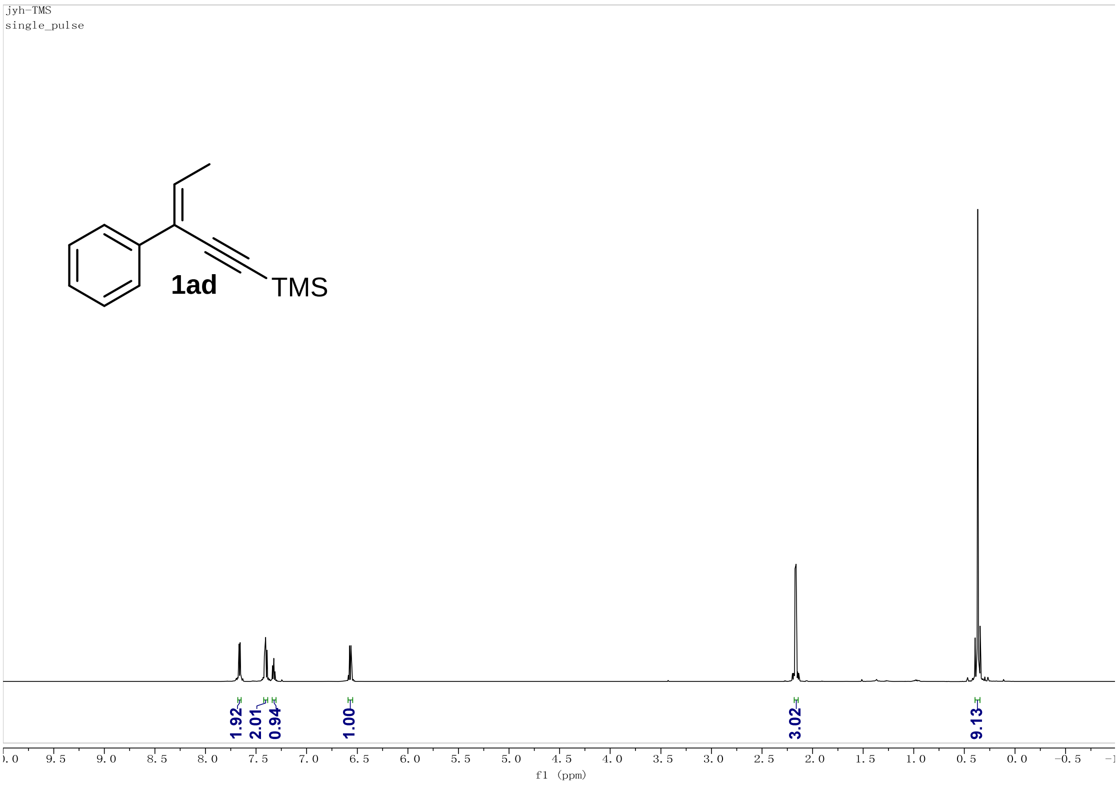

Supplementary Figure 64. ^{1}H NMR spectrum of compound 1ad.

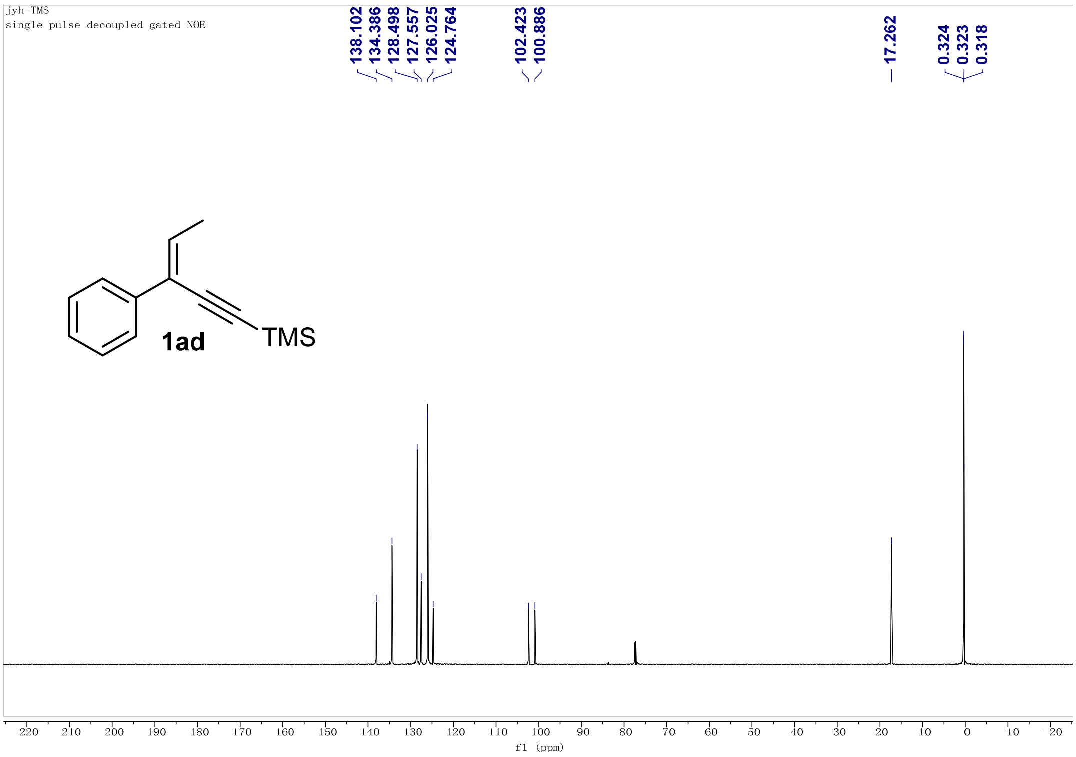

Supplementary Figure 65. ^{13}C **NMR spectrum of compound 1ad.**

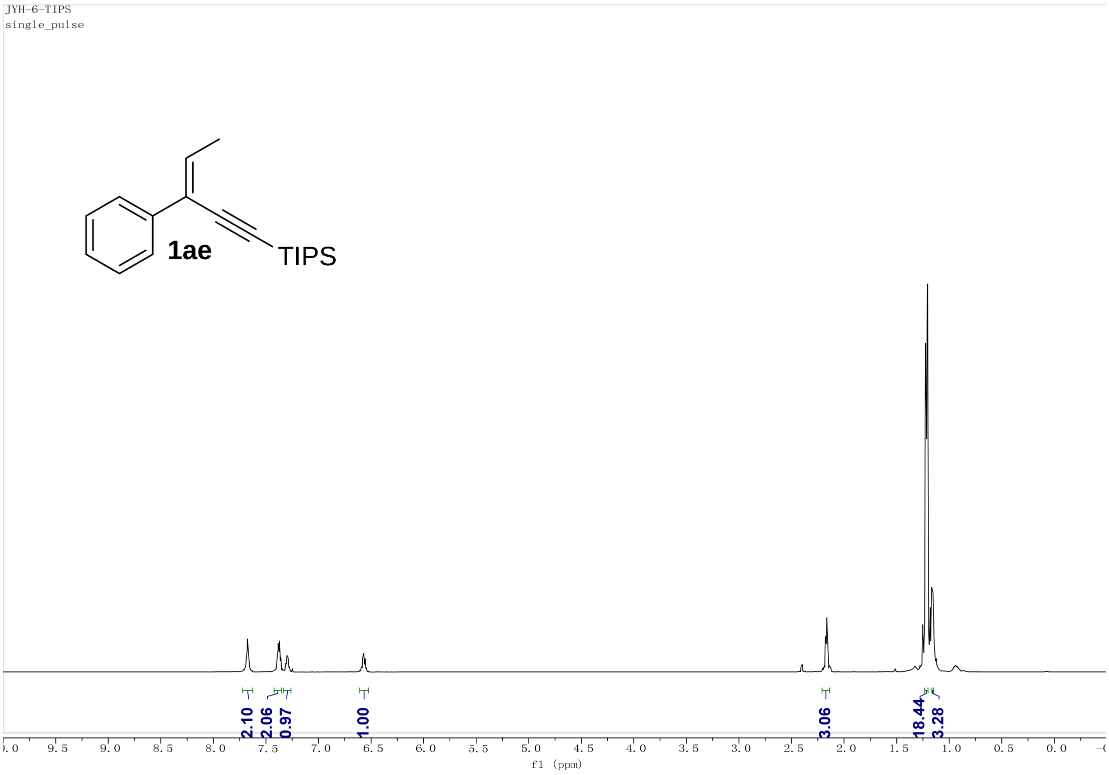

Supplementary Figure 66. ^{1}H NMR spectrum of compound 1ae.

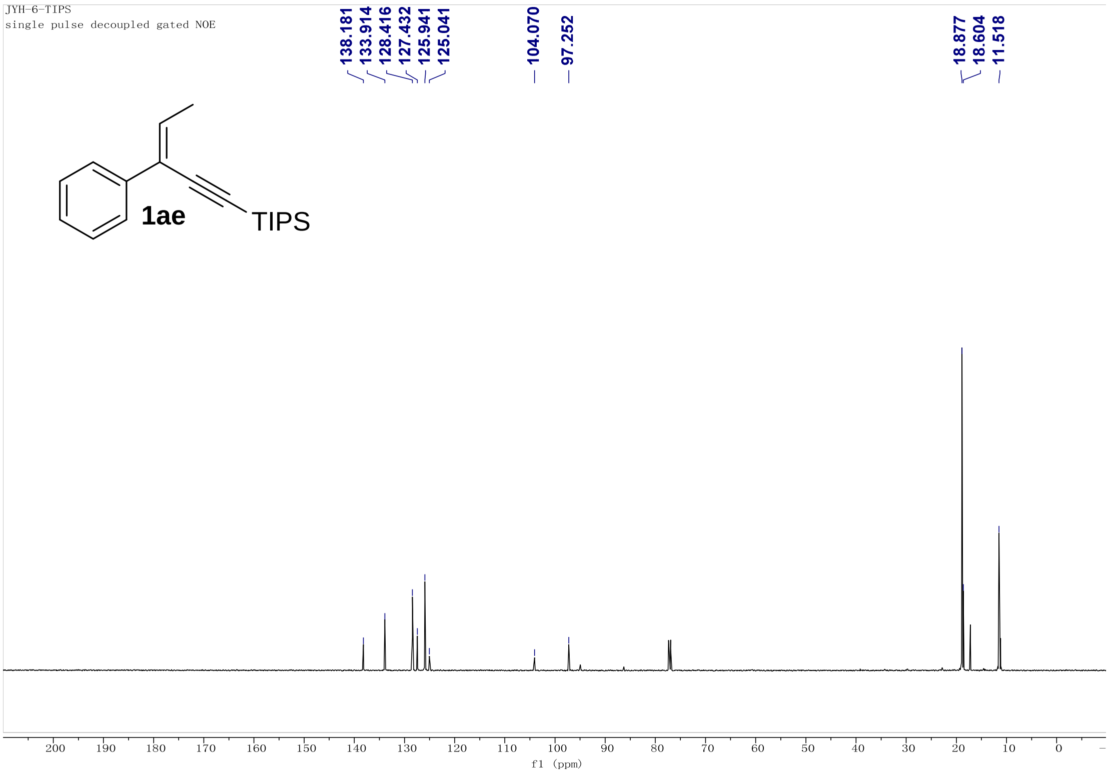

Supplementary Figure 67. ^{13}C NMR spectrum of compound 1ae.

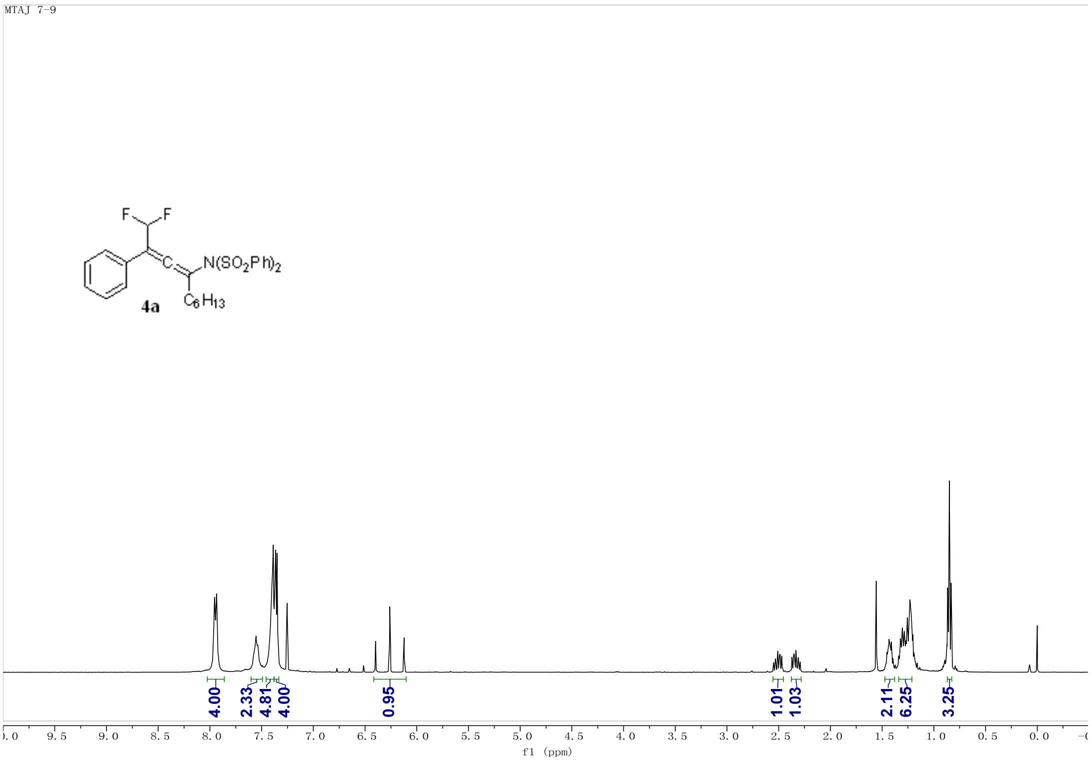

Supplementary Figure 68. ^{1}H NMR spectrum of compound 4a.

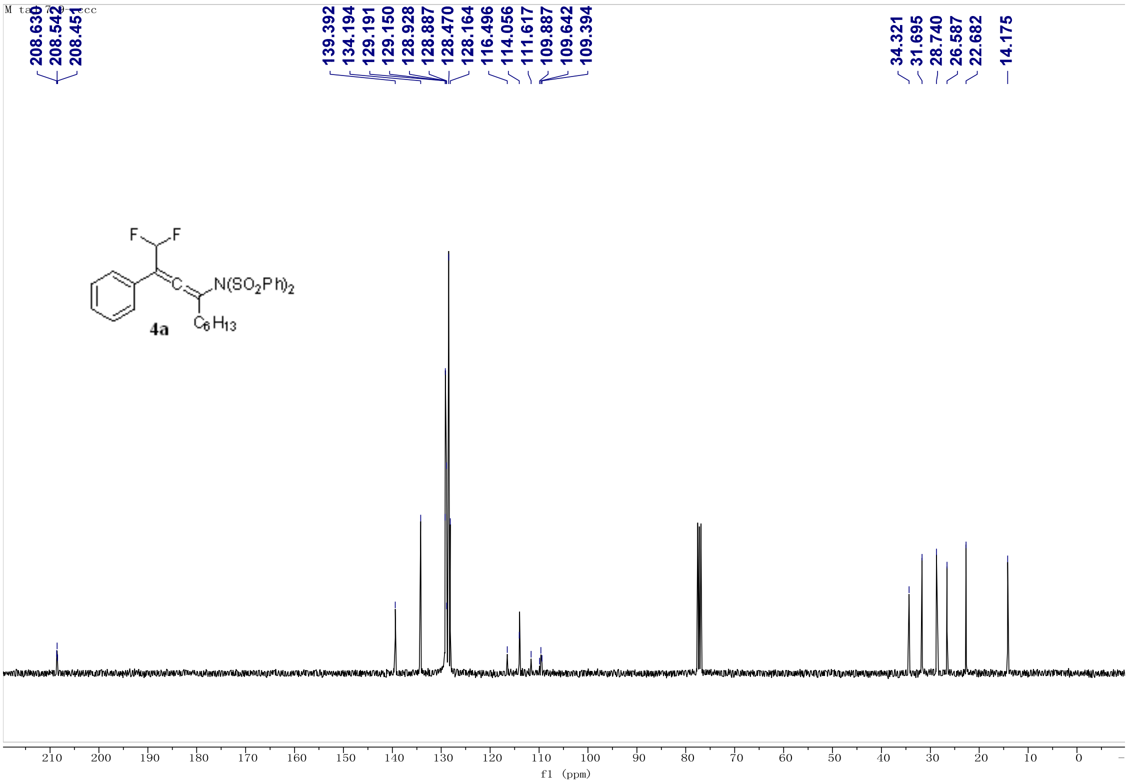

Supplementary Figure 69. ^{13}C NMR spectrum of compound 4a.

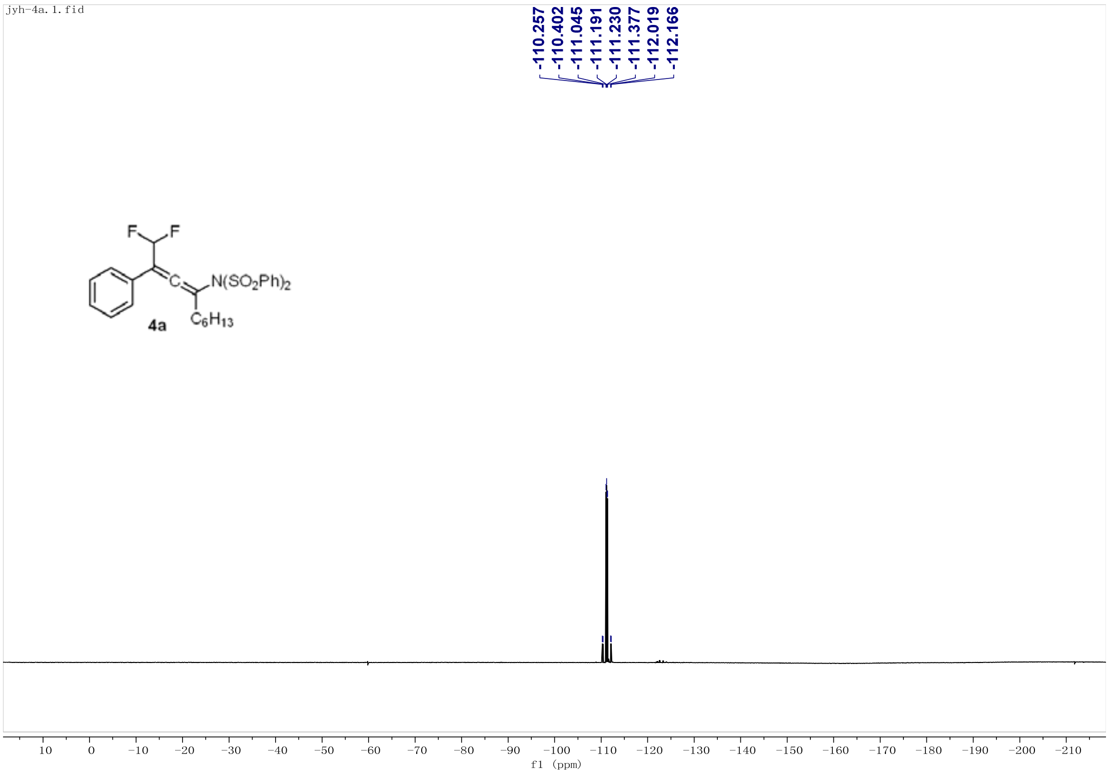

Supplementary Figure 70. ^{19}F **NMR spectrum of compound 4a.**

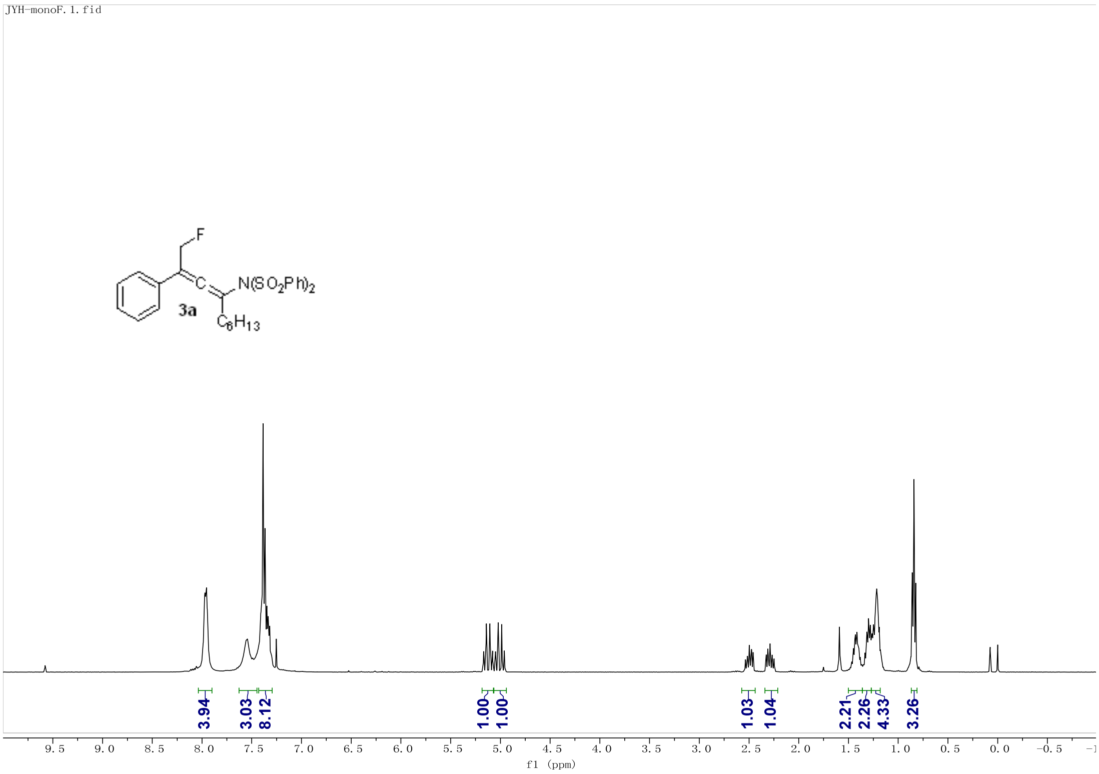

Supplementary Figure 71. ^{1}H **NMR spectrum of compound 3a.**

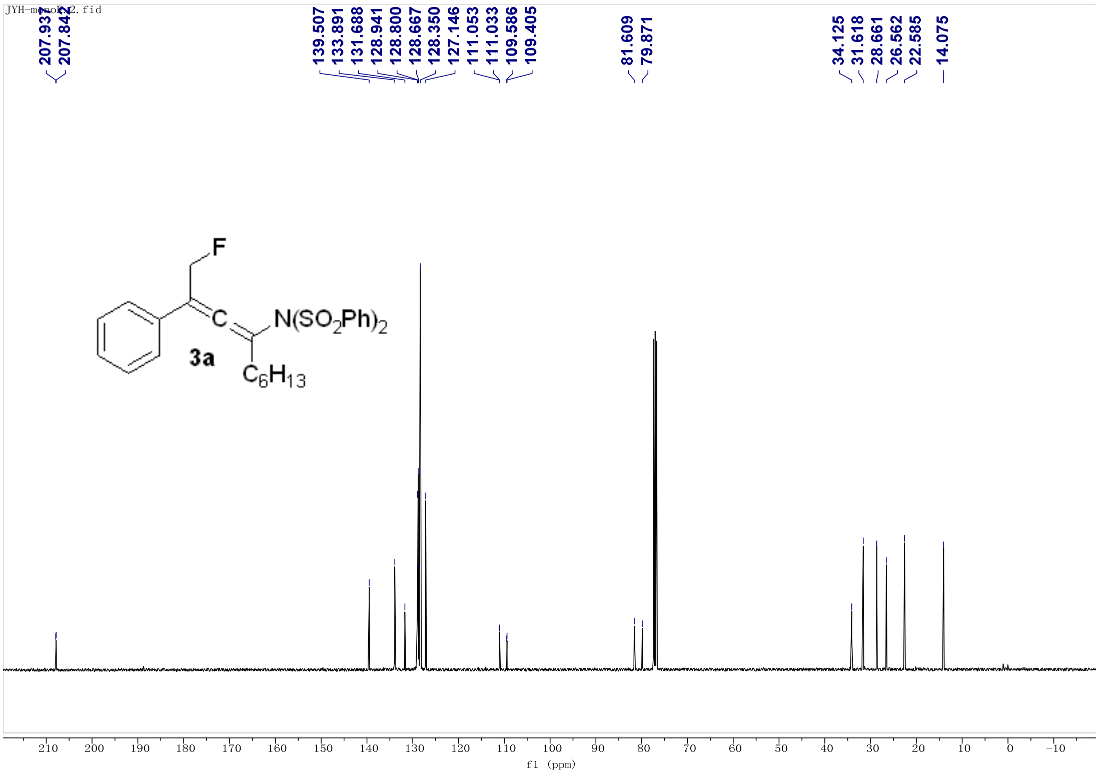

Supplementary Figure 72. ^{13}C NMR spectrum of compound 3a.

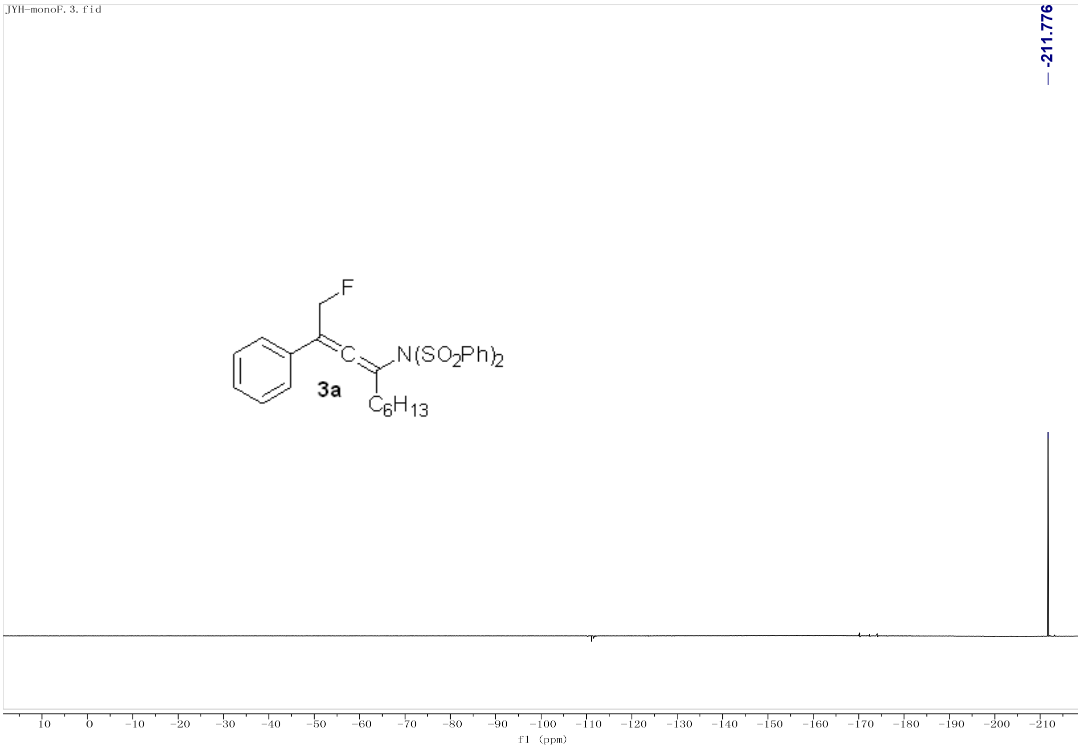

Supplementary Figure 73. ^{19}F NMR spectrum of compound 3a.

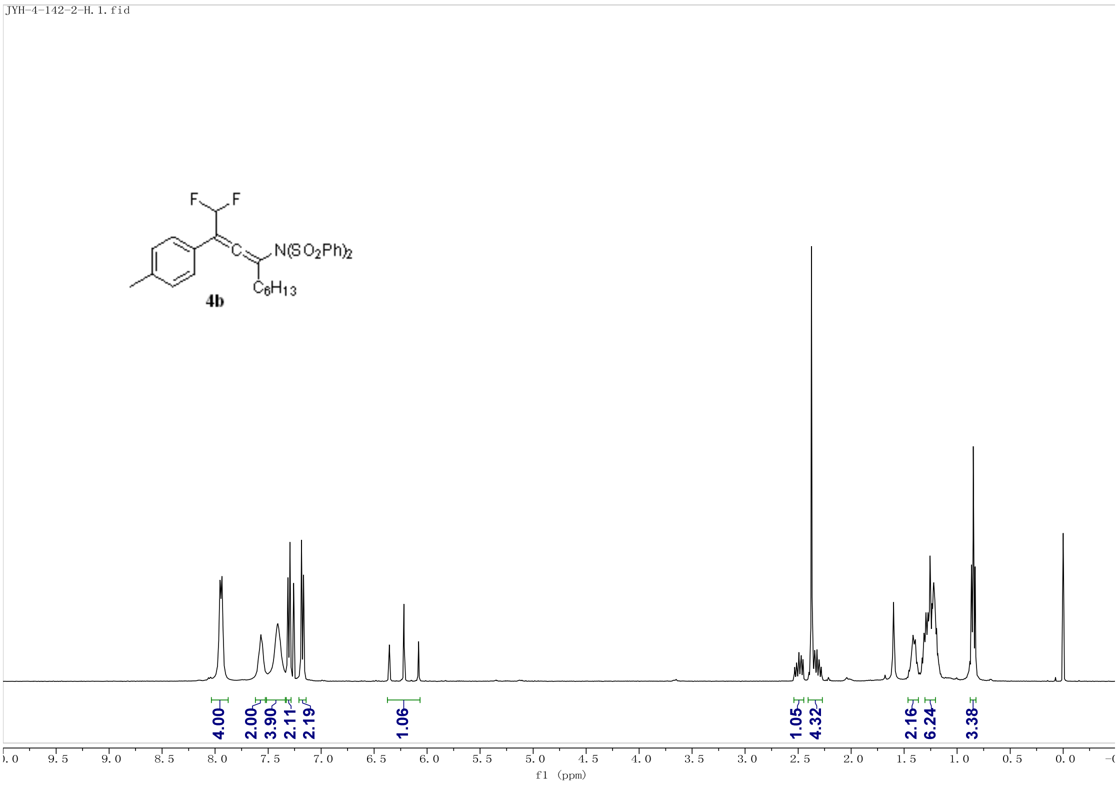

Supplementary Figure 74. ^{1}H NMR spectrum of compound 4b.

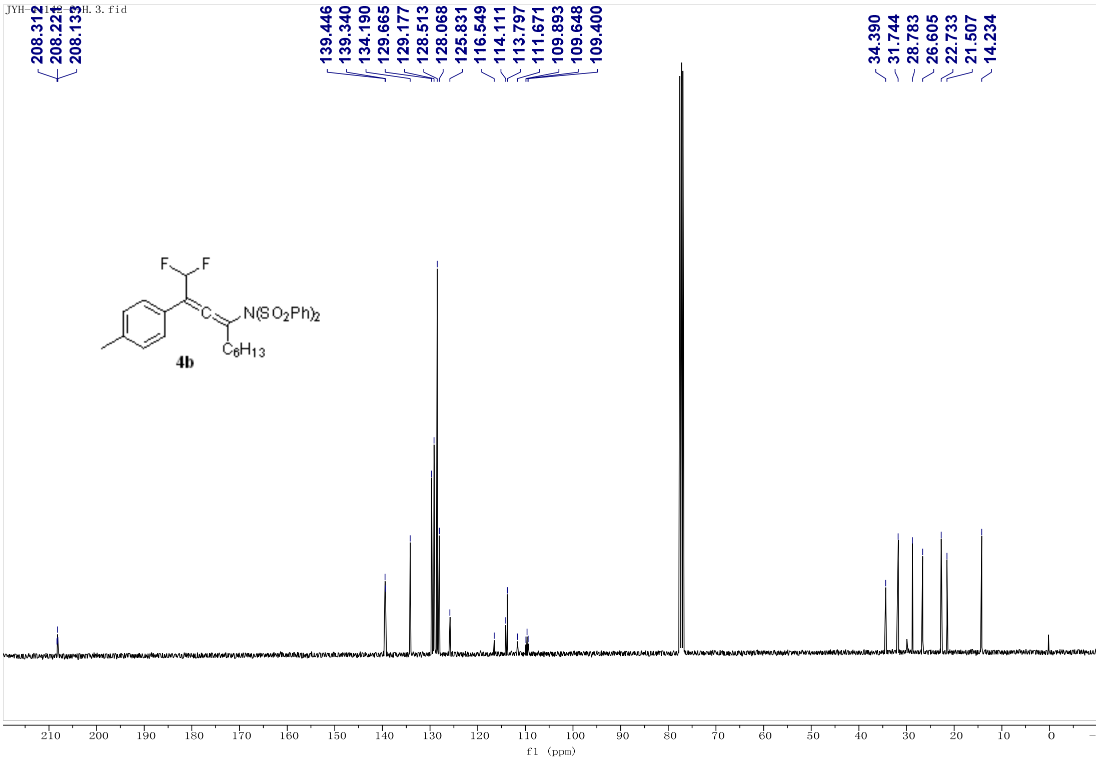

Supplementary Figure 75. ^{13}C NMR spectrum of compound 4b.

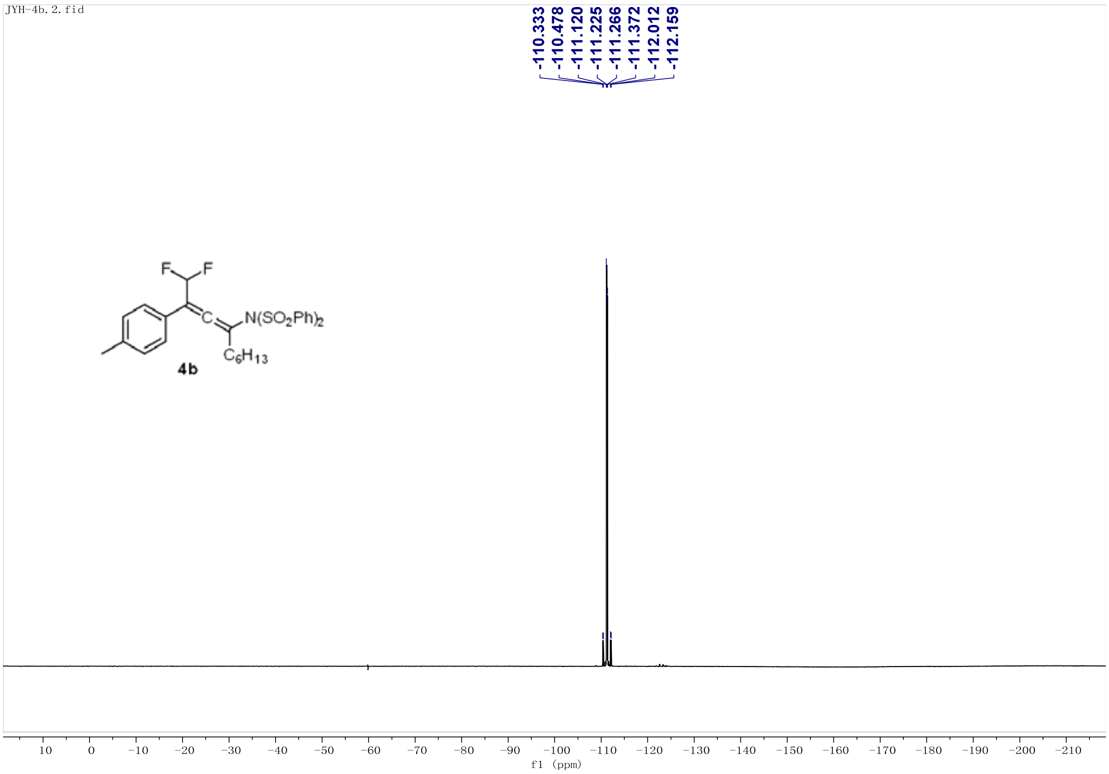

Supplementary Figure 76. ^{19}F NMR spectrum of compound 4b.

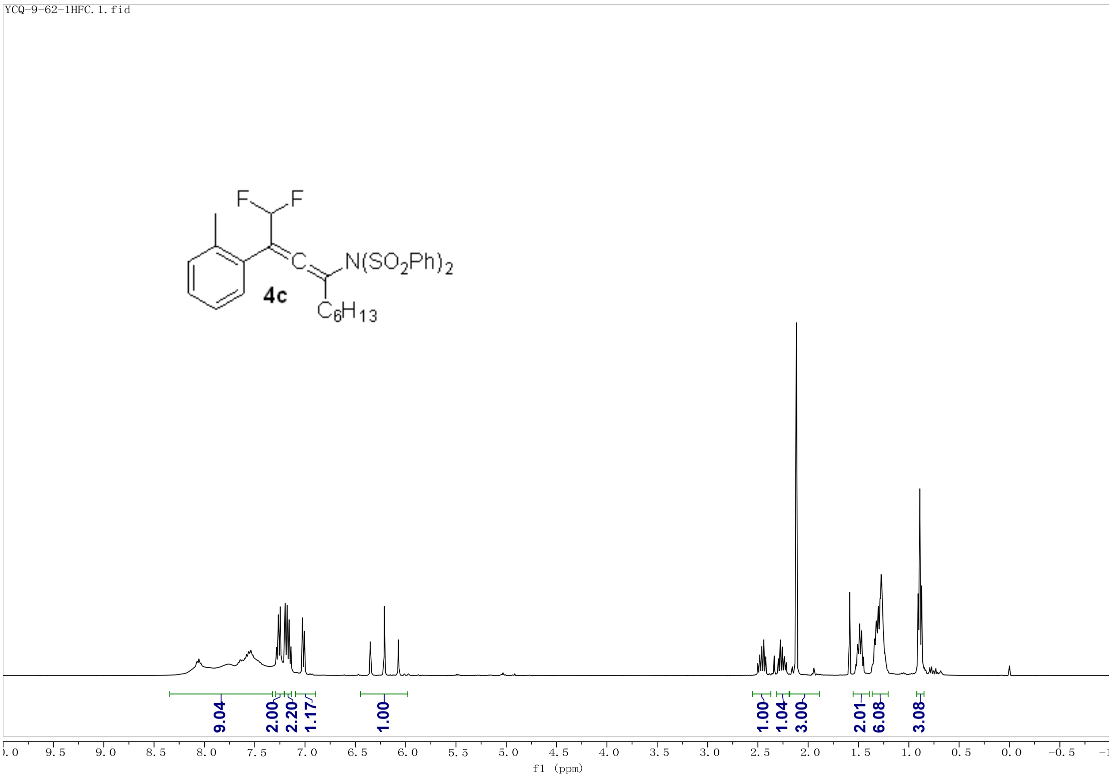

Supplementary Figure 77. ^{1}H NMR spectrum of compound 4c.

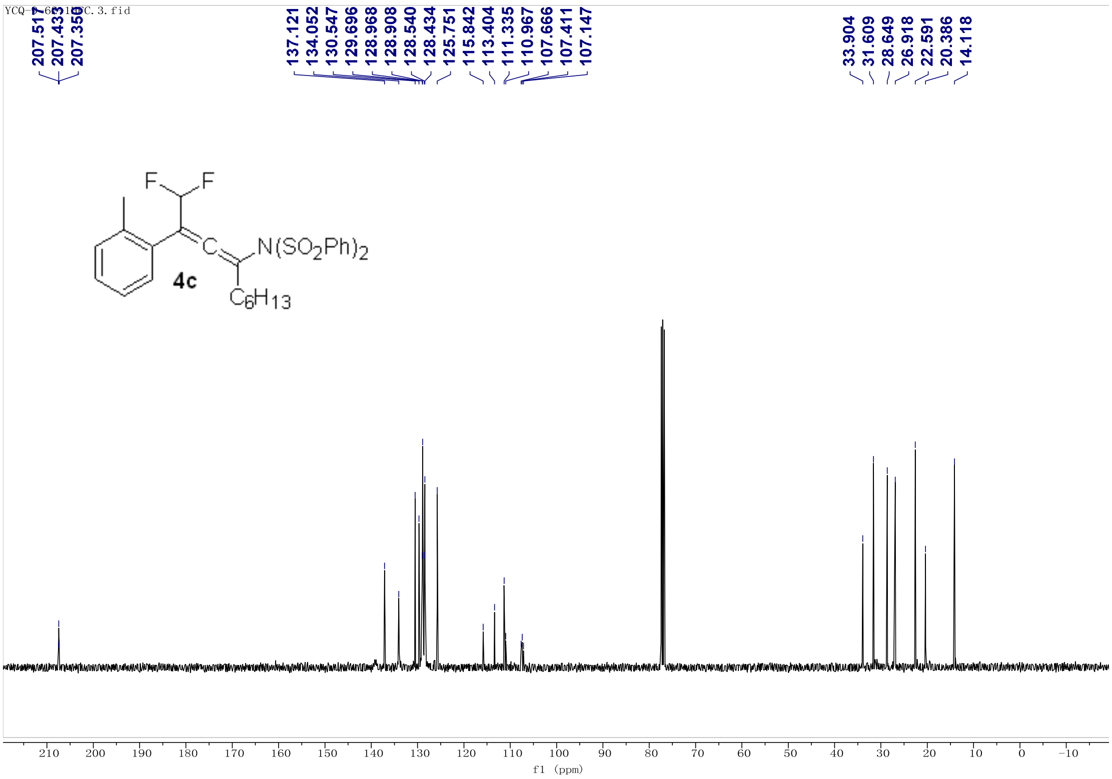

Supplementary Figure 78. **^{13}C NMR spectrum of compound 4b.**

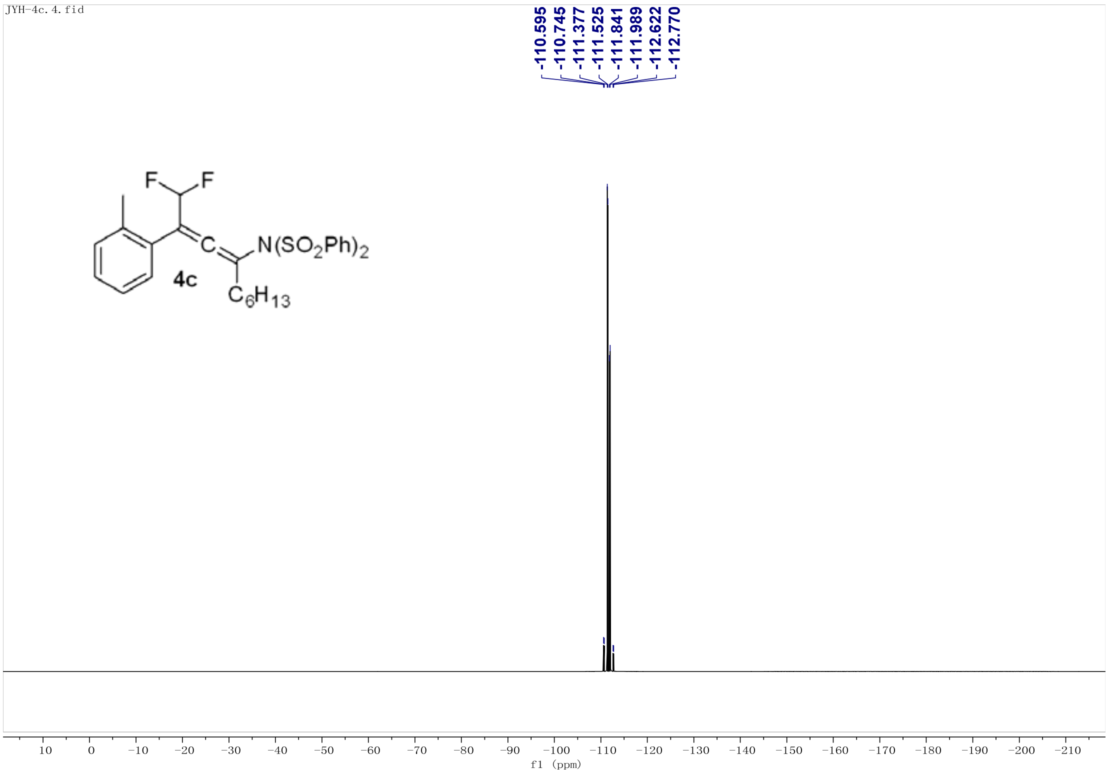

Supplementary Figure 79. **^{19}F NMR spectrum of compound 4c.**

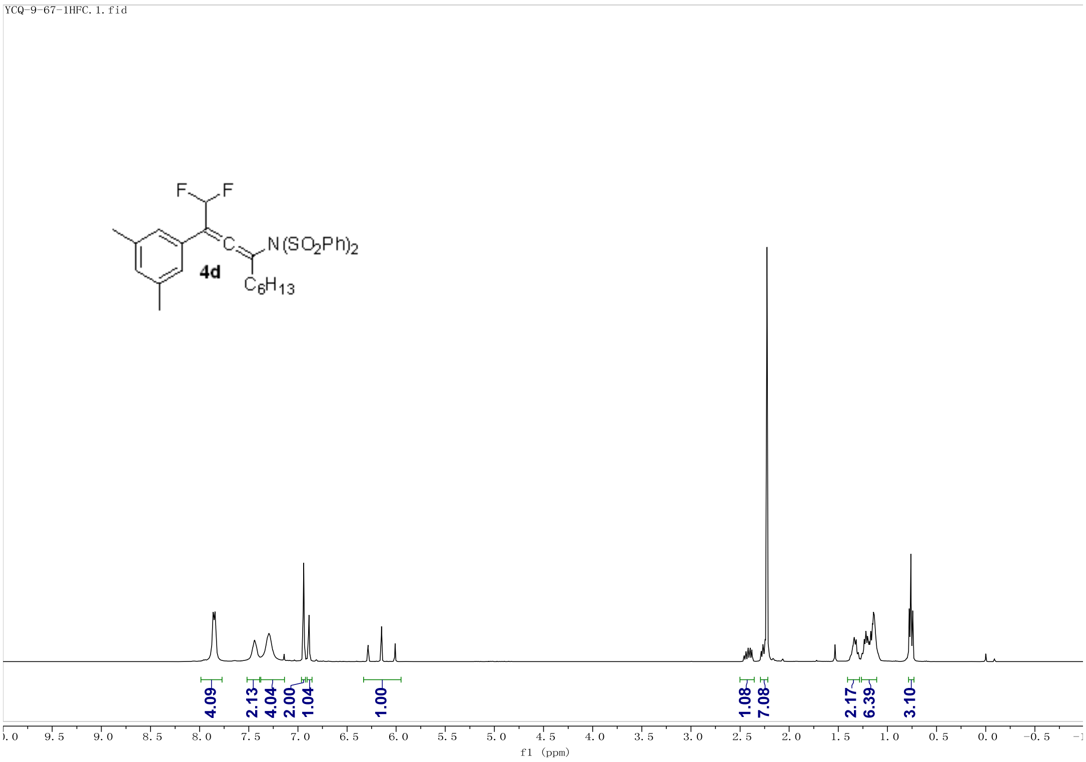

Supplementary Figure 80. **^{1}H NMR spectrum of compound 4d.**

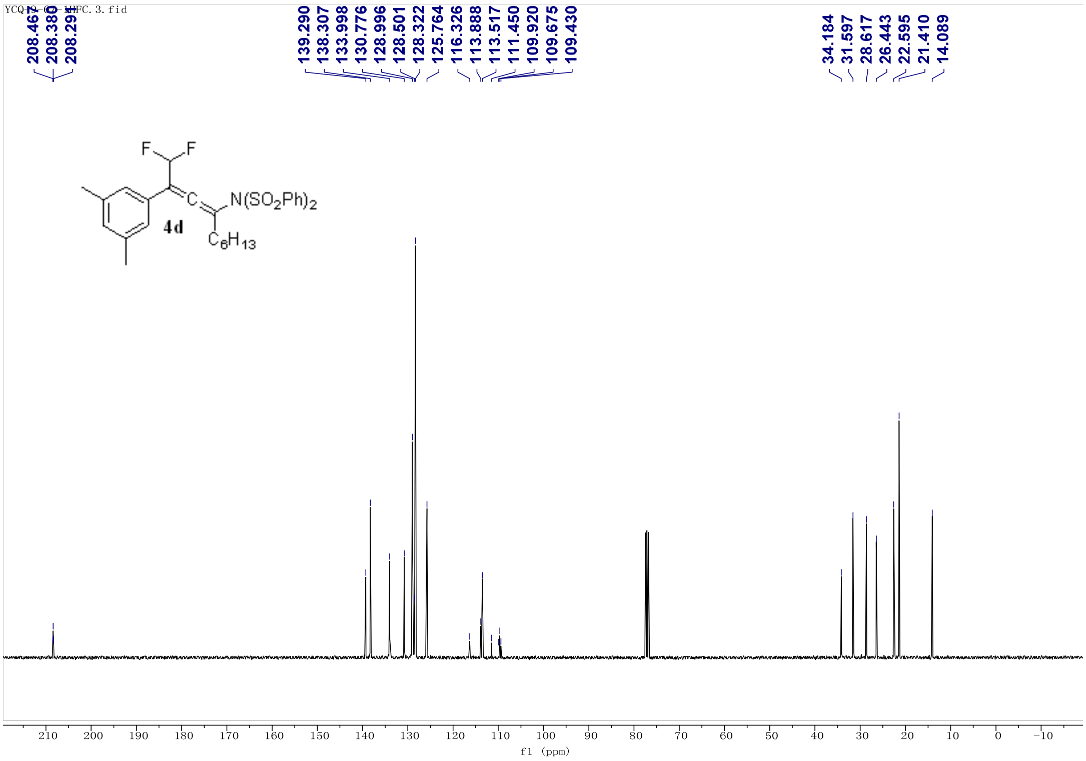

Supplementary Figure 81. **^{13}C NMR spectrum of compound 4d.**

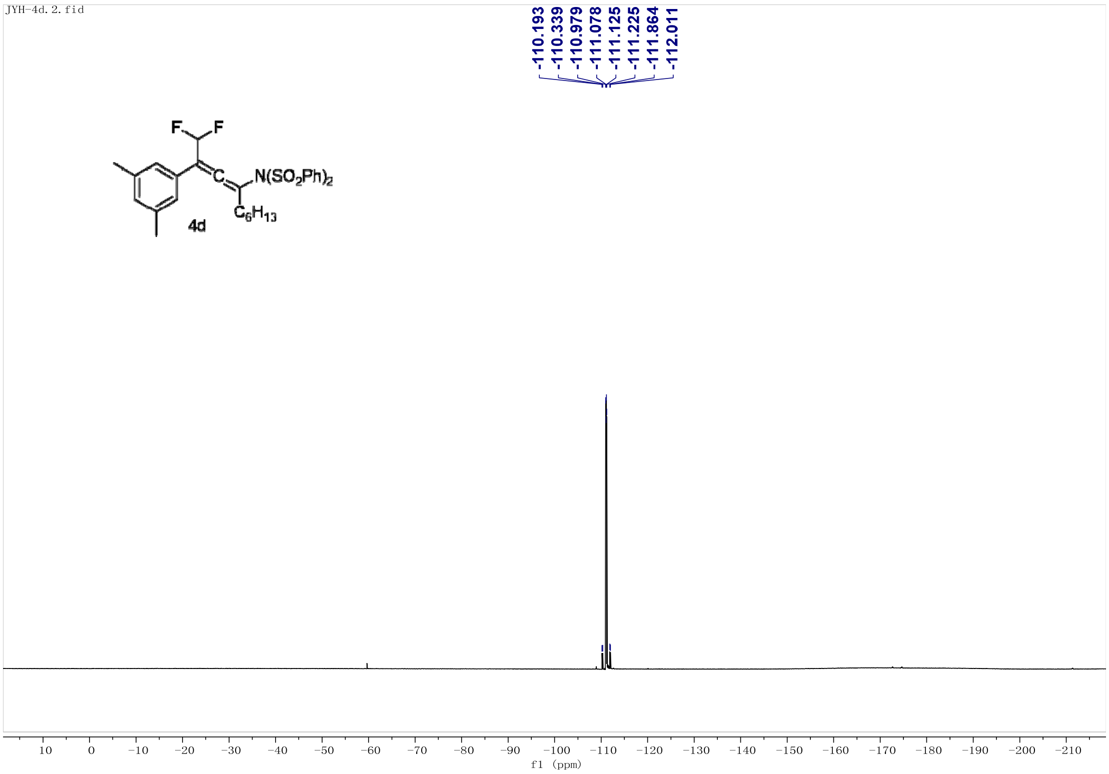

Supplementary Figure 82. ^{19}F **NMR spectrum of compound 4d.**

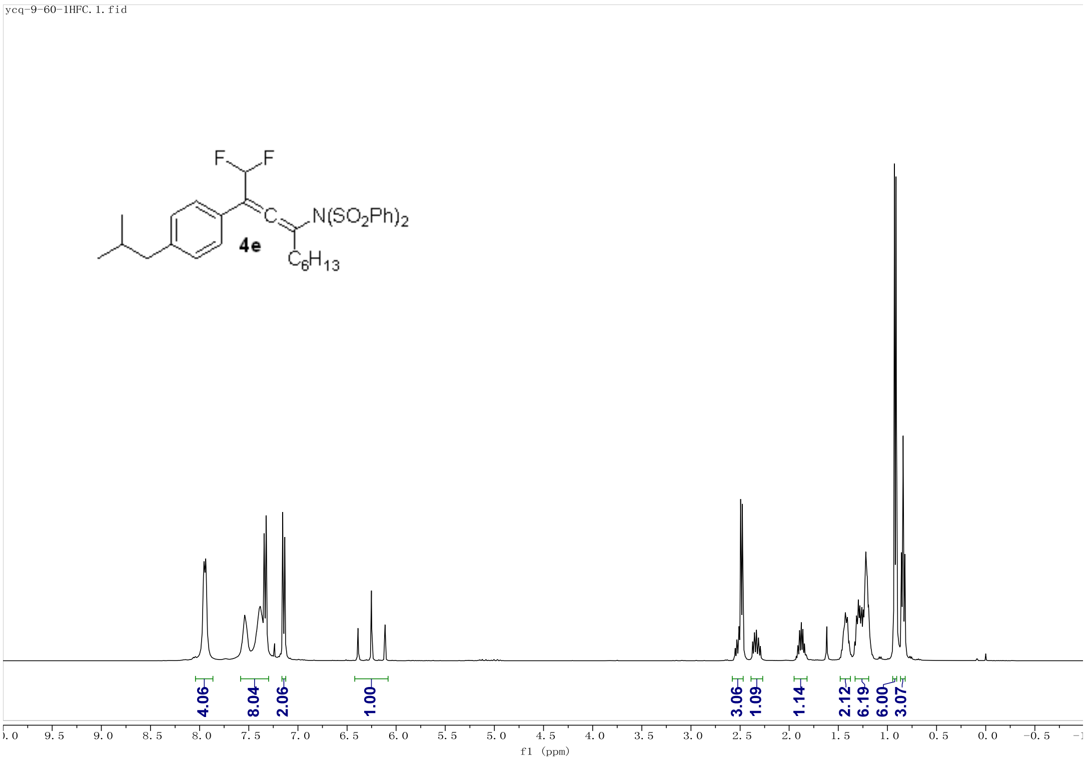

Supplementary Figure 83. ^{1}H **NMR spectrum of compound 4e.**

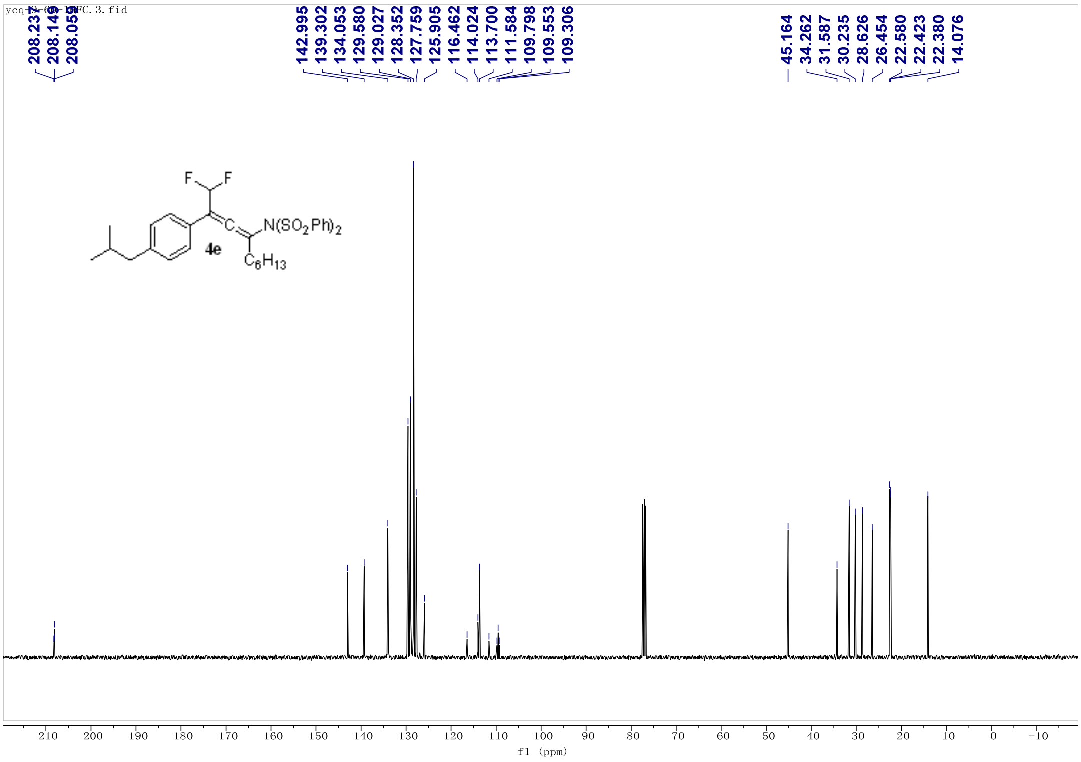

Supplementary Figure 84. ^{13}C NMR spectrum of compound 4e.

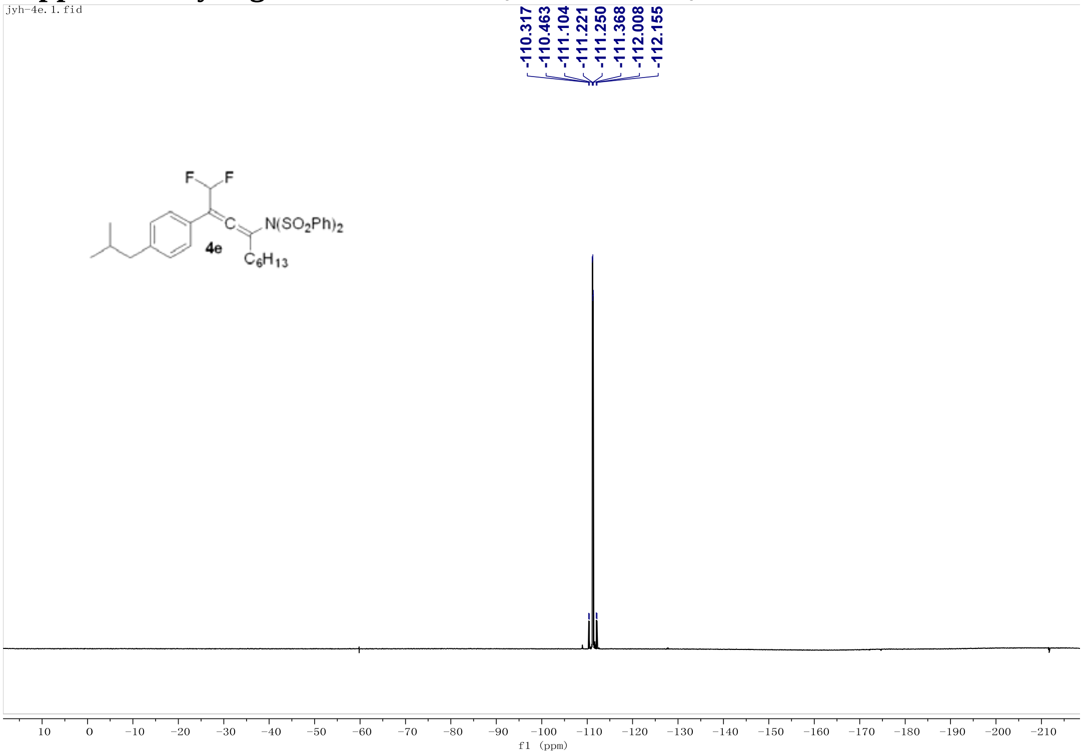

Supplementary Figure 85. ^{19}F NMR spectrum of compound 4e.

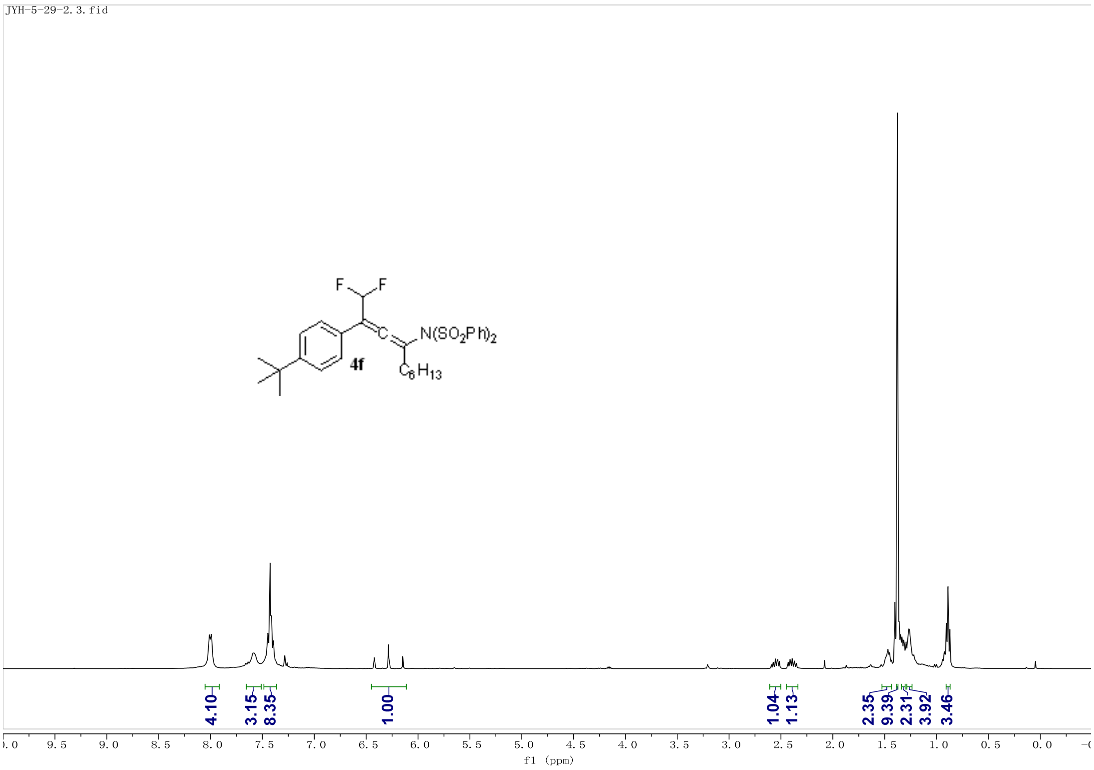

Supplementary Figure 86. ^{1}H NMR spectrum of compound 4f.

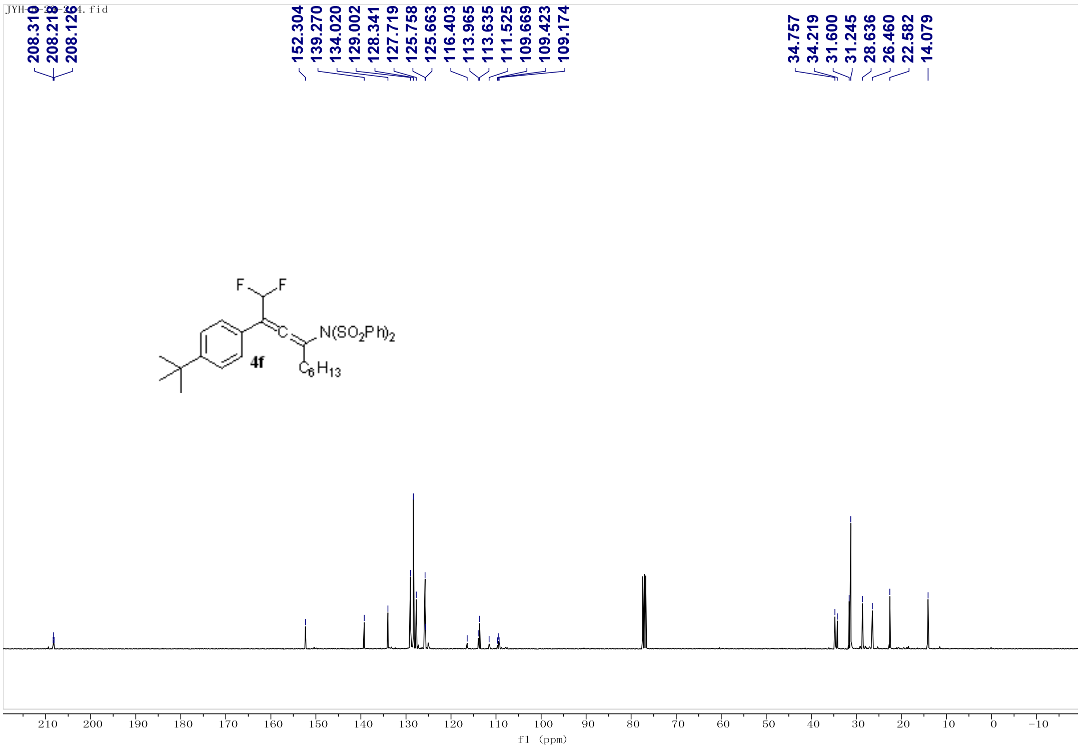

Supplementary Figure 87. ^{13}C NMR spectrum of compound 4f.

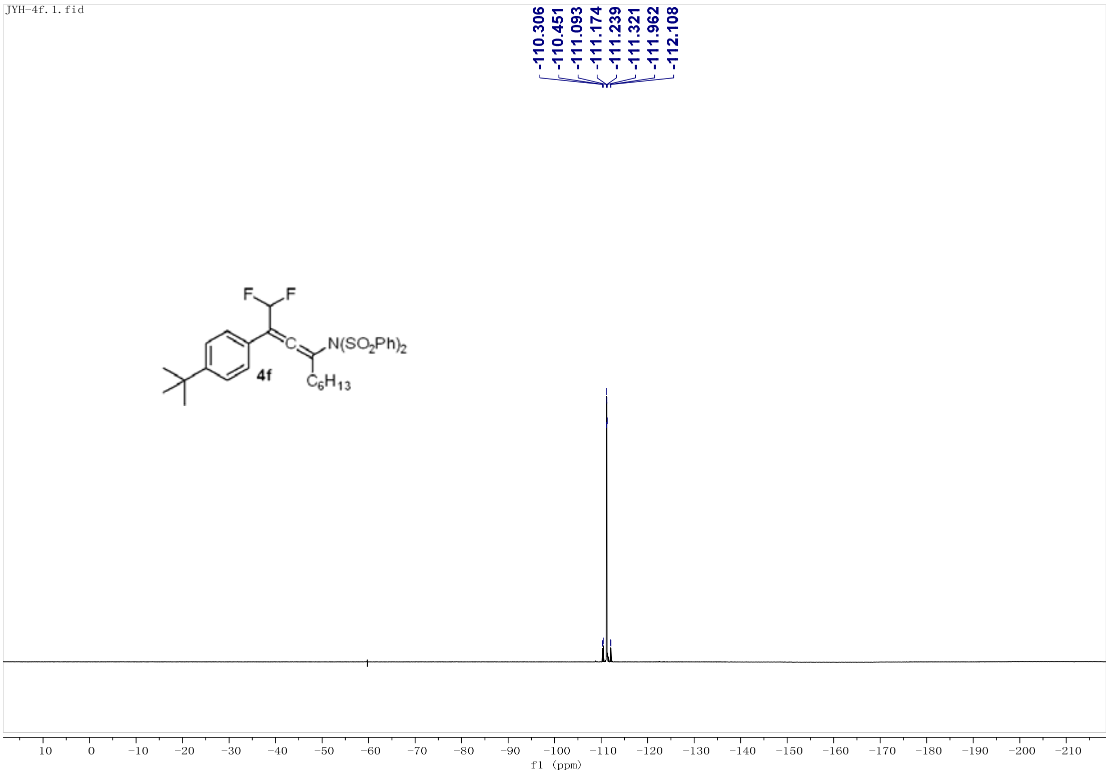

Supplementary Figure 88. ^{19}F **NMR spectrum of compound 4f.**

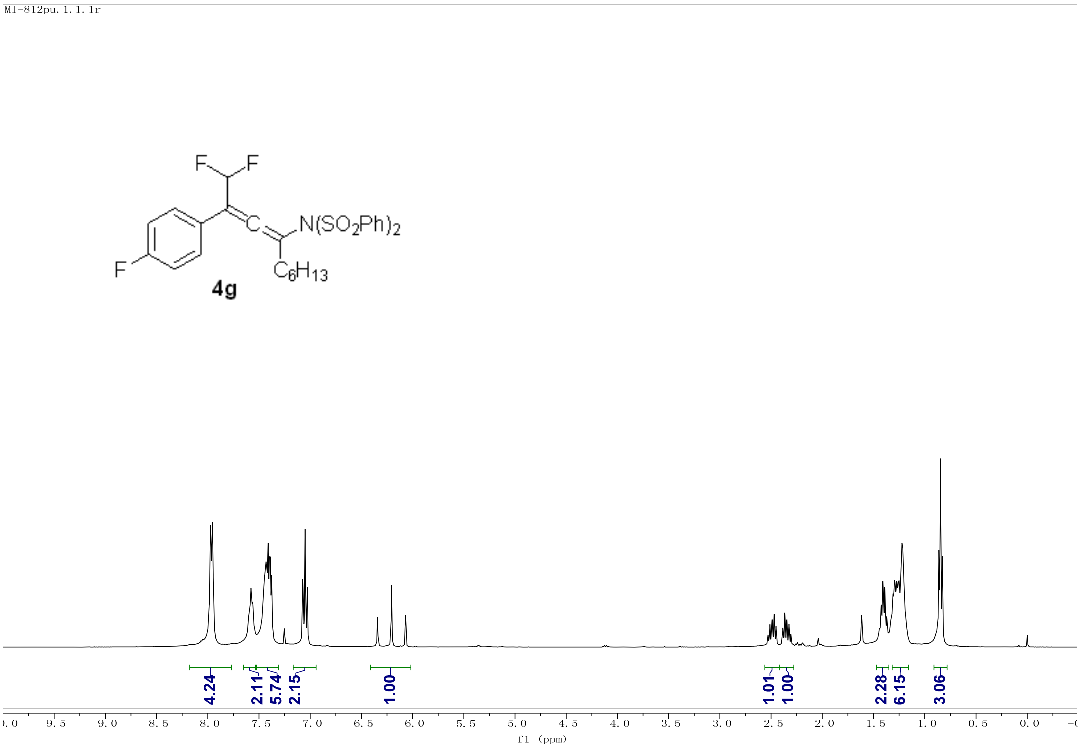

Supplementary Figure 89. ^{1}H **NMR spectrum of compound 4g.**

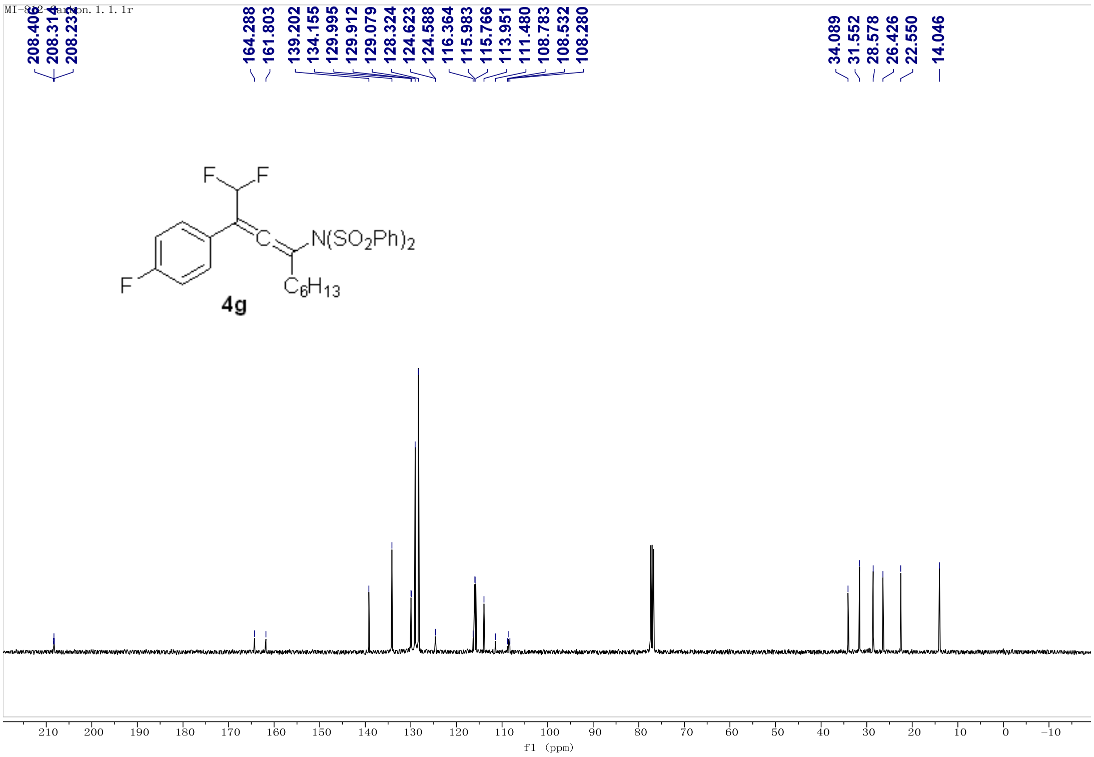

Supplementary Figure 90. ^{13}C **NMR spectrum of compound 4g.**

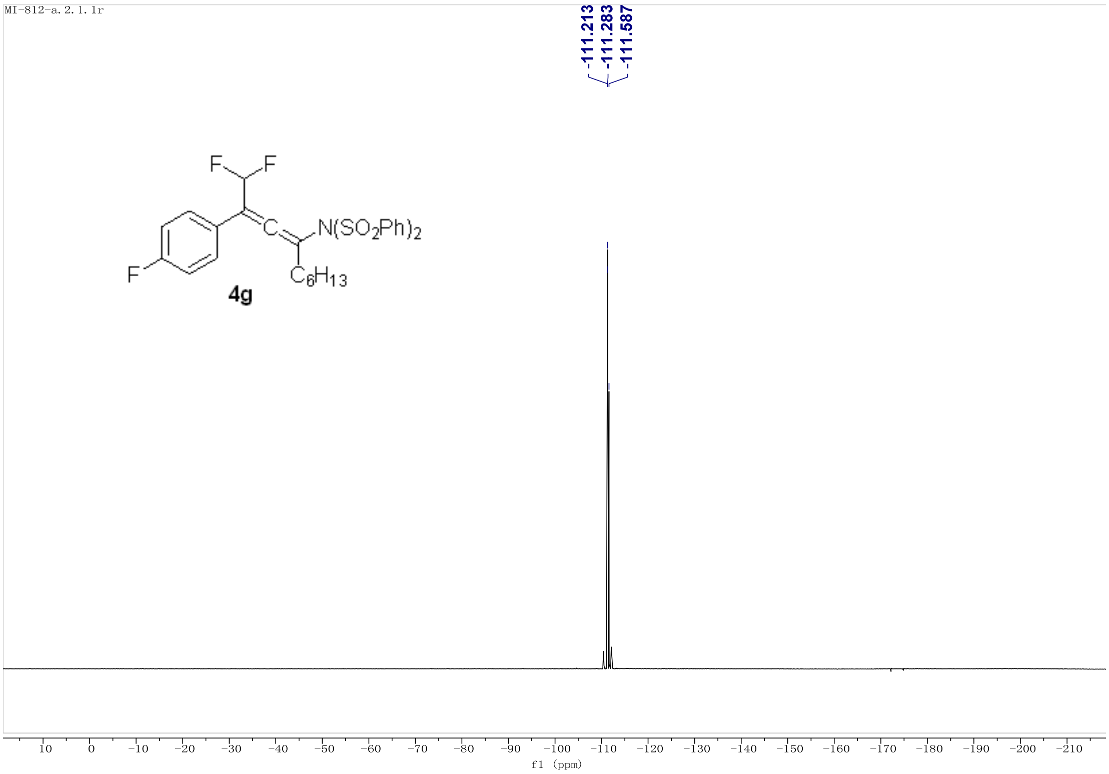

Supplementary Figure 91. ^{19}F **NMR spectrum of compound 4g.**

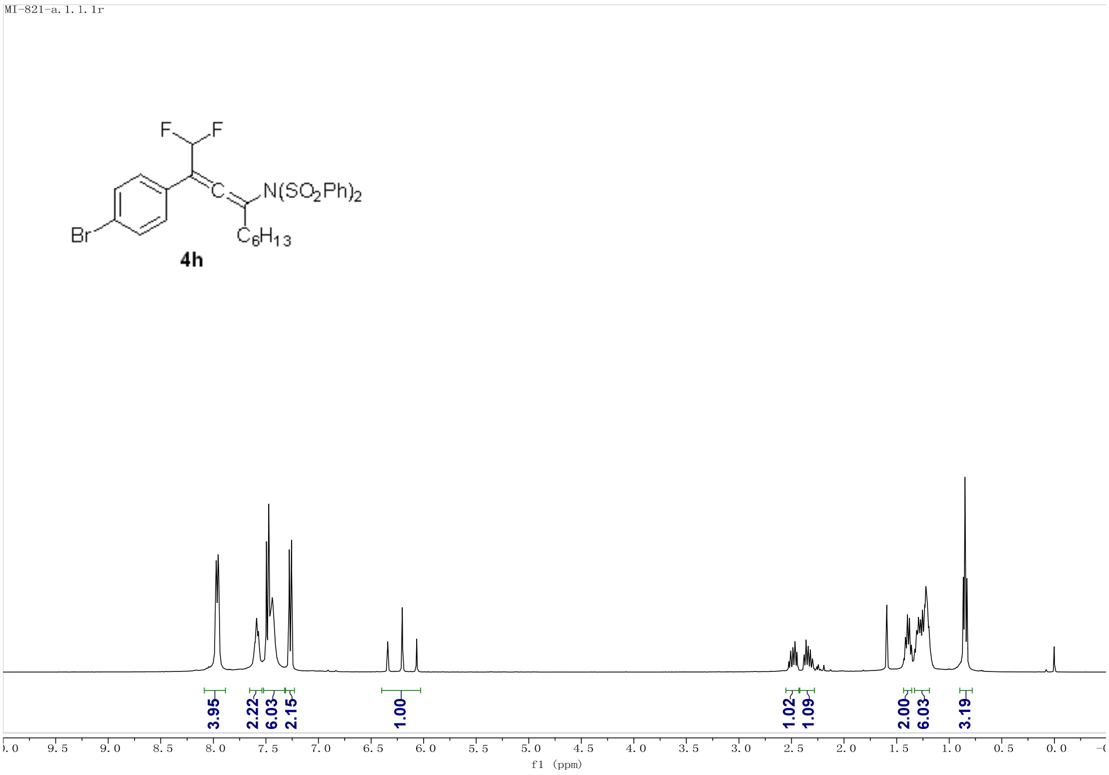

Supplementary Figure 92. ^{1}H NMR spectrum of compound 4h.

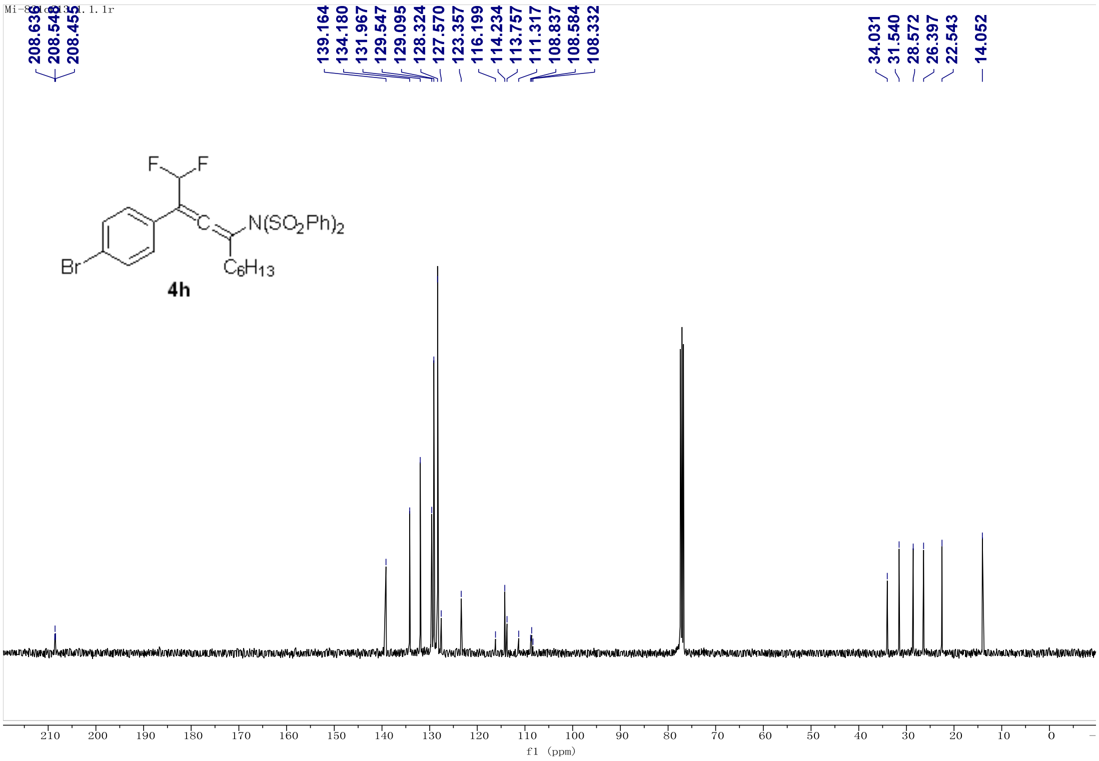

Supplementary Figure 93. ^{13}C NMR spectrum of compound 4h.

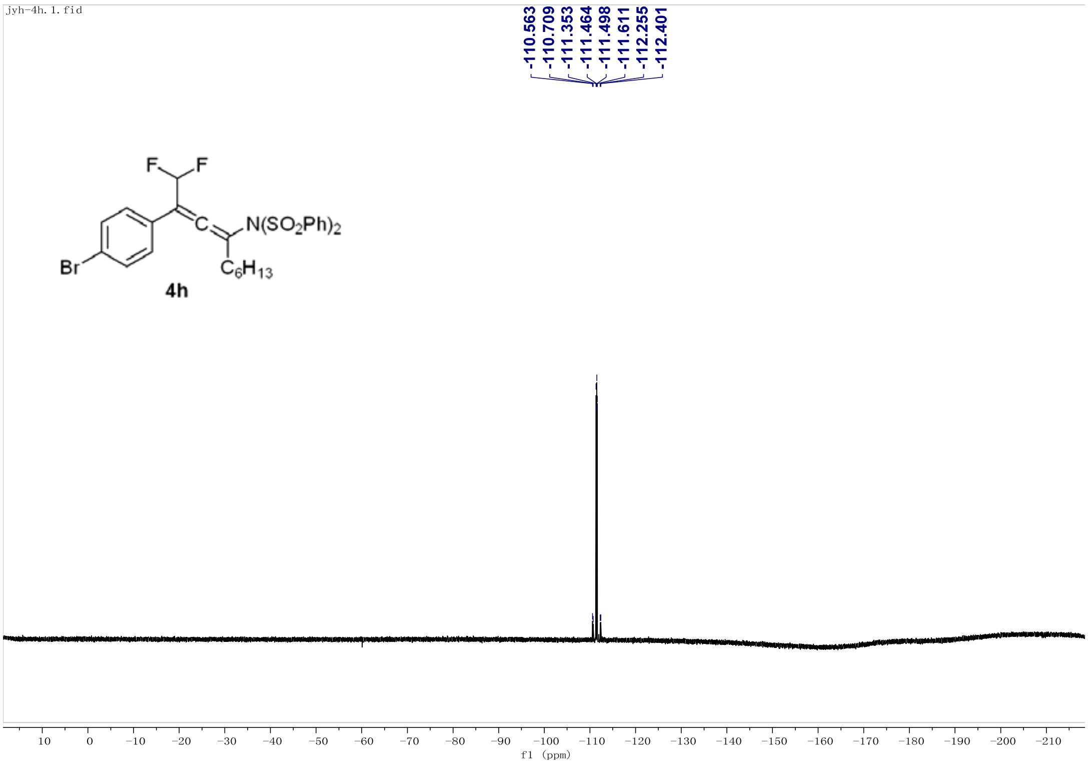

Supplementary Figure 94. ^{19}F NMR spectrum of compound 4h.

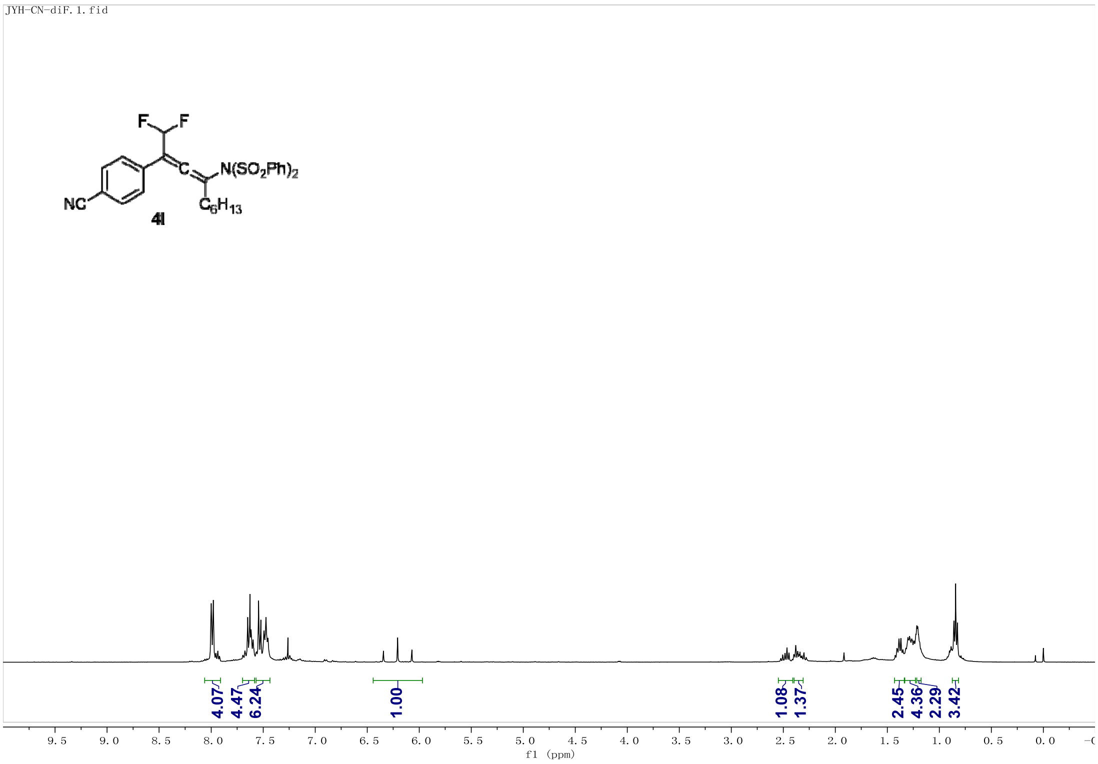

Supplementary Figure 95. ^{1}H NMR spectrum of compound 4i.

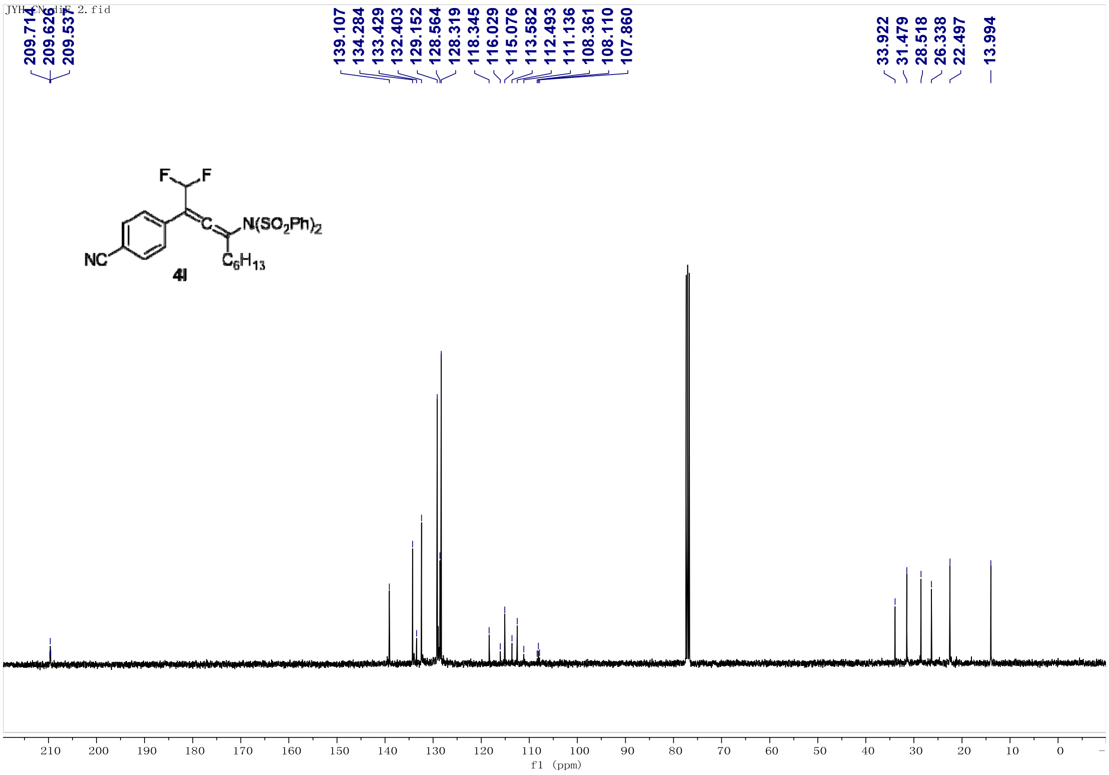

Supplementary Figure 96. ^{13}C NMR spectrum of compound 4i.

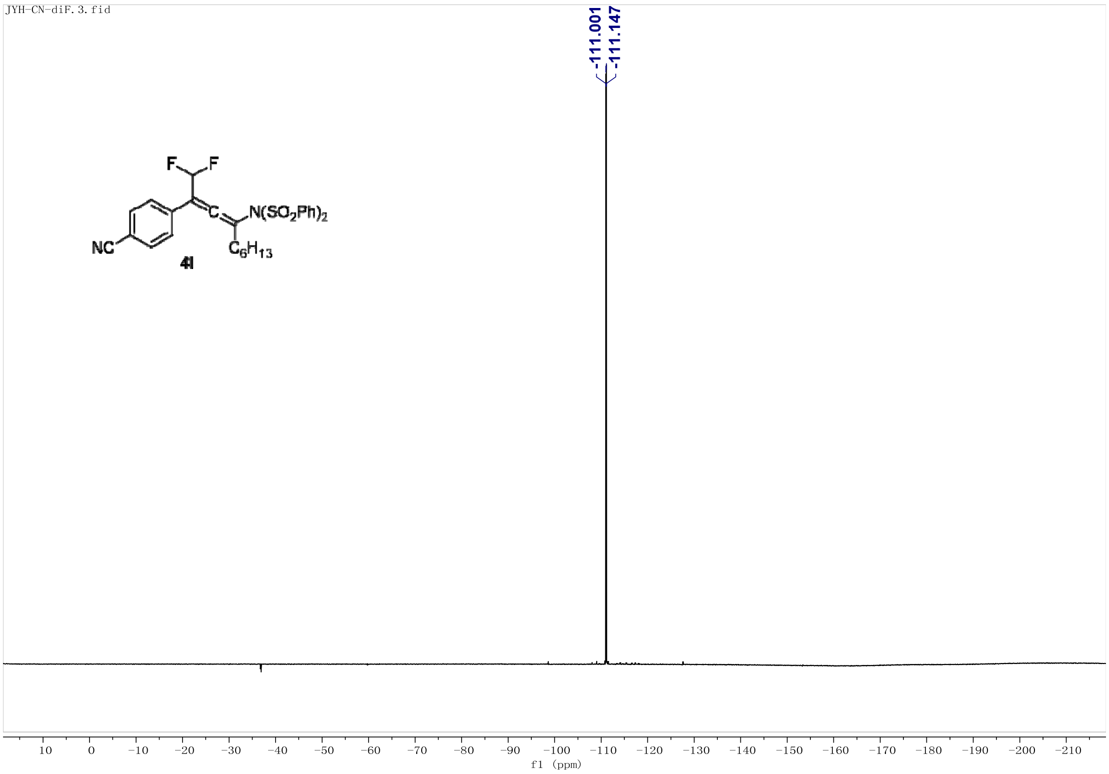

Supplementary Figure 97. ^{19}F NMR spectrum of compound 4i.

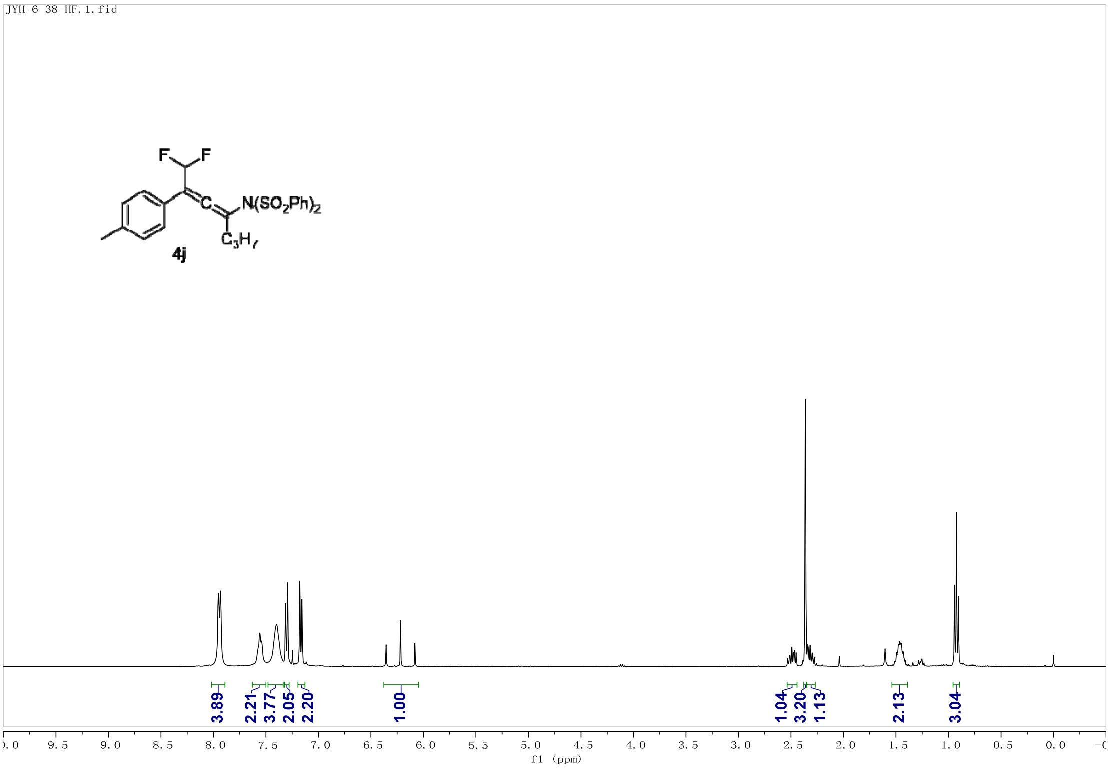

Supplementary Figure 98. ^{1}H NMR spectrum of compound 4j.

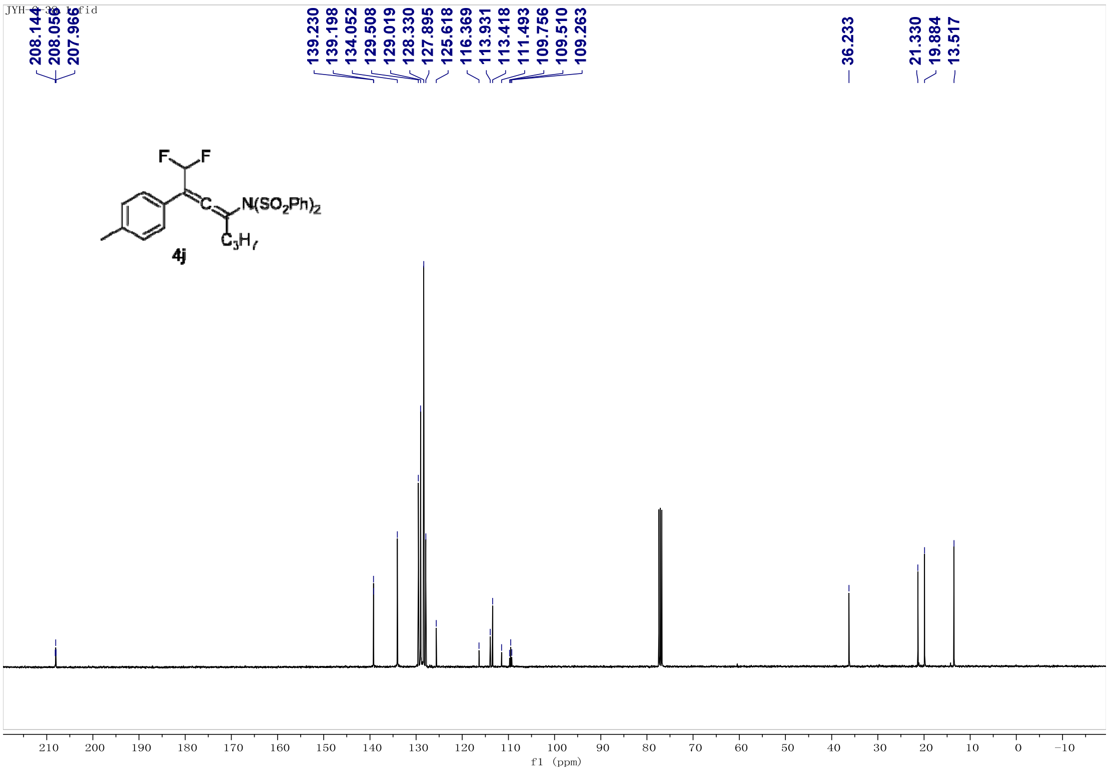

Supplementary Figure 99. ^{13}C NMR spectrum of compound 4j.

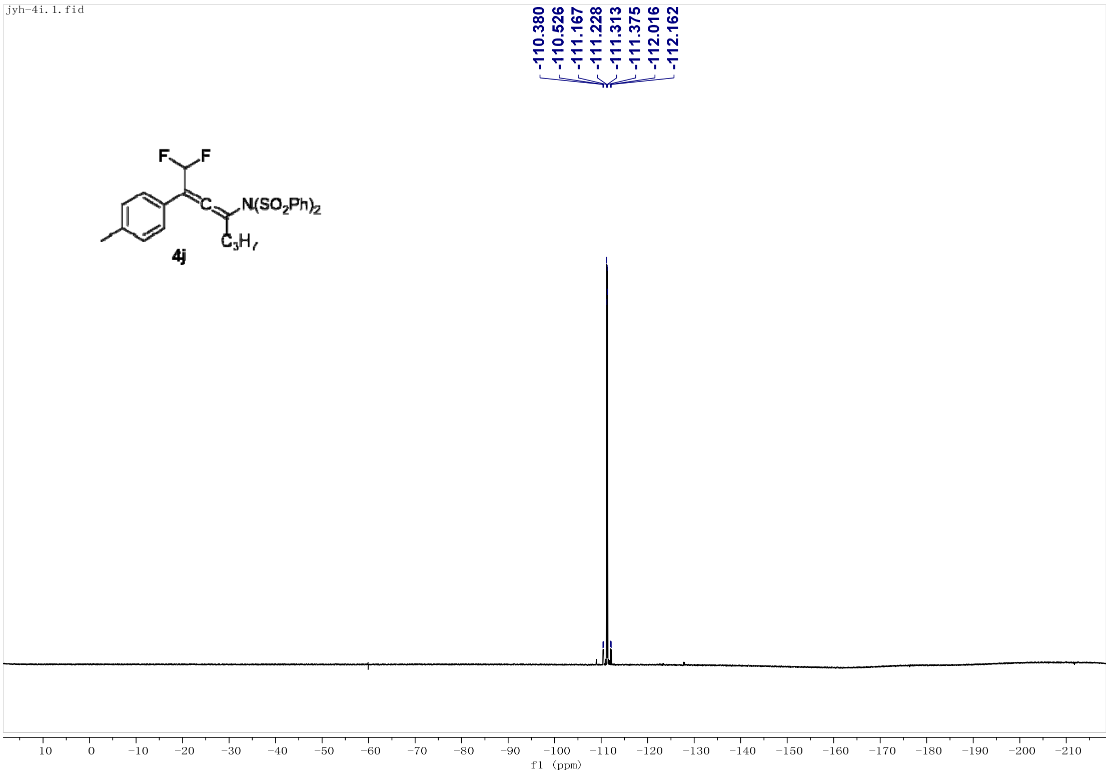

Supplementary Figure 100. ^{19}F **NMR spectrum of compound 4j.**

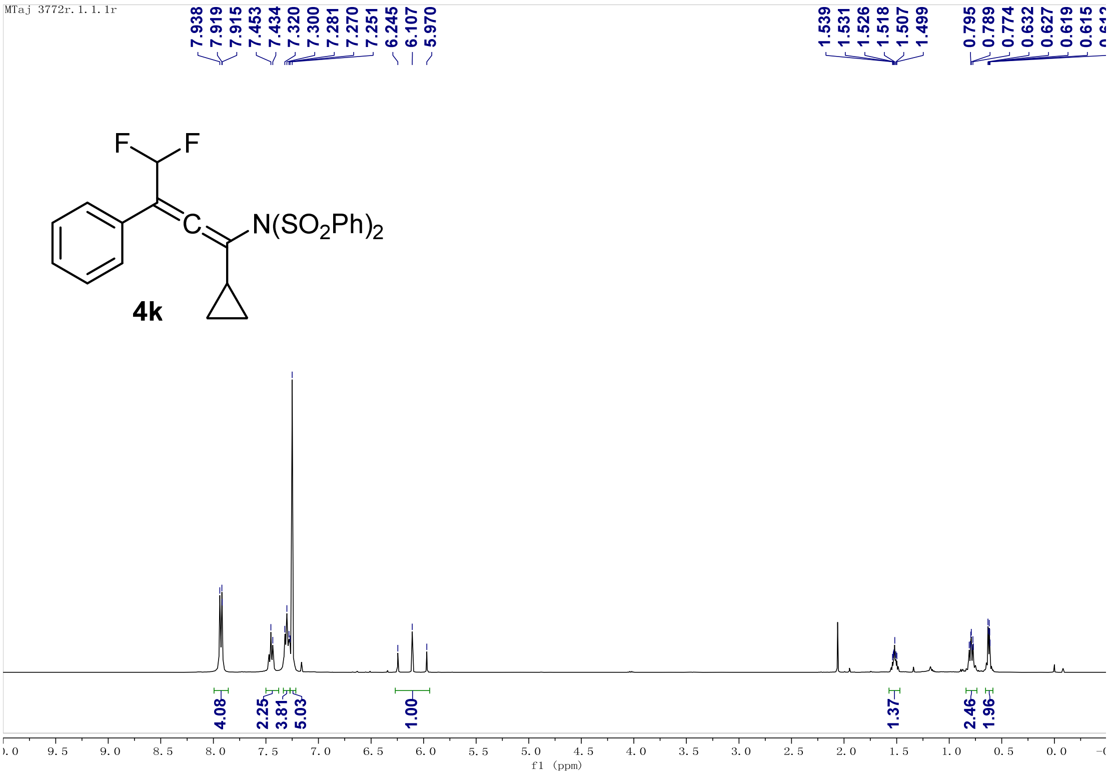

Supplementary Figure 101. ^{1}H **NMR spectrum of compound 4k.**

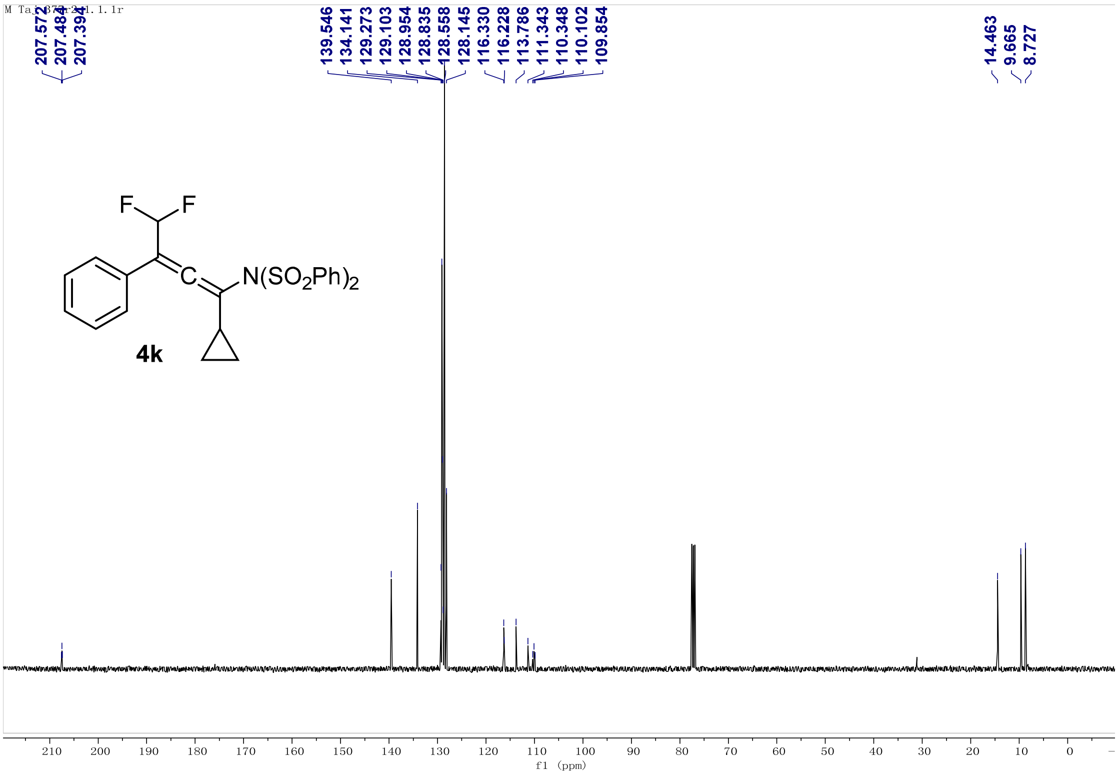

Supplementary Figure 102. **^{13}C NMR spectrum of compound 4k.**

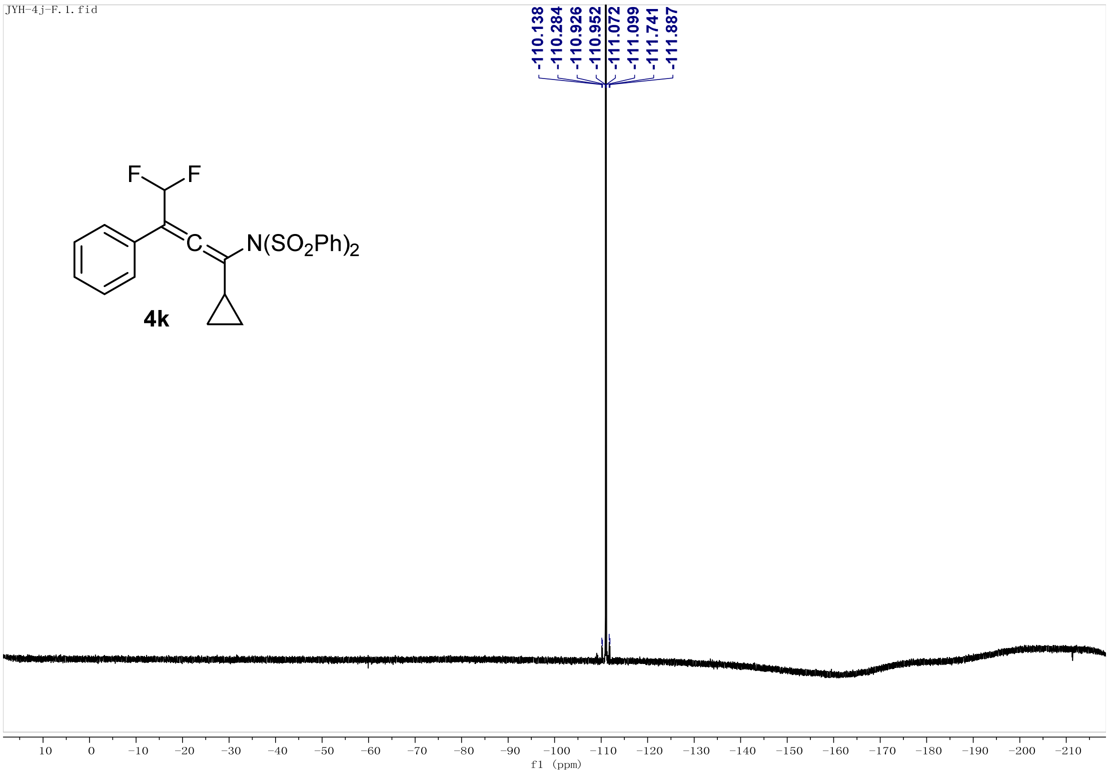

Supplementary Figure 103. **^{19}F NMR spectrum of compound 4k.**

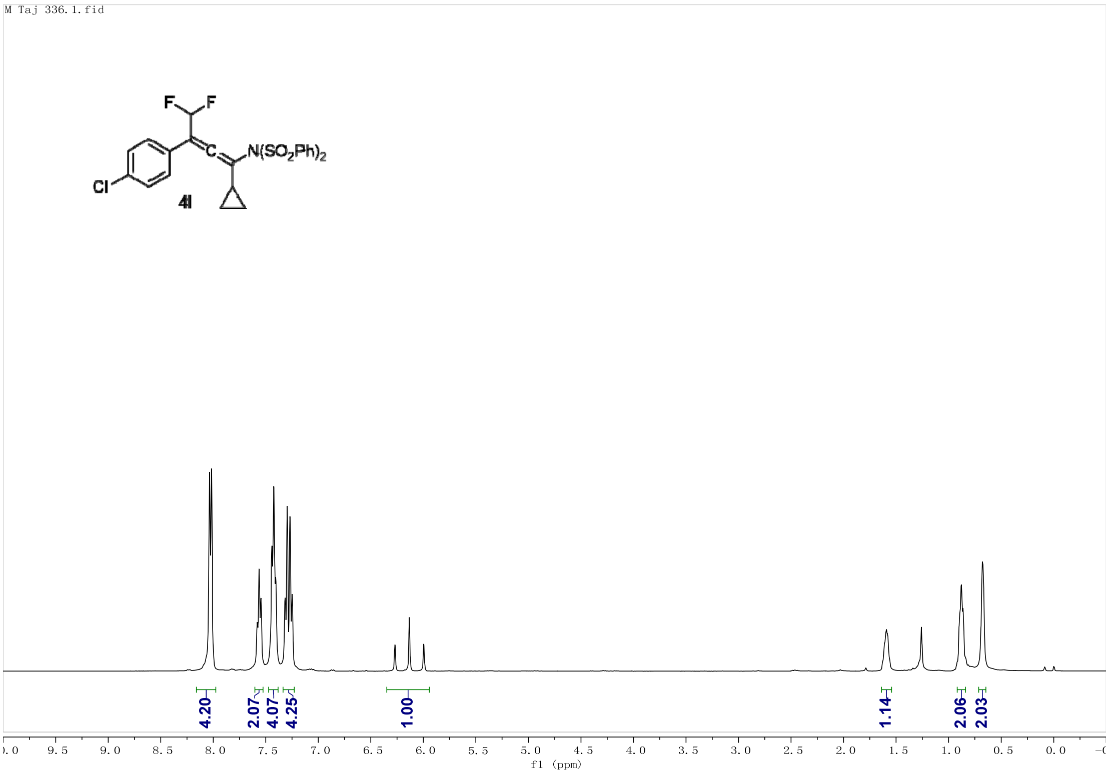

Supplementary Figure 104. ^{1}H NMR spectrum of compound 4l.

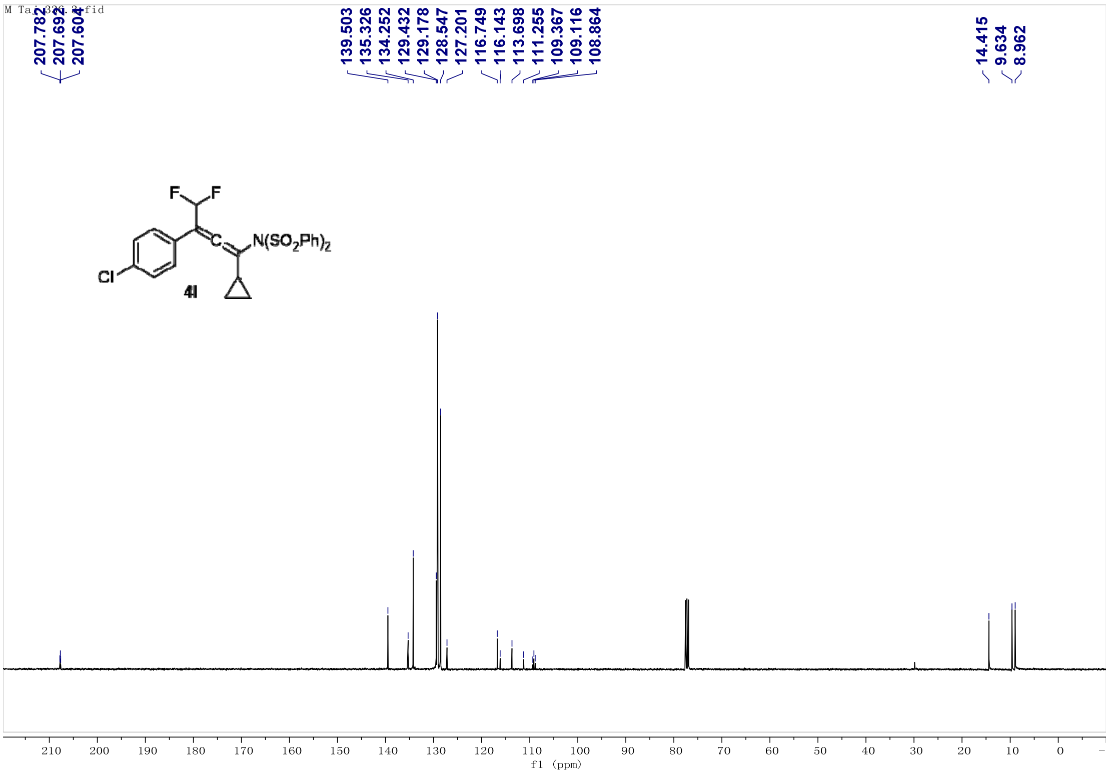

Supplementary Figure 105. ^{13}C NMR spectrum of compound 4l.

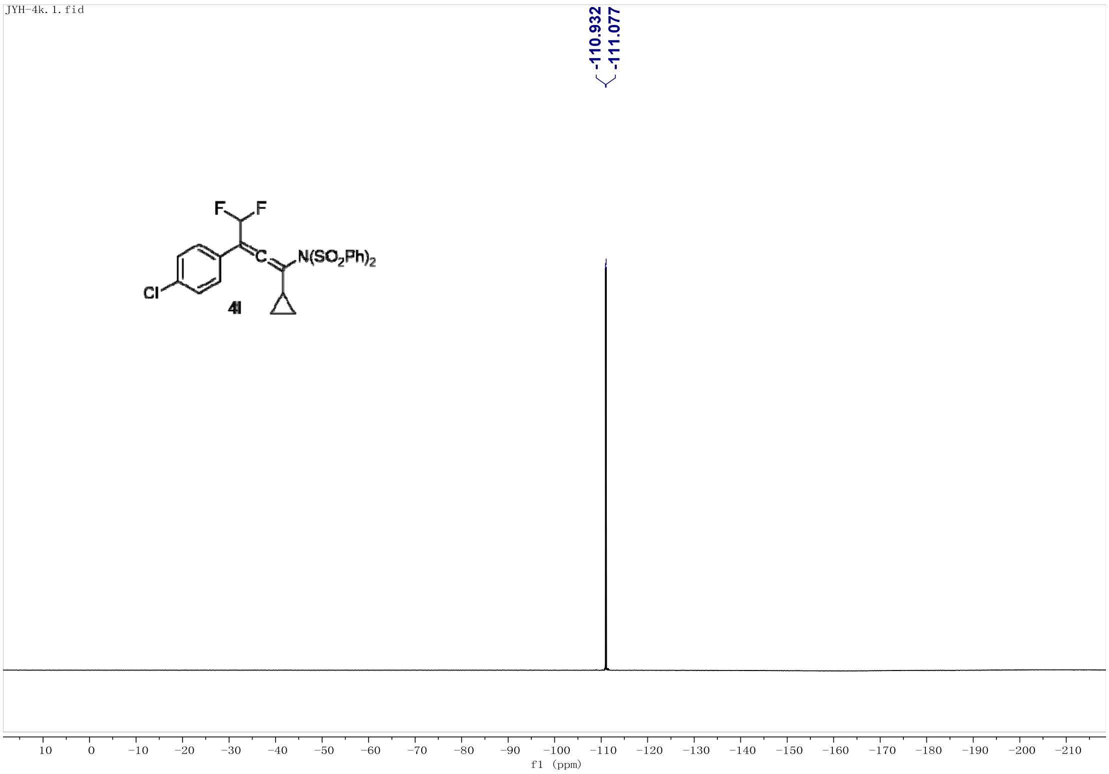

Supplementary Figure 106. ^{19}F NMR spectrum of compound 4l.

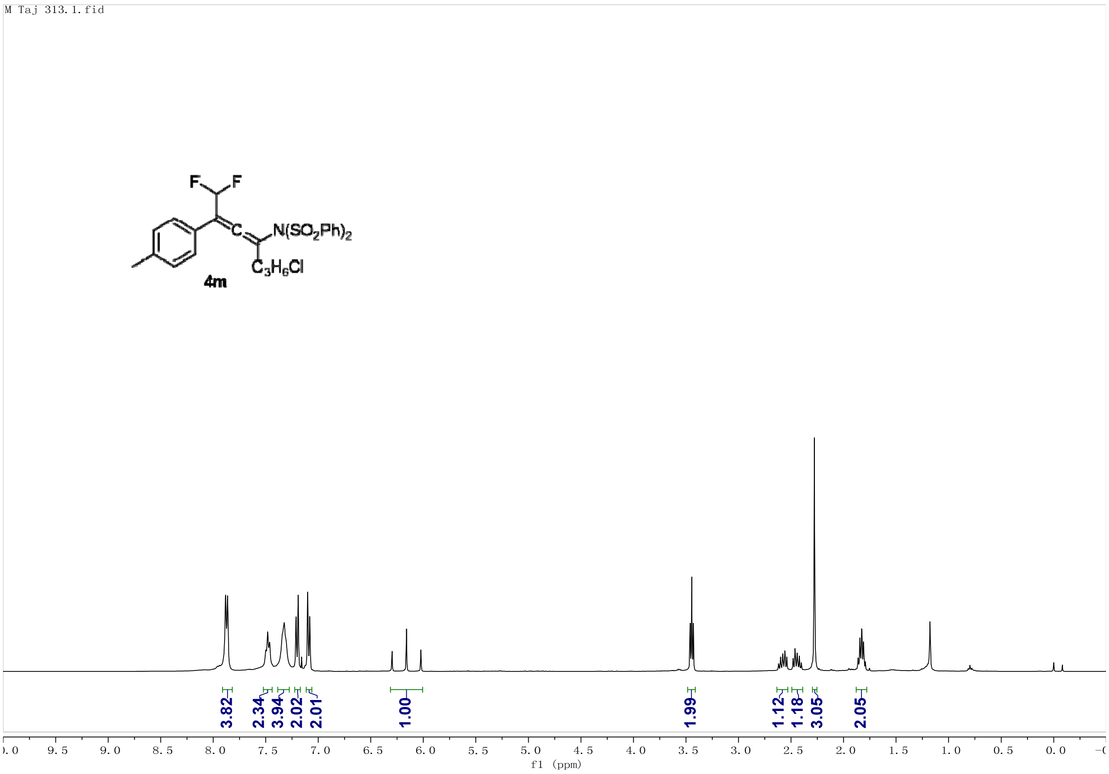

Supplementary Figure 107. ^{1}H NMR spectrum of compound 4m.

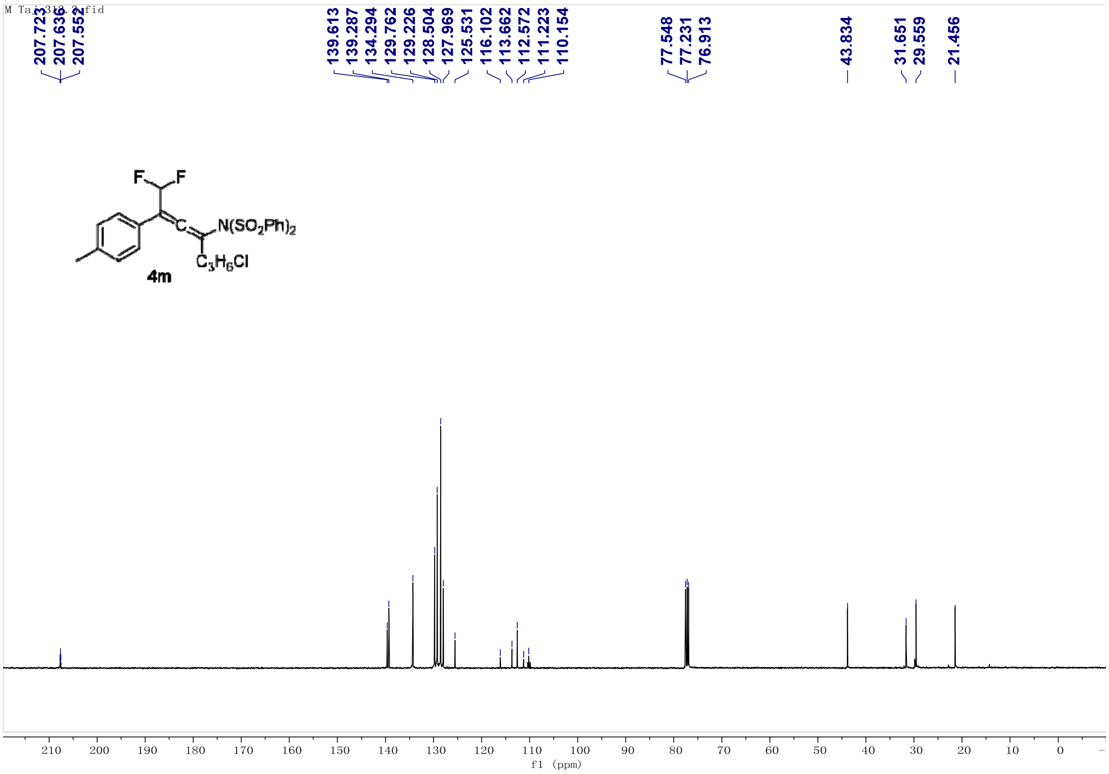

Supplementary Figure 108. ^{13}C **NMR spectrum of compound 4m.**

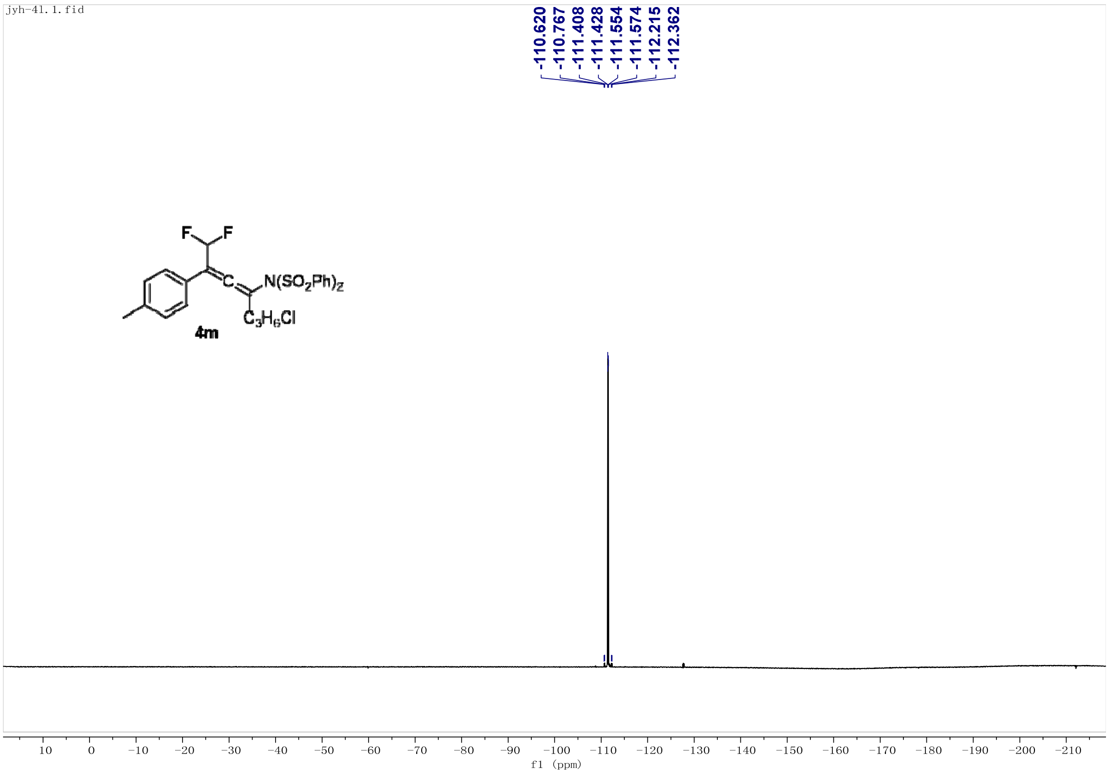

Supplementary Figure 109. ^{19}F **NMR spectrum of compound 4m.**

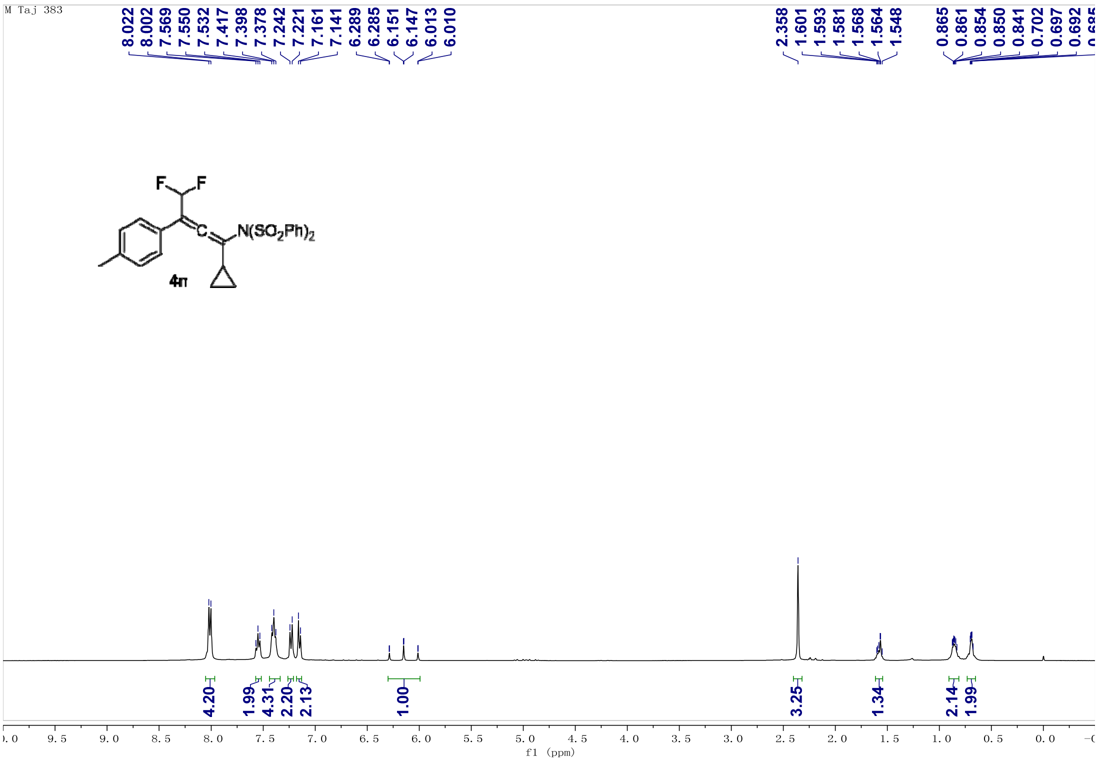

Supplementary Figure 110. ^1H NMR spectrum of compound 4n.

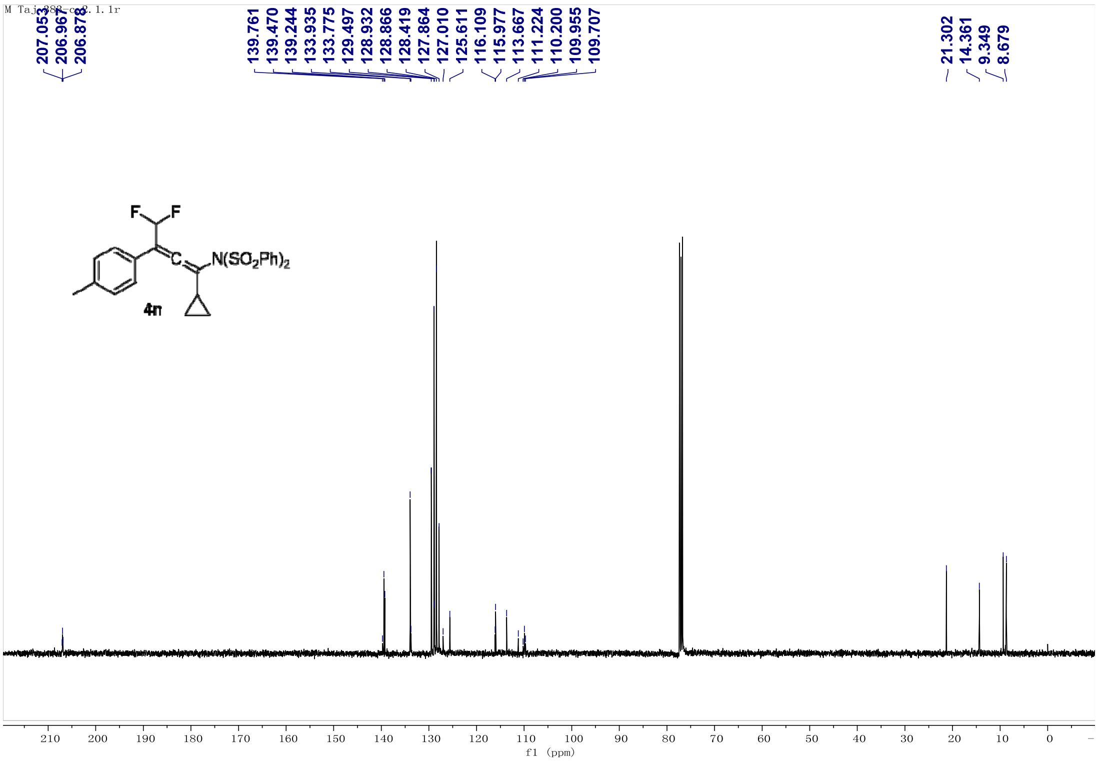

Supplementary Figure 111. ^{13}C NMR spectrum of compound 4n.

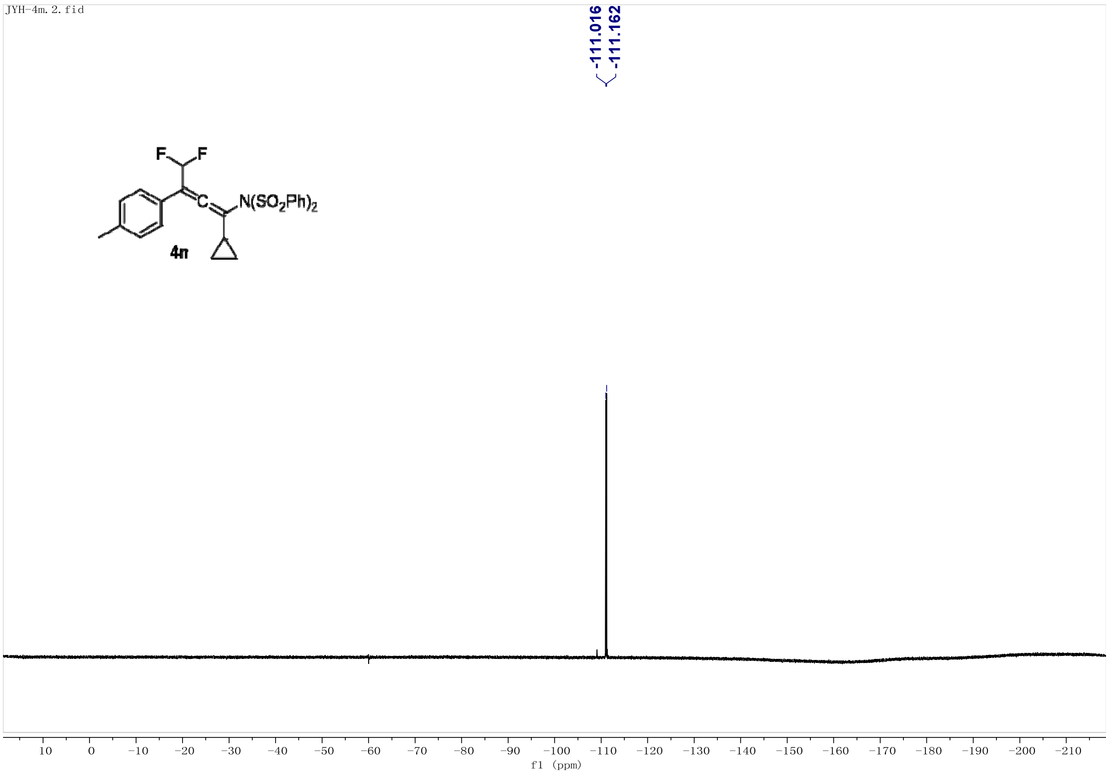

Supplementary Figure 112. **^{19}F NMR spectrum of compound 4n.**

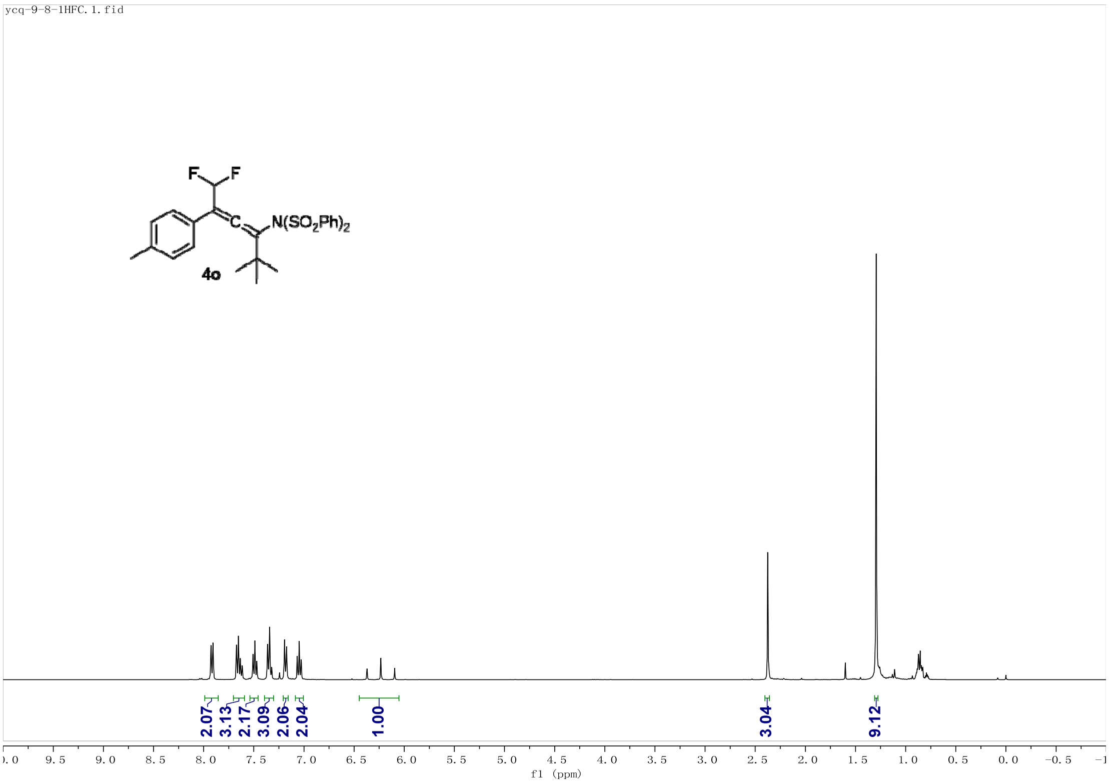

Supplementary Figure 113. **^{1}H NMR spectrum of compound 4o.**

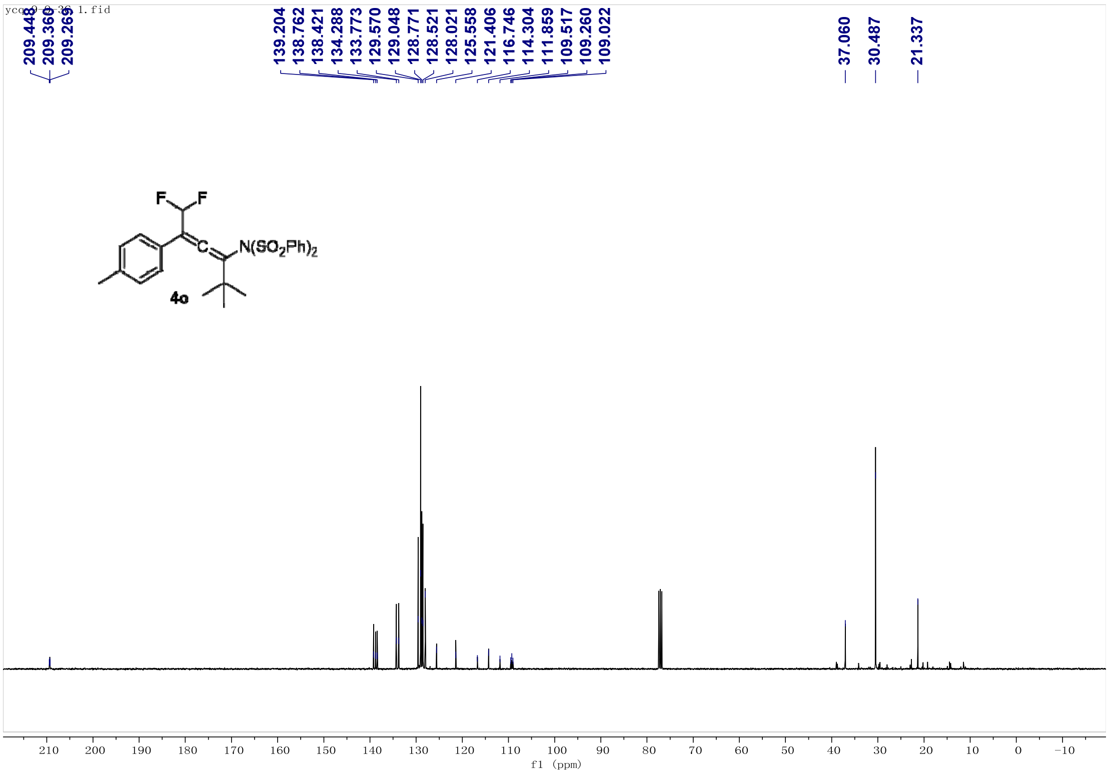

Supplementary Figure 114. **^{13}C NMR spectrum of compound 4o.**

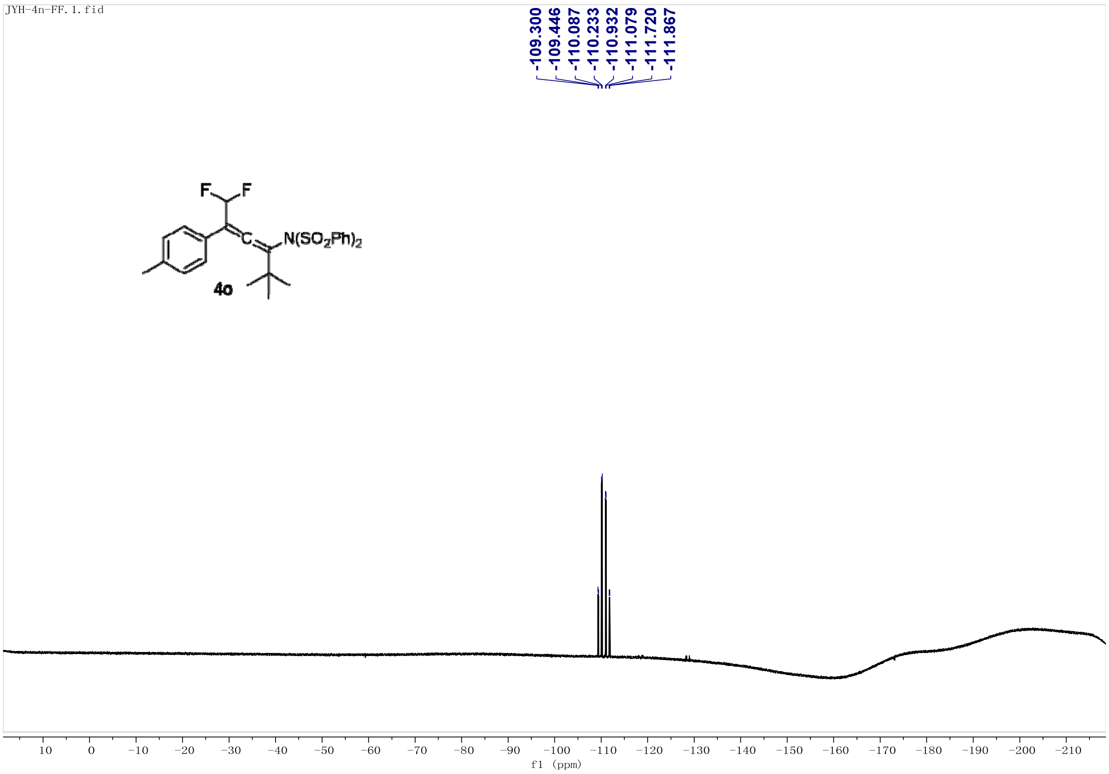

Supplementary Figure 115. **^{19}F NMR spectrum of compound 4o.**

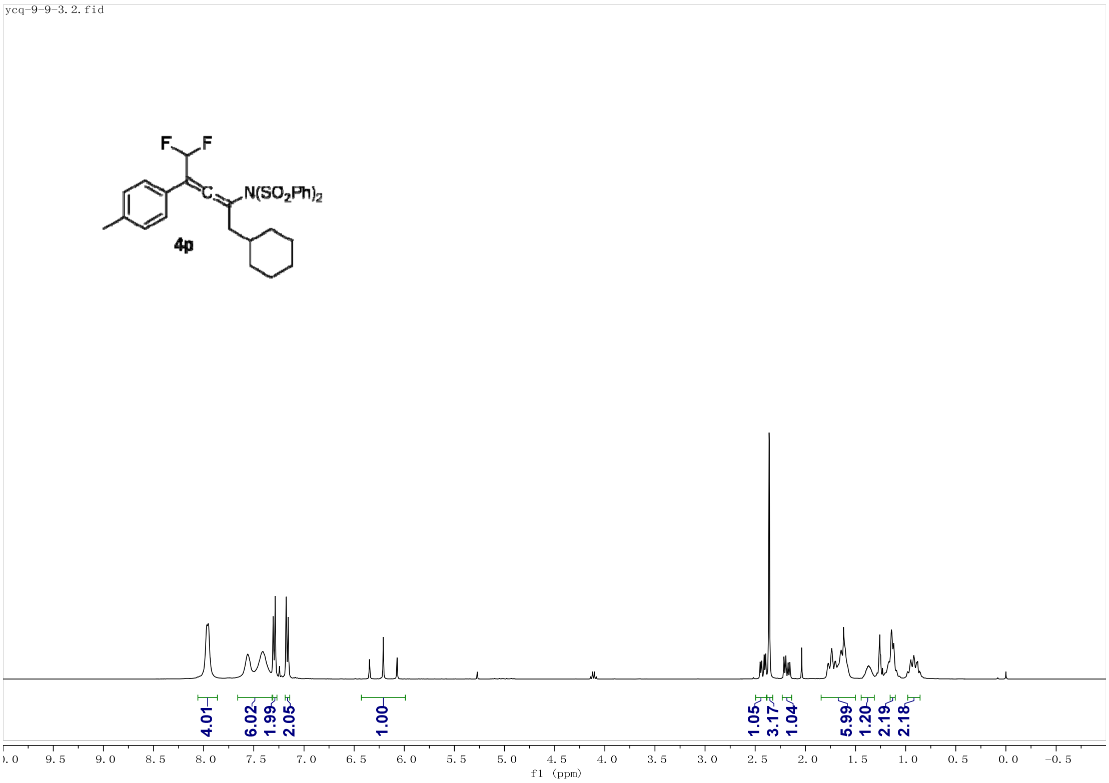

Supplementary Figure 116. ^{1}H NMR spectrum of compound 4p.

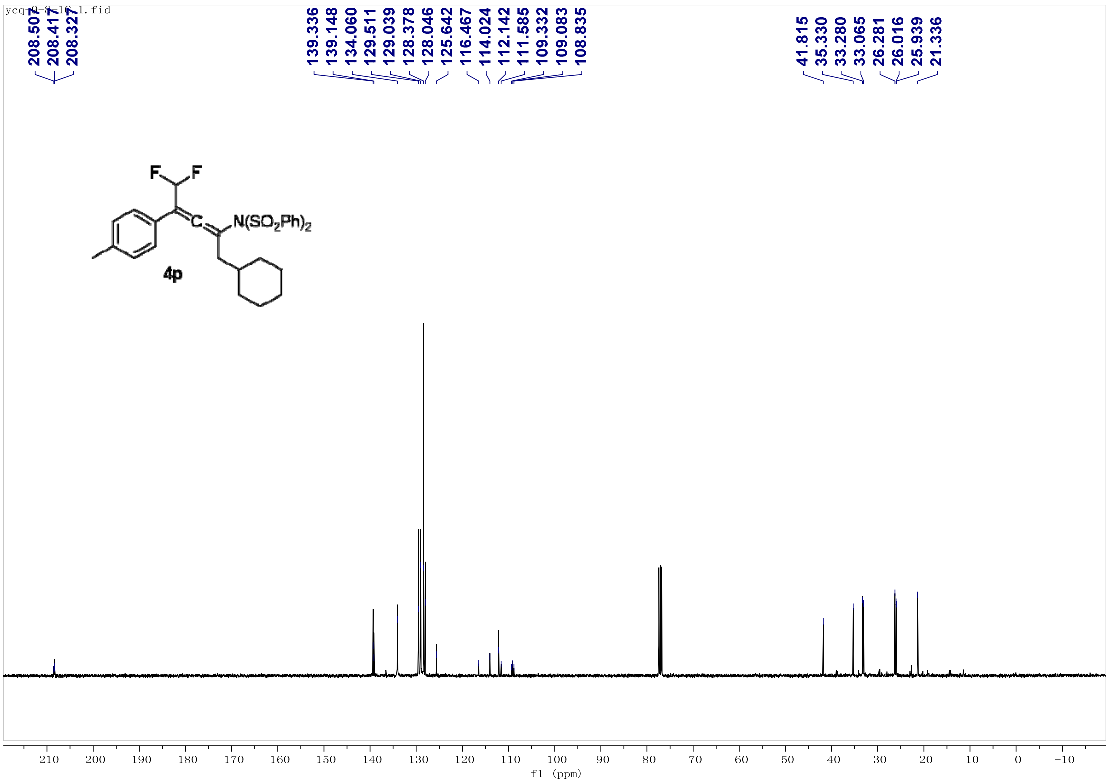

Supplementary Figure 117. ^{13}C NMR spectrum of compound 4p.

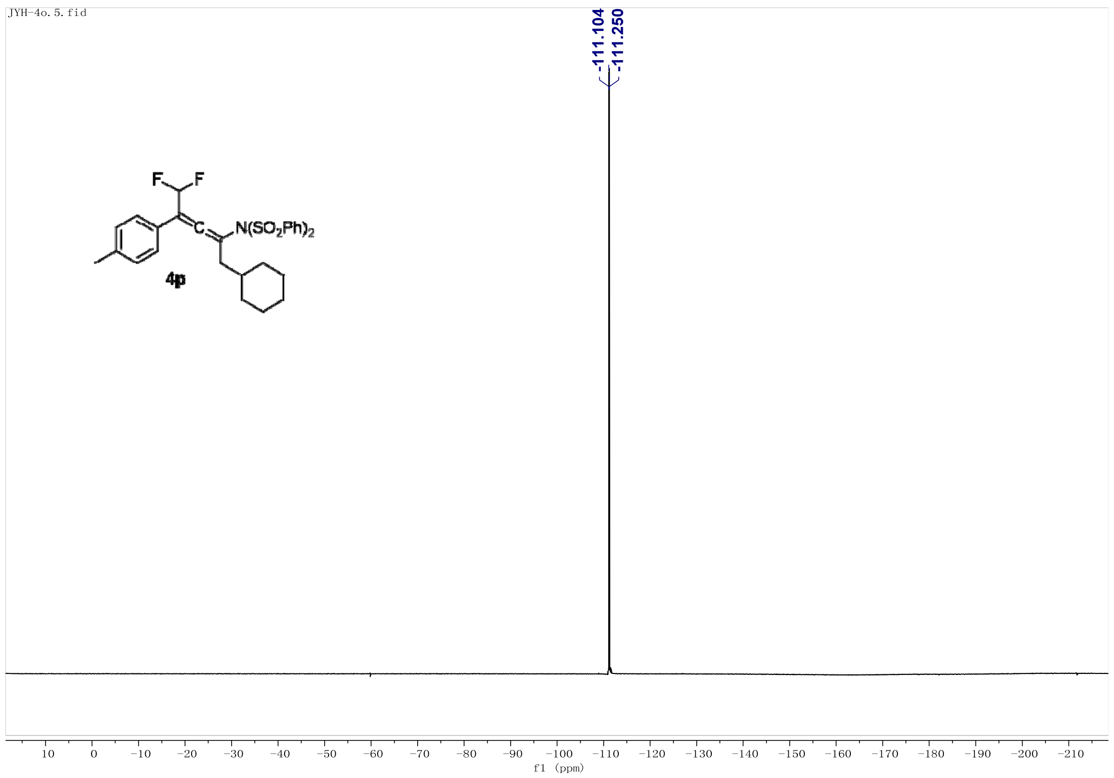

Supplementary Figure 118. ^{19}F **NMR spectrum of compound 4p.**

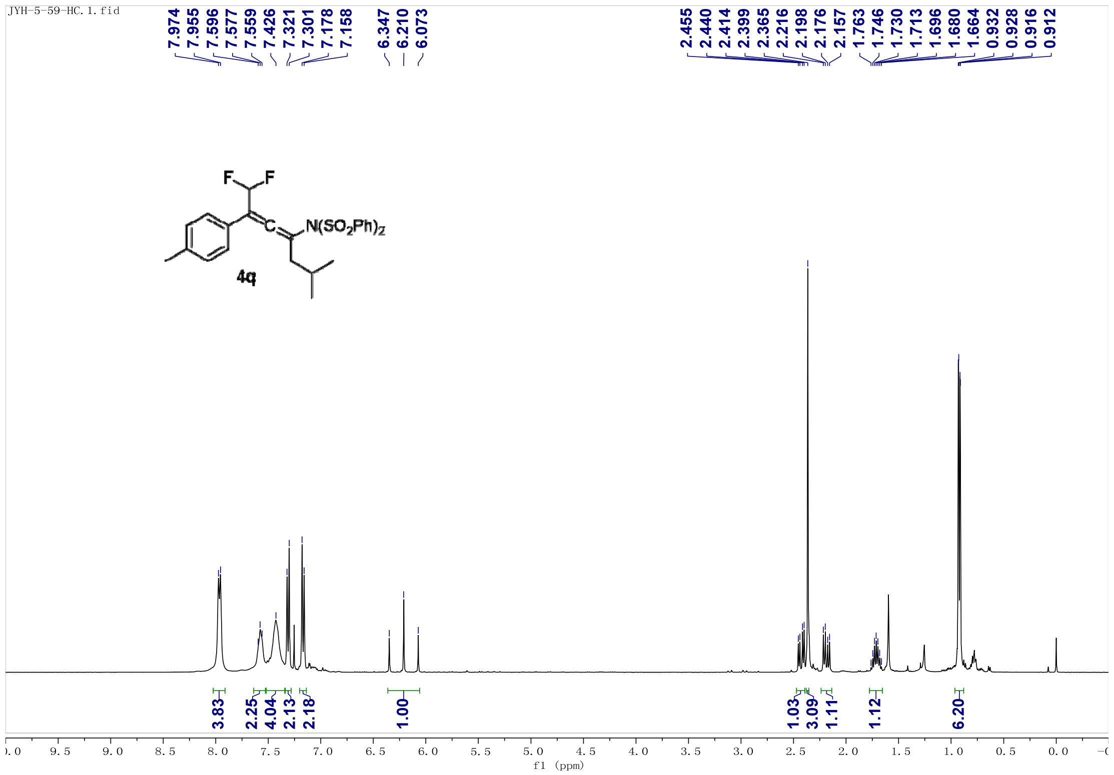

Supplementary Figure 119. ^{1}H **NMR spectrum of compound 4q.**

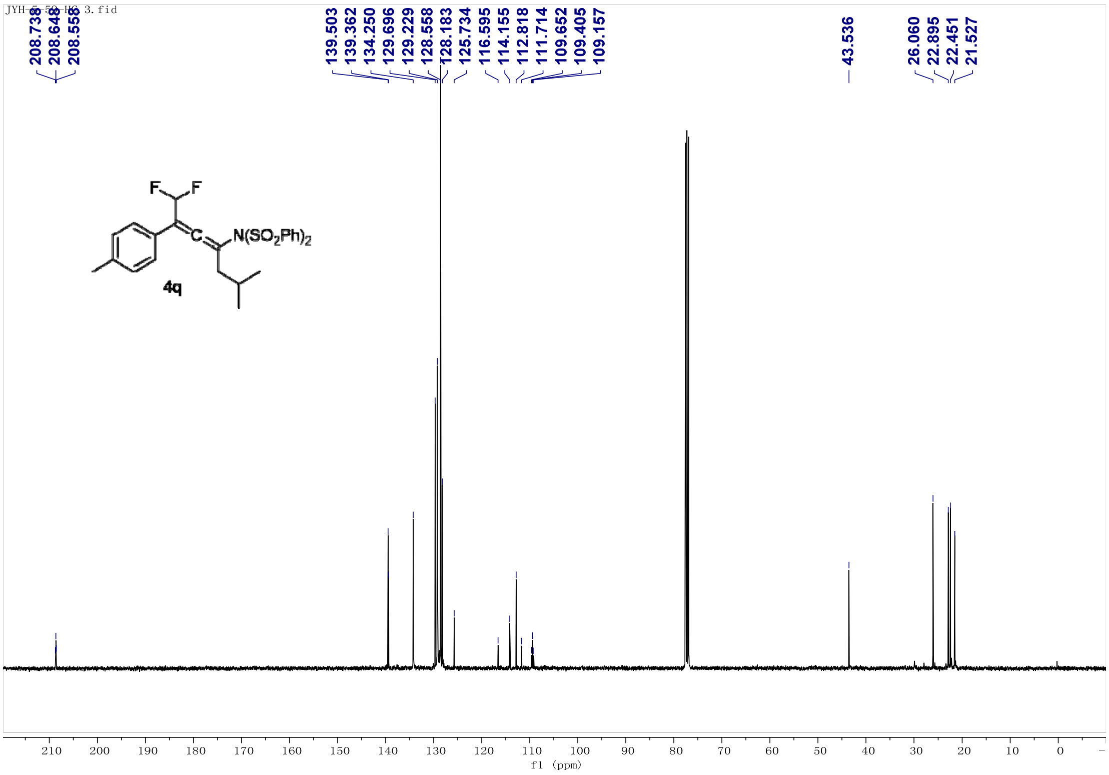

Supplementary Figure 120. ^{13}C **NMR spectrum of compound 4q.**

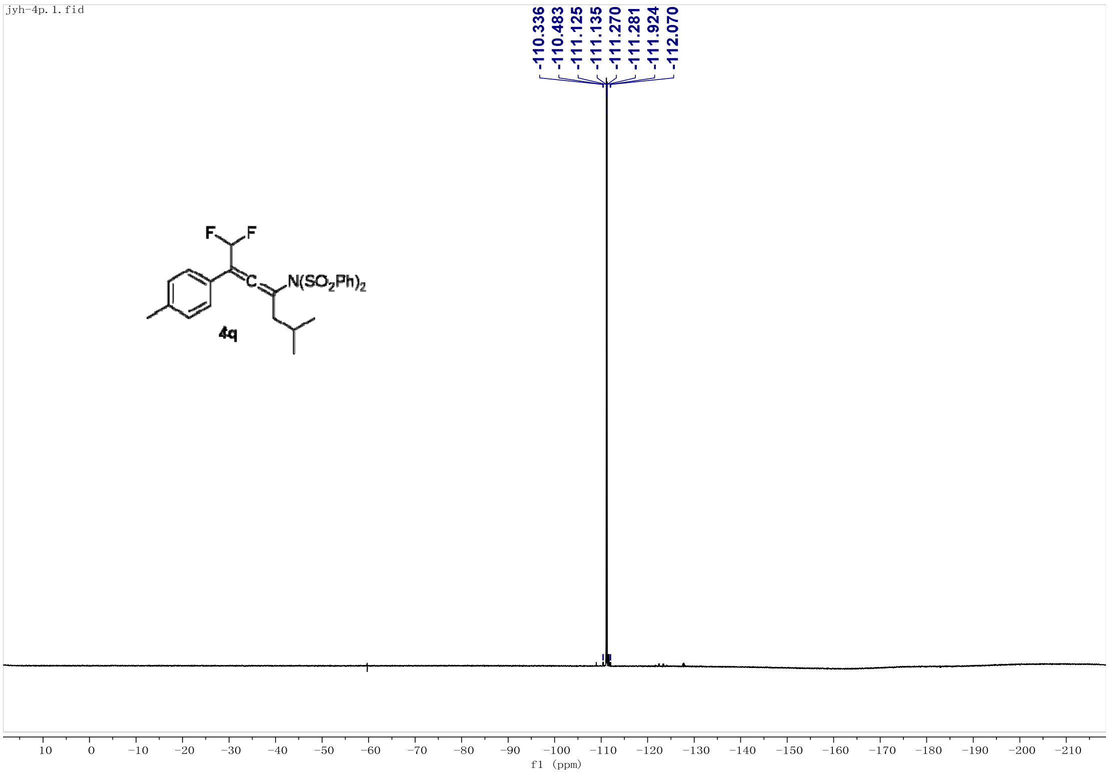

Supplementary Figure 121. ^{19}F **NMR spectrum of compound 4q.**

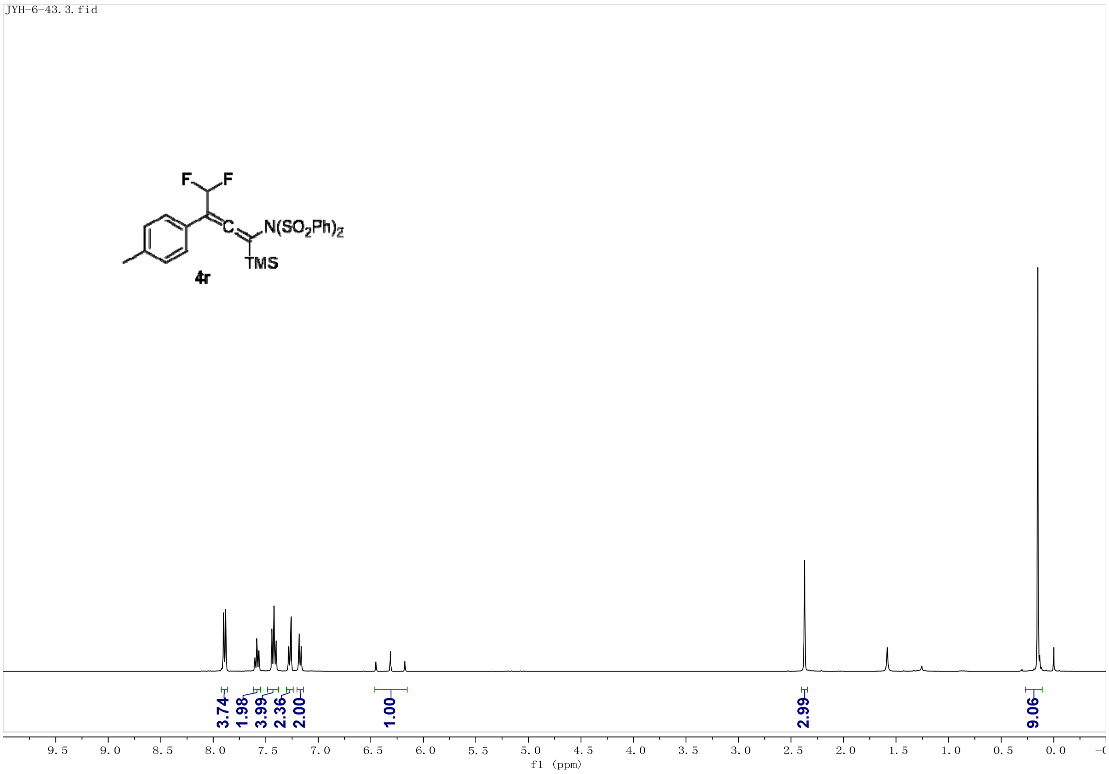

Supplementary Figure 122. **^{1}H NMR spectrum of compound 4r.**

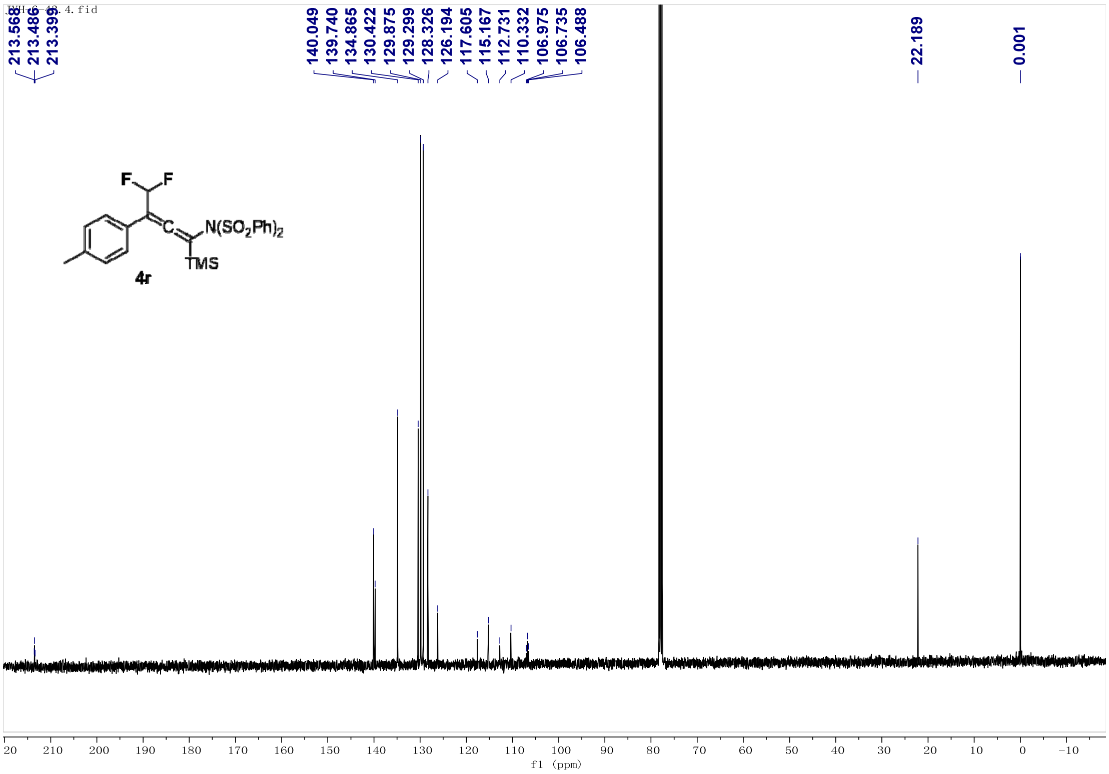

Supplementary Figure 123. ^{13}C NMR spectrum of compound 4r.

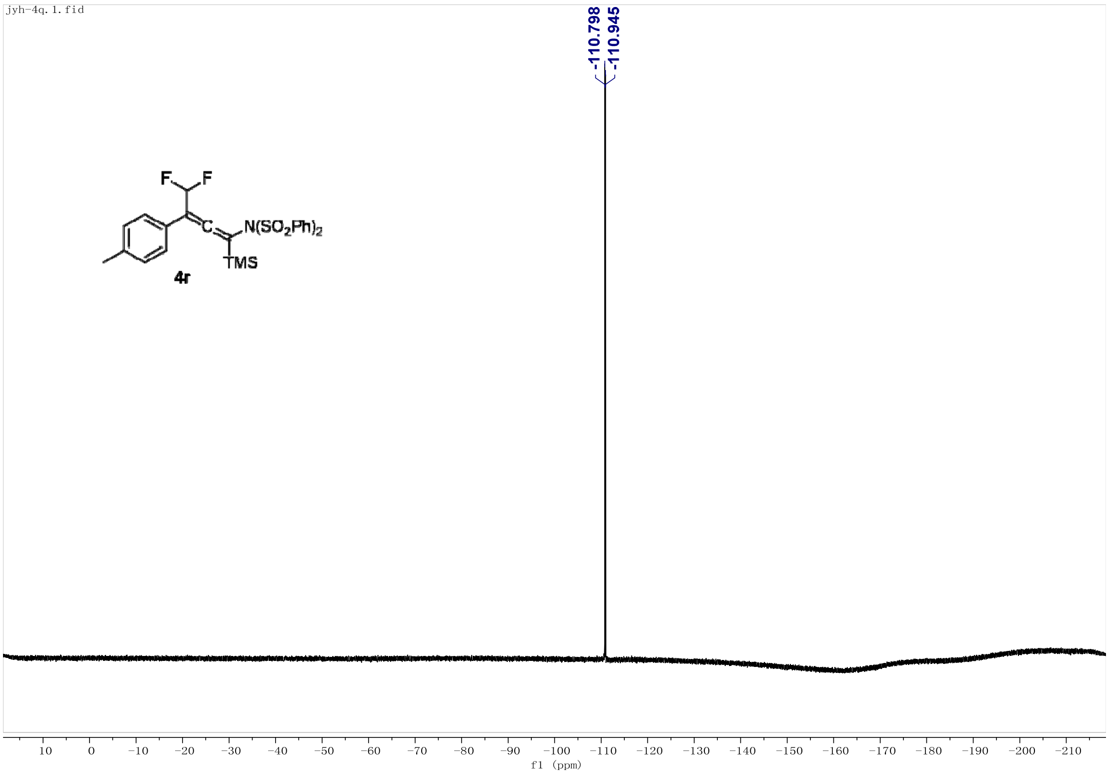

Supplementary Figure 124. ^{19}F NMR spectrum of compound 4r.

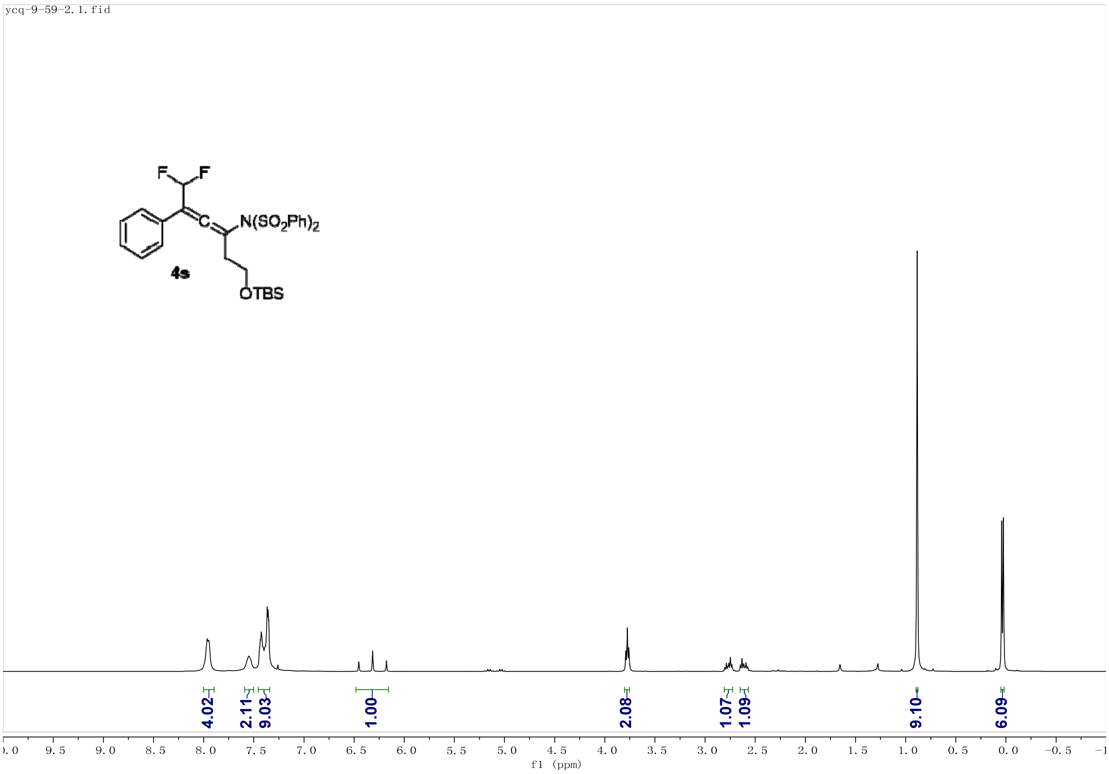

Supplementary Figure 125. ^{1}H NMR spectrum of compound 4s.

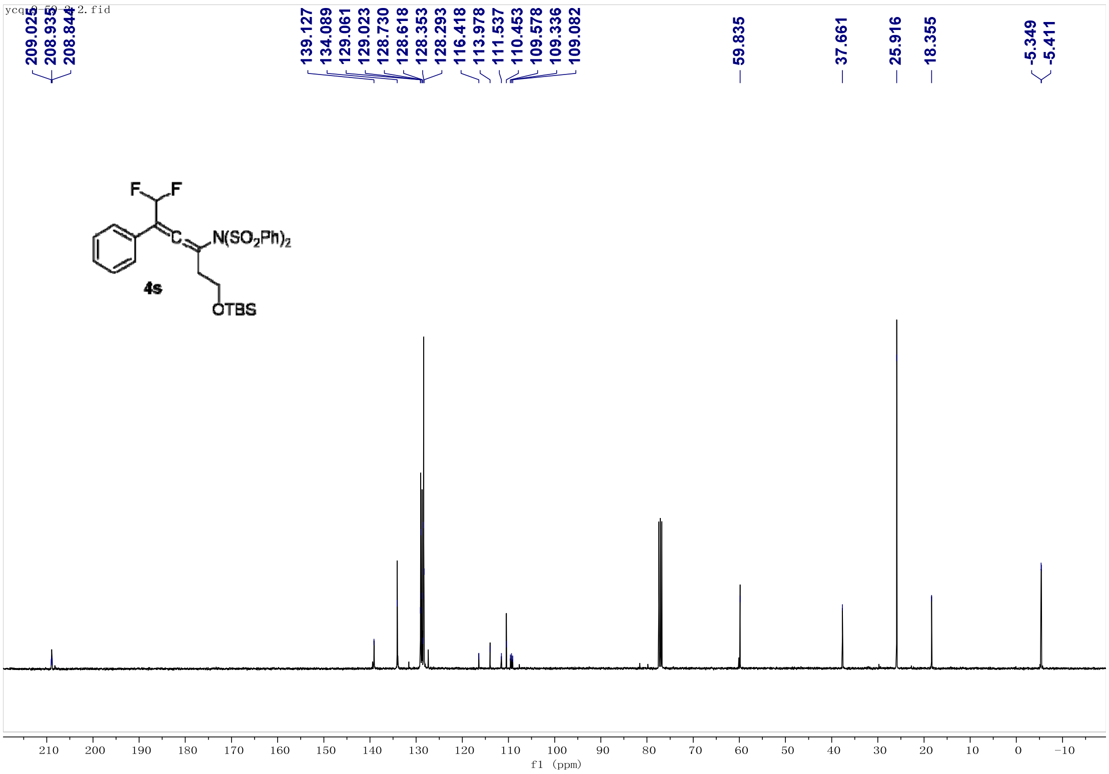

Supplementary Figure 126. ^{13}C NMR spectrum of compound 4s.

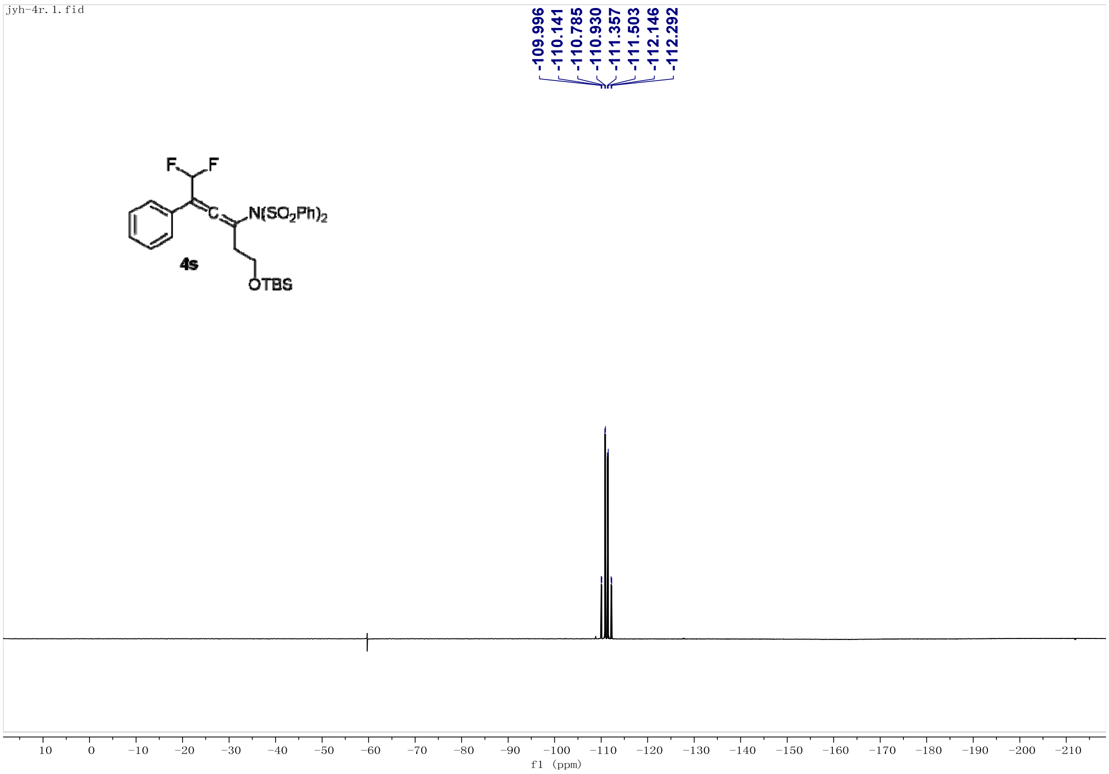

Supplementary Figure 127. ^{19}F NMR spectrum of compound 4s.

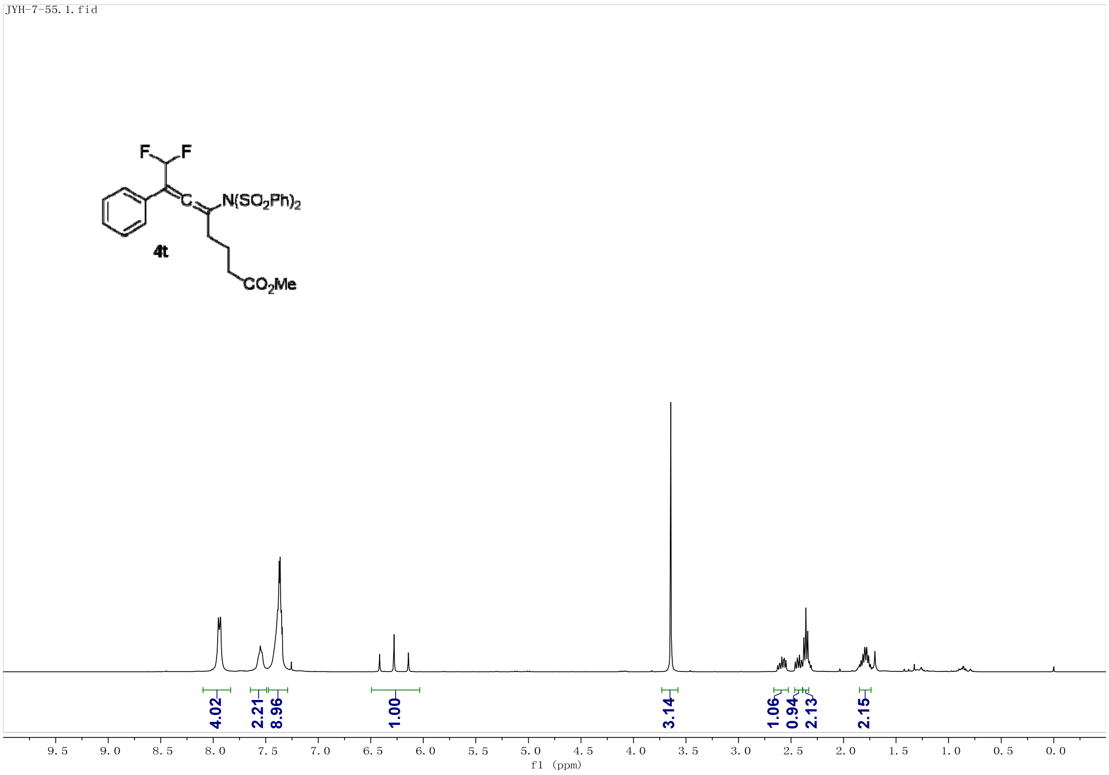

Supplementary Figure 128. ^{1}H NMR spectrum of compound 4t.

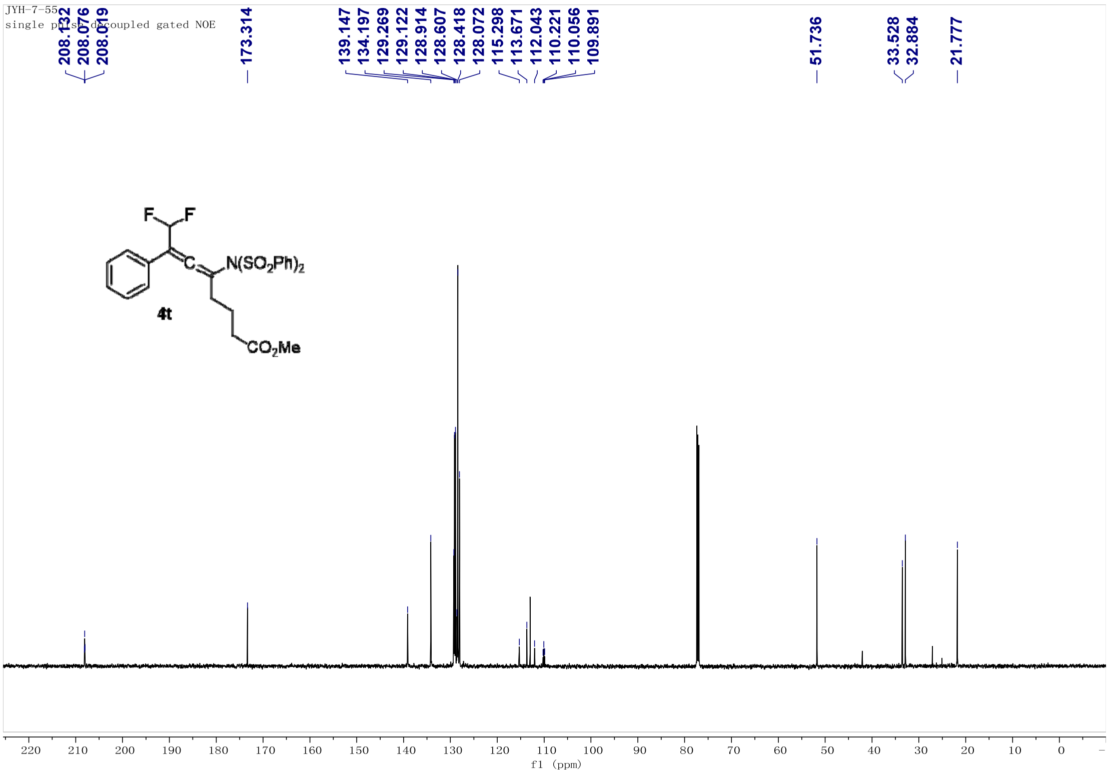

Supplementary Figure 129. ^{13}C NMR spectrum of compound 4t.

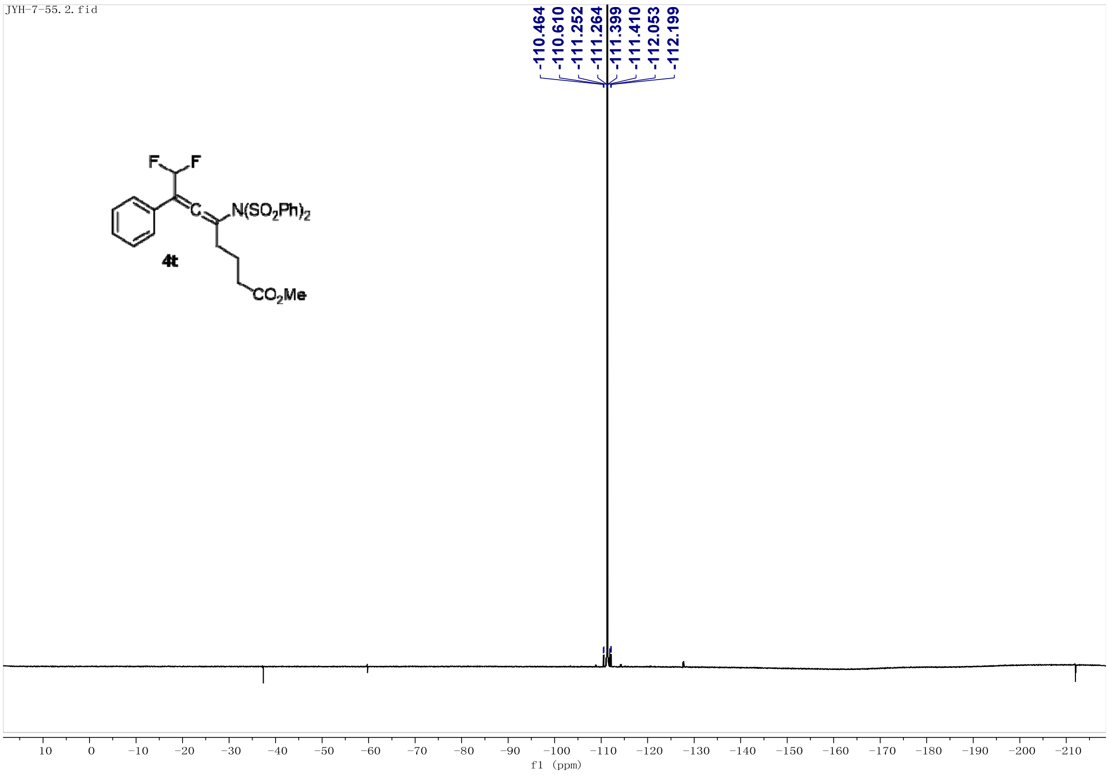

Supplementary Figure 130. **^{19}F NMR spectrum of compound 4t.**

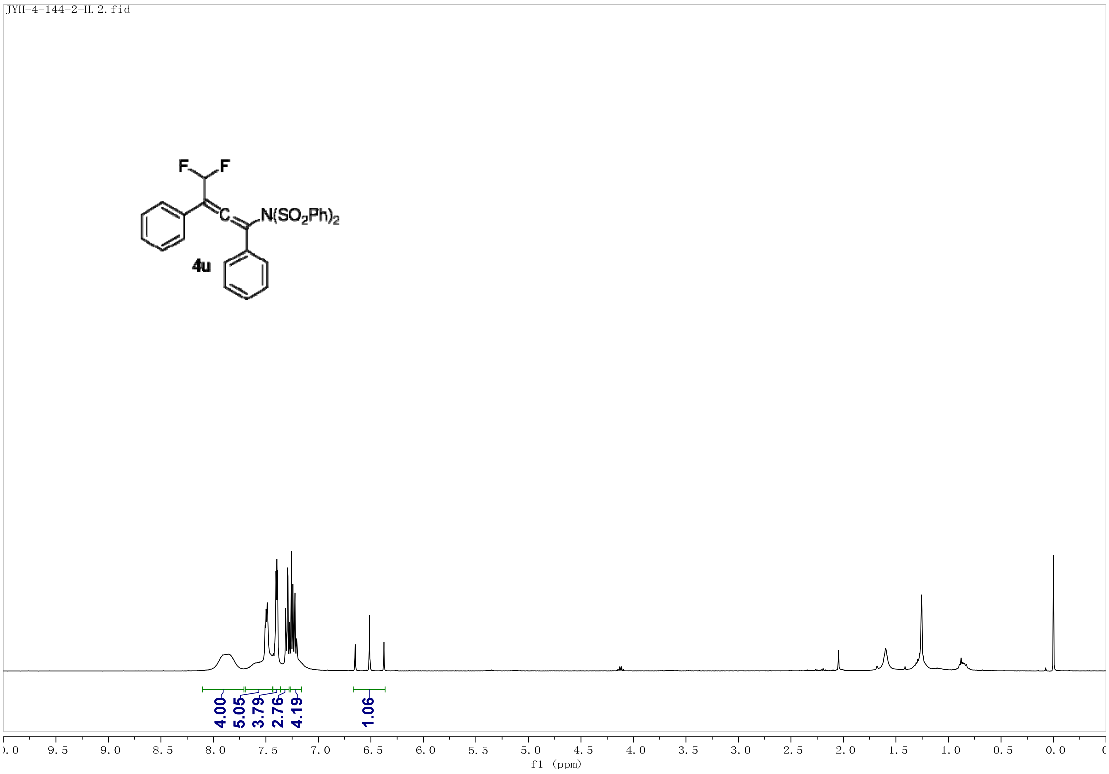

Supplementary Figure 131. **^{1}H NMR spectrum of compound 4u.**

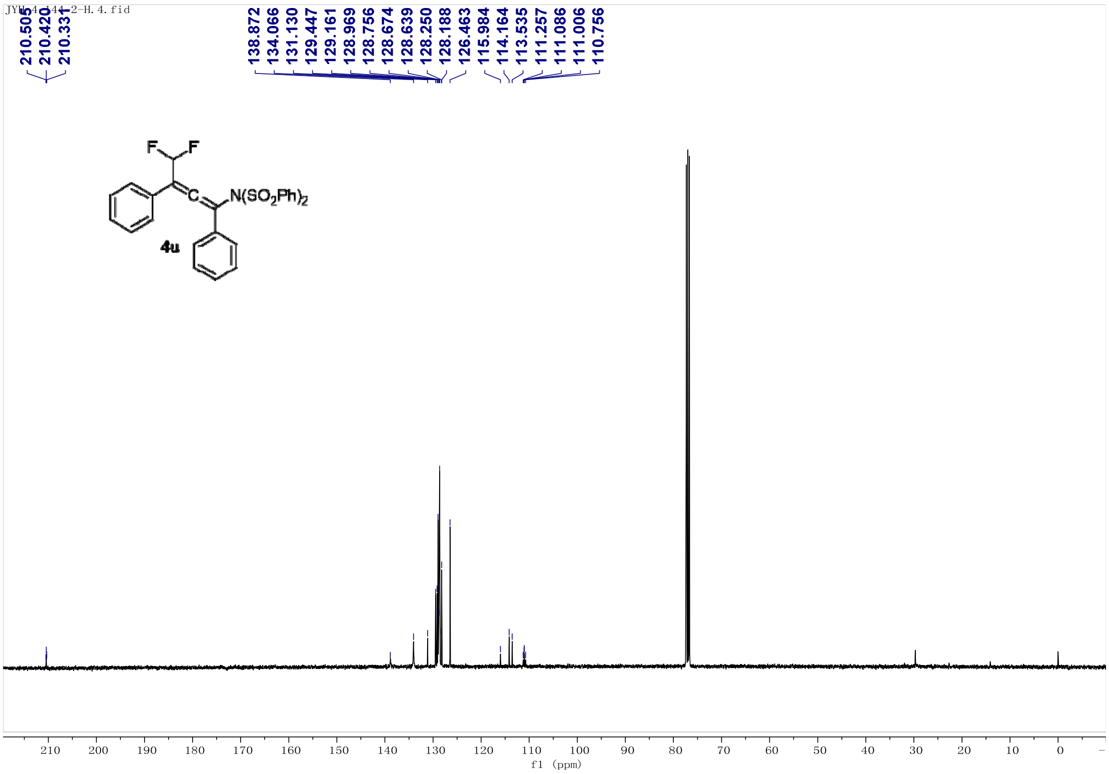

Supplementary Figure 132. ^{13}C NMR spectrum of compound 4u.

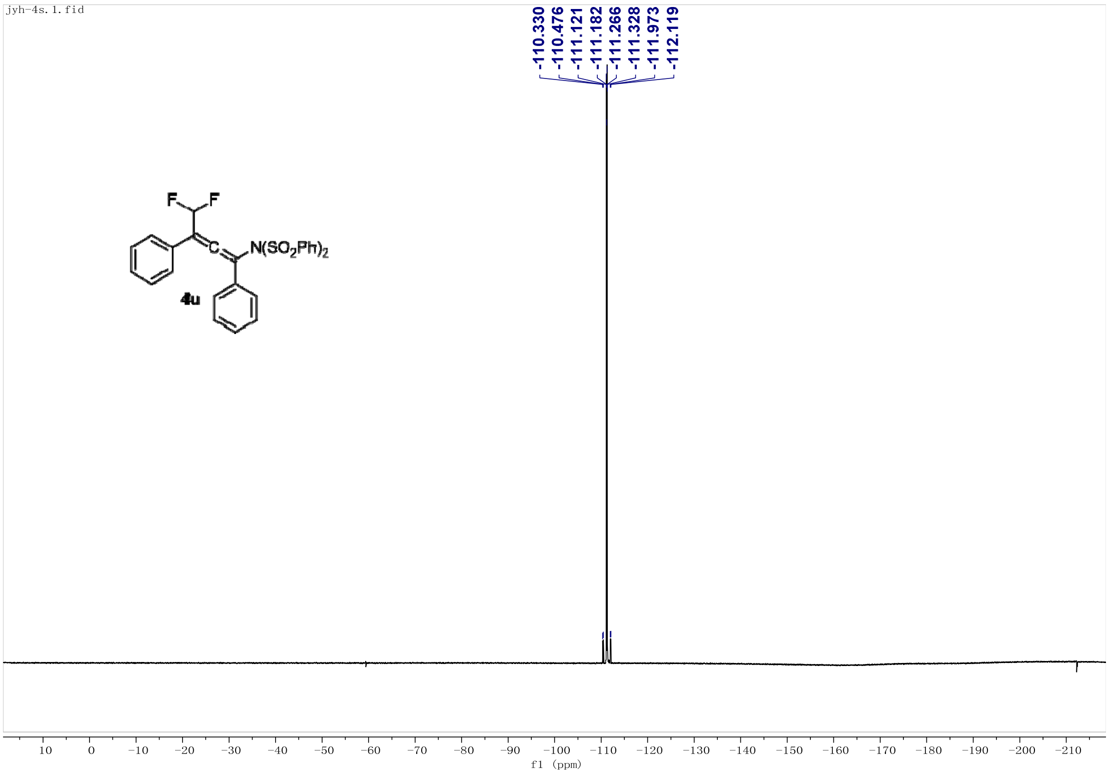

Supplementary Figure 133. **^{19}F NMR spectrum of compound 4u.**

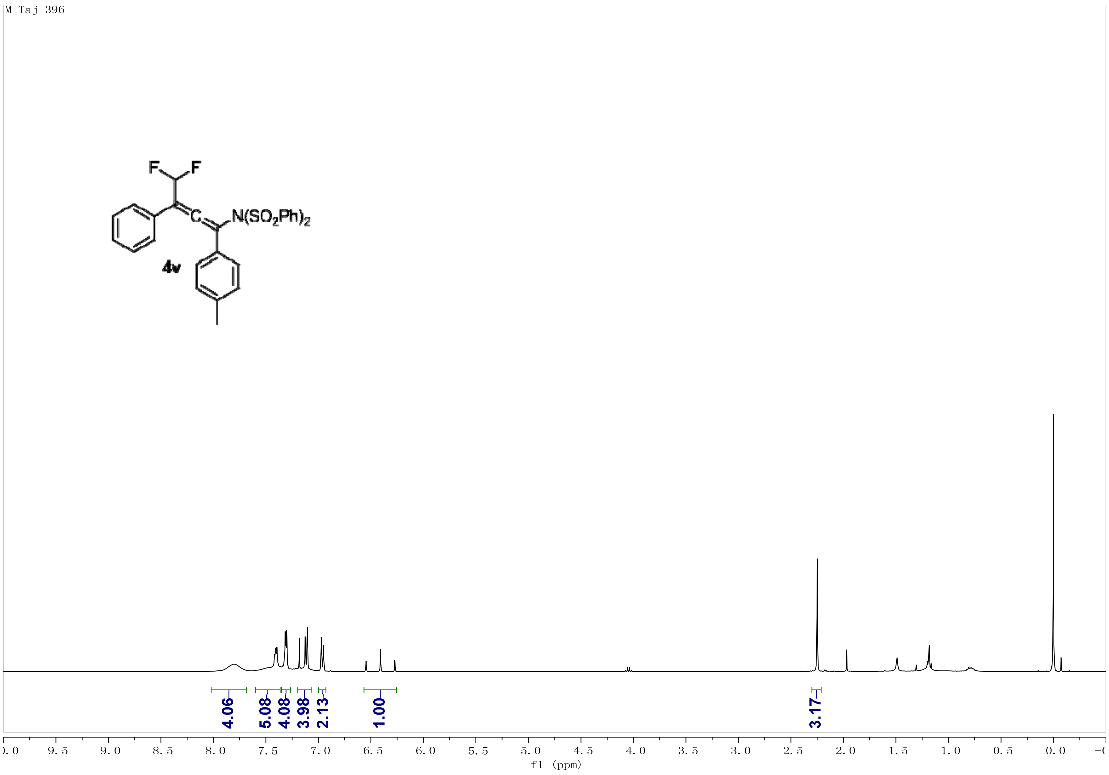

Supplementary Figure 134. ^{1}H NMR spectrum of compound 4v.

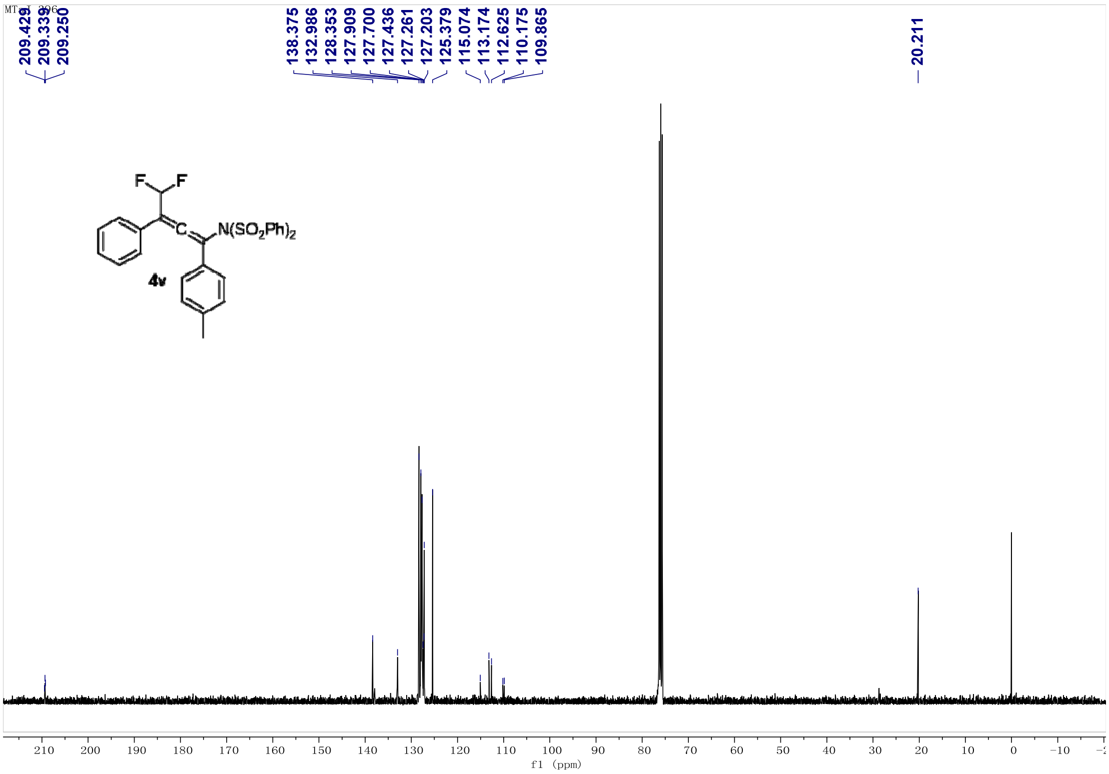

Supplementary Figure 135. ^{13}C NMR spectrum of compound 4v.

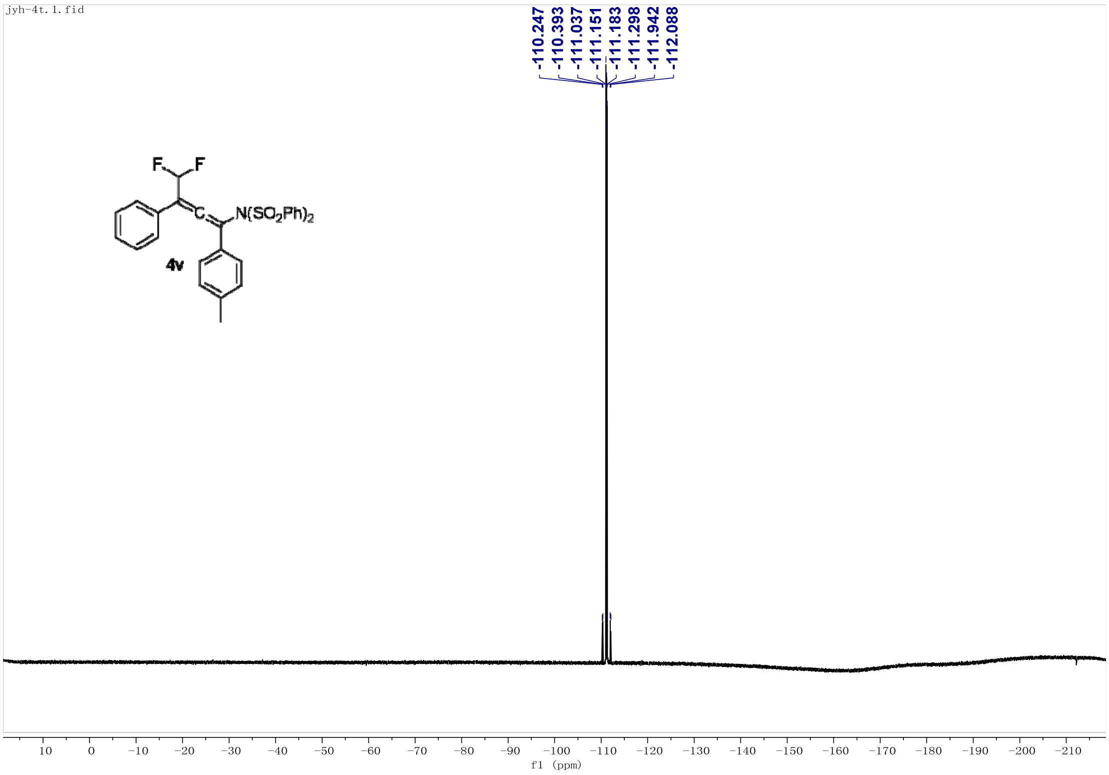

Supplementary Figure 136. ^{19}**F NMR spectrum of compound 4v.**

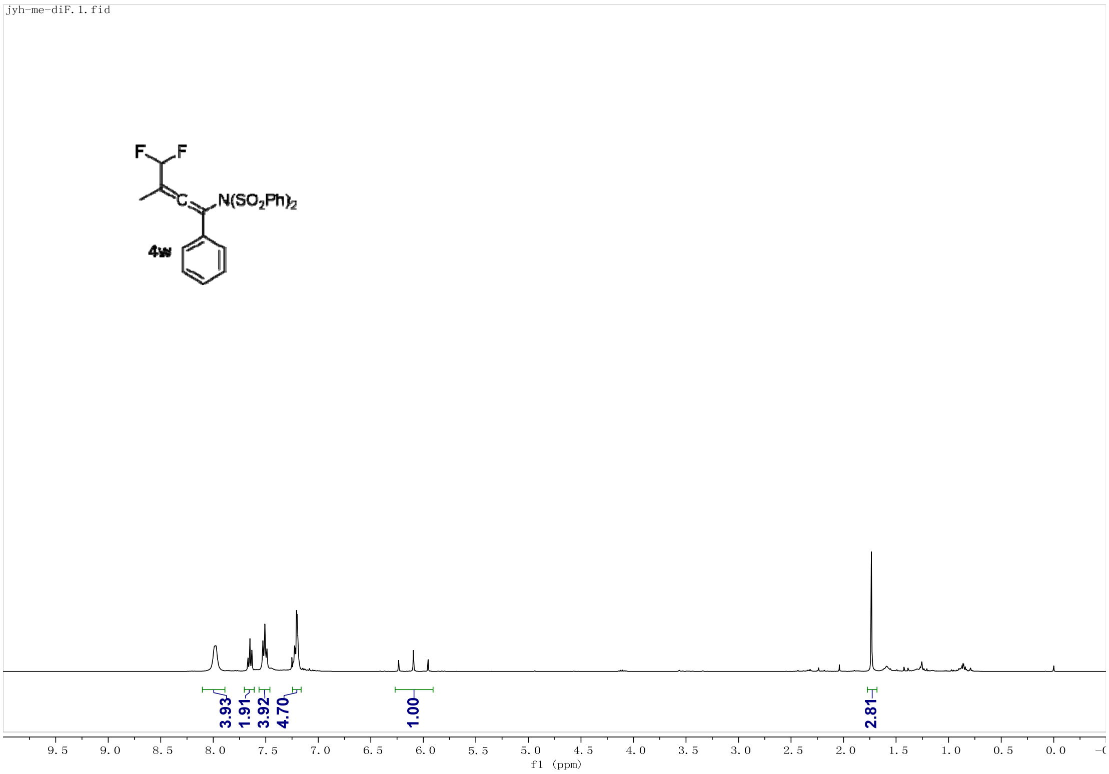

Supplementary Figure 137. ^{1}**H NMR spectrum of compound 4w.**

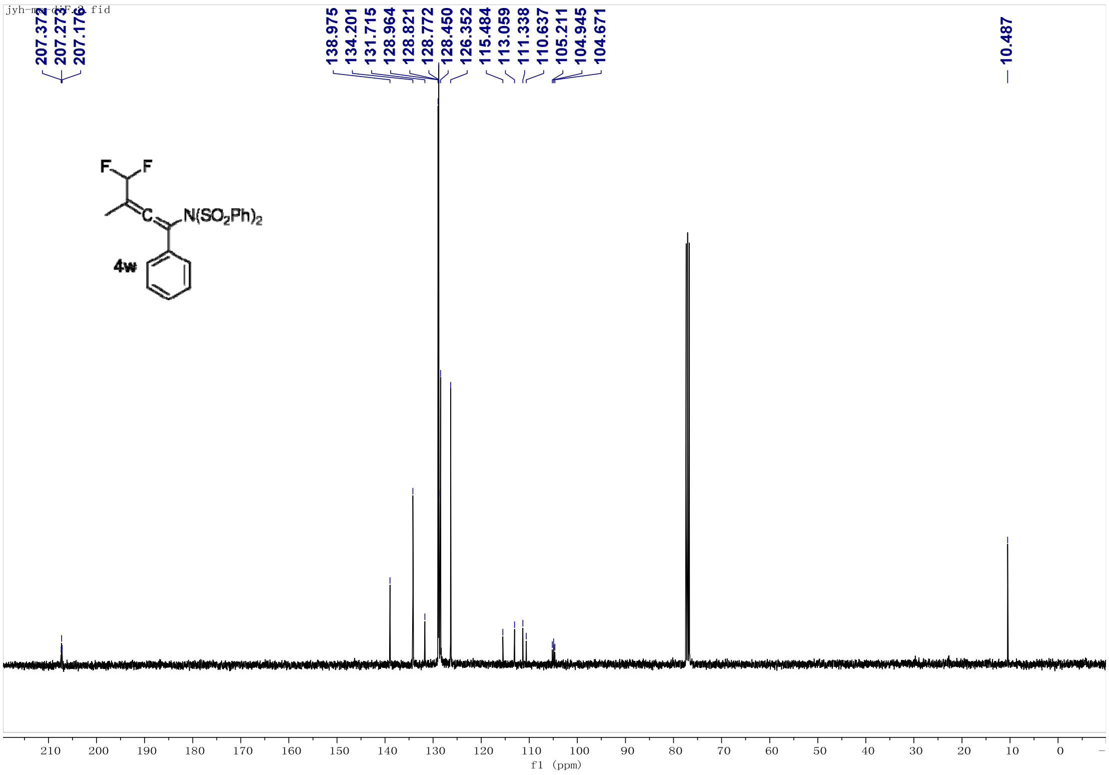

Supplementary Figure 138. ^{13}C NMR spectrum of compound 4w.

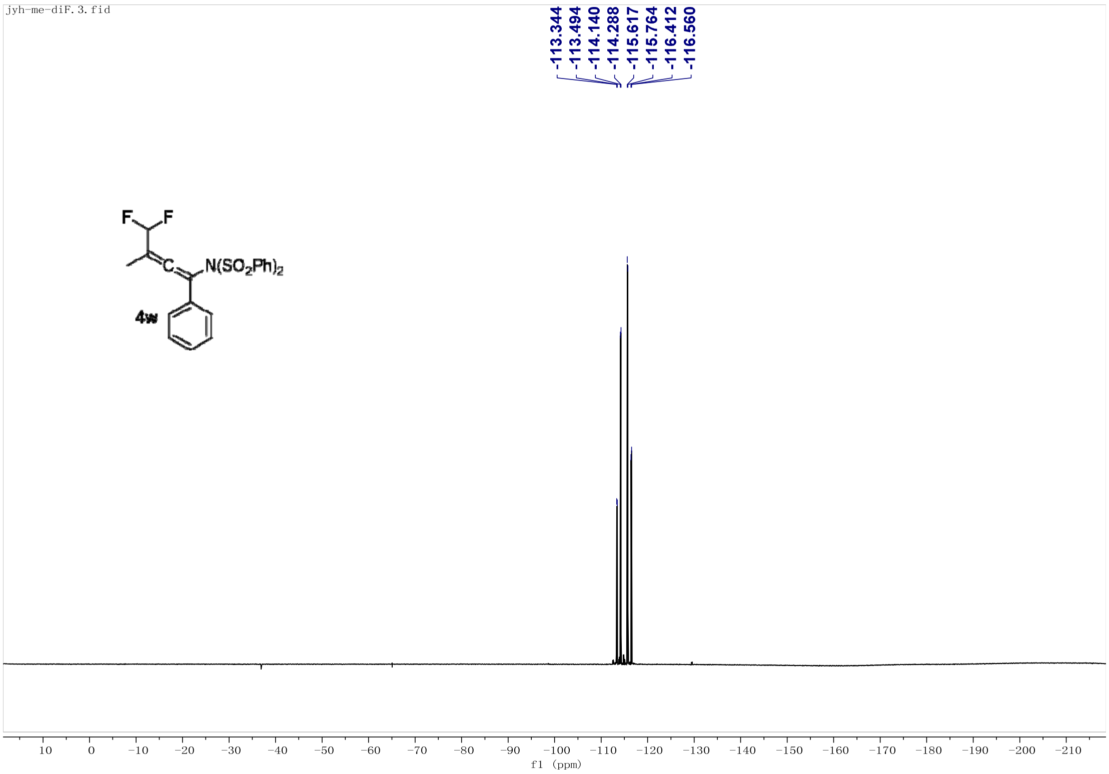

Supplementary Figure 139. ^{19}F NMR spectrum of compound 4w.

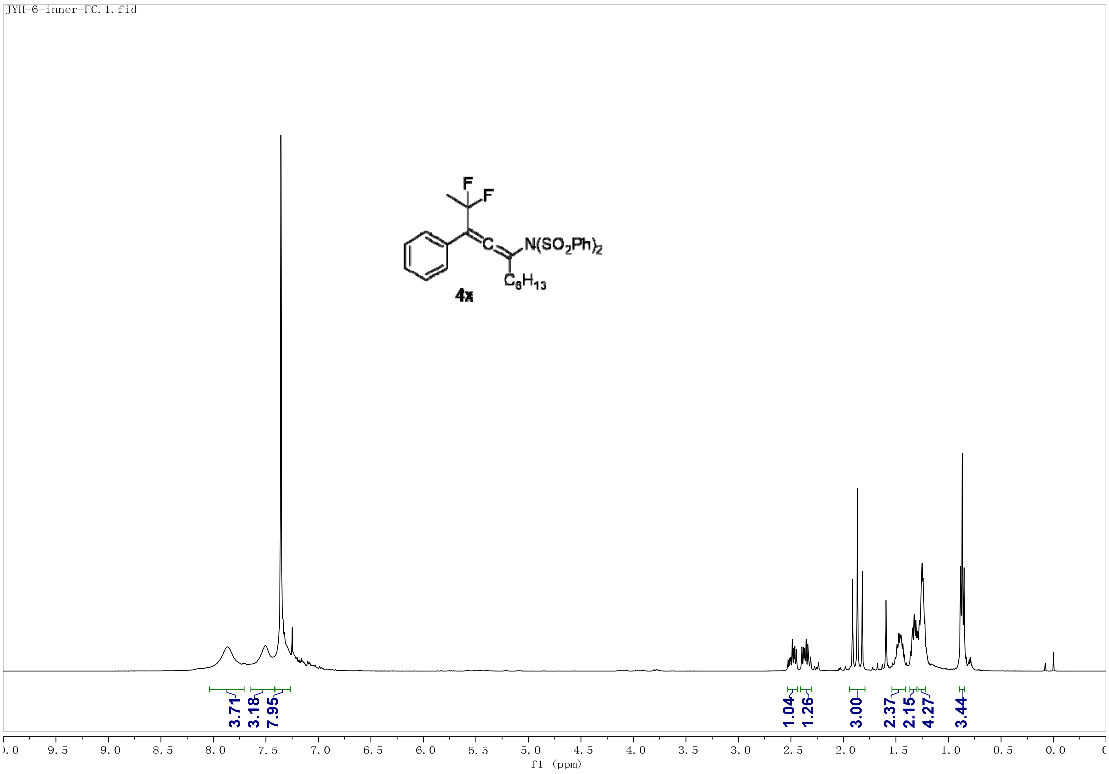

Supplementary Figure 140. ^{1}H NMR spectrum of compound 4x.

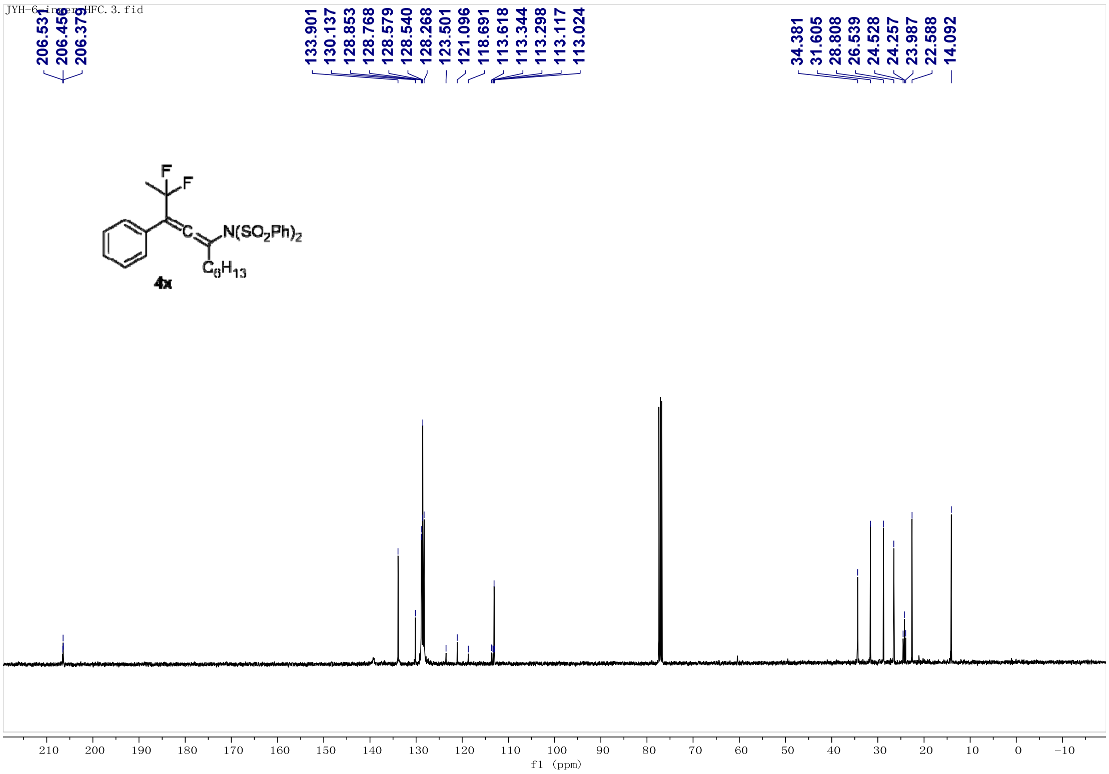

Supplementary Figure 141. ^{13}C NMR spectrum of compound 4x.

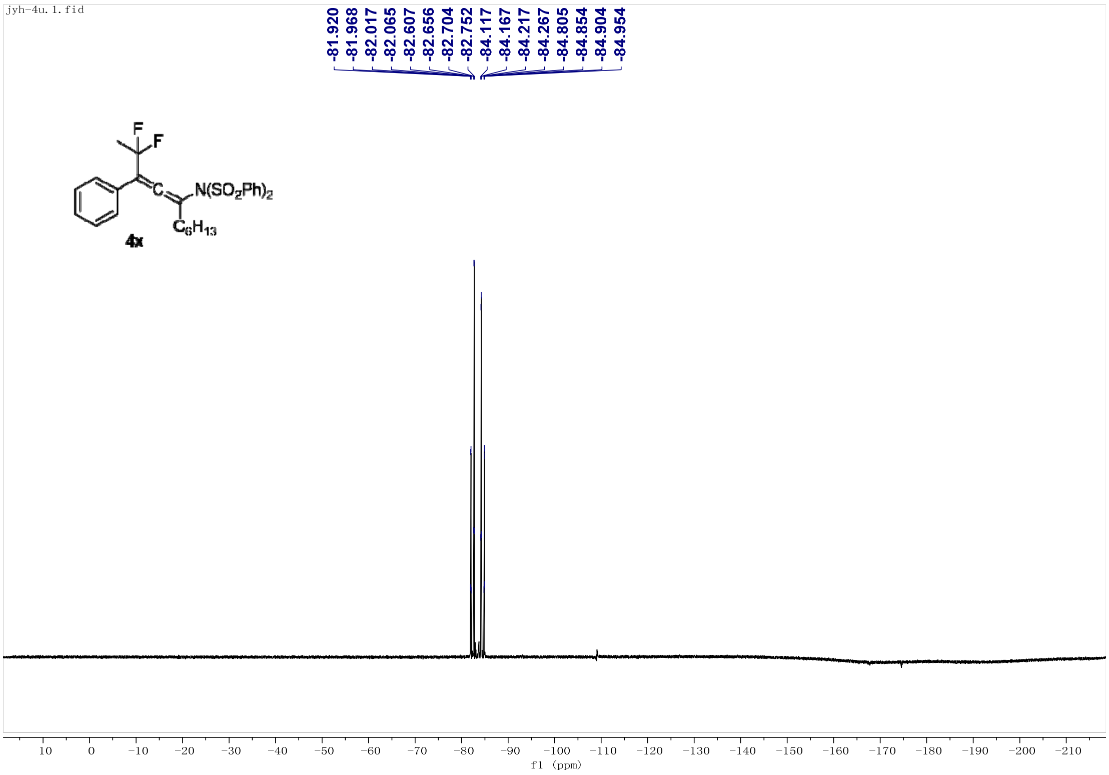

Supplementary Figure 142. ^{19}F NMR spectrum of compound 4x.

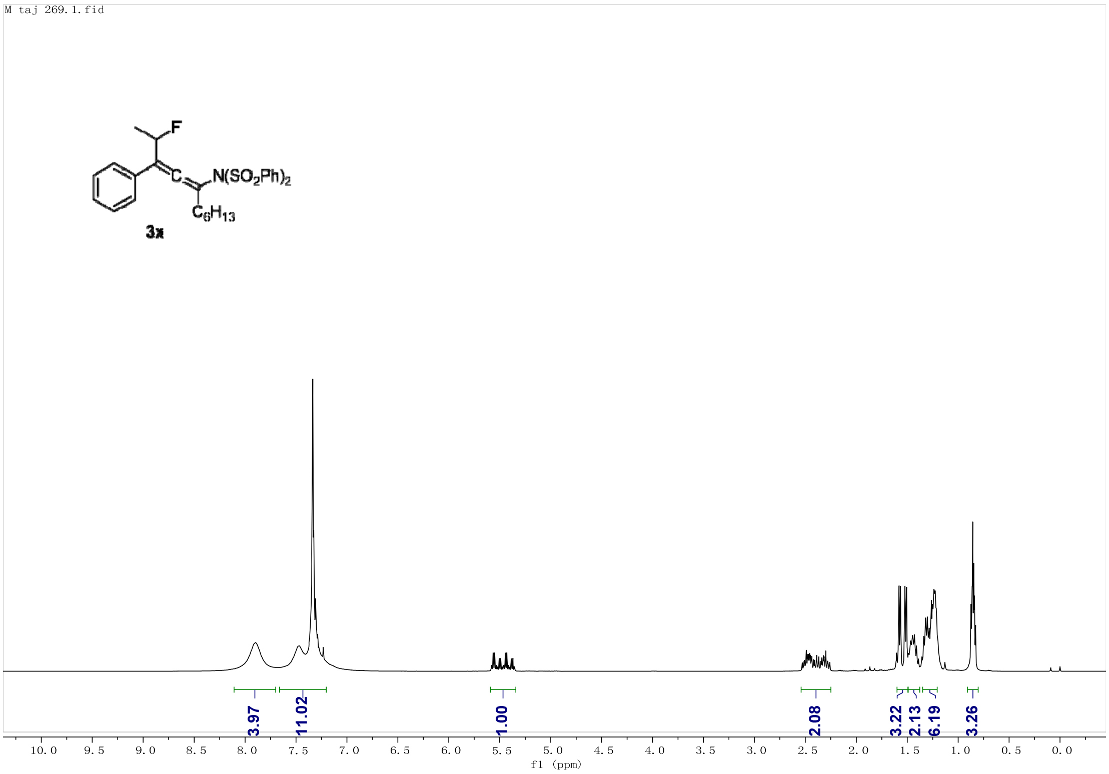

Supplementary Figure 143. **^{1}H NMR spectrum of compound 3x.**

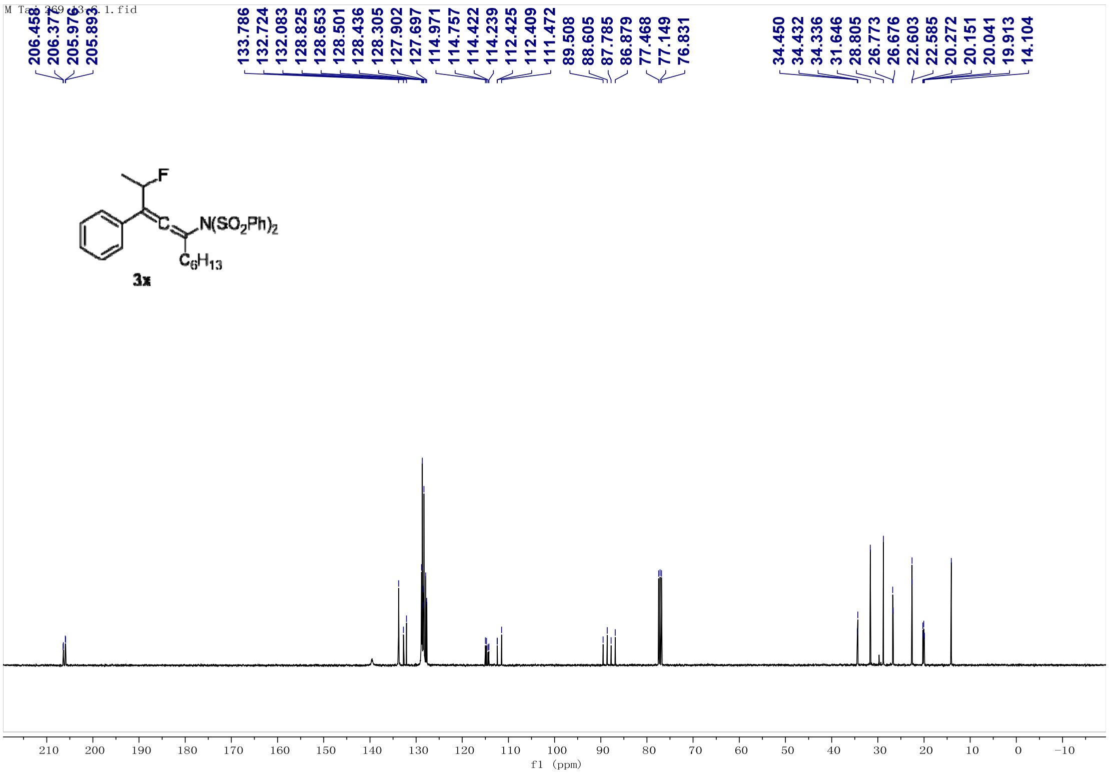

Supplementary Figure 144. ^{13}**C NMR spectrum of compound 3x.**

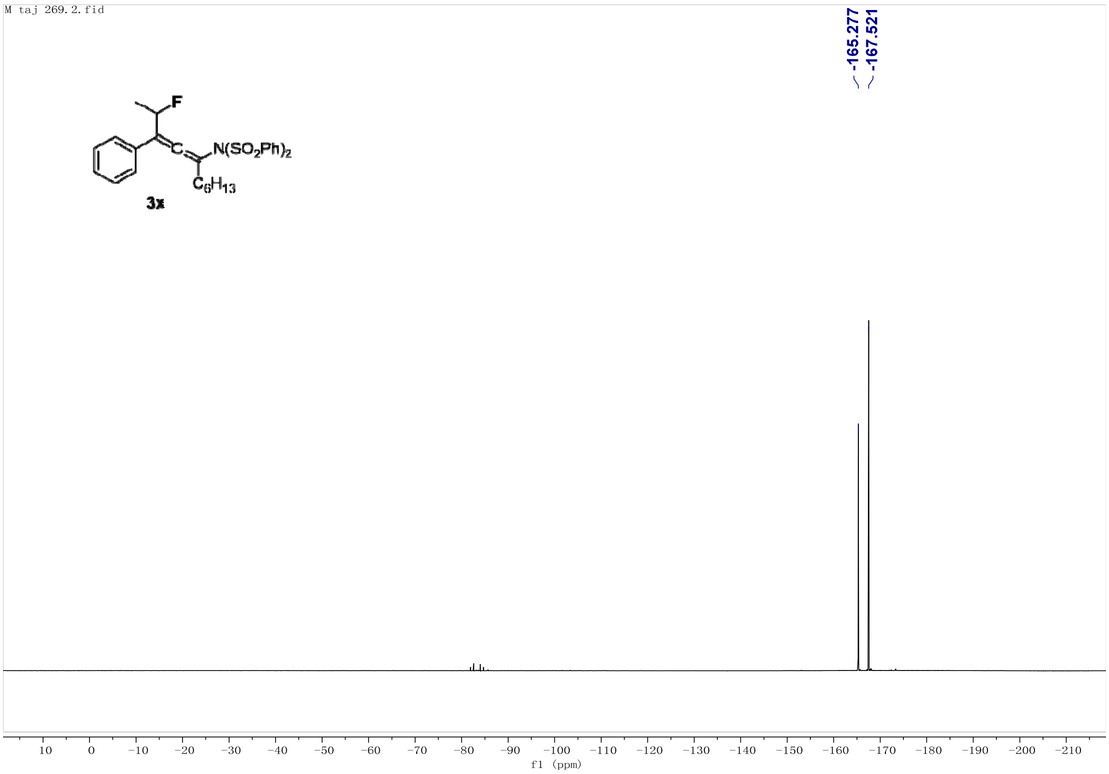

Supplementary Figure 145. ^{19}**F NMR spectrum of compound 3x.**

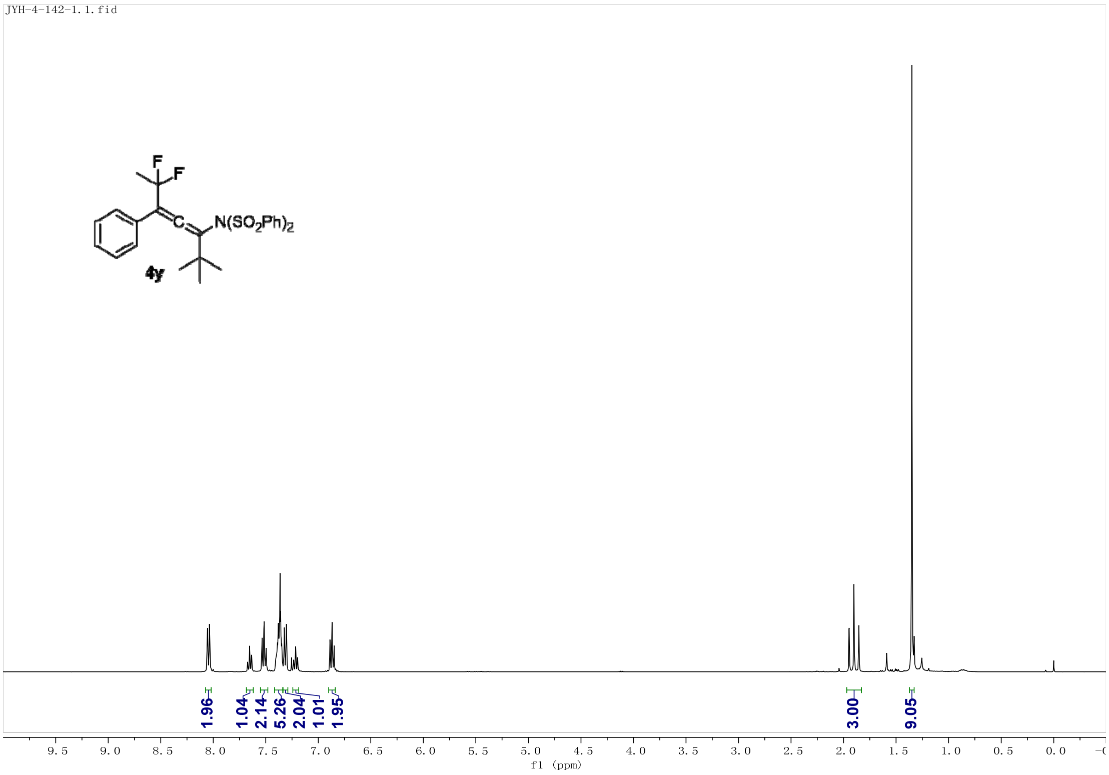

Supplementary Figure 146. **^{1}H NMR spectrum of compound 4y.**

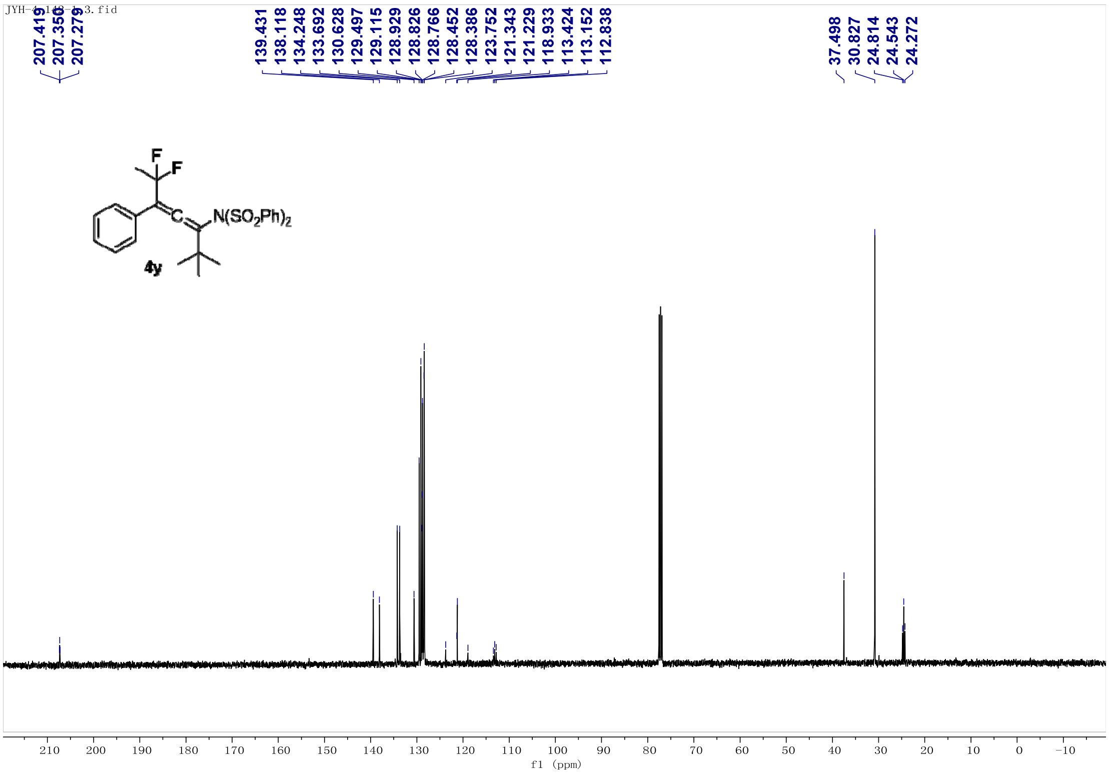

Supplementary Figure 147. **^{13}C NMR spectrum of compound 4y.**

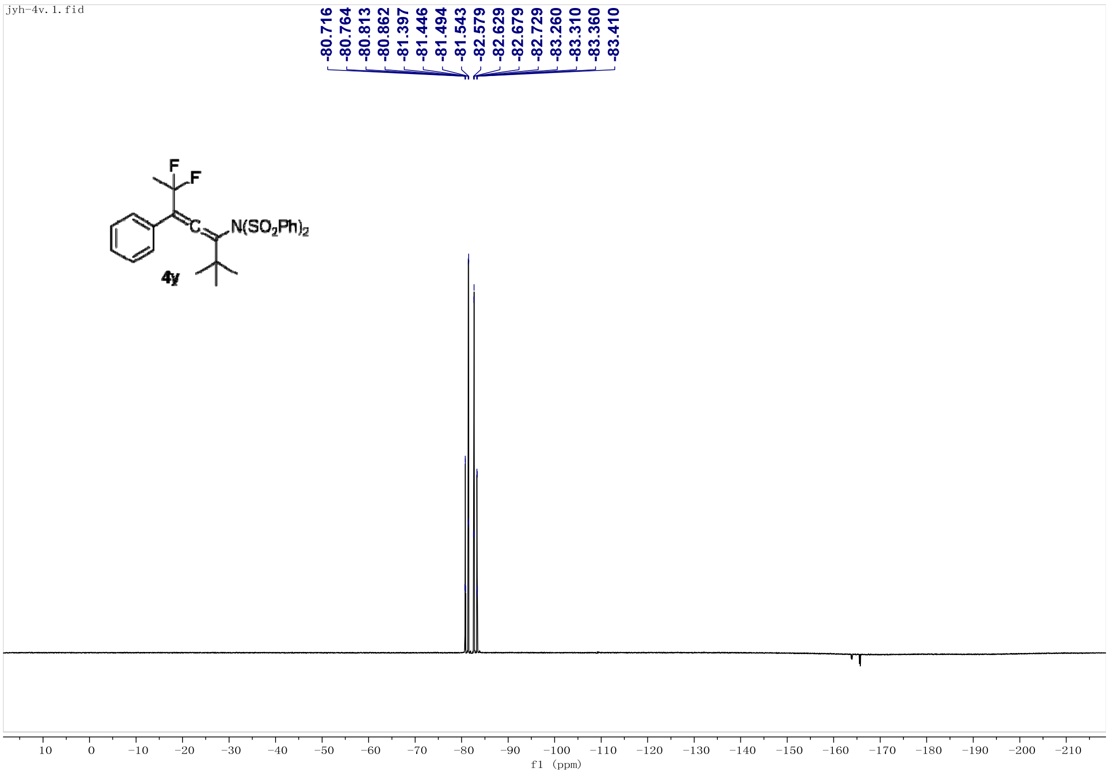

Supplementary Figure 148. ^{19}F **NMR spectrum of compound 4y.**

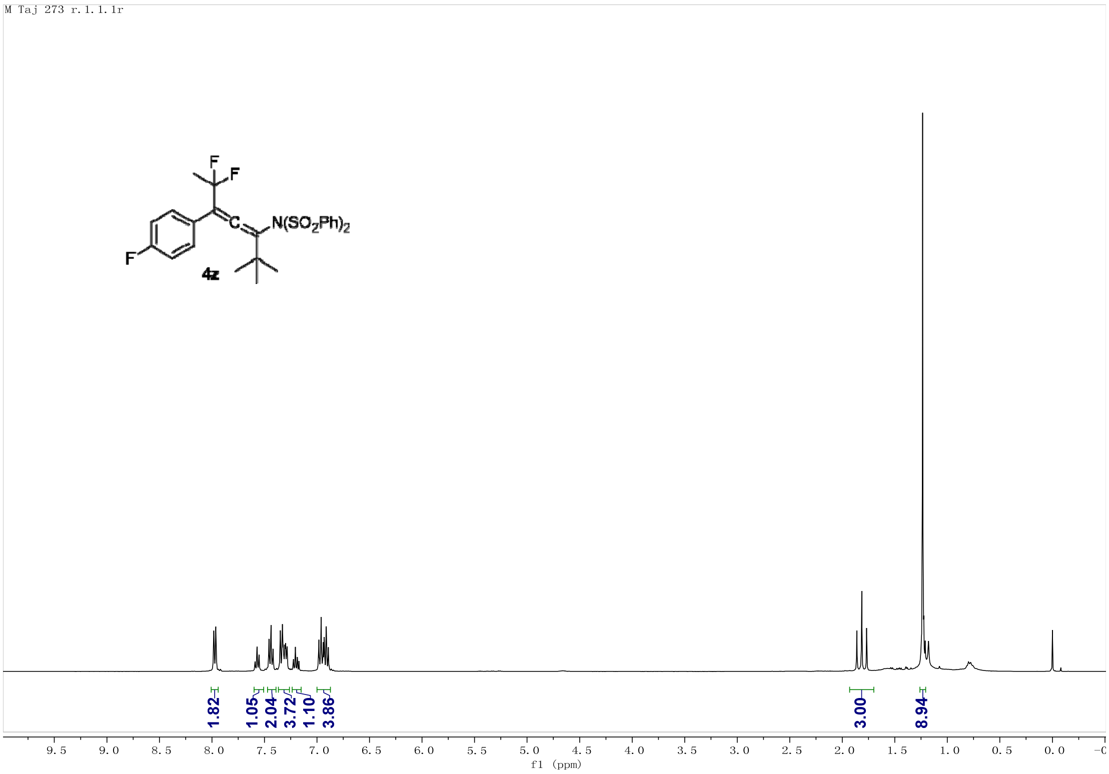

Supplementary Figure 149. ^{1}H **NMR spectrum of compound 4z.**

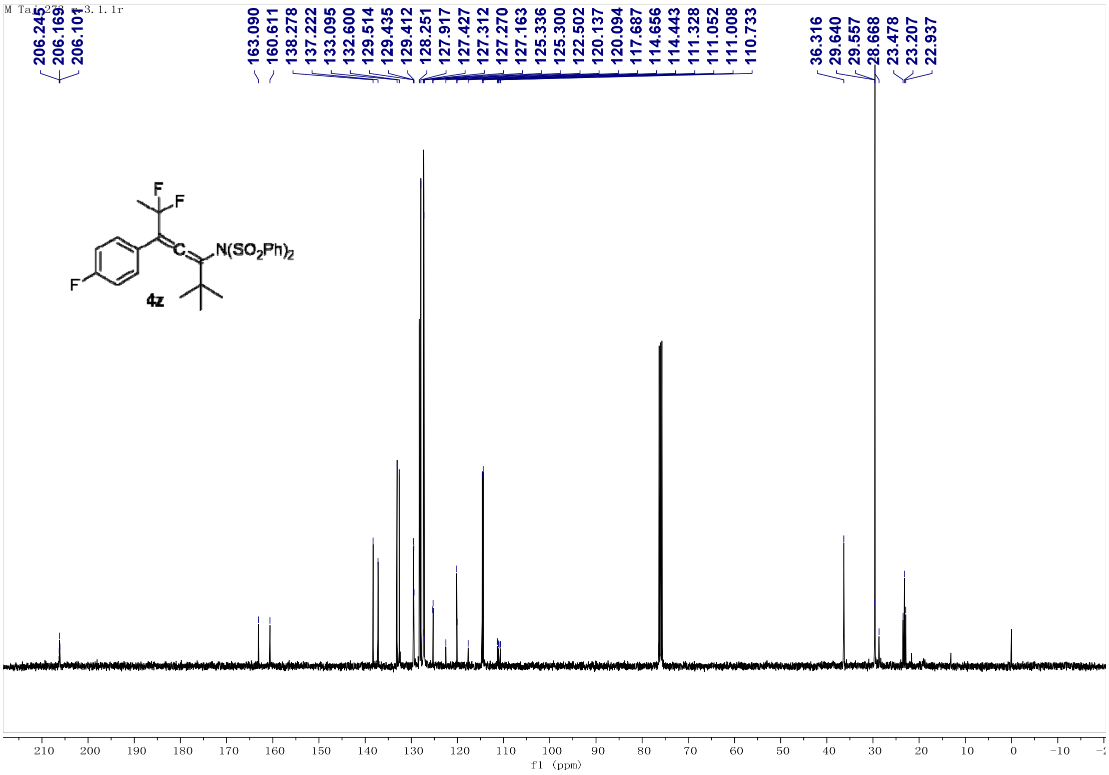

Supplementary Figure 150. ^{13}C NMR spectrum of compound 4z.

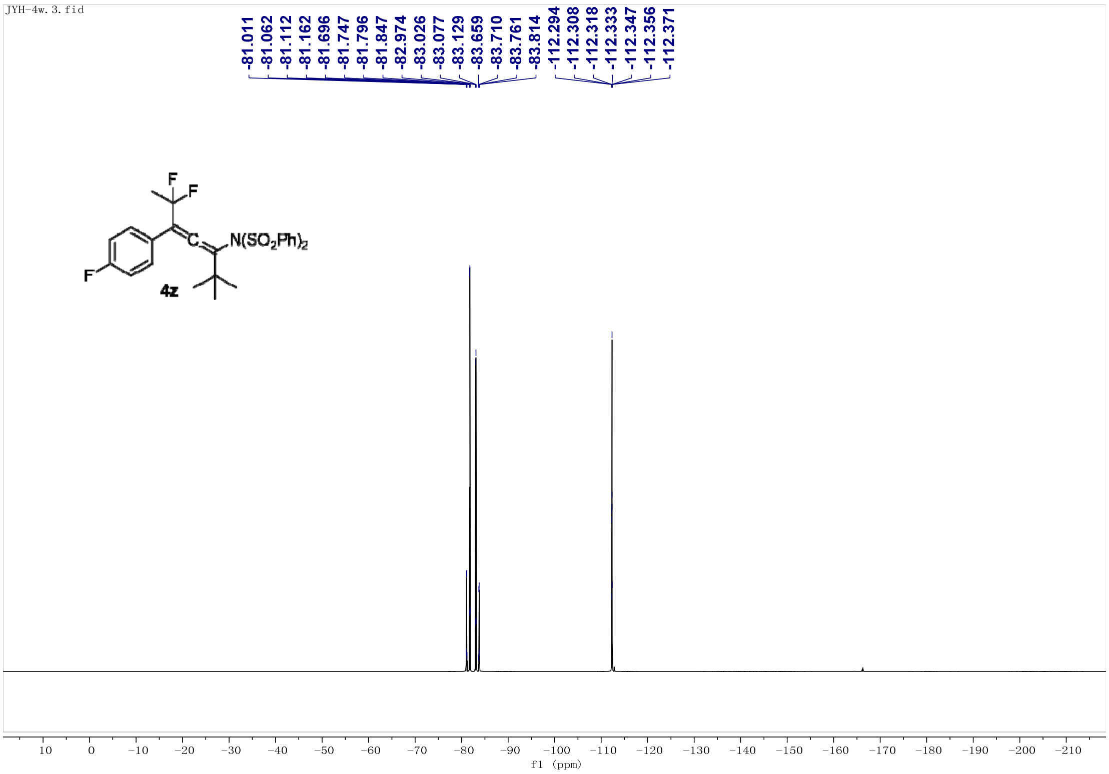

Supplementary Figure 151. ^{19}F NMR spectrum of compound 4z.

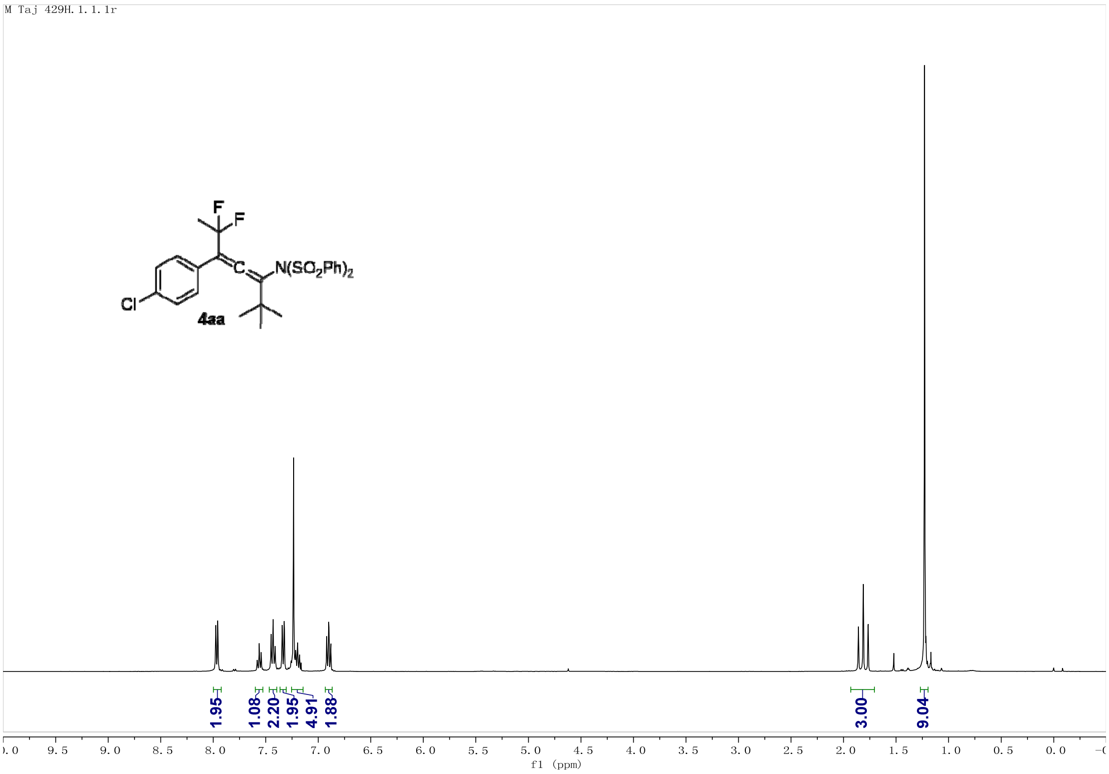

Supplementary Figure 152. ^{1}H NMR spectrum of compound 4aa.

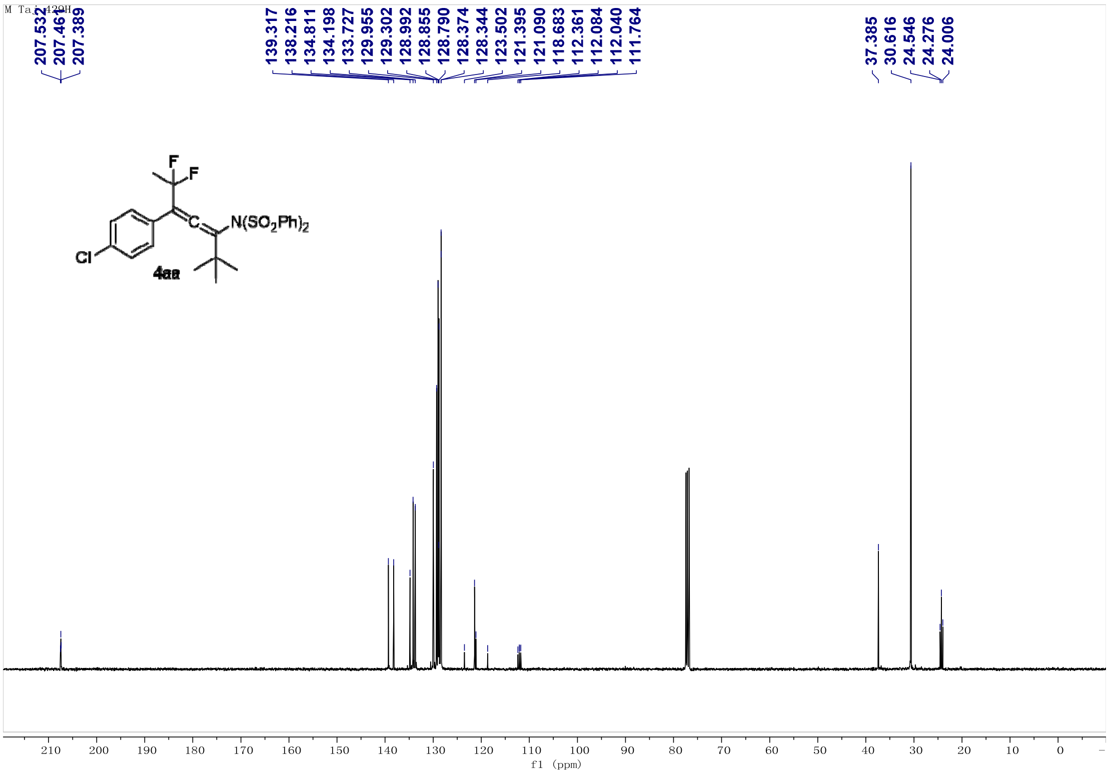

Supplementary Figure 153. ^{13}C NMR spectrum of compound 4aa.

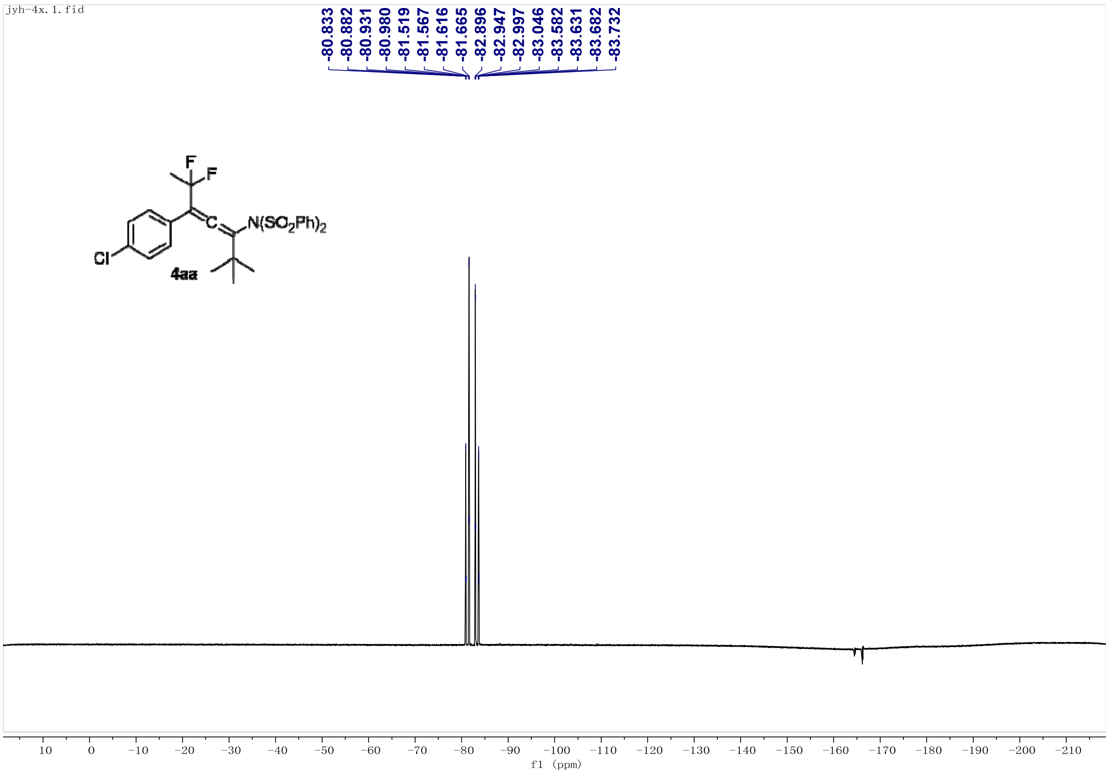

Supplementary Figure 154. **^{19}F NMR spectrum of compound 4aa.**

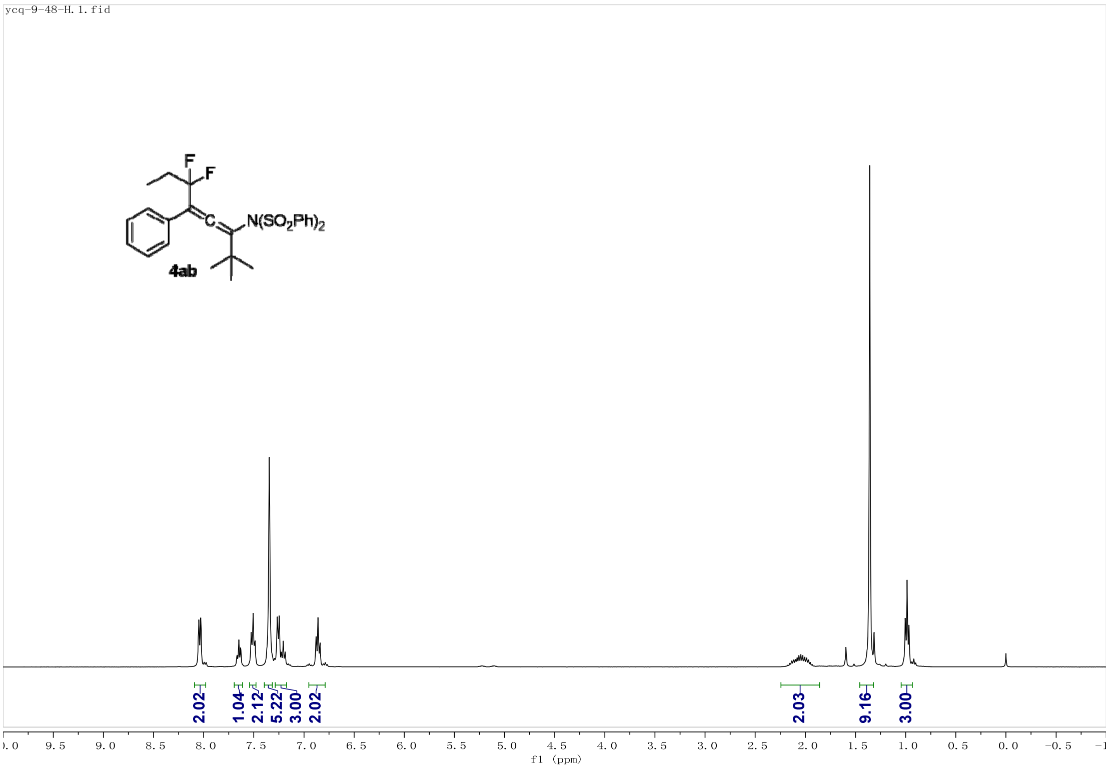

Supplementary Figure 155. **^{1}H NMR spectrum of compound 4ab.**

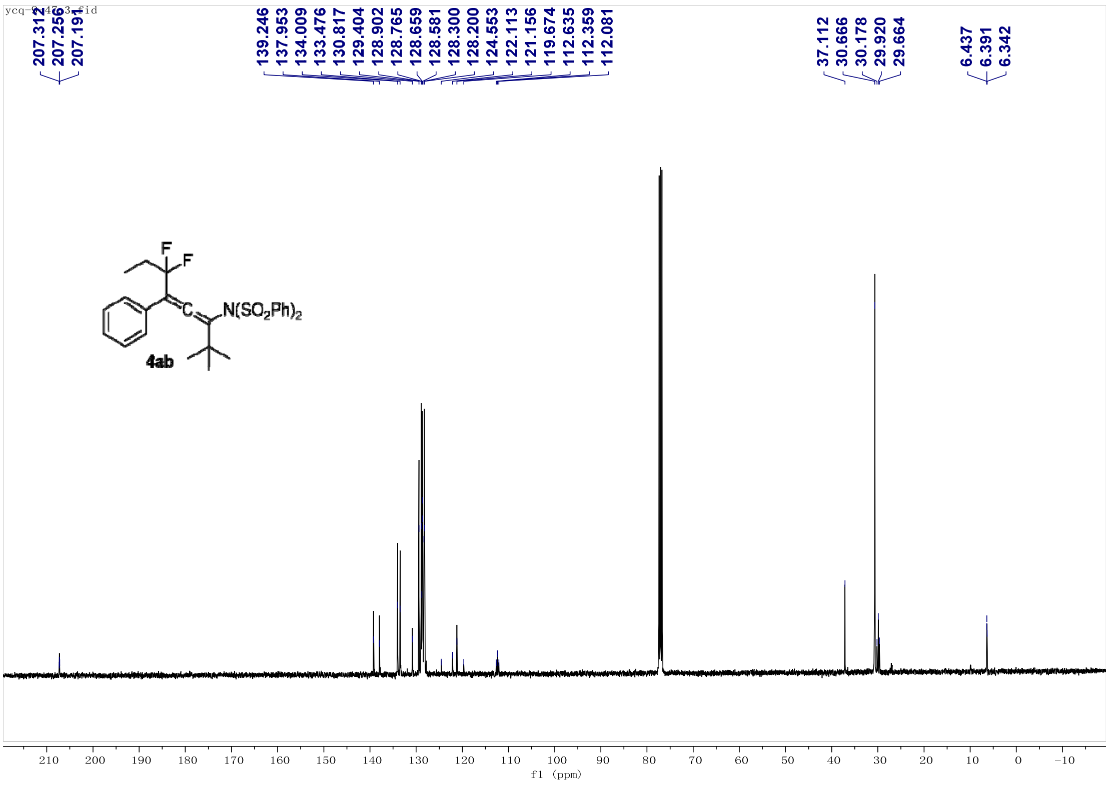

Supplementary Figure 156. ^{13}C NMR spectrum of compound 4ab.

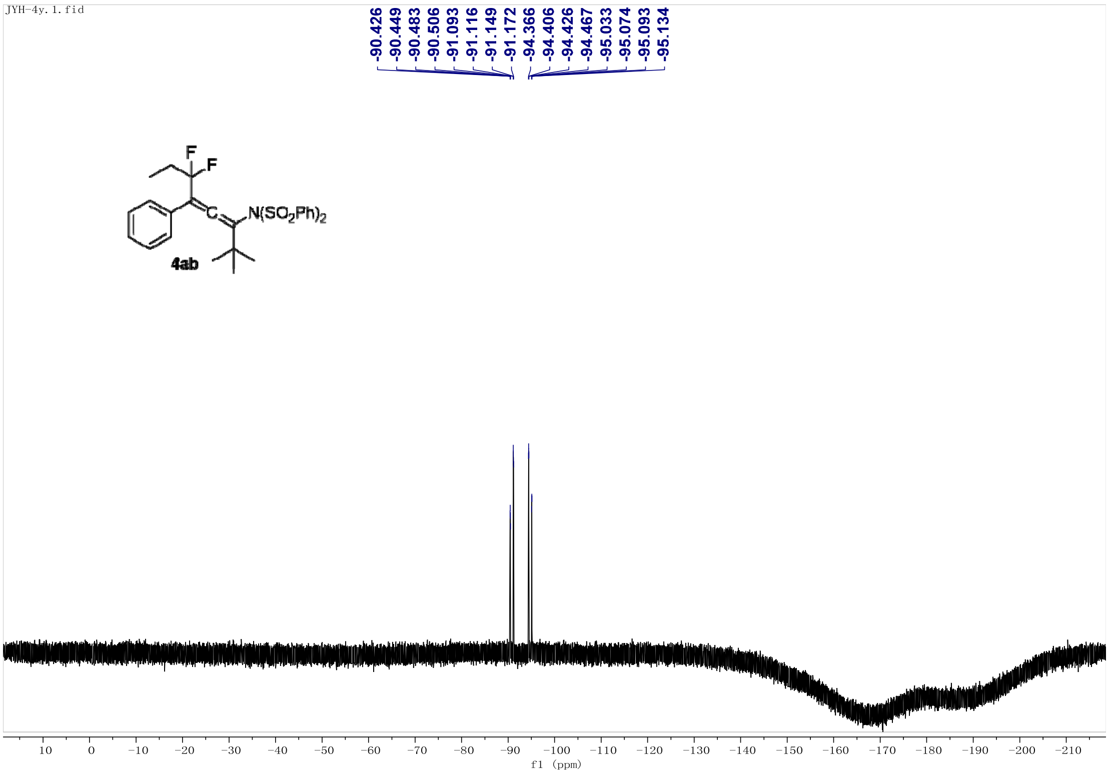

Supplementary Figure 157. ^{19}F NMR spectrum of compound 4ab.

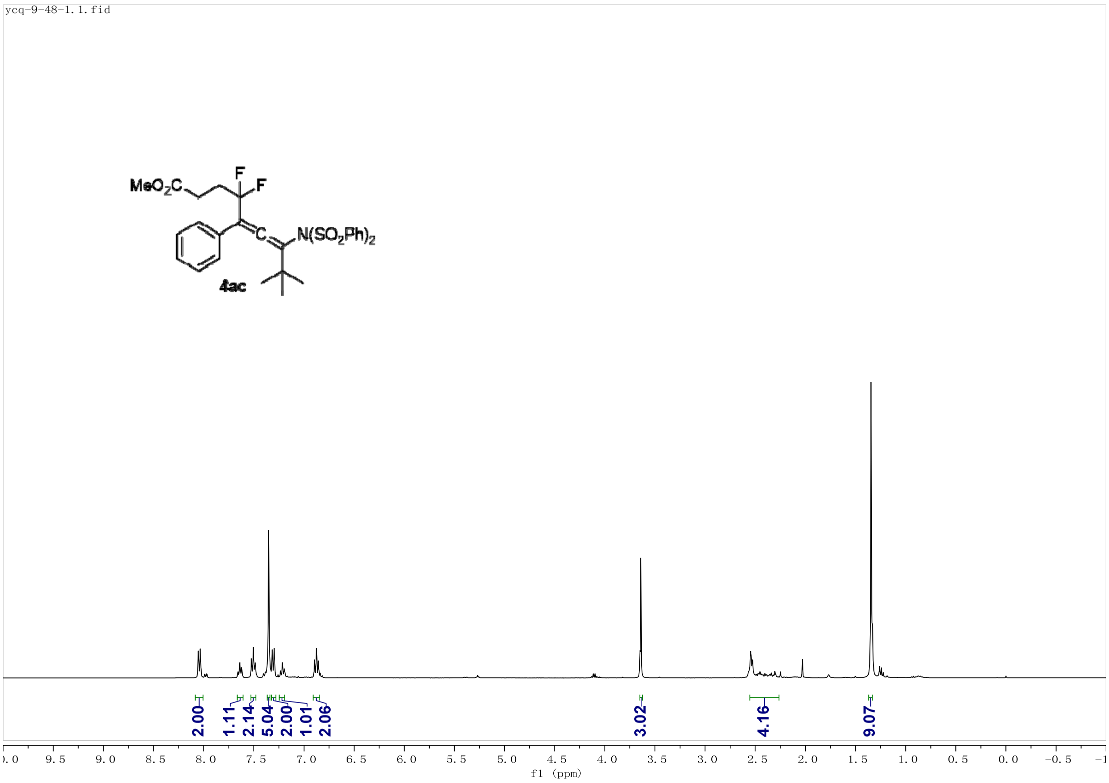

Supplementary Figure 158. ^{1}H **NMR spectrum of compound 4ac.**

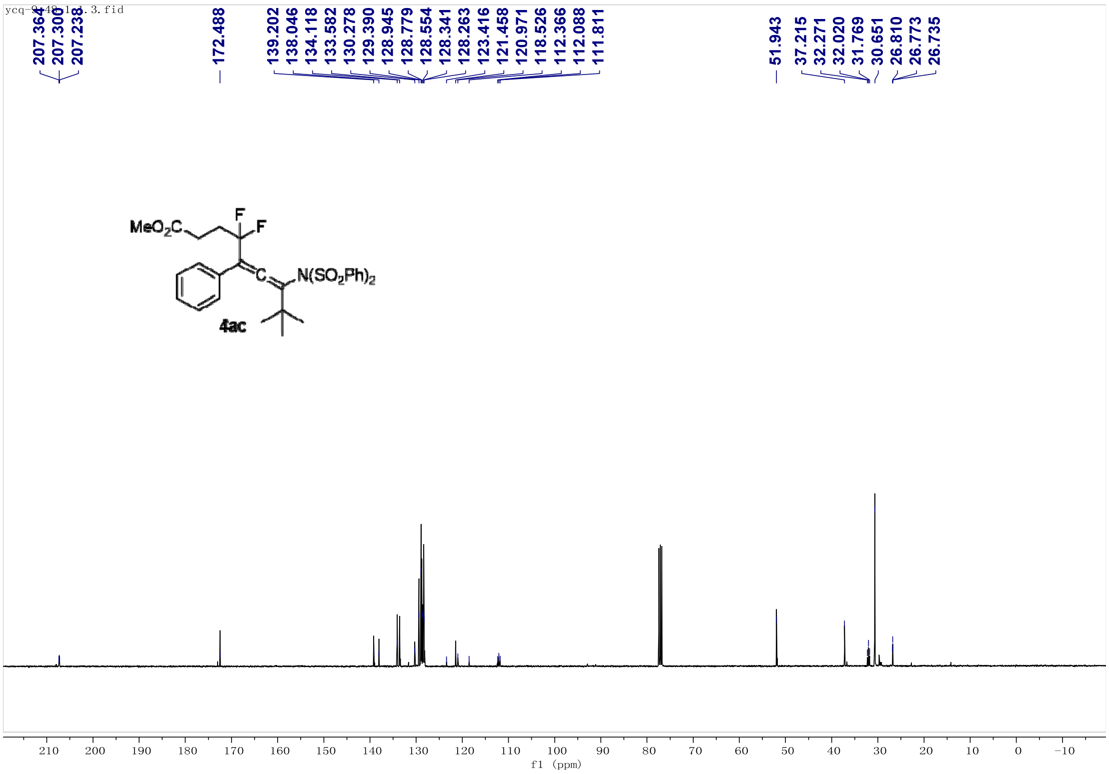

Supplementary Figure 159. ^{13}C **NMR spectrum of compound 4ac.**

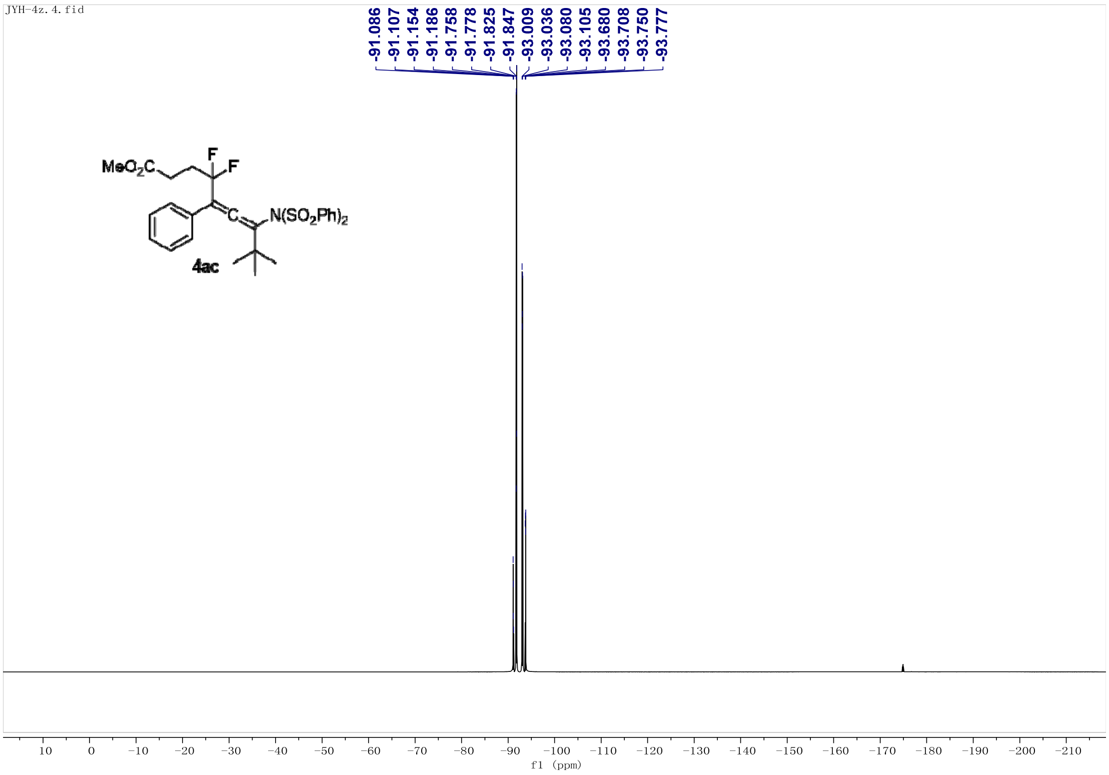

Supplementary Figure 160. **^{19}F NMR spectrum of compound 4ac.**

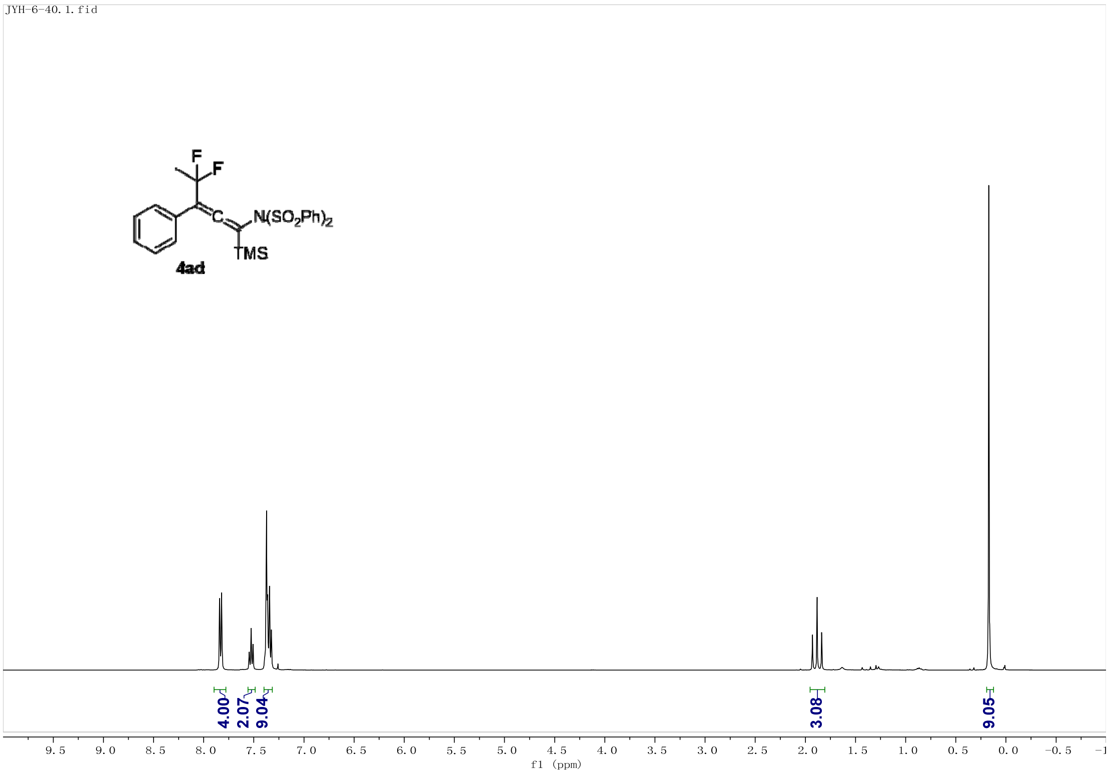

Supplementary Figure 161. ^{1}H NMR spectrum of compound 4ad.

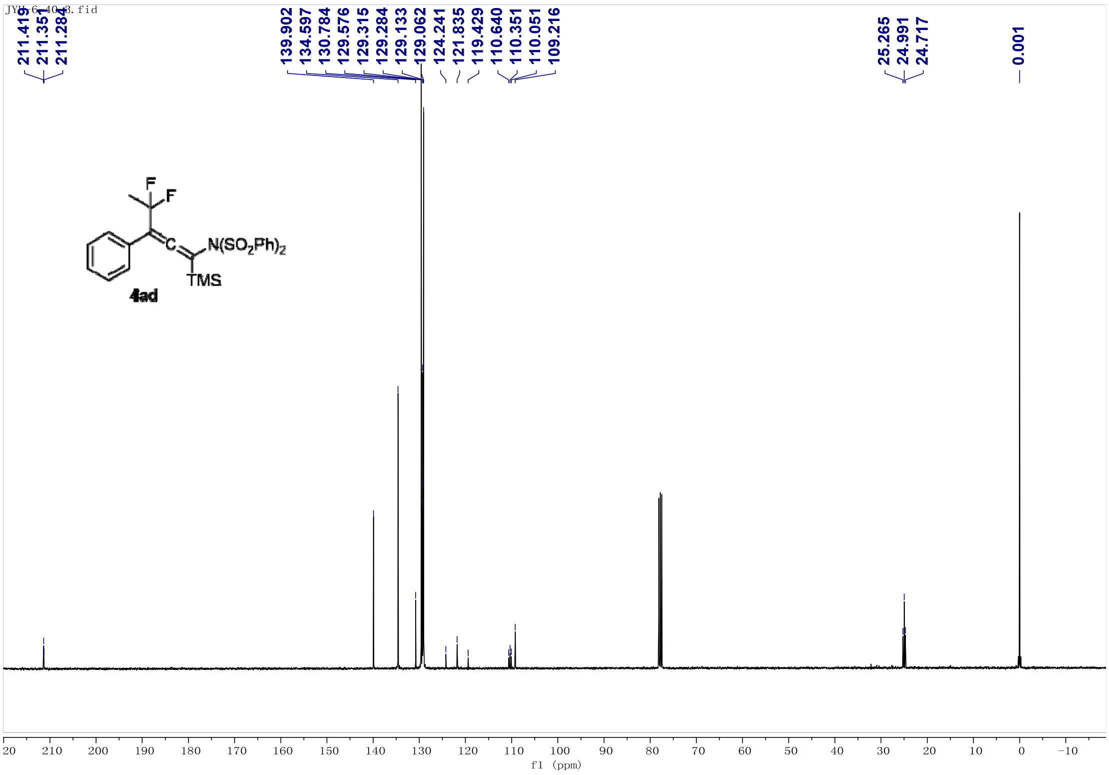

Supplementary Figure 162. ^{13}C NMR spectrum of compound 4ad.

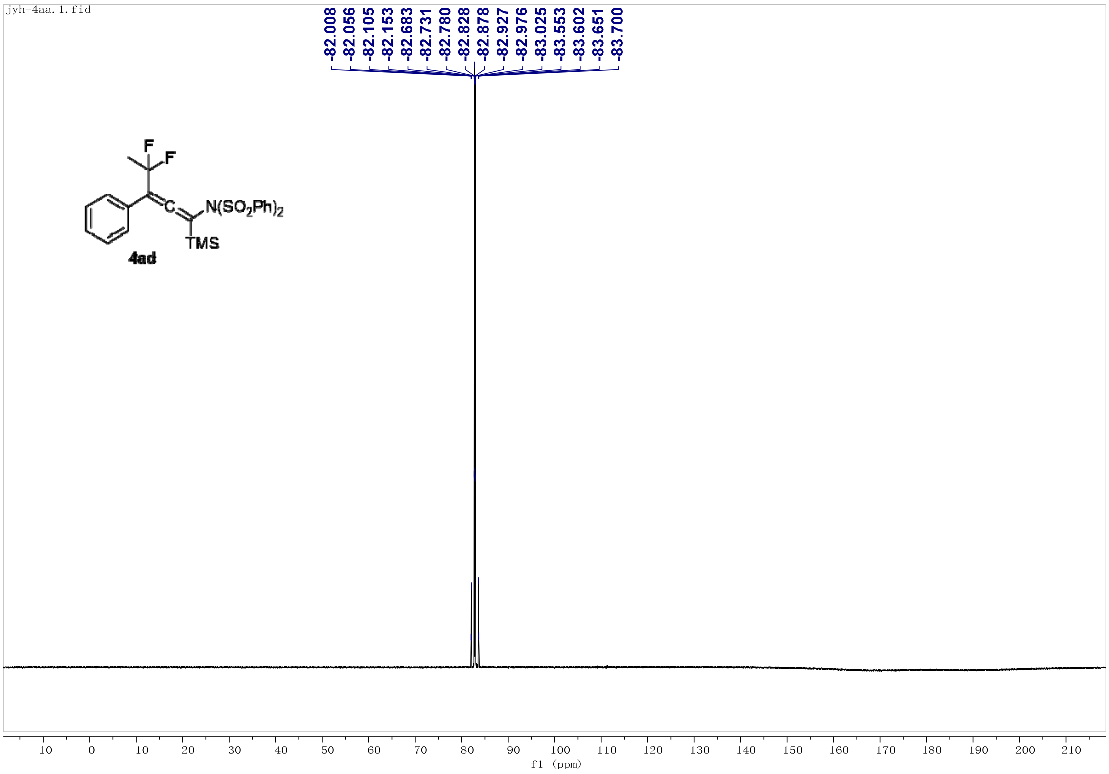

Supplementary Figure 163. ^{19}F NMR spectrum of compound 4ad.

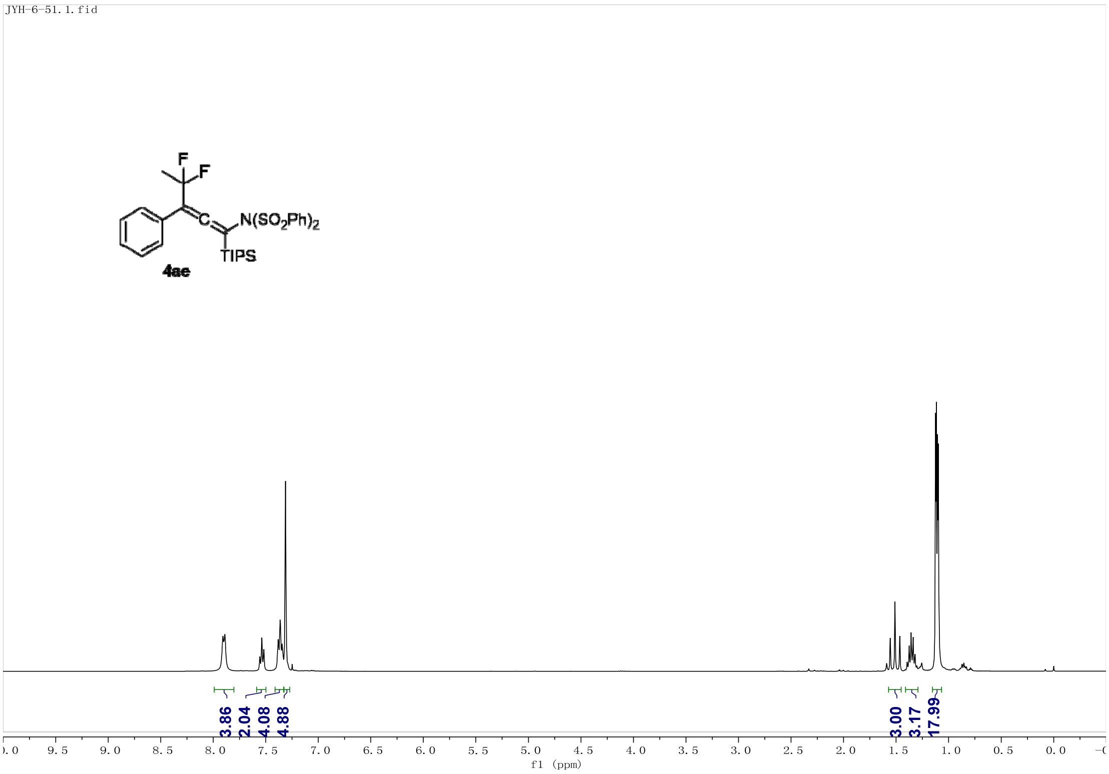

Supplementary Figure 164. ^{1}H NMR spectrum of compound 4ae.

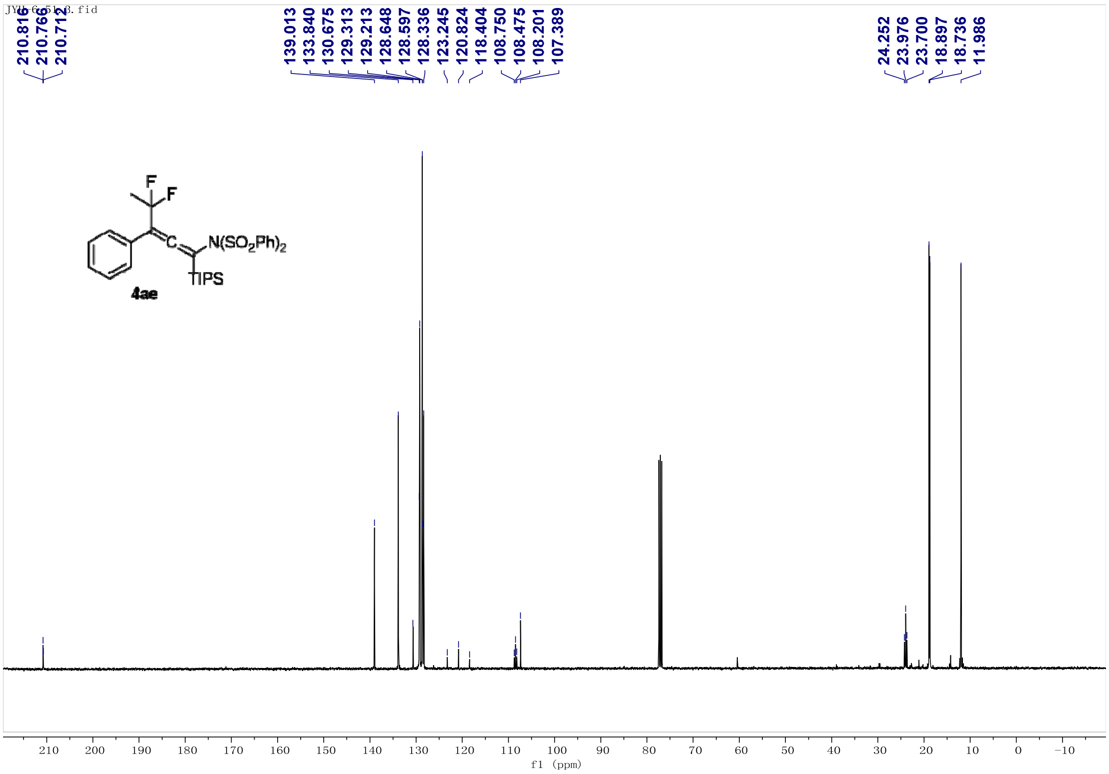

Supplementary Figure 165. ^{13}C NMR spectrum of compound 4ae.

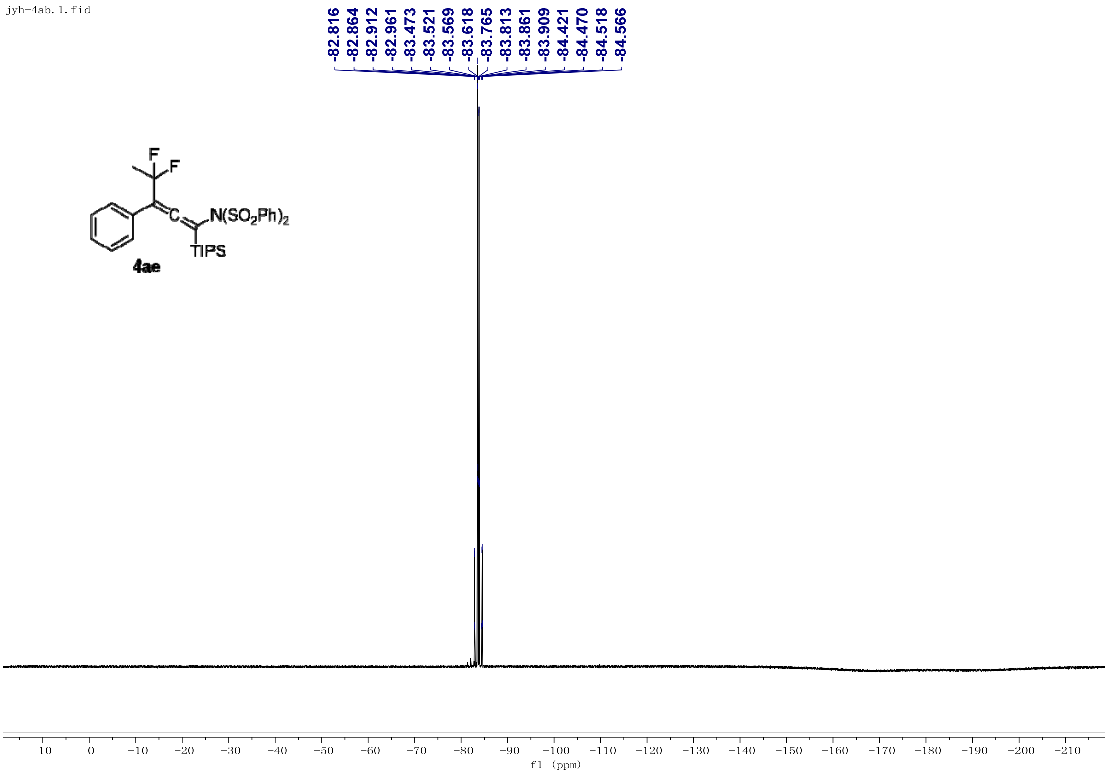

Supplementary Figure 166. ^{19}F **NMR spectrum of compound 4ae.**

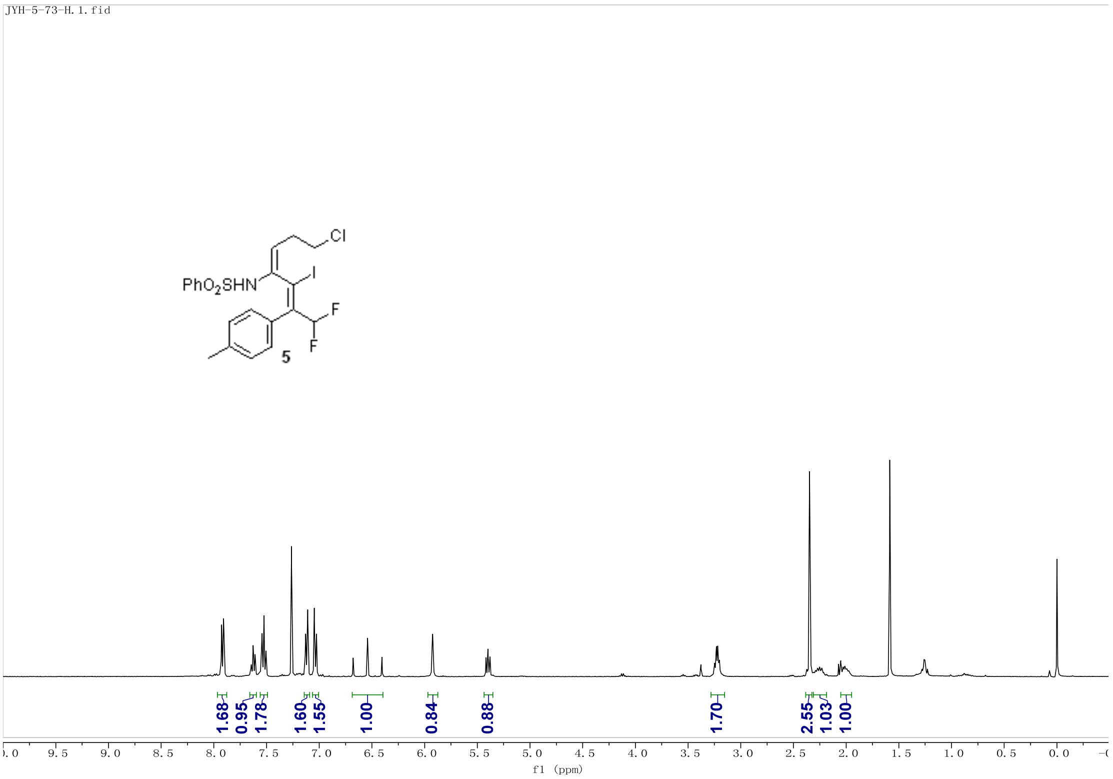

Supplementary Figure 167. ^{1}H **NMR spectrum of compound 5.**

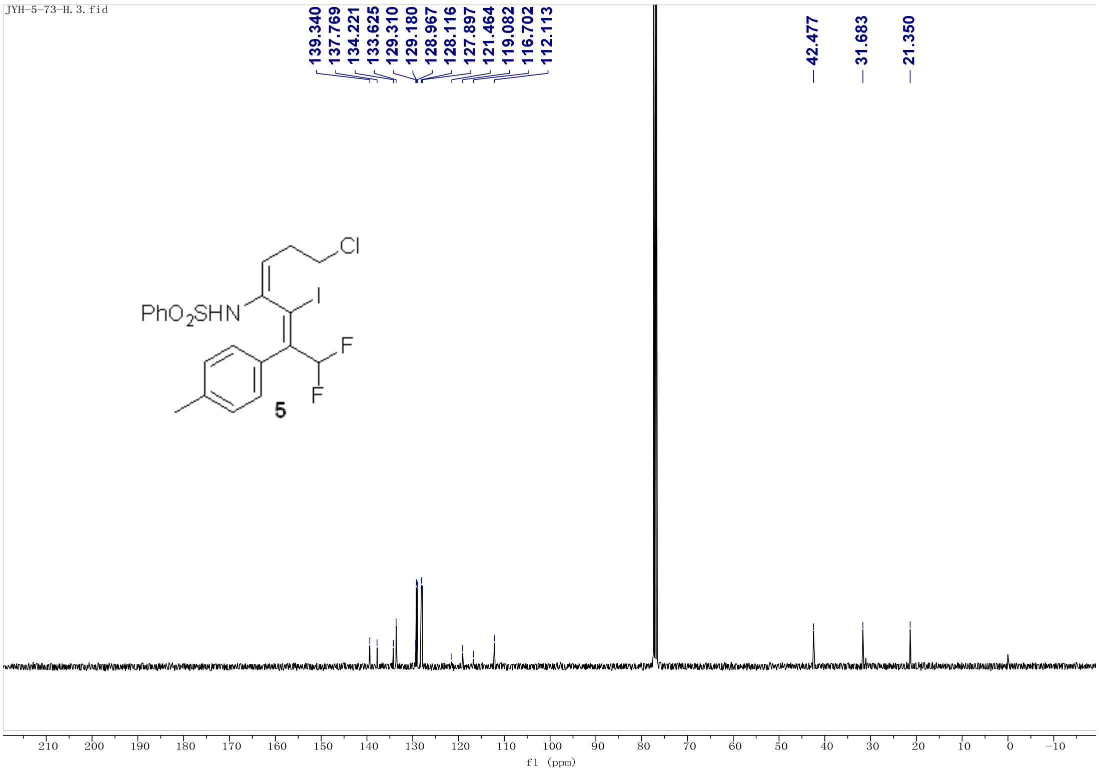

Supplementary Figure 168. **^{13}C NMR spectrum of compound 5.**

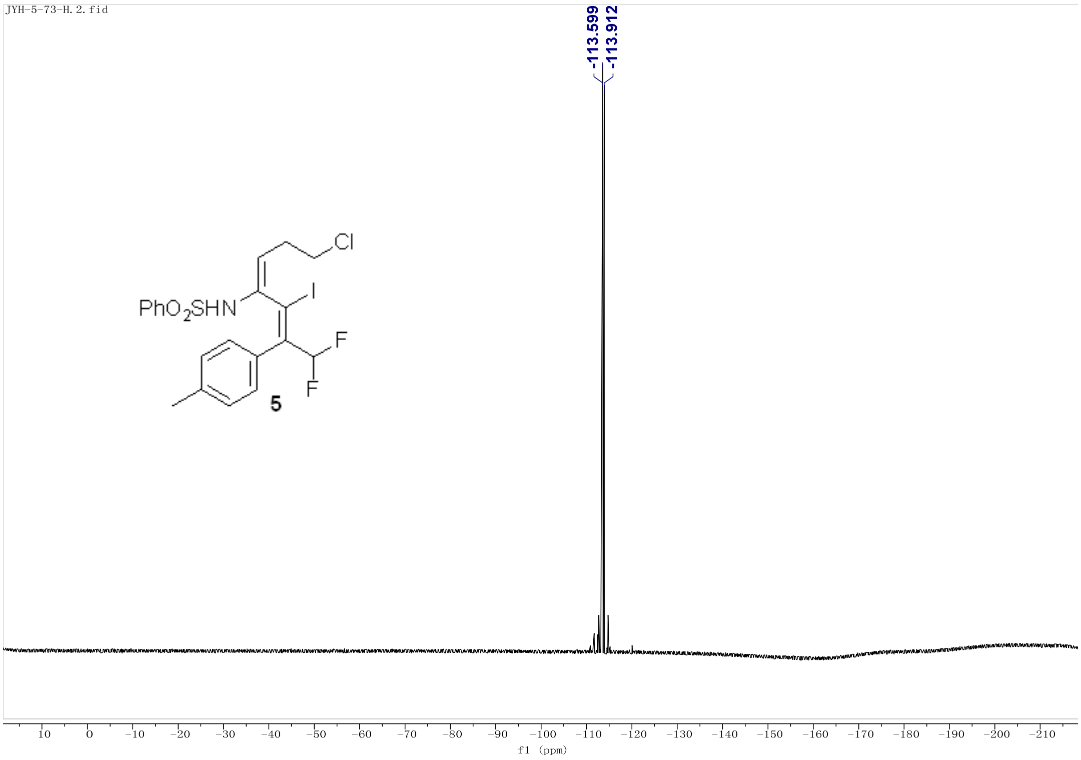

Supplementary Figure 169. ^{19}F NMR spectrum of compound 5.

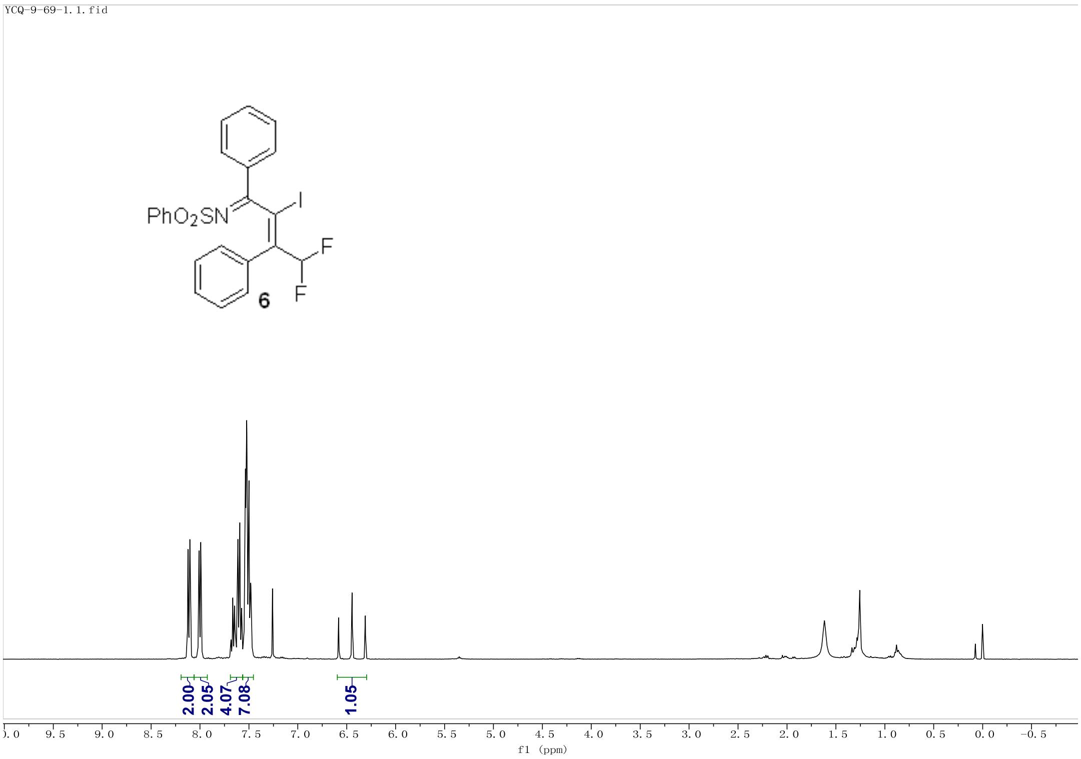

Supplementary Figure 170. **^{1}H NMR spectrum of compound 6.**

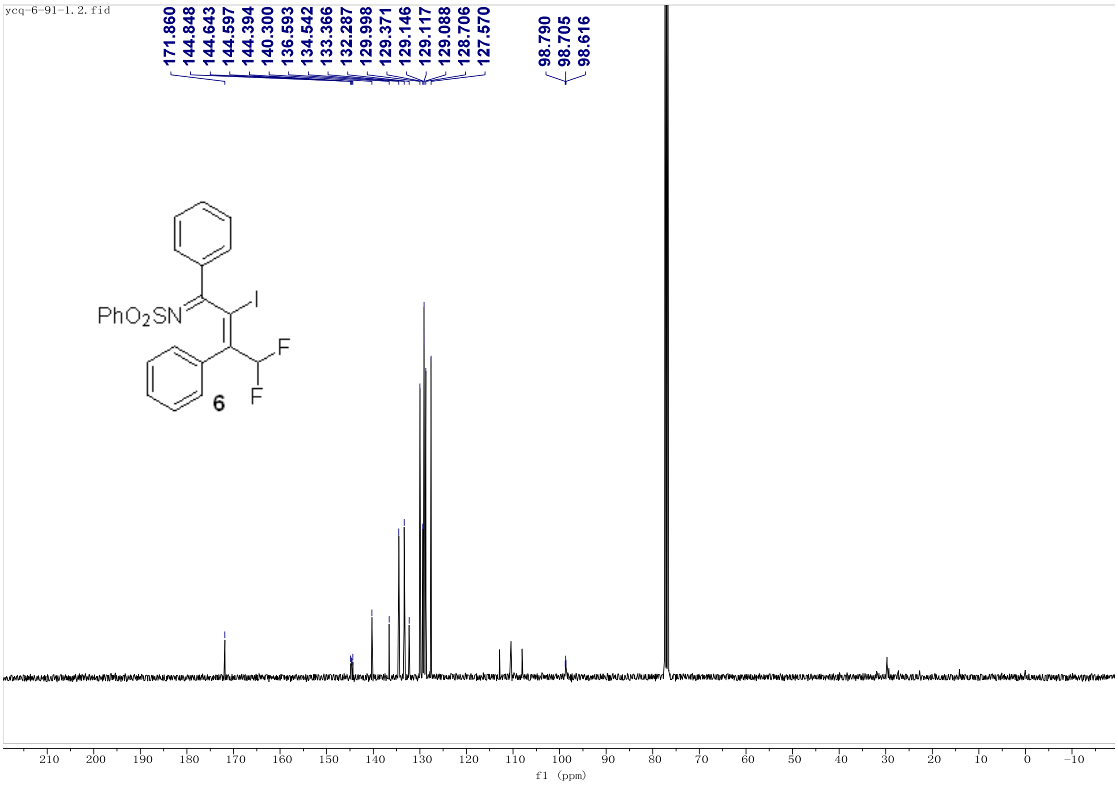

Supplementary Figure 171. **^{13}C NMR spectrum of compound 6.**

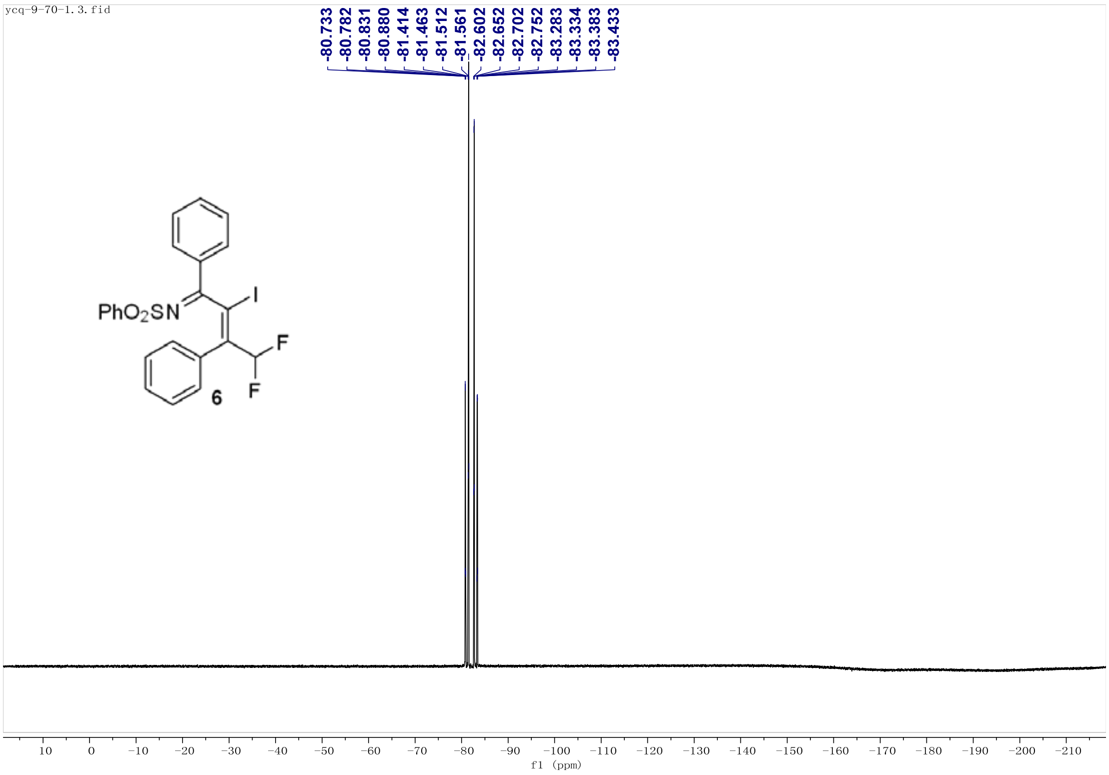

Supplementary Figure 172. ^{19}F NMR spectrum of compound 6.

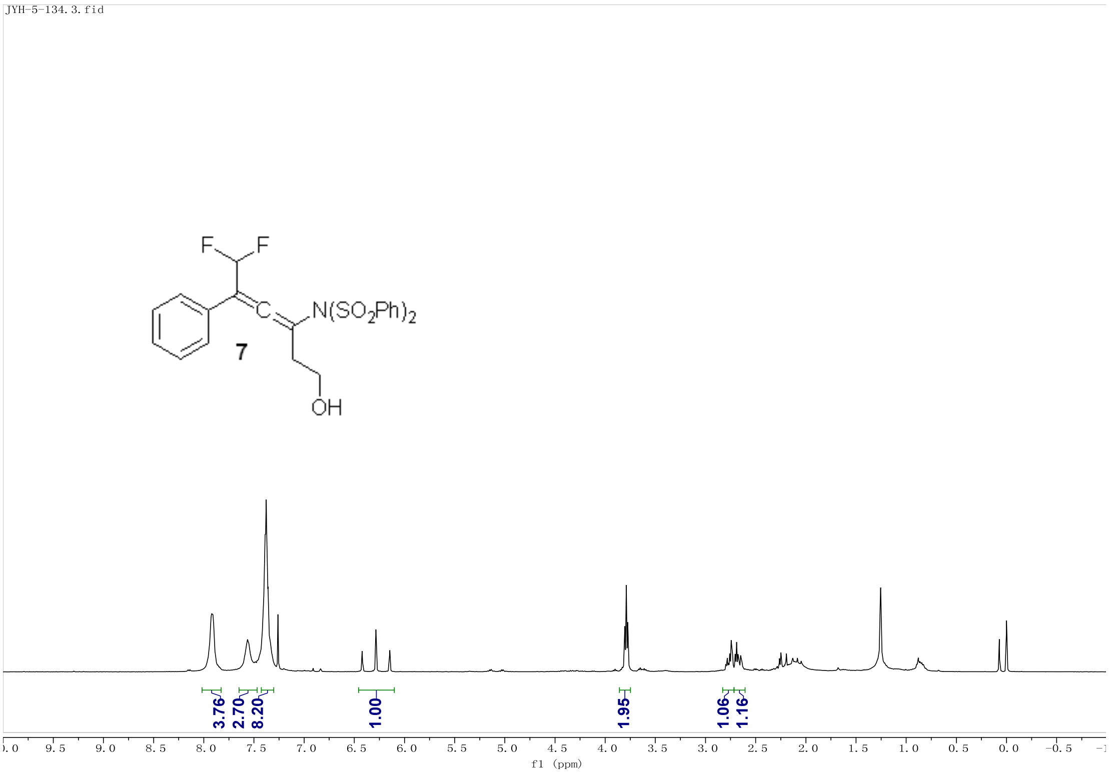

Supplementary Figure 173. ^{1}H NMR spectrum of compound 7.

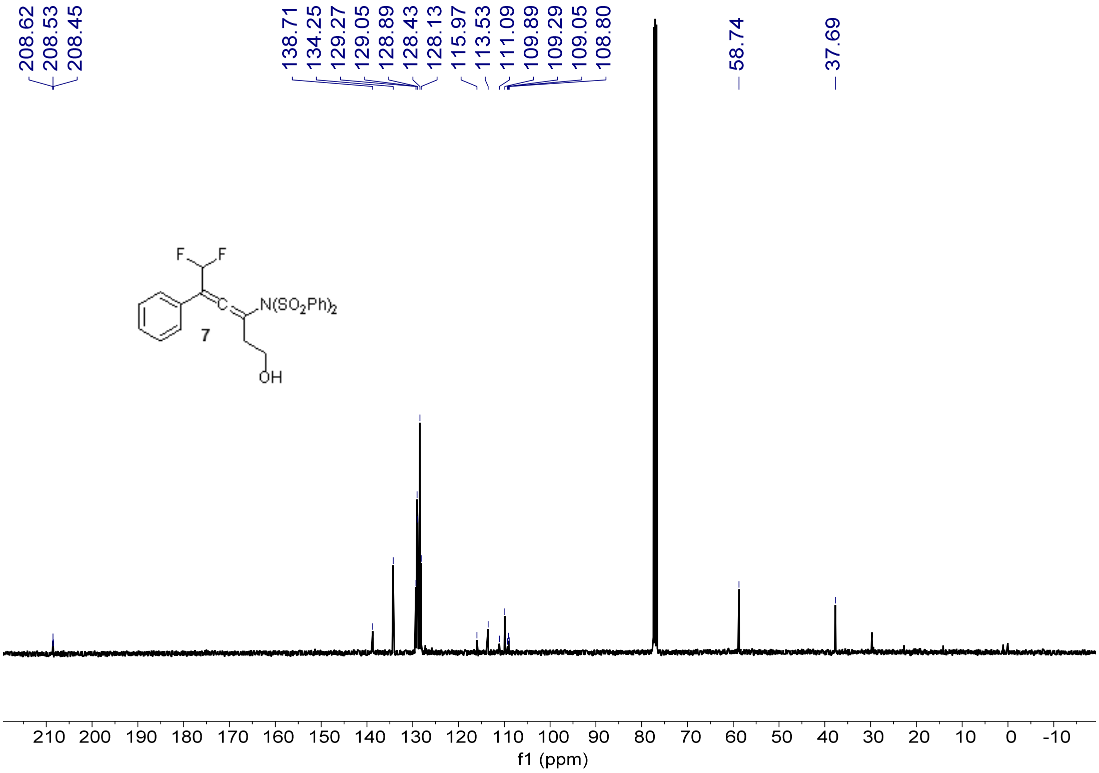

Supplementary Figure 174. **^{13}C NMR spectrum of compound 7.**

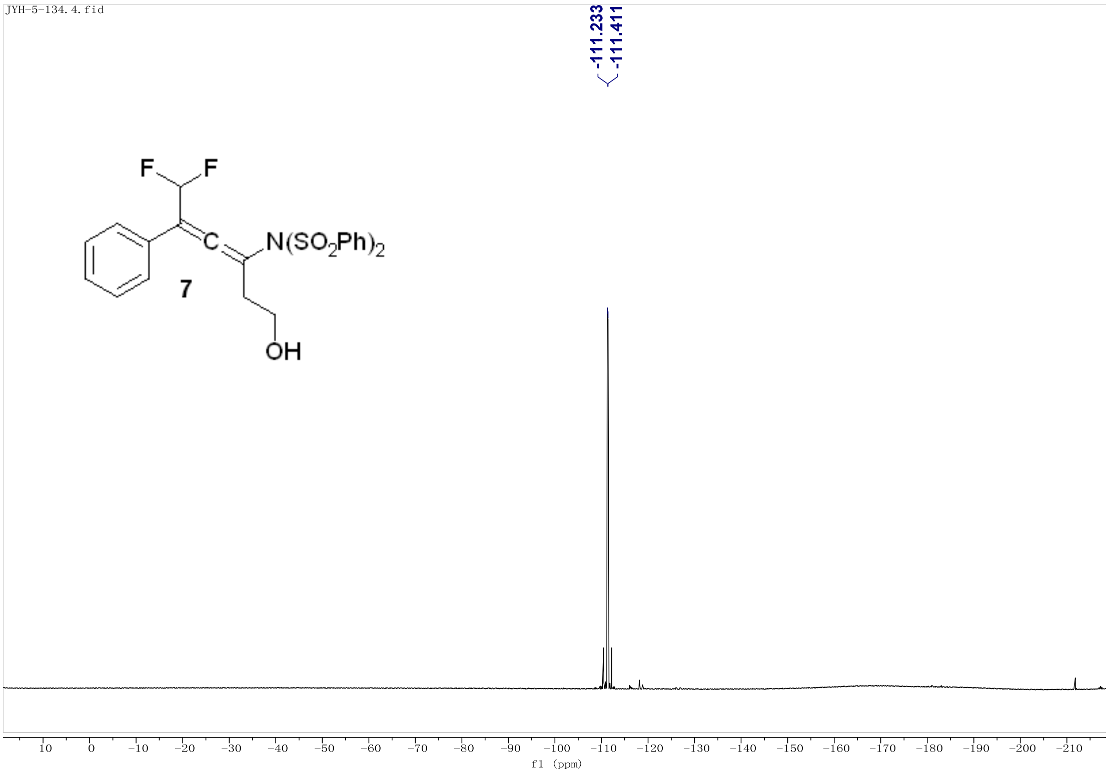

Supplementary Figure 175. **^{19}F NMR spectrum of compound 7.**

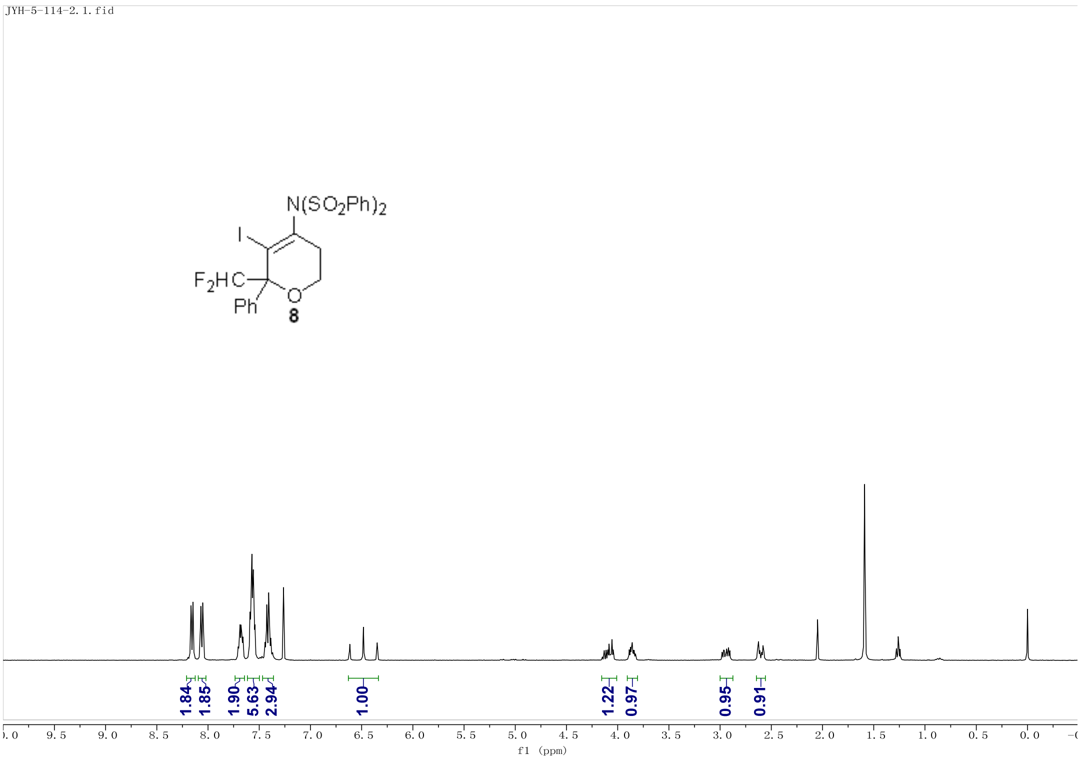

Supplementary Figure 176. ^{1}H **NMR spectrum of compound 8.**

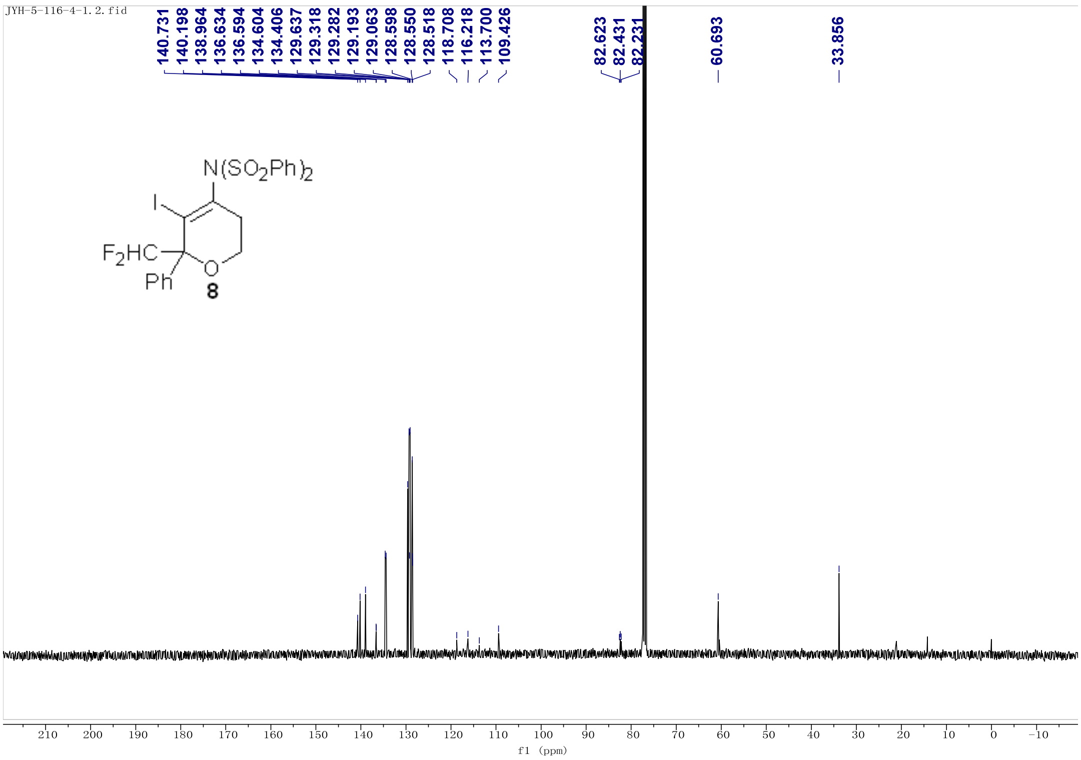

Supplementary Figure 177. ^{13}C **NMR spectrum of compound 8.**

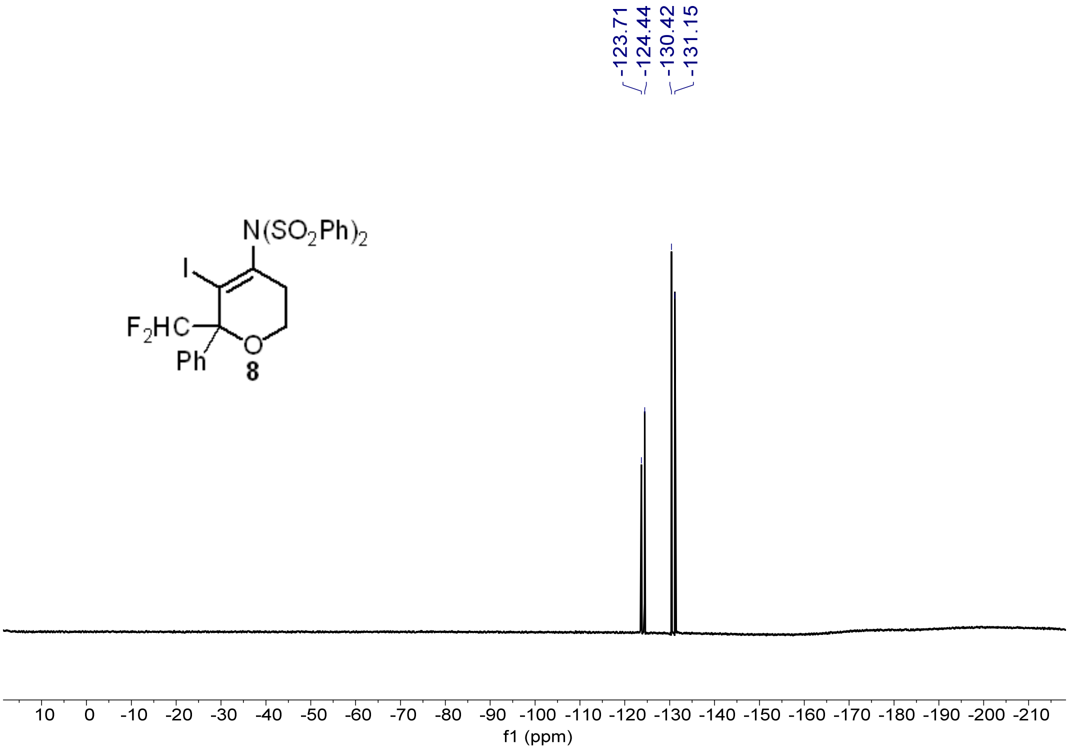

Supplementary Figure 178. ^{19}F **NMR spectrum of compound 8.**

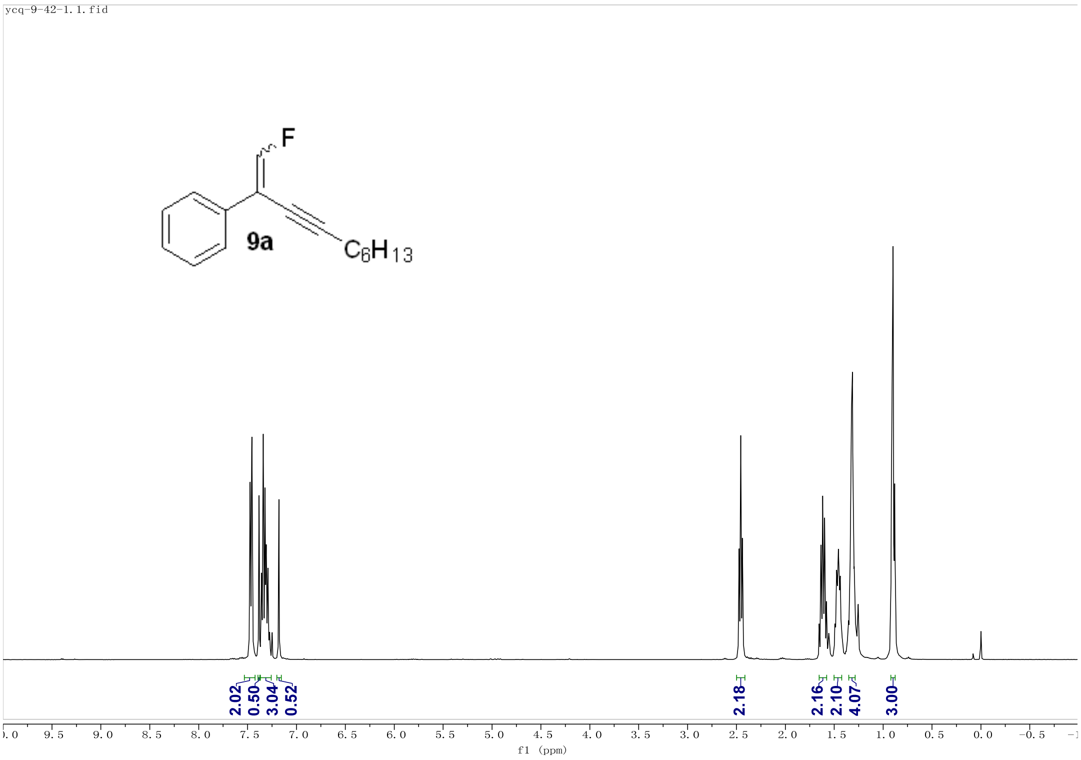

Supplementary Figure 179. ^{1}H **NMR spectrum of compound 9a.**

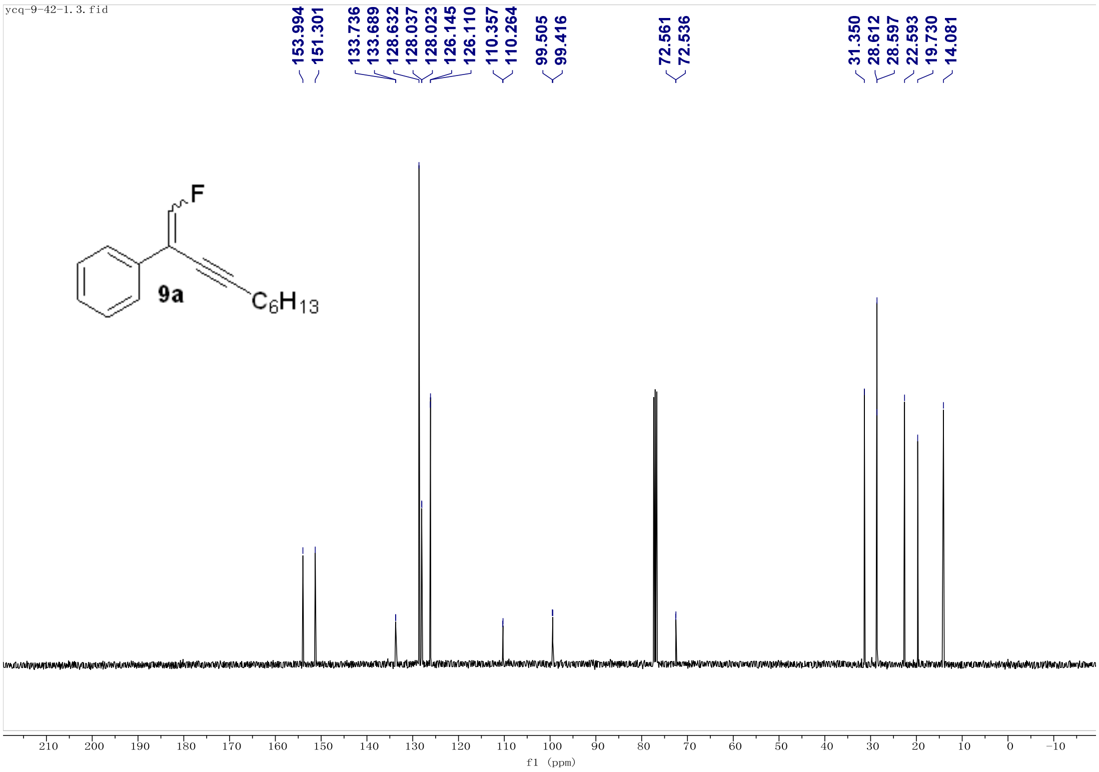

Supplementary Figure 180. ^{13}C **NMR spectrum of compound 9a.**

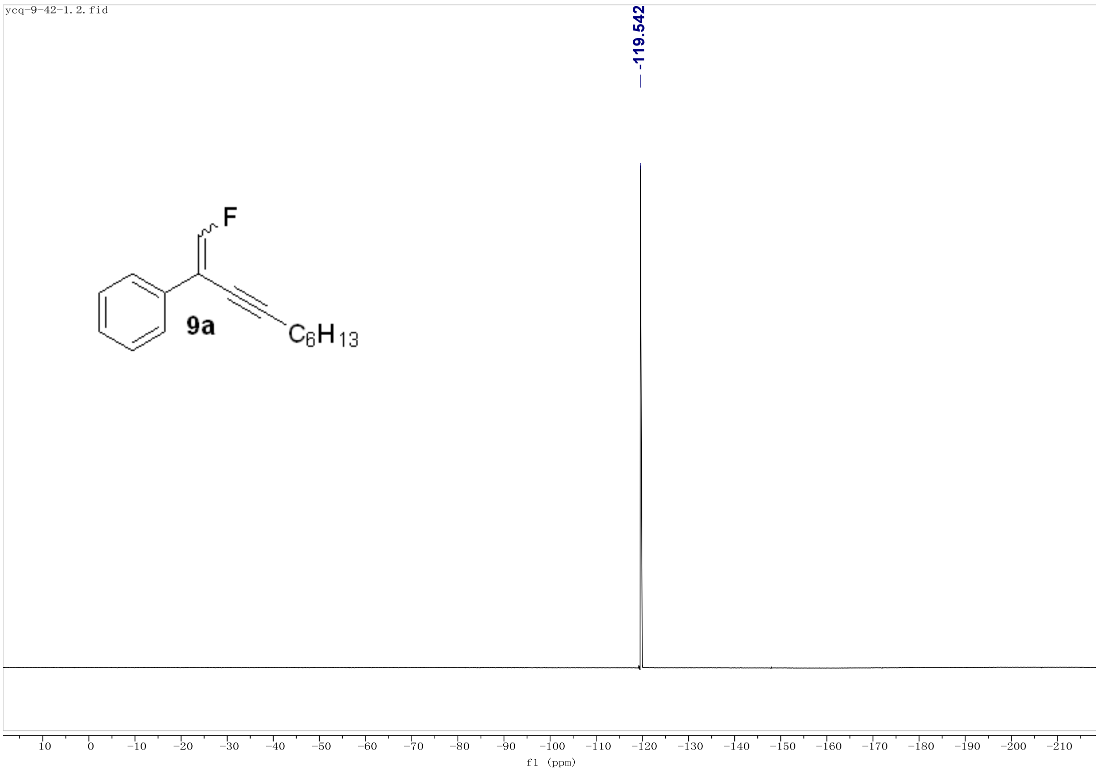

Supplementary Figure 181. ^{19}F **NMR spectrum of compound 9a.**

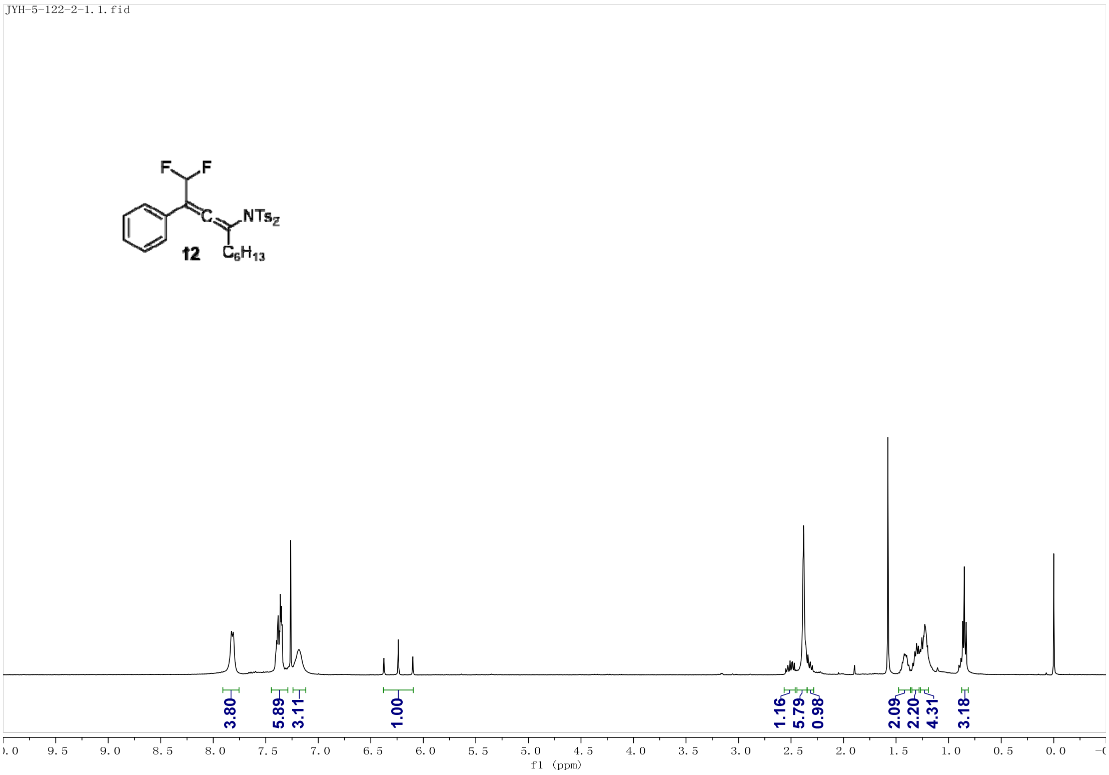

Supplementary Figure 182. **^{1}H NMR spectrum of compound 12.**

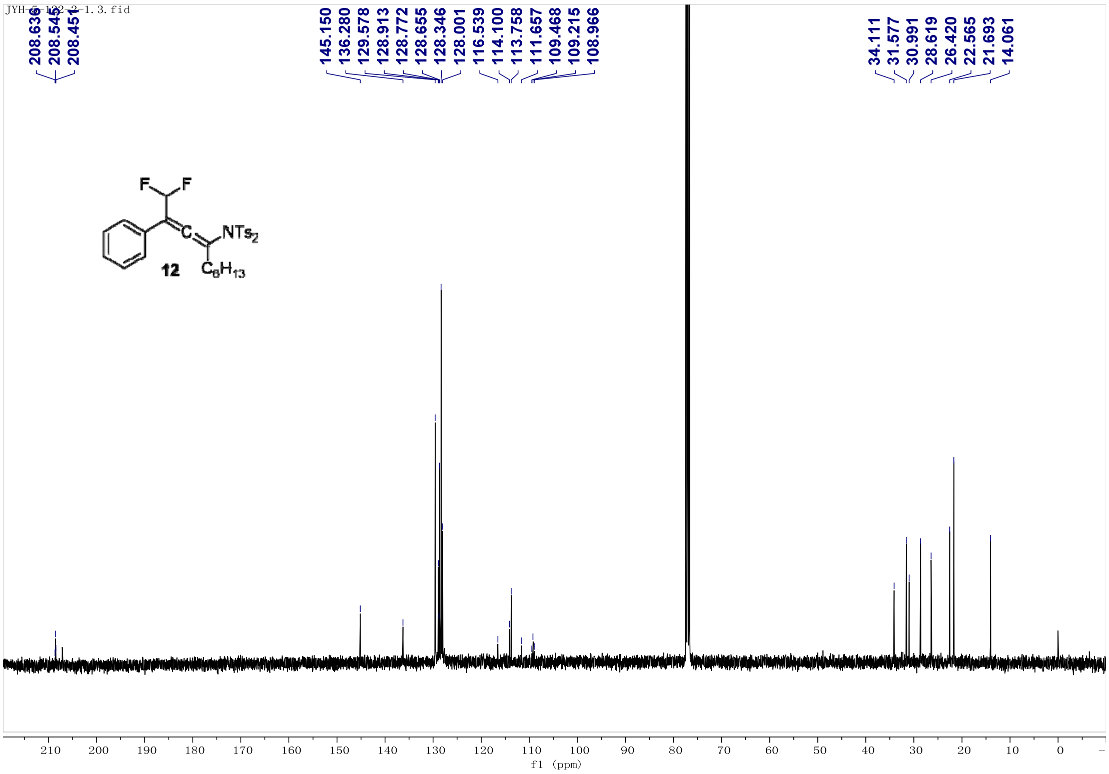

Supplementary Figure 183. **^{13}C NMR spectrum of compound 12.**

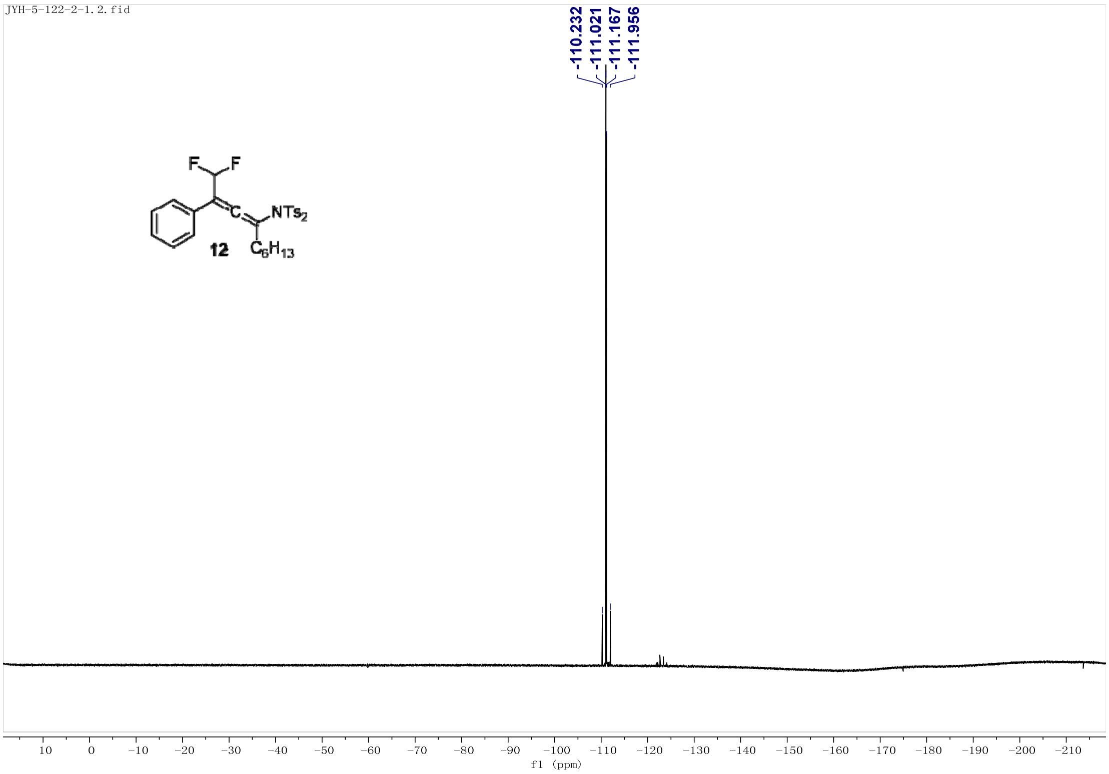

Supplementary Figure 184. **^{19}F NMR spectrum of compound 12.**

Supplementary References

1. Song, X. R., *et al.* Lewis acid mediated tandem reaction of propargylic alcohols to tetrazoles involving C-O- and C-C-bond cleavage reactions and a C-N-bond formation. *Chem. Eur. J.* **20**, 12046-12050 (2014).

2. Wakayama, M., Nemoto, H., Shibuya, M. Pyridoxal-mediated cycloaromatization of an enediyne model system. *Tetrahedron Lett.* **37**, 5397-5400 (1996).

3. Zhu, X., *et al.* Copper-Catalyzed Radical 1,4-Difunctionalization of 1,3-Enynes with Alkyl Diacyl Peroxides and N-Fluorobenzenesulfonimide. *J. Am. Chem. Soc.* **141**, 548-559 (2019).

4. Yan, W., Ye, X., Akhmedov, N. G., Petersen, J. L., Shi, X. 1,2,3-triazole: unique ligand in promoting iron-catalyzed propargyl alcohol dehydration. *Org. Lett.* **14**, 2358-2361 (2012).

5. Zatolochnaya, O. V., Gordeev, E. G., Jahier, C., Ananikov, V. P., Gevorgyan, V. Carboxylate switch between hydro- and carbopalladation pathways in regiodivergent dimerization of alkynes. *Chem. Eur. J.* **20**, 9578-9588 (2014).

6. Ye, C., Qian, B., Li, Y., Su, M., Li, D., Bao, H. Iron-Catalyzed Dehydrative Alkylation of Propargyl Alcohol with Alkyl Peroxides To Form Substituted 1,3-Enynes. *Org. Lett.* **20**, 3202-3205 (2018).

7. Tsukada, N., Ninomiya, S., Aoyama, Y., Inoue, Y. Palladium-catalyzed selective cross-addition of triisopropylsilylacetylene to internal and terminal unactivated alkynes. *Org. Lett.* **9**, 2919-2921 (2007).

8. Lee, C., Yang, W., Parr, R. G. Development of the Colle-Salvetti correlation-energy formula into a functional of the electron density. *Phys. Rev. B* **37**, 785-789 (1988).

9. Becke, A. D. Density-functional thermochemistry. III. The role of exact exchange. *J. Chem. Phys.* **98**, 5648-5652 (1993).

10. Grimme, S., Antony, J., Ehrlich, S., Krieg, H. A consistent and accurate ab initio parametrization of density functional dispersion correction (DFT-D) for the 94 elements H-Pu. *J. Chem. Phys.* **132**, 154104 (2010).

11. Grimme, S., Ehrlich, S., Goerigk, L. Effect of the damping function in dispersion corrected density functional theory. *J. Comput. Chem.* **32**, 1456-1465 (2011).

12. Weigend, F. Accurate Coulomb-fitting basis sets for H to Rn. *Phys. Chem. Chem. Phys.* **8**, 1057-1065 (2006).

13. Weigend, F., Ahlrichs, R. Balanced basis sets of split valence, triple zeta valence and quadruple zeta valence quality for H to Rn: Design and assessment of accuracy. *Phys. Chem. Chem. Phys.* **7**, 3297-3305 (2005).

14. Besalú, E., Bofill, J. M. On the automatic restricted-step rational-function-optimization method. *Theor. Chem. Acc.* **100**, 265-274 (1998).

15. Fukui, K. The path of chemical reactions - the IRC approach. *Acc. Chem. Res.* **14**, 363-368 (1981).

16. Marenich, A. V., Cramer, C. J., Truhlar, D. G. Universal Solvation Model Based on Solute Electron Density and on a Continuum Model of the Solvent Defined by the Bulk Dielectric Constant and Atomic Surface Tensions. *J. Phys. Chem. B* **113**, 6378-6396 (2009).

17. Foster, J. P., Weinhold, F. Natural hybrid orbitals. *J. Am. Chem. Soc.* **102**, 7211-7218 (1980).

18. Reed, A. E., Weinhold, F. Natural localized molecular orbitals. *J. Chem. Phys.* **83**, 1736-1740 (1985).

19. Reed, A. E., Weinstock, R. B., Weinhold, F. Natural population analysis. *J. Chem. Phys.* **83**, 735-746 (1985).

20. Reed, A. E., Curtiss, L. A., Weinhold, F. Intermolecular interactions from a natural bond orbital, donor-acceptor viewpoint. *Chem. Rev.* **88**, 899-926 (1988).

21. Frisch, M. J.; Trucks, G. W.; Schlegel, H. B.; Scuseria, G. E.; Robb, M. A.; Cheeseman, J. R.; Scalmani, G.; Barone, V.; Mennucci, B.; Petersson, G. A.; Nakatsuji, H.; Caricato, M.; Li, X.; Hratchian, H. P.; Izmaylov, A. F.; Bloino, J.; Zheng, G.; Sonnenberg, J. L.; Hada, M.; Ehara, M.; Toyota, K.; Fukuda, R.; Hasegawa, J.; Ishida, M.; Nakajima, T.; Honda, Y.; Kitao, O.; Nakai, H.; Vreven, T.; Montgomery Jr., J. A.; Peralta, J. E.; Ogliaro, F.; Bearpark, M. J.; Heyd, J.; Brothers, E. N.; Kudin, K. N.; Staroverov, V. N.; Kobayashi, R.; Normand, J.; Raghavachari, K.; Rendell, A. P.; Burant, J. C.; Iyengar, S. S.; Tomasi, J.; Cossi, M.; Rega, N.; Millam, N. J.; Klene, M.; Knox, J. E.; Cross, J. B.; Bakken, V.; Adamo, C.; Jaramillo, J.; Gomperts, R.; Stratmann, R. E.; Yazyev, O.; Austin, A. J.; Cammi, R.; Pomelli, C.; Ochterski, J. W.; Martin, R. L.; Morokuma, K.; Zakrzewski, V. G.; Voth, G. A.; Salvador, P.; Dannenberg, J. J.; Dapprich, S.; Daniels, A. D.; Farkas, Ö.; Foresman, J. B.; Ortiz, J. V.; Cioslowski, J.; Fox, D. J.; Gaussian 09, Revision D.01 ed.; Gaussian, Inc.: Wallingford, CT, USA, (2013).
