# Supplementary material for: Synthesis of difluoromethylated allenes through trifunctionalization of 1,3-enynes
Source: Nat Commun. 2020 Jan 21;11:416. doi: 10.1038/s41467-019-14254-3 (PMC6972827; doi:10.1038/s41467-019-14254-3)
Supplement: Supplementary file 2 — Source Data [file 41467_2019_14254_MOESM2_ESM.pdf]

Coordinate of optimized structures

Structure S1. **1**

E(B3LYP)sol = -503.964226598 E(B3LYP)gas = -503.403188727

6	-3.392857	-1.834552	0.049095
6	-2.071659	-2.010650	-0.371449
6	-1.194755	-0.924389	-0.412625
6	-1.622715	0.364808	-0.046744
6	-2.951826	0.524633	0.388635
6	-3.827250	-0.561159	0.432903
1	-1.718506	-3.001718	-0.668306
1	-0.162939	-1.069763	-0.738911
1	-3.299485	1.505175	0.720441
1	-4.853050	-0.414145	0.780960
6	-1.104554	2.800651	-0.285979
1	-2.162666	3.043626	-0.401400
6	-0.683887	1.525637	-0.122179
6	0.720501	1.238983	-0.031103
6	1.904225	0.972412	0.045958
6	3.318779	0.618584	0.139878
1	3.883690	1.150601	-0.648029
1	3.723545	0.990647	1.099672
6	3.584093	-0.895545	0.027202
1	3.012927	-1.416524	0.814265
1	3.181752	-1.256782	-0.934717
6	5.069443	-1.236151	0.138228
1	5.653724	-0.743339	-0.656999
1	5.484106	-0.906190	1.105576
1	-4.078062	-2.685217	0.087459
1	-0.390100	3.625002	-0.325391
1	5.239836	-2.320948	0.054468

Structure S2. NFSI

E(B3LYP)sol = -1715.50509779 E(B3LYP)gas = -1714.05449305

9	-0.731959	1.910549	1.020143
7	0.111865	0.809944	0.858081
16	-0.854123	-0.634640	1.239553
6	-2.337818	-0.472046	0.269251
6	-3.369583	0.331267	0.768017
6	-2.432907	-1.139531	-0.954927
6	-4.528928	0.473043	0.004657

1	-3.259695	0.823436	1.734949
6	-3.604566	-0.994115	-1.699461
1	-1.601619	-1.746714	-1.310478
6	-4.644762	-0.187953	-1.223912
1	-5.346133	1.097777	0.372172
1	-3.702388	-1.508723	-2.657917
1	-5.556310	-0.074565	-1.816012
8	-1.193323	-0.475391	2.644919
8	-0.019059	-1.724745	0.742837
16	0.854455	0.949298	-0.796574
8	0.119343	0.141314	-1.767225
8	1.019002	2.386411	-0.958052
6	2.419685	0.180052	-0.440433
6	3.430350	0.970357	0.116163
6	2.603079	-1.170979	-0.744434
6	4.665669	0.377830	0.380462
1	3.245333	2.024953	0.326882
6	3.846296	-1.746032	-0.474027
1	1.782969	-1.751438	-1.165330
6	4.871000	-0.975724	0.086625
1	5.471032	0.974632	0.814505
1	4.012952	-2.801757	-0.699138
1	5.840328	-1.434391	0.297140

Structure S3. **TS1**

E(B3LYP)sol = -2219.45282922 E(B3LYP)gas = -2217.44858796

9	-0.018588	-0.478164	-1.353347
7	1.524361	-0.966077	-0.490092
16	0.979993	-1.568538	1.020582
6	-0.273537	-0.454894	1.642892
6	-1.597796	-0.663454	1.247826
6	0.083902	0.570049	2.521419
6	-2.585591	0.189532	1.739116
1	-1.836710	-1.472886	0.558638
6	-0.917298	1.414069	3.006174
1	1.126837	0.703615	2.803356
6	-2.246372	1.227360	2.613918
1	-3.620721	0.054565	1.419143
1	-0.655170	2.226757	3.687536
1	-3.022762	1.901073	2.983586
8	0.334254	-2.837410	0.704052
8	2.149993	-1.507277	1.907787

16	2.369675	0.529822	-0.597066
8	2.075846	1.427754	0.527927
8	2.153871	1.007187	-1.966648
6	4.049443	-0.060485	-0.453736
6	4.719813	-0.448686	-1.618082
6	4.651309	-0.111892	0.806355
6	6.035671	-0.901541	-1.510930
1	4.212820	-0.383309	-2.581977
6	5.968259	-0.566200	0.894998
1	4.087300	0.181231	1.690944
6	6.657084	-0.960366	-0.257804
1	6.578581	-1.207056	-2.408490
1	6.457024	-0.616880	1.870679
1	7.686920	-1.317827	-0.179103
6	-2.149225	4.200189	0.446875
6	-3.364570	3.614264	0.069496
6	-3.370979	2.456723	-0.703936
6	-2.159818	1.879360	-1.142971
6	-0.942397	2.487899	-0.768196
6	-0.941807	3.628679	0.030152
1	-4.310091	4.058433	0.390632
1	-4.317473	1.988074	-0.981362
1	0.007374	2.038461	-1.048218
1	0.013948	4.054177	0.343526
6	-1.073002	0.241069	-2.702232
1	-0.310027	0.963938	-2.989977
6	-2.162207	0.632016	-1.919469
6	-3.246989	-0.265211	-1.760487
6	-4.144302	-1.067563	-1.565053
6	-5.154933	-2.069938	-1.251905
1	-5.323359	-2.714382	-2.133358
1	-6.118297	-1.569199	-1.045375
6	-4.759860	-2.946242	-0.040434
1	-4.590374	-2.293821	0.832604
1	-3.792829	-3.431975	-0.251997
6	-5.821860	-3.995874	0.281468
1	-5.984444	-4.675363	-0.571612
1	-6.790400	-3.526565	0.522370
1	-2.144902	5.096206	1.072860
1	-1.158366	-0.657753	-3.312683
1	-5.522152	-4.609255	1.145120

Frequencies -- -501.6486

Red. masses -- 15.9062

Frc consts -- 2.3584
IR Inten -- 1517.5415

Optimized with SMD(toluene) method

E(B3LYP)sol = -2219.45376628 E(B3LYP)sol = -2217.47783496

9	-0.080364	-0.433138	-1.420575
7	-1.620789	-0.937546	-0.607968
16	-1.103490	-1.725195	0.823775
6	0.159117	-0.721206	1.592385
6	1.481554	-0.877523	1.167283
6	-0.191312	0.167505	2.611501
6	2.475654	-0.112931	1.777718
1	1.717607	-1.578141	0.366989
6	0.815831	0.923063	3.215103
1	-1.232081	0.265073	2.916162
6	2.143757	0.785803	2.797712
1	3.510631	-0.212728	1.445465
1	0.559499	1.625719	4.011480
1	2.926076	1.387695	3.266397
8	-0.480874	-2.961595	0.358164
8	-2.285133	-1.759407	1.697760
16	-2.439868	0.577293	-0.539497
8	-2.103640	1.344651	0.666636
8	-2.234796	1.186874	-1.857601
6	-4.128380	0.007496	-0.425293
6	-4.796280	-0.340759	-1.604184
6	-4.741828	-0.063105	0.828163
6	-6.120957	-0.770871	-1.517890
1	-4.284394	-0.266656	-2.564939
6	-6.068222	-0.493007	0.895908
1	-4.184468	0.204389	1.725200
6	-6.754513	-0.845876	-0.271545
1	-6.661740	-1.044532	-2.426958
1	-6.566850	-0.554616	1.866060
1	-7.792239	-1.183385	-0.209901
6	2.101293	4.112413	0.632768
6	3.315768	3.491432	0.314944
6	3.331463	2.369215	-0.509531
6	2.132913	1.858658	-1.053966
6	0.916013	2.499141	-0.731734
6	0.904478	3.608371	0.110070
1	4.253267	3.881402	0.719287

1	4.278200	1.879023	-0.744854
1	-0.028998	2.097831	-1.092922
1	-0.051230	4.066491	0.374550
6	1.081313	0.316231	-2.730830
1	0.340302	1.063747	-3.010913
6	2.144680	0.651008	-1.893658
6	3.231648	-0.251861	-1.774875
6	4.150595	-1.044737	-1.651597
6	5.238416	-1.992522	-1.450193
1	4.916490	-2.987972	-1.805555
1	6.087532	-1.702044	-2.097014
6	5.712440	-2.086829	0.014605
1	6.042670	-1.088298	0.348634
1	4.854167	-2.361720	0.650414
6	6.840434	-3.100359	0.190463
1	6.521278	-4.112507	-0.109959
1	7.719587	-2.834444	-0.420539
1	2.087368	4.982361	1.294517
1	1.162748	-0.563331	-3.369793
1	7.167400	-3.152545	1.241073

Frequencies -- -506.0339
Red. masses -- 15.9778
Frc consts -- 2.4106
IR Inten -- 2890.9865

Structure S4. 9

E(B3LYP)sol = -603.243725228 E(B3LYP)gas = -602.560905248

9	0.280491	3.491427	-0.258442
6	3.403148	-2.143109	0.014671
6	2.104920	-2.304695	-0.479293
6	1.215613	-1.228581	-0.490710
6	1.608583	0.035255	-0.014152
6	2.910493	0.179999	0.500910
6	3.800239	-0.896068	0.507368
1	1.780716	-3.277106	-0.859168
1	0.202366	-1.359889	-0.876457
1	3.222839	1.135592	0.928144
1	4.805342	-0.762801	0.915941
6	1.102258	2.442373	-0.234954
6	0.654612	1.180732	-0.058628
6	-0.747424	0.923230	0.056343
6	-1.931403	0.666265	0.154337

6	-3.348515	0.330757	0.266967
1	-3.700829	0.576850	1.286118
1	-3.932309	0.975901	-0.415263
6	-3.657052	-1.148836	-0.035796
1	-3.306923	-1.384201	-1.055378
1	-3.065660	-1.782808	0.646514
6	-5.144388	-1.471908	0.098512
1	-5.507283	-1.267785	1.119863
1	-5.750088	-0.864539	-0.594869
1	4.097647	-2.986856	0.027150
1	2.149468	2.714153	-0.387971
1	-5.346210	-2.531825	-0.122104

Structure S5. DBSI

E(B3LYP)sol = -1616.31726298 E(B3LYP)gas = -1614.98574751

7	0.000060	0.000108	-1.067917
16	0.934213	-1.214584	-0.320278
6	2.496298	-0.422592	0.043081
6	3.566425	-0.622884	-0.833402
6	2.609887	0.366894	1.191638
6	4.786535	-0.003515	-0.551454
1	3.439061	-1.261962	-1.708677
6	3.835563	0.981067	1.455170
1	1.749711	0.507336	1.845748
6	4.919092	0.797208	0.588023
1	5.636313	-0.150878	-1.222140
1	3.944409	1.606502	2.344233
1	5.875181	1.280712	0.803659
8	1.143963	-2.197778	-1.379836
8	0.275671	-1.540921	0.940761
16	-0.934343	1.214775	-0.320497
8	-0.275861	1.541533	0.940460
8	-1.144289	2.197672	-1.380294
6	-2.496277	0.422580	0.043053
6	-3.566315	0.622128	-0.833718
6	-2.609850	-0.366313	1.192009
6	-4.786293	0.002573	-0.551657
1	-3.438967	1.260780	-1.709307
6	-3.835412	-0.980673	1.455665
1	-1.749752	-0.506172	1.846344
6	-4.918833	-0.797575	0.588234
1	-5.635998	0.149331	-1.222568

1	-3.944236	-1.605657	2.345048
1	-5.874830	-1.281225	0.803948
1	-0.000025	-0.000024	-2.087049

Structure S6. TS2

E(B3LYP)sol = -2318.73151498 E(B3LYP)gas = -2316.60797658

9	1.076068	-1.421630	-3.169405
7	-1.622239	-1.016398	-0.203284
16	-1.128789	-1.333633	1.414085
6	0.153069	-0.160509	1.833639
6	1.475470	-0.486055	1.516864
6	-0.182095	1.028302	2.484650
6	2.485851	0.412072	1.857886
1	1.694744	-1.421636	1.002427
6	0.841927	1.916782	2.819767
1	-1.224541	1.249388	2.707754
6	2.169713	1.611413	2.506005
1	3.521126	0.179678	1.600581
1	0.598879	2.855984	3.321943
1	2.964128	2.317544	2.757996
8	-0.525350	-2.659542	1.363513
8	-2.314140	-1.063335	2.239110
16	-2.453122	0.439018	-0.605674
8	-2.182339	1.528053	0.341876
8	-2.192324	0.654026	-2.033602
6	-4.137746	-0.115167	-0.405101
6	-4.769706	-0.731048	-1.490307
6	-4.780475	0.084278	0.819681
6	-6.089699	-1.157704	-1.338138
1	-4.230685	-0.859088	-2.430238
6	-6.101189	-0.347293	0.953333
1	-4.244335	0.549831	1.645793
6	-6.752412	-0.966397	-0.119750
1	-6.603425	-1.638997	-2.173622
1	-6.622353	-0.203561	1.902580
1	-7.785387	-1.304565	-0.005329
6	2.108147	4.156641	-0.120236
6	3.311580	3.490283	-0.382758
6	3.297812	2.217884	-0.947645
6	2.077219	1.594175	-1.286405
6	0.871584	2.280837	-1.026938
6	0.891991	3.543603	-0.440747

1	4.265444	3.963485	-0.136475
1	4.236128	1.692964	-1.136851
1	-0.087817	1.812414	-1.228685
1	-0.056781	4.034734	-0.214140
6	0.987448	-0.246319	-2.579634
9	-0.045781	-0.659773	-1.069124
6	2.081184	0.240964	-1.858848
6	3.166124	-0.627222	-1.603849
6	4.085735	-1.374844	-1.318737
6	5.162100	-2.264245	-0.903522
1	4.897198	-3.302156	-1.174197
1	6.074597	-2.024359	-1.480920
6	5.469477	-2.179111	0.606881
1	5.740951	-1.139029	0.856424
1	4.547944	-2.401936	1.170418
6	6.587845	-3.132216	1.024342
1	6.321512	-4.179682	0.806397
1	7.525559	-2.909101	0.488478
1	2.119299	5.147341	0.341188
1	0.196868	0.390997	-2.979705
1	6.792108	-3.057215	2.103570

Frequencies -- -501.0049
Red. masses -- 16.0428
Frc consts -- 2.3725
IR Inten -- 1557.9333

Structure S7. int1

E(B3LYP)sol = -2318.81659704 E(B3LYP)gas = -2316.69076495

9	0.128599	-2.089540	-3.171093
7	-1.240435	0.075898	-0.136722
16	-0.309418	-0.074344	1.167182
6	0.744671	1.395917	1.276698
6	2.101544	1.203047	1.562459
6	0.210390	2.677962	1.127148
6	2.938856	2.314673	1.690642
1	2.486706	0.188139	1.669979
6	1.061007	3.780805	1.250569
1	-0.852559	2.803873	0.909521
6	2.420977	3.603873	1.531012
1	3.999490	2.172037	1.914851
1	0.653619	4.787856	1.129731
1	3.076707	4.472987	1.629463

8	0.645353	-1.170814	0.816454
8	-1.026291	-0.197801	2.443587
16	-2.696701	0.826417	-0.242201
8	-2.788076	2.025627	0.612752
8	-2.946547	0.972310	-1.688801
6	-3.884588	-0.363157	0.391672
6	-4.606834	-1.145477	-0.512228
6	-4.056303	-0.481741	1.773941
6	-5.521620	-2.078306	-0.015411
1	-4.452694	-1.007555	-1.583652
6	-4.971655	-1.419858	2.255068
1	-3.463588	0.135353	2.449301
6	-5.702475	-2.215874	1.364814
1	-6.097238	-2.696100	-0.709353
1	-5.114565	-1.529852	3.332874
1	-6.417895	-2.947158	1.749951
6	0.841714	3.049417	-2.312632
6	2.114521	2.616619	-1.879408
6	2.344467	1.274836	-1.669562
6	1.310471	0.313680	-1.907082
6	0.039633	0.775776	-2.370742
6	-0.182832	2.137654	-2.561132
1	2.900318	3.346139	-1.678530
1	3.310453	0.931015	-1.300007
1	-0.764803	0.085054	-2.598685
1	-1.179300	2.465128	-2.858543
6	0.403573	-2.074303	-1.831449
9	0.806467	-3.309155	-1.479093
6	1.515466	-1.050567	-1.587554
6	2.733956	-1.531509	-1.107858
6	3.759631	-1.962695	-0.605813
6	4.933049	-2.437503	0.103984
1	4.953294	-3.541178	0.086144
1	5.845918	-2.091315	-0.413733
6	4.944763	-1.935589	1.570464
1	4.935984	-0.832623	1.567127
1	4.008740	-2.252058	2.058694
6	6.156018	-2.456755	2.340970
1	6.164217	-3.558476	2.375476
1	7.100542	-2.129448	1.875623
1	0.658186	4.118340	-2.449490
1	-0.499543	-1.786609	-1.269917
1	6.147473	-2.089889	3.378832

Structure S8. **int2**

E(B3LYP)sol = -3935.15681299 E(B3LYP)gas = -3931.73086195

1	-0.357961	-0.524397	0.010519
7	0.157682	-1.455765	-0.213985
16	-0.962907	-2.746106	-0.250005
6	-2.163632	-2.337754	-1.494965
6	-3.297338	-1.619034	-1.110292
6	-1.897624	-2.653517	-2.829447
6	-4.181149	-1.183898	-2.096522
1	-3.470661	-1.386010	-0.063202
6	-2.798169	-2.220643	-3.801950
1	-0.993787	-3.197618	-3.097774
6	-3.929149	-1.481285	-3.438388
1	-5.050963	-0.590828	-1.809425
1	-2.604743	-2.446510	-4.853123
1	-4.615441	-1.125869	-4.211135
8	-1.615073	-2.669437	1.065623
8	-0.221394	-3.948478	-0.640137
16	1.369503	-1.234433	-1.382010
8	0.946335	-1.628094	-2.722989
8	1.856535	0.129404	-1.107078
6	2.653231	-2.388830	-0.885185
6	3.676543	-1.934307	-0.054103
6	2.620254	-3.704854	-1.353665
6	4.676620	-2.821953	0.346024
1	3.687129	-0.892818	0.254835
6	3.621628	-4.586725	-0.940806
1	1.814734	-4.030811	-2.008834
6	4.643748	-4.150792	-0.089758
1	5.484070	-2.473711	0.995237
1	3.602212	-5.622331	-1.287966
1	5.422400	-4.848906	0.227717
6	6.095665	1.987783	-0.389923
6	5.938206	1.158497	0.731498
6	4.693325	1.042528	1.331791
6	3.585815	1.788658	0.843891
6	3.765891	2.637344	-0.279390
6	5.009543	2.714200	-0.894418
1	6.790539	0.602380	1.127335
1	4.558306	0.401560	2.205268
1	2.918214	3.177110	-0.695025
1	5.134001	3.341835	-1.779268

6	1.230465	2.716622	1.503511
1	0.365704	2.430605	0.882797
6	2.300335	1.617347	1.462900
6	1.941427	0.446781	2.077193
6	1.416672	-0.590921	2.486900
6	0.766476	-1.774716	2.958712
1	0.636664	-1.652300	4.053956
1	-0.264428	-1.711514	2.557908
6	1.429005	-3.114554	2.595577
1	1.533246	-3.161580	1.502307
1	2.449285	-3.150909	3.013502
6	0.593747	-4.298599	3.077133
1	0.454012	-4.279993	4.171210
1	-0.397198	-4.279211	2.600052
1	7.072843	2.065249	-0.873316
1	1.080627	-5.249743	2.812889
9	0.854381	2.866523	2.788805
7	-1.294197	0.780753	0.210812
16	-1.967256	1.271695	1.634886
6	-3.649430	0.641715	1.704998
6	-4.699011	1.406534	1.189870
6	-3.866747	-0.619009	2.268894
6	-5.993098	0.882191	1.226352
1	-4.492994	2.381574	0.751781
6	-5.166216	-1.131707	2.297488
1	-3.024637	-1.192963	2.653604
6	-6.227062	-0.384401	1.774614
1	-6.823201	1.466573	0.821796
1	-5.347068	-2.120637	2.725400
1	-7.241951	-0.789941	1.795791
8	-2.043654	2.743877	1.704788
8	-1.210381	0.555938	2.678332
16	-1.720147	1.434303	-1.251781
8	-1.261473	0.459202	-2.251191
8	-3.117870	1.895901	-1.268457
6	-0.684662	2.895525	-1.485051
6	-1.053233	4.112878	-0.902913
6	0.457247	2.779008	-2.283110
6	-0.249139	5.234185	-1.119518
1	-1.941873	4.164016	-0.274124
6	1.241086	3.915888	-2.506640
1	0.717091	1.809728	-2.708743
6	0.891811	5.139365	-1.924301
1	-0.518023	6.188230	-0.659858

1	2.125876	3.843243	-3.145211
1	1.509845	6.024067	-2.098848
9	1.724947	3.903075	1.072702

Structure S9. TS3

E(B3LYP)sol = -3935.14120414 E(B3LYP)gas = -3931.72289517

1	0.451249	-0.401677	0.027224
7	-0.142944	-1.373544	0.138263
16	1.063120	-2.673615	0.135614
6	2.250145	-2.260281	1.390022
6	3.335894	-1.458969	1.028654
6	2.064699	-2.723194	2.695841
6	4.243999	-1.078795	2.014592
1	3.457347	-1.124892	0.003026
6	2.990362	-2.343713	3.666201
1	1.208415	-3.346471	2.943662
6	4.067220	-1.515073	3.330127
1	5.073074	-0.421594	1.748552
1	2.860861	-2.685904	4.695382
1	4.773004	-1.203927	4.104331
8	1.699592	-2.535649	-1.176322
8	0.366907	-3.909703	0.500015
16	-1.286242	-1.296497	1.461423
8	-0.693636	-1.851029	2.672413
8	-1.791527	0.078416	1.409403
6	-2.614260	-2.395778	0.964138
6	-3.751274	-1.838612	0.377780
6	-2.517177	-3.765271	1.228564
6	-4.800813	-2.678660	0.001570
1	-3.814605	-0.762517	0.236548
6	-3.571943	-4.594666	0.841770
1	-1.626381	-4.169704	1.704880
6	-4.706724	-4.056542	0.223375
1	-5.694714	-2.249527	-0.457432
1	-3.506703	-5.668949	1.028767
1	-5.526780	-4.714445	-0.075105
6	-6.268848	1.877197	0.038803
6	-5.923064	1.019689	-1.012747
6	-4.594189	0.915390	-1.415298
6	-3.593151	1.693144	-0.791013
6	-3.954143	2.566929	0.256570
6	-5.281846	2.640139	0.673058

1	-6.692226	0.432003	-1.519415
1	-4.316057	0.246304	-2.231940
1	-3.185597	3.145026	0.764940
1	-5.548798	3.298892	1.502567
6	-1.309468	2.754677	-1.496404
1	-0.280558	2.623988	-1.142287
6	-2.199668	1.535582	-1.213954
6	-1.698410	0.323950	-1.503378
6	-1.215028	-0.803303	-1.765717
6	-0.855399	-1.892518	-2.651189
1	-1.179679	-1.528245	-3.647245
1	0.242332	-1.941189	-2.692996
6	-1.476123	-3.261108	-2.338681
1	-1.248826	-3.527265	-1.298057
1	-2.574843	-3.192177	-2.407760
6	-0.931252	-4.346215	-3.263988
1	-1.123160	-4.114802	-4.325189
1	0.157554	-4.448232	-3.129437
1	-7.309740	1.951346	0.363236
1	-1.391543	-5.321200	-3.041764
9	-1.313805	2.926288	-2.841436
7	1.322666	0.790528	-0.180222
16	1.950931	1.291269	-1.630092
6	3.630297	0.666130	-1.760345
6	4.685071	1.385117	-1.189934
6	3.841287	-0.543403	-2.429065
6	5.976441	0.859950	-1.273110
1	4.485852	2.321760	-0.672357
6	5.138940	-1.055796	-2.504449
1	2.995213	-1.076815	-2.859488
6	6.203545	-0.358703	-1.923954
1	6.810318	1.406997	-0.826379
1	5.315537	-2.005285	-3.015281
1	7.216688	-0.764983	-1.981194
8	2.030921	2.763913	-1.672610
8	1.160381	0.596390	-2.659120
16	1.726689	1.488482	1.274661
8	1.269876	0.515819	2.278330
8	3.122950	1.952427	1.287752
6	0.685053	2.946413	1.466605
6	1.073372	4.160156	0.890007
6	-0.477257	2.835909	2.235333
6	0.265747	5.283357	1.079609
1	1.981426	4.209203	0.290184

6	-1.265710	3.974467	2.429304
1	-0.749220	1.871780	2.663731
6	-0.898497	5.193459	1.850551
1	0.549803	6.235032	0.624242
1	-2.168711	3.905995	3.041819
1	-1.521832	6.078865	1.999960
9	-1.826130	3.875765	-0.937015

Frequencies -- -230.2572

Red. masses -- 8.1982

Frc consts -- 0.2561

IR Inten -- 350.3315

Structure S10. 4

E(B3LYP)sol = -2318.87640424 E(B3LYP)gas = -2316.76706633

7	0.998489	-0.244808	0.348720
16	1.305118	-1.937844	0.322462
6	-0.246455	-2.660339	-0.208003
6	-0.850858	-3.613051	0.615707
6	-0.790691	-2.286053	-1.441590
6	-2.043176	-4.206081	0.187821
1	-0.393916	-3.870844	1.572195
6	-1.986543	-2.879984	-1.845713
1	-0.302143	-1.524277	-2.052224
6	-2.609983	-3.837073	-1.036040
1	-2.533076	-4.951499	0.818644
1	-2.443543	-2.581010	-2.791121
1	-3.551865	-4.287716	-1.356991
8	1.536180	-2.335588	1.710178
8	2.319946	-2.148006	-0.705468
16	1.506050	0.815312	-0.948271
8	1.062216	0.252564	-2.222835
8	1.048357	2.135621	-0.508392
6	3.291892	0.808042	-0.905640
6	3.940711	1.719611	-0.066030
6	3.993178	-0.044896	-1.761735
6	5.336525	1.749163	-0.064009
1	3.360017	2.400917	0.555145
6	5.388149	0.002185	-1.750180
1	3.450182	-0.732877	-2.407560
6	6.057494	0.889705	-0.900437
1	5.861025	2.452928	0.586317
1	5.954186	-0.660358	-2.408780

1	7.149959	0.917666	-0.895672
6	-4.156826	4.004144	-1.313493
6	-2.775968	3.779285	-1.335275
6	-2.238367	2.632773	-0.750771
6	-3.077951	1.689128	-0.128890
6	-4.466314	1.921130	-0.111106
6	-4.997074	3.070560	-0.700447
1	-2.109036	4.498523	-1.817395
1	-1.161596	2.461859	-0.784575
1	-5.134133	1.207825	0.370182
1	-6.077190	3.236506	-0.677303
6	-3.365050	-0.753968	0.705397
1	-2.772429	-1.635224	0.994276
6	-2.495007	0.471822	0.495711
6	-1.238805	0.359866	0.880859
6	0.003897	0.223167	1.292818
6	0.470874	0.489064	2.704996
1	-0.371448	0.910116	3.276469
1	0.725891	-0.483427	3.160298
6	1.691473	1.412484	2.784416
1	2.522210	0.933779	2.241426
1	1.469786	2.354830	2.255895
6	2.116049	1.693024	4.225241
1	1.314675	2.199439	4.788784
1	2.356216	0.757878	4.758038
1	-4.576288	4.901450	-1.775777
1	3.008427	2.337894	4.262067
9	-4.294776	-0.526179	1.682888
9	-4.055741	-1.052620	-0.430660

Structure S11. **TS4**

E(B3LYP)sol = -3835.79300725 E(B3LYP)gas = -3832.48783284

1	0.245607	-0.272270	0.124301
7	0.677867	-1.086731	-0.334183
16	-0.189818	-2.543593	-0.206892
6	-1.503417	-2.513763	-1.414137
6	-2.766565	-2.071578	-1.020302
6	-1.233600	-2.910077	-2.726717
6	-3.781141	-1.991973	-1.973926
1	-2.942090	-1.781954	0.010569
6	-2.260330	-2.831534	-3.667957
1	-0.238657	-3.251096	-3.005058

6	-3.526707	-2.366419	-3.295909
1	-4.762597	-1.618391	-1.678088
1	-2.065496	-3.126686	-4.701571
1	-4.319963	-2.293754	-4.044232
8	-0.783328	-2.493418	1.133486
8	0.753647	-3.600855	-0.579987
16	1.703074	-0.659612	-1.632837
8	1.238037	-1.293342	-2.862323
8	1.846355	0.793914	-1.509030
6	3.267958	-1.412579	-1.200683
6	4.215945	-0.636348	-0.534118
6	3.505036	-2.749233	-1.533913
6	5.429262	-1.221886	-0.167917
1	4.002344	0.406934	-0.314358
6	4.720778	-3.322642	-1.156462
1	2.741813	-3.328439	-2.050902
6	5.678305	-2.564055	-0.472119
1	6.176829	-0.622090	0.356167
1	4.919906	-4.369480	-1.397156
1	6.626057	-3.022748	-0.178937
6	5.512524	3.283641	-0.122601
6	5.669536	2.448929	0.990068
6	4.549369	1.971038	1.669163
6	3.251971	2.343716	1.261929
6	3.106172	3.191358	0.146775
6	4.227588	3.647851	-0.542707
1	6.670105	2.163382	1.324280
1	4.667204	1.301777	2.523998
1	2.107449	3.441019	-0.209792
1	4.099309	4.279574	-1.425006
6	0.900520	2.564772	2.112410
1	0.835143	3.569705	1.692983
6	2.074931	1.832699	1.989214
6	2.116493	0.521036	2.513490
6	2.037093	-0.660135	2.810608
6	1.884117	-2.085247	3.054615
1	2.087834	-2.305833	4.119185
1	0.821856	-2.330268	2.878312
6	2.764878	-2.962599	2.142675
1	2.610523	-2.647849	1.102346
1	3.828137	-2.778856	2.371735
6	2.423135	-4.444337	2.269221
1	2.531023	-4.803282	3.307062
1	1.387537	-4.625881	1.942453

1	6.389526	3.643677	-0.666074
1	0.146183	2.267027	2.839041
1	3.080438	-5.052146	1.628202
9	-0.211195	1.539604	0.826265
7	-1.762302	0.701001	0.387616
16	-2.678046	1.176941	1.790348
6	-3.868372	-0.151701	1.799345
6	-5.095131	0.023586	1.152900
6	-3.516506	-1.347073	2.435545
6	-5.997216	-1.041812	1.147461
1	-5.313985	0.964620	0.649087
6	-4.430932	-2.401164	2.417477
1	-2.535616	-1.449123	2.899253
6	-5.665453	-2.248897	1.775320
1	-6.961575	-0.930909	0.646203
1	-4.171391	-3.348105	2.895598
1	-6.373825	-3.080900	1.758285
8	-3.359922	2.455626	1.580164
8	-1.780497	0.994246	2.930343
16	-2.159409	1.299142	-1.182427
8	-1.297766	0.539174	-2.082749
8	-3.622339	1.217629	-1.282808
6	-1.643380	3.005936	-1.235429
6	-2.544065	4.011600	-0.877726
6	-0.337693	3.279031	-1.657757
6	-2.114296	5.339495	-0.938715
1	-3.548631	3.751348	-0.547192
6	0.070123	4.612924	-1.716714
1	0.336364	2.460684	-1.918161
6	-0.812402	5.638707	-1.354813
1	-2.800782	6.142546	-0.660708
1	1.081659	4.853786	-2.052939
1	-0.483944	6.680196	-1.402663

Frequencies -- -440.8247
Red. masses -- 11.9933
Frc consts -- 1.3732
IR Inten -- 1697.4253

Structure S12. int3

E(B3LYP)sol = -3835.87897747 E(B3LYP)gas = -3832.57426962

1	-0.495421	-0.606541	0.077979
7	-0.649734	-1.653423	0.017661

16	-2.294869	-2.022005	0.311418
6	-3.224900	-1.366703	-1.057186
6	-3.528055	-0.004691	-1.064744
6	-3.572222	-2.212206	-2.113756
6	-4.190424	0.531492	-2.167461
1	-3.229360	0.629205	-0.235885
6	-4.248420	-1.664568	-3.204509
1	-3.303764	-3.267239	-2.081222
6	-4.550741	-0.297693	-3.233554
1	-4.397007	1.602968	-2.192834
1	-4.528502	-2.306850	-4.042706
1	-5.064428	0.124707	-4.100992
8	-2.603764	-1.244232	1.517118
8	-2.407554	-3.482657	0.320625
16	0.247007	-2.420797	-1.210581
8	-0.615953	-2.827468	-2.314353
8	1.410419	-1.548045	-1.420994
6	0.854474	-3.918176	-0.423590
6	2.146919	-3.925067	0.109336
6	0.041647	-5.054199	-0.388731
6	2.625094	-5.089366	0.714750
1	2.765040	-3.029304	0.053625
6	0.530066	-6.209788	0.225078
1	-0.958640	-5.021218	-0.816629
6	1.815261	-6.228158	0.778070
1	3.632489	-5.105944	1.137434
1	-0.099090	-7.101761	0.269498
1	2.189695	-7.136856	1.256035
6	5.970905	0.063069	-1.198840
6	4.774077	-0.650517	-1.049867
6	3.803898	-0.191732	-0.171440
6	3.997827	1.010352	0.559792
6	5.194090	1.750836	0.356835
6	6.175384	1.259065	-0.496365
1	4.584619	-1.555679	-1.630462
1	2.855675	-0.721932	-0.091810
1	5.354473	2.681142	0.894685
1	7.105524	1.816498	-0.626122
6	2.774978	2.775779	2.060653
1	2.818394	2.745555	3.164840
6	2.989284	1.396248	1.513744
6	2.098046	0.460122	2.003650
6	1.266364	-0.275691	2.532544
6	0.291765	-1.139811	3.137246

1	0.059043	-0.747672	4.143923
1	-0.641274	-0.947837	2.569992
6	0.626377	-2.640215	3.138874
1	0.922394	-2.933818	2.123394
1	1.499143	-2.822541	3.789412
6	-0.567533	-3.483955	3.582468
1	-0.910028	-3.200690	4.591661
1	-1.412111	-3.357064	2.889396
1	6.743225	-0.303988	-1.879940
1	1.732654	3.067481	1.789126
1	-0.302795	-4.552465	3.599634
9	3.650098	3.728116	1.596706
7	-0.626180	1.181113	0.222980
16	-0.970585	2.218004	1.448100
6	-2.696633	2.706729	1.329757
6	-3.049653	3.887830	0.674047
6	-3.655025	1.863246	1.900423
6	-4.401772	4.225225	0.582824
1	-2.275649	4.512809	0.231839
6	-5.003947	2.211903	1.796481
1	-3.343951	0.941407	2.392495
6	-5.376872	3.388957	1.138749
1	-4.694885	5.144569	0.069952
1	-5.764781	1.557616	2.228827
1	-6.433646	3.656958	1.058864
8	-0.168481	3.469695	1.360008
8	-0.850365	1.451842	2.700853
16	-0.586830	1.702193	-1.350026
8	-0.768625	0.490598	-2.163170
8	-1.473009	2.857376	-1.575862
6	1.098886	2.269015	-1.662885
6	1.516189	3.519497	-1.190865
6	1.938790	1.460508	-2.431937
6	2.813015	3.951558	-1.475007
1	0.834818	4.129096	-0.598738
6	3.227863	1.915723	-2.725492
1	1.581834	0.488115	-2.771784
6	3.666819	3.153905	-2.245987
1	3.156112	4.915394	-1.092704
1	3.894881	1.293099	-3.326029
1	4.678023	3.499573	-2.474620

Structure S13. **TS5**

E(B3LYP)sol = -3835.86222324 E(B3LYP)gas = -3832.56497497

1	0.409847	-0.357125	-0.007538
7	-0.218515	-1.300056	0.112066
16	0.954751	-2.646811	0.169287
6	2.165023	-2.223325	1.396208
6	3.284400	-1.493780	0.988362
6	1.972053	-2.619342	2.722904
6	4.219961	-1.114470	1.948635
1	3.411561	-1.214916	-0.053074
6	2.924921	-2.241426	3.666787
1	1.090746	-3.190261	3.005900
6	4.036266	-1.481545	3.284056
1	5.077019	-0.511595	1.645840
1	2.790357	-2.530479	4.711464
1	4.764364	-1.170026	4.037114
8	1.582738	-2.585963	-1.151328
8	0.214210	-3.836408	0.595626
16	-1.344506	-1.120445	1.459866
8	-0.747044	-1.656385	2.676105
8	-1.783515	0.272825	1.358597
6	-2.730605	-2.175014	1.036334
6	-3.864905	-1.582006	0.480782
6	-2.682237	-3.539360	1.338247
6	-4.965285	-2.384207	0.173510
1	-3.885508	-0.508252	0.309159
6	-3.787891	-4.330298	1.019865
1	-1.790065	-3.970176	1.788506
6	-4.922784	-3.758113	0.432712
1	-5.857626	-1.928202	-0.261901
1	-3.762665	-5.400809	1.235799
1	-5.783144	-4.386053	0.188008
6	-6.123632	2.319159	-0.161645
6	-5.853382	1.293983	-1.075713
6	-4.542947	1.056031	-1.485745
6	-3.485949	1.854423	-1.001824
6	-3.771693	2.902947	-0.103175
6	-5.080408	3.116359	0.324323
1	-6.667039	0.680847	-1.470692
1	-4.324008	0.256322	-2.196321
1	-2.961691	3.523499	0.277164
1	-5.289870	3.914835	1.039914
6	-1.123865	2.700029	-1.615813
1	-0.491705	2.490072	-2.491150

6	-2.104253	1.572767	-1.408871
6	-1.686893	0.305933	-1.584186
6	-1.254766	-0.867581	-1.716224
6	-1.042892	-2.036990	-2.565068
1	-1.403682	-1.700143	-3.556322
1	0.037496	-2.208796	-2.669022
6	-1.767352	-3.322580	-2.143006
1	-1.421328	-3.615092	-1.143325
1	-2.848456	-3.123792	-2.052816
6	-1.502495	-4.464638	-3.121295
1	-1.839653	-4.215880	-4.141738
1	-0.424992	-4.691935	-3.169107
1	-7.149747	2.501653	0.167141
1	-0.458151	2.745208	-0.738306
1	-2.024956	-5.381684	-2.807911
9	-1.757713	3.912043	-1.770599
7	1.336410	0.824068	-0.217612
16	1.970360	1.302262	-1.671958
6	3.579133	0.522330	-1.848160
6	4.705049	1.125725	-1.279311
6	3.663499	-0.689025	-2.540356
6	5.938013	0.478939	-1.387778
1	4.602197	2.066744	-0.741429
6	4.904250	-1.323488	-2.641445
1	2.764076	-1.128417	-2.968783
6	6.037897	-0.744147	-2.062240
1	6.826321	0.933682	-0.942357
1	4.981664	-2.276640	-3.169758
1	7.005713	-1.246411	-2.138516
8	2.182653	2.759776	-1.703206
8	1.099341	0.700470	-2.699918
16	1.798642	1.511709	1.226946
8	1.296648	0.579485	2.246967
8	3.218757	1.897608	1.222820
6	0.835253	3.024610	1.392345
6	1.287860	4.205589	0.794624
6	-0.368777	2.971990	2.100461
6	0.493121	5.350503	0.883894
1	2.227573	4.208311	0.244372
6	-1.145512	4.130944	2.193037
1	-0.690519	2.032124	2.547019
6	-0.721188	5.314509	1.579252
1	0.824326	6.275786	0.406639
1	-2.087298	4.104669	2.747199

1	-1.337183	6.215132	1.644616

Frequencies -- -265.6259
Red. masses -- 8.6665
Frc consts -- 0.3603
IR Inten -- 422.9200

Structure S14. 3

E(B3LYP)sol = -2219.58751812 E(B3LYP)gas = -2217.59641458

7	0.872752	-0.246044	0.353173
16	1.211302	-1.928120	0.324798
6	-0.319369	-2.687960	-0.222599
6	-0.886101	-3.684423	0.575732
6	-0.871515	-2.313441	-1.452088
6	-2.042924	-4.327459	0.124796
1	-0.425509	-3.939072	1.531327
6	-2.032850	-2.958740	-1.881314
1	-0.411979	-1.519890	-2.044727
6	-2.614067	-3.964991	-1.099158
1	-2.500562	-5.109013	0.735664
1	-2.485944	-2.673037	-2.833468
1	-3.521660	-4.465940	-1.444850
8	1.439078	-2.329916	1.711774
8	2.236237	-2.120879	-0.697040
16	1.361849	0.825338	-0.941278
8	0.931060	0.253655	-2.217055
8	0.877550	2.136309	-0.502668
6	3.147857	0.856598	-0.900436
6	3.777878	1.786492	-0.066649
6	3.866750	0.014781	-1.753008
6	5.172778	1.846195	-0.066363
1	3.183247	2.458479	0.551427
6	5.260378	0.091978	-1.743318
1	3.338111	-0.687820	-2.394916
6	5.911352	0.998108	-0.898941
1	5.682529	2.564606	0.579674
1	5.839889	-0.561550	-2.399226
1	7.002976	1.049643	-0.895510
6	-4.384714	3.824475	-1.377186
6	-2.993752	3.672391	-1.337514
6	-2.421732	2.555948	-0.728455
6	-3.236110	1.566038	-0.144422
6	-4.634316	1.727129	-0.186583

6	-5.200404	2.848129	-0.799472
1	-2.345740	4.426550	-1.791458
1	-1.336906	2.440790	-0.718394
1	-5.281412	0.986397	0.283835
1	-6.287700	2.959354	-0.819114
6	-3.479249	-0.882635	0.664886
1	-2.859418	-1.732353	0.982708
6	-2.632799	0.363934	0.491375
6	-1.377731	0.301781	0.891442
6	-0.130155	0.203155	1.300488
6	0.338323	0.489427	2.708091
1	-0.511219	0.895015	3.280055
1	0.616258	-0.473886	3.169893
6	1.538013	1.440238	2.778901
1	2.377602	0.978079	2.234964
1	1.293610	2.375010	2.246944
6	1.962298	1.737067	4.216572
1	1.151967	2.228056	4.780999
1	2.225013	0.809978	4.752879
1	-4.829755	4.699691	-1.857599
1	-3.972007	-1.135568	-0.289247
1	2.840351	2.401749	4.247226
9	-4.471095	-0.683010	1.619293

Structure S15. TS1'

E(B3LYP)sol = -2219.44114674 E(B3LYP)gas = -2217.43602039

9	0.249052	-1.570801	0.339614
7	0.751140	0.113633	-0.105386
16	2.088792	0.550243	0.799755
6	3.446086	-0.413127	0.154239
6	3.642025	-1.703353	0.656055
6	4.242134	0.119771	-0.862505
6	4.670486	-2.479981	0.119823
1	2.997167	-2.075162	1.453097
6	5.268990	-0.669513	-1.385544
1	4.040257	1.122457	-1.238799
6	5.481634	-1.963550	-0.897742
1	4.842154	-3.490367	0.498889
1	5.903143	-0.271999	-2.181433
1	6.285745	-2.575586	-1.314207
8	1.810025	0.094330	2.163056
8	2.328022	1.976062	0.536080

16	0.488138	0.456048	-1.743552
8	1.692086	1.027219	-2.354678
8	-0.159893	-0.713527	-2.334936
6	-0.733730	1.749755	-1.532976
6	-2.087185	1.407474	-1.553085
6	-0.292398	3.044047	-1.246338
6	-3.029663	2.403138	-1.283980
1	-2.386709	0.379315	-1.763074
6	-1.247879	4.027092	-0.980147
1	0.777206	3.255564	-1.200795
6	-2.611312	3.707899	-0.998040
1	-4.093833	2.155407	-1.293468
1	-0.925900	5.045563	-0.750221
1	-3.352729	4.481510	-0.782631
6	-4.831464	-2.013379	-1.569323
6	-5.127754	-1.845894	-0.210591
6	-4.118294	-1.961803	0.743329
6	-2.801185	-2.280159	0.353813
6	-2.508772	-2.436124	-1.017878
6	-3.519625	-2.296343	-1.968070
1	-6.148724	-1.615744	0.103700
1	-4.341131	-1.813821	1.802468
1	-1.476185	-2.565417	-1.342353
1	-3.272639	-2.384659	-3.028586
6	-0.564591	-3.111398	1.141352
1	-0.525316	-3.840446	0.331543
6	-1.723484	-2.357074	1.352327
6	-1.711356	-1.406156	2.395075
6	-1.426995	-0.393549	3.017918
6	-0.957195	0.881433	3.533909
1	-1.522912	1.184653	4.433081
1	0.097018	0.749055	3.829278
6	-1.044607	1.977994	2.440394
1	-0.685938	1.554527	1.491192
1	-2.102657	2.246796	2.281939
6	-0.209749	3.207194	2.789150
1	-0.490605	3.633521	3.767222
1	0.860335	2.948328	2.812055
1	-5.620677	-1.907651	-2.318098
1	0.167174	-3.202802	1.942957
1	-0.341181	3.989034	2.025012

Frequencies -- -486.9694

Red. masses -- 15.3984

Frc consts -- 2.1514
IR Inten -- 1405.4147

Structure S16. 10

E(B3LYP)sol = -2219.55295356 E(B3LYP)gas = -2217.56825384

9	0.713345	-1.469636	2.838978
7	0.405525	-0.338362	0.301737
16	1.821892	0.691291	0.074292
6	3.115557	-0.502557	-0.228357
6	3.327573	-1.509740	0.715907
6	3.918466	-0.346738	-1.357263
6	4.389728	-2.392115	0.510278
1	2.661881	-1.612992	1.574436
6	4.982564	-1.231934	-1.540294
1	3.690821	0.438102	-2.079355
6	5.216518	-2.250920	-0.610571
1	4.569994	-3.195439	1.228340
1	5.623707	-1.132173	-2.419224
1	6.045684	-2.946088	-0.764179
8	2.088085	1.305301	1.377057
8	1.647039	1.562158	-1.091087
16	-0.044988	-1.302700	-1.078130
8	1.044834	-1.114349	-2.033198
8	-0.384243	-2.646971	-0.625763
6	-1.508099	-0.510447	-1.732953
6	-2.653235	-1.281074	-1.935679
6	-1.443061	0.844420	-2.071916
6	-3.778761	-0.666973	-2.488934
1	-2.661609	-2.329168	-1.633815
6	-2.581368	1.444100	-2.612804
1	-0.525041	1.410559	-1.902410
6	-3.744357	0.691279	-2.820935
1	-4.689165	-1.249503	-2.647149
1	-2.559040	2.504107	-2.876637
1	-4.630906	1.169024	-3.245659
6	-4.290763	-2.228704	1.427325
6	-4.292782	-0.866629	1.119752
6	-3.093125	-0.152188	1.094634
6	-1.874506	-0.785218	1.374708
6	-1.881290	-2.153518	1.692356
6	-3.080036	-2.867140	1.714564
1	-5.229544	-0.353614	0.889076
1	-3.101096	0.907370	0.838975

1	-0.944847	-2.664314	1.899859
1	-3.064012	-3.933084	1.954301
6	0.121127	-0.216792	2.801549
1	-0.656069	-0.168396	3.579781
6	-0.564408	0.033548	1.422373
6	-0.835316	1.474820	1.317969
6	-0.890299	2.674736	1.165384
6	-0.800265	4.110765	0.921517
1	-1.463472	4.382028	0.079479
1	-1.181206	4.665348	1.798225
6	0.647568	4.545890	0.608208
1	1.289342	4.274798	1.462535
1	1.021347	3.948017	-0.238396
6	0.750431	6.039516	0.308218
1	0.137020	6.317017	-0.565913
1	0.404368	6.648047	1.161037
1	-5.227655	-2.791649	1.445241
1	0.897402	0.531826	2.983003
1	1.789983	6.330542	0.090596

Structure S17. **TS6**

E(B3LYP)sol = -2219.39967193 E(B3LYP)gas = -2217.40260656

9	1.342379	-1.518100	1.063329
7	0.779287	0.228382	0.393115
16	2.448019	0.750314	0.626567
6	3.653323	-0.387528	-0.047091
6	4.080792	-1.461041	0.739625
6	4.202999	-0.111874	-1.300199
6	5.090980	-2.283764	0.243853
1	3.605830	-1.643904	1.702133
6	5.213350	-0.948192	-1.781270
1	3.835891	0.732331	-1.881796
6	5.654799	-2.029745	-1.013108
1	5.438559	-3.130701	0.840209
1	5.653912	-0.752348	-2.761574
1	6.444032	-2.681892	-1.396037
8	2.628188	0.831013	2.072711
8	2.460720	1.980160	-0.180898
16	0.232662	-0.205857	-1.228236
8	1.375328	-0.016310	-2.117788
8	-0.497875	-1.464205	-1.203304
6	-0.921005	1.137869	-1.469405

6	-2.290821	0.882519	-1.373532
6	-0.411885	2.413656	-1.735977
6	-3.179287	1.948708	-1.536243
1	-2.648583	-0.127391	-1.177017
6	-1.314076	3.464708	-1.900271
1	0.667005	2.571436	-1.780051
6	-2.692393	3.234227	-1.795795
1	-4.253599	1.766598	-1.459281
1	-0.939826	4.470689	-2.103476
1	-3.391609	4.065011	-1.920051
6	-5.678612	-1.674923	-0.607951
6	-5.358306	-0.874527	0.493893
6	-4.121128	-1.016111	1.125814
6	-3.189391	-1.965555	0.671483
6	-3.517147	-2.760603	-0.440012
6	-4.754071	-2.616973	-1.072321
1	-6.074617	-0.135831	0.862519
1	-3.867479	-0.383952	1.980301
1	-2.776440	-3.457432	-0.836605
1	-4.990618	-3.232457	-1.943850
6	-1.183766	-3.251799	1.471681
1	-1.665250	-4.199479	1.219468
6	-1.842754	-2.070387	1.304044
6	-1.206410	-0.866191	1.633958
6	-0.544308	0.186195	1.790144
6	-0.356365	1.435577	2.552296
1	-1.104283	1.412963	3.364106
1	0.639589	1.415236	3.020389
6	-0.518204	2.723479	1.726967
1	0.261551	2.751464	0.953779
1	-1.485288	2.703145	1.196602
6	-0.412525	3.971456	2.601377
1	-1.195285	3.991883	3.378654
1	0.564375	4.014005	3.110578
1	-6.643850	-1.560847	-1.107704
1	-0.139937	-3.242782	1.788536
1	-0.515009	4.886823	1.997579

Frequencies -- -528.2098
Red. masses -- 11.1442
Frc consts -- 1.8319
IR Inten -- 260.3007

Structure S18. **11**

E(B3LYP)sol = -2219.60527889 E(B3LYP)gas = -2217.61861450

9	-0.596167	-1.762452	-1.250972
7	0.754300	-0.050636	0.255265
16	1.771716	-1.041638	1.240333
6	1.481537	-2.693656	0.624258
6	0.535493	-3.491995	1.272070
6	2.155641	-3.115659	-0.524717
6	0.250776	-4.752737	0.741902
1	0.040410	-3.124601	2.171889
6	1.861143	-4.379092	-1.038560
1	2.867500	-2.451669	-1.014771
6	0.909708	-5.192395	-0.411416
1	-0.485772	-5.393260	1.232954
1	2.372556	-4.727802	-1.938707
1	0.681480	-6.178154	-0.824645
8	1.217938	-0.955639	2.590412
8	3.145740	-0.640615	0.954329
16	1.345369	0.663409	-1.237245
8	2.244267	-0.281947	-1.892972
8	0.134055	1.153043	-1.898073
6	2.296771	2.082275	-0.705763
6	1.650079	3.318671	-0.609544
6	3.663373	1.937143	-0.451579
6	2.392812	4.434790	-0.219444
1	0.590085	3.398145	-0.849702
6	4.390588	3.064085	-0.063042
1	4.133086	0.959468	-0.544685
6	3.758316	4.306454	0.057761
1	1.903697	5.408322	-0.138462
1	5.458690	2.969102	0.145565
1	4.335359	5.182698	0.363722
6	-5.414786	2.154827	-0.353856
6	-5.775610	0.957665	0.272650
6	-4.918884	-0.144724	0.225014
6	-3.687960	-0.065862	-0.448330
6	-3.331806	1.143204	-1.075716
6	-4.192535	2.241274	-1.029675
1	-6.724481	0.884448	0.810250
1	-5.191298	-1.071332	0.736105
1	-2.372767	1.218114	-1.594254
1	-3.905091	3.172579	-1.524419
6	-3.220206	-2.485884	-0.791272
1	-4.281475	-2.681997	-0.959205

6	-2.777002	-1.240688	-0.526877
6	-1.323180	-1.014912	-0.398243
6	-0.662284	-0.177338	0.433787
6	-1.277318	0.640762	1.535012
1	-2.353765	0.432413	1.597613
1	-0.830558	0.299272	2.484091
6	-1.040374	2.149947	1.392182
1	0.043935	2.342366	1.432900
1	-1.382385	2.479170	0.397780
6	-1.755055	2.953223	2.477126
1	-2.847679	2.817345	2.413406
1	-1.436985	2.634724	3.483936
1	-6.082457	3.019142	-0.312497
1	-2.526100	-3.326226	-0.863162
1	-1.545377	4.030918	2.384852
